# Site-Specific and Programmable Editing of Serine and Threonine in Unprotected Peptides

**DOI:** 10.1021/jacs.6c09445

**Published:** 2026-07-09

**Authors:** Zhenquan Sun, Percy Man-Kit Liao, Adrian Kin Nam Chu, Alvin Wai Leung Lam, Yaoyue Zhang, Jie Yu, Xuechen Li

**Affiliations:** † Department of Chemistry, State Key Laboratory of Synthetic Chemistry, 25809 The University of Hong Kong, Pokfulam Road, Hong Kong, Hong Kong SAR 999077, P. R. China; ‡ Laboratory for Marine Drugs and Bioproducts, Qingdao Marine Science and Technology Center, School of Medicine and Pharmacy, Ocean University of China, Qingdao 266237, P. R. China; § Shanghai-Hong Kong Joint Laboratory in Chemical Synthesis, Shanghai Institute of Organic Chemistry, Chinese Academy of Sciences, University of Chinese Academy of Sciences, 345 Lingling Road, Shanghai 200032, P. R. China

## Abstract

Serine and threonine residues dominate regulatory post-translational modifications in nature, yet remain largely inaccessible to programmable chemical editing. Their weak hydroxyl nucleophilicity and the presence of multiple indistinguishable sites within proteins have prevented the development of general, site-selective strategies for modification in unprotected polypeptides. Here, we report that aminooxy serine or aminooxy threonine can mediate an effective aminooxy ligation (AOL) in aqueous solution through a transient 1,2,4-oxadiazinane intermediate, enabling chemoselective peptide ligation at the N-terminal position. Furthermore, the embedded aminooxy functionality after AOL subsequently undergoes a chemoselective ester ligation (CEL) with keto acids to restore aminooxy into native Ser/Thr and install O-acylation. This two-stage strategy offers site-specific Ser/Thr modification under mild conditions, permitting reductive restoration, neoglycosylation, or O-acylation. Applications range from late-stage modification of therapeutic peptide analogues to the convergent chemical synthesis of histone H2B bearing site-specific O-neoglycosylation and O-acylation. Together, this work establishes a general chemical framework for serine and threonine editing for protein chemical synthesis and engineering.

## Graphical abstract

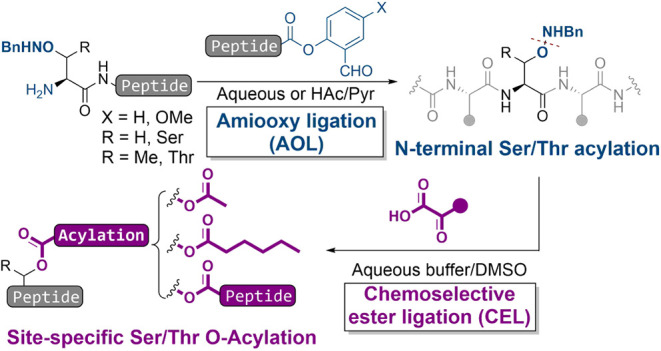

## Introduction

The ability to selectively synthesize and modify peptides and proteins with precision and simplicity underpins modern chemical biology, fundamental research, therapeutic development, and material innovations.^1^ Over the past few decades, chemoselective strategies have transformed the modification of cysteine, lysine, and tyrosine residues into routine operations.^2^ In striking contrast, analogous control over serine and threonine remains largely undeveloped, despite their central roles in biology (Figure 1A).^3^ Serine and threonine are among the most abundant residues in the proteome and serve as primary sites for phosphorylation and O-glycosylation.^4,5^ Proteomics data sets reveal that a substantial fraction of regulatory PTMs occurs on these hydroxyl-containing residues, underscoring their importance in signal transduction, chromatin regulation, and metabolic control.^6^ Yet, chemical methods for direct and site-selective modification of internal serine or threonine residues are still rare.^7^ This discrepancy reflects a fundamental paradox: residues that dominate regulatory PTMs in nature remain largely inaccessible to programmable chemical editing.

**Figure 1:**
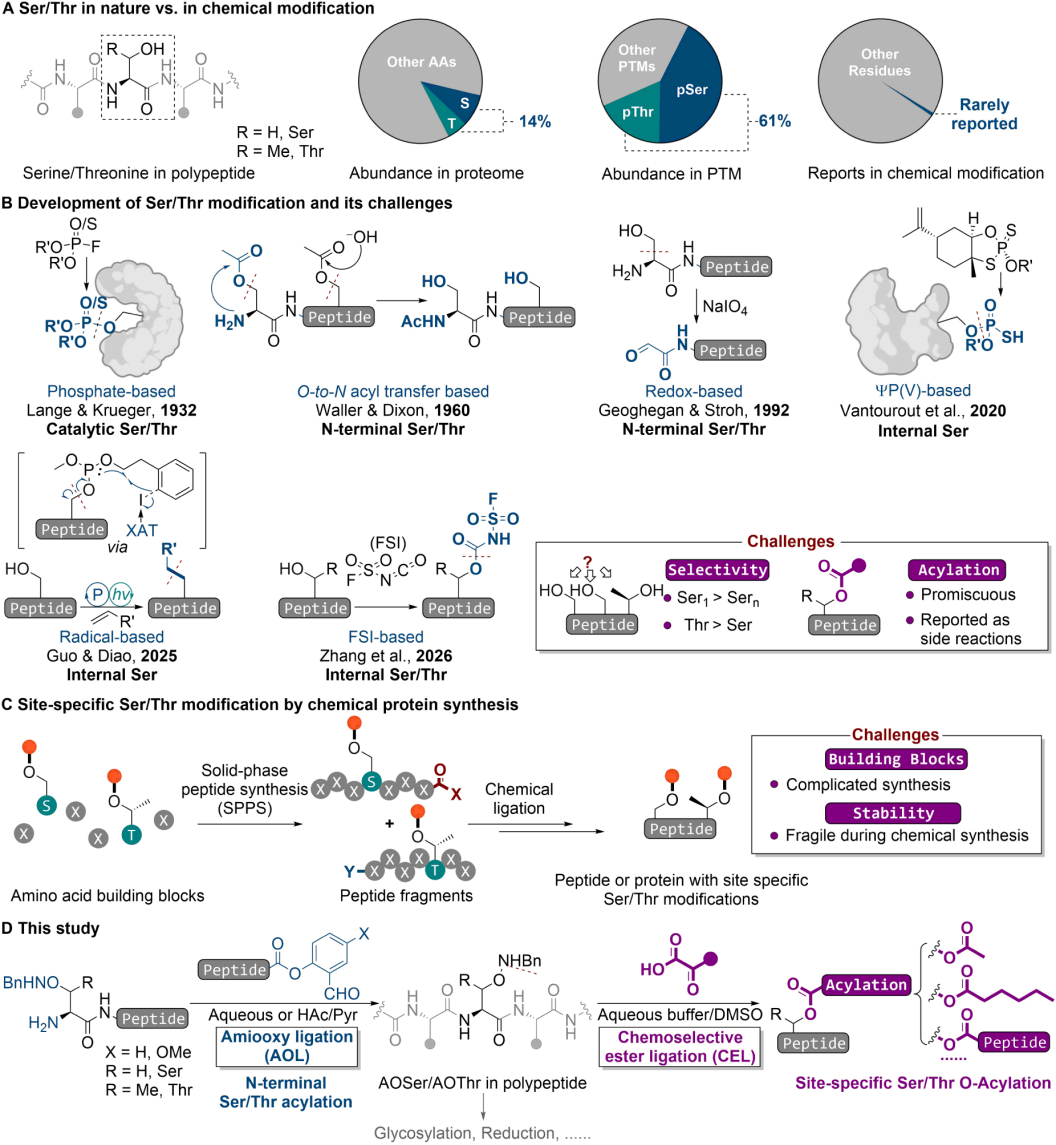
Ser/Thr selective modifications. A. Comparison of Ser modifications in nature and in chemical engineering; B. Development of Ser/Thr modifications and their challenges; C. Proposed chemoselective ester ligation and aminooxy ligation in this study.

The difficulty arises from intrinsic chemical constraints. The hydroxyl side chains of serine and threonine are weak nucleophiles under physiological conditions and are poorly differentiated from those of other polar residues within complex peptide environments.^8^ Early efforts to target catalytic serine residues were achieved through highly reactive electrophiles, such as organophosphates, first discovered nearly a century ago by Lange and Krueger (Figure 1B).^9^ Subsequently, an *O*-to-*N* acyl transfer chemistry was developed by Waller and Dixon in 1960 to differentiate the N-terminal Ser from the other internal ones through an irreversible acyl transfer step.^10^ Later, another N-terminal Ser differentiation method was established by Geoghegan and Stroh in 1992 via periodide oxidation to generate glyoxyamide for bioconjugation on peptides and proteins.^11^ In 2020, internal Ser bioconjugation was reported through psi-phosphate reagents by the Baran group.^12^ Recently, phosphite labeling of serine, followed by radical-based transformations, further expanded the repertoire of serine chemical modification by Guo and Diao in 2025.^13^ Very recently, the Chen group developed a fluorosulfuryl isocyanate (FSI) reagent for internal serine and threonine modification under hexafluoroisopropanol conditions, enabling subsequent SuFEx or deoxygenative elimination for further derivatization.^14^ However, the selectivity of one specific Ser over others requires sophisticated optimization and cannot be easily predicted. In addition, internal Thr-specific modification methods remain limited. More importantly, for O-acylation on Ser/Thr, it remains an untouched field since the developed acylation methods suffer from promiscuity and competing side reactions, making it nearly impossible to distinguish one serine or threonine residue from many.^15^

Chemical protein synthesis provides another way to access site-specifically modified peptide proteins in a bottom-up manner (Figure 1C).^3^ By combining solid-phase peptide synthesis^16^ and chemical ligation strategies^17^ with Ser/Thr building blocks bearing preinstalled modifications, peptides and proteins carrying tailor-made modifications can be obtained with atomic-level precision. However, these customized building blocks, such as those used for glycosylation, are complicated to prepare, and some modifications like acylation or phosphorylation, are also fragile during the process of chemical protein synthesis, which limits their applications.^18–20^ Consequently, a general and site-selective chemical strategy for better accessing peptides or proteins with designated serine or threonine modifications is still required. We reasoned that this long-standing limitation could be overcome not by forcing direct hydroxyl reactivity or synthesizing sophisticated building blocks but by temporarily replacing it with an easy-to-access surrogate that can be programmably converted back to Ser/Thr modifications at a later stage. This logic was inspired by the conceptual breakthrough of native chemical ligation (NCL)^17^ and desulfurization,^21^ in which Cys serves as a chemoselective handle for peptide ligation and is subsequently converted into native Ala residues through desulfurization. We envisioned that if a temporary and reactive serine/threonine surrogate could be developed for selective modification under mild conditions and restored back to native O-functionalized residues, site-specific Ser and Thr editing would become feasible.

Herein, we integrate protein chemical synthesis and late-stage modification by using aminooxy-substituted serine and threonine (AOSer/AOThr) as programmable surrogates to achieve synthetic proteins with Ser/Thr modifications (Figure 1D). AOSer/AOThr can mediate an N-terminal selective modification termed aminooxy ligation (AOL), enabling efficient peptide ligation in aqueous solution via a transient 1,2,4-oxadiazinane intermediate. Crucially, the embedded aminooxy group after AOL can then undergo a chemoselective ester ligation (CEL) with keto acids, resulting in direct O-acylation of serine and threonine. This transformation proceeds under mild aqueous conditions and furnishes native O-modified residues, thereby achieving traceless site-specific hydroxyl editing. We demonstrate these aminooxy-based strategies through applications ranging from model peptides and therapeutic analogues to the convergent chemical synthesis of histone H2B bearing well-defined O-neoglycosylation and O-acylation. Mechanistic studies confirmed the 1,2,4-oxadiazinane formation in AOL and unveiled the ester formation pathway in CEL. Together, this work establishes a general platform for serine and threonine modification in unprotected polypeptides, providing a new perspective on how hydroxyl residues can be programmed in protein synthesis and engineering.

## Results and Discussion

### AOSer/AOThr Mediates Selective N-Terminal Modification via 1,2,4-Oxadiazinane

With the bifunctional group at the N-terminus, the AOSer/AOThr peptide may undergo chemoselective N-terminal ligation with the C-terminal carboxylic surrogate of the other peptide, such as salicylaldehyde (SAL) esters, through aminooxy capture and 1,2,4-oxadiazinane formation.^22–25^ The aminooxy group, with stronger nucleophilicity than hydroxyl, may provide efficient modification even under aqueous conditions. As a proof of concept, the model peptide **1**, containing different C-terminal salicylaldehyde esters, was incubated with aminooxy peptide **2** under pH 4.5 citrate buffer with 6 M guanidine at room temperature (Figure 2A). Excitingly, the desired ligated product **3** could be observed under both aqueous and pyridine-acetic acid conditions. While only 27% of starting materials converted in aqueous conditions for R^1^ = H due to the hydrolysis of peptidyl salicylaldehyde ester **1**, clean and complete conversion of peptide **2** to **3** was observed in pyridine-acetic acid buffer (Figure 2A entries 1–2). Interestingly, the proposed intermediate 1,2,4-oxadiazinane **4** was also present but found unstable during the AOL, where it was formed in the first few hours under Pyr/HAc conditions and gradually decayed to the ligated product **3**, accompanied by the increasing SAL peak (Figure 2B).

**Figure 2:**
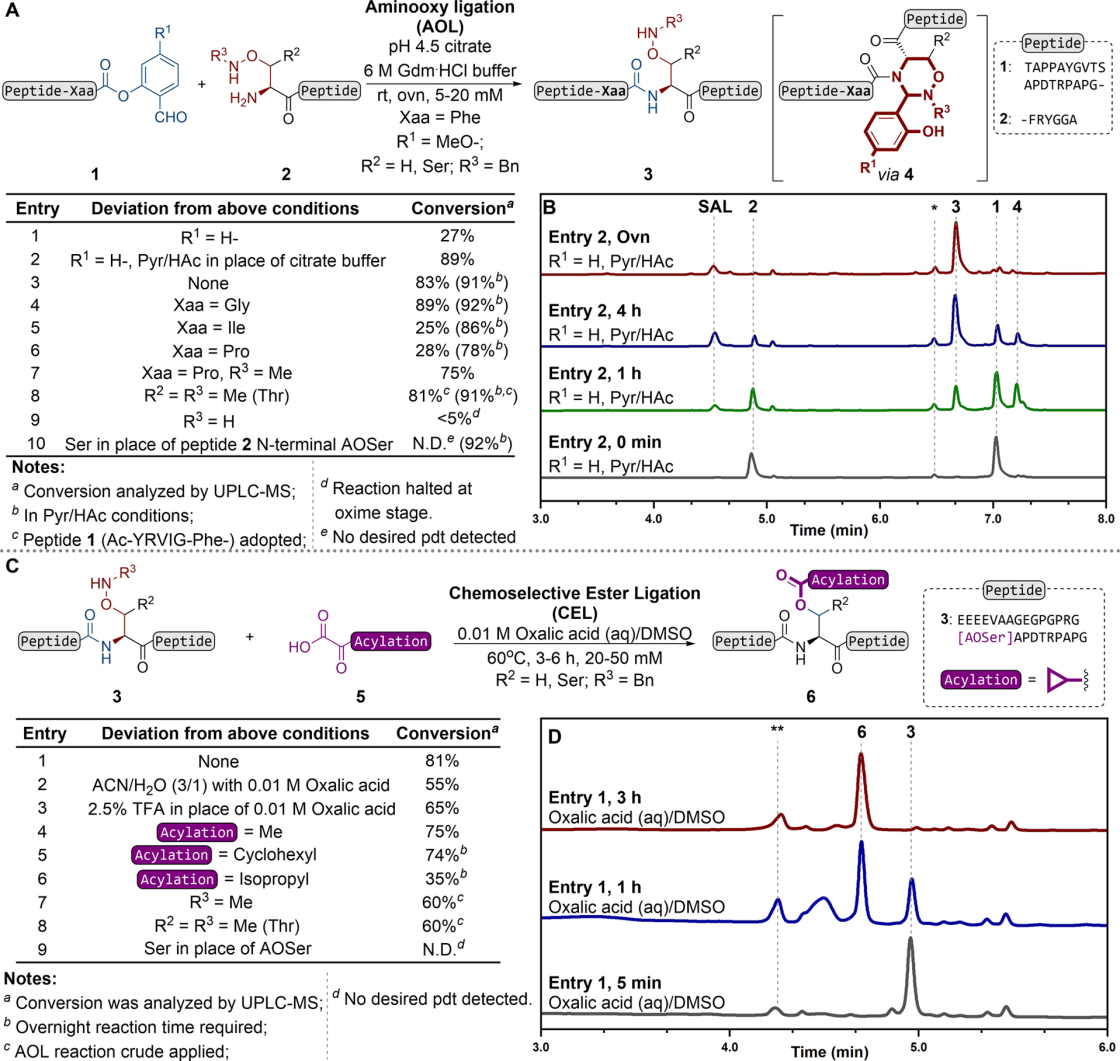
Discovery of untouched reactivity of aminooxy serine/threonine (AOSer/AOThr) to mediate aminooxy ligation (AOL) and chemoselective O-ester ligation (CEL). A. Condition optimizations of AOL under aqueous conditions; B. Representative UPLC spectra of AOL at different time points showing the formation of the semistable AOL intermediate, 1,2,4-oxadiazinane **4**; C. Condition optimizations of CEL under aqueous DMSO conditions; D. Representative UPLC spectra of CEL at different time points. **SAL**: salicylaldehyde released from AOL. *: Hydrolyzed SAL ester **1**. **: Deamination side product.

Due to the rapid hydrolysis of native SAL ester in aqueous conditions, a more stable *p*-methoxy salicylaldehyde was adopted as R^1^, and, pleasingly, a dramatic boost in AOL from 27% to 83% was achieved under aqueous citrate conditions (Figure 2A entry 3). Faster and higher conversion was realized when Xaa = Gly was applied (Figure 2A, entry 4). For those with bulky side chains, such as Ile or Pro, the AOL efficiency decreased in aqueous conditions, and Pyr/HAc conditions were recommended for these cases (Figure 2A entries 5–6). This difference may indicate that the acyl transfer step is the rate-determining step in AOL. We hypothesized that with faster formation of **4**, AOL at the sterically hindered site could be further improved. Indeed, a simple substitution of R^3^ with a methyl group led to around a threefold improvement at the Pro site under citrate buffer conditions (Figure 2A entries 6–7). Meanwhile, the AOL could also be applicable for AOThr (R^2^ = Me, Figure 2A entry 8). More importantly, the R^3^ substitution at the aminooxy group was crucial to mediate efficient peptide ligation, as the nonsubstituted one (R^3^ = H) could result in stable oxime formation, disfavoring the six-membered ring formation process (Figure 2A entry 9). Lastly, the aminooxy group at peptide **2** was important to carry out the AOL in citrate buffer (Figure 2A entry 10). The Ser/Thr peptide under citrate conditions remained intact, suggesting the compatibility of aqueous AOL with free, unprotected N-terminal Ser/Thr, which was further demonstrated in the following scope and limitation section. These studies indicated that AOL could be adopted as an effective strategy for chemoselective N-terminal modification of unprotected peptides under aqueous conditions.

### AOSer/AOThr Undergoes Traceless Ser/Thr O-Acylation

Inspired by the ketoacid hydroxyamine (KAHA) ligation, which can selectively cleave the N–O bond of hydroxyamine to generate an amide product with ketoacid through dicarboxylic rearrangement,^26^ we envisioned that the native Ser/Thr residue could be restored by reacting AOSer/AOThr with ketoacid in a similar manner. To our surprise, under KAHA conditions together with ketoacid **5**, an O-cyclopropoylation ester product **6** was observed from peptide **3** in large excess compared to the deamination product that was expected to form exclusively (Figure 2C,D). The ester bond nature was further confirmed by the complete removal of the O-cyclopropoylation in **6** under pH 10 conditions. These interesting results may imply an efficient chemistry to achieve postmodified Ser/Thr O-acylation in a site-precision manner, which we termed chemoselective ester ligation (CEL). The aqueous 0.01 M oxalic acid/DMSO at 60 °C was optimal for CEL among several other conditions varying the solvents and additives investigated (Figure 2C entries 2–3). Other ketoacids, such as pyruvic acid and cyclohexyl keto acid, also gave the ester product in CEL with 75% and 74% conversion, respectively, albeit bulkier 3-methyl-2-ketobutanoic acid resulted in 34% O-acylated product (Figure 2C entries 4–6). Furthermore, the crude reaction mixture from AOL could be directly applied for CEL without purification, and it was fully compatible with different substituted aminooxy groups as well as AOThr peptides (Figure 2C entries 7–8). Replacing AOSer with Ser residue demolished the ester conversion, as expected (Figure 2C entry 9). These data suggest that AOSer/AOThr can undergo chemoselective N–O cleavage and O-acylation via CEL. Combining CEL with AOL developed above may provide a new platform to achieve site-specific and traceless modification of Ser/Thr on unprotected peptides.

### Scope and Limitations of AOL and CEL

With the above preliminary studies, we were excited to examine the scope and limitations of AOL and CEL (Figure 3A). For AOL, a systematic investigation was carried out to evaluate various peptide substrates **1** and **2** to generate ligated peptides **3** (Figure 3A,B). Generally, all ligation products **10**-**27** were obtained smoothly in decent HPLC yields with a flexible aminooxy group as well as different SAL ester C-terminal amino acid residues (33–64%). For example, the corresponding peptide **2** containing N-terminal AOSer ligated with peptide SAL ester **1** cleanly under pH 4.5 citrate buffer conditions to produce peptide **23** with a 58% isolated yield, as shown in the representative UPLC spectra in Figure 3B. Furthermore, AOL was orthogonal to other chemical ligations, such as STL. In the synthesis of peptide **11**, the peptidyl SAL ester bearing an N-terminal serine was chemoselectively ligated to the N-terminal AOSer of the other substrate peptide exclusively under aqueous conditions, demonstrating the tolerance of N-terminal Ser/Thr in AOL and omitting its protecting group during the tandem ligation strategy for convenient peptide and protein assembly. This broad substrate scope highlights the robustness and adaptability of aminooxy ligation for peptide N-terminal modifications.

**Figure 3:**
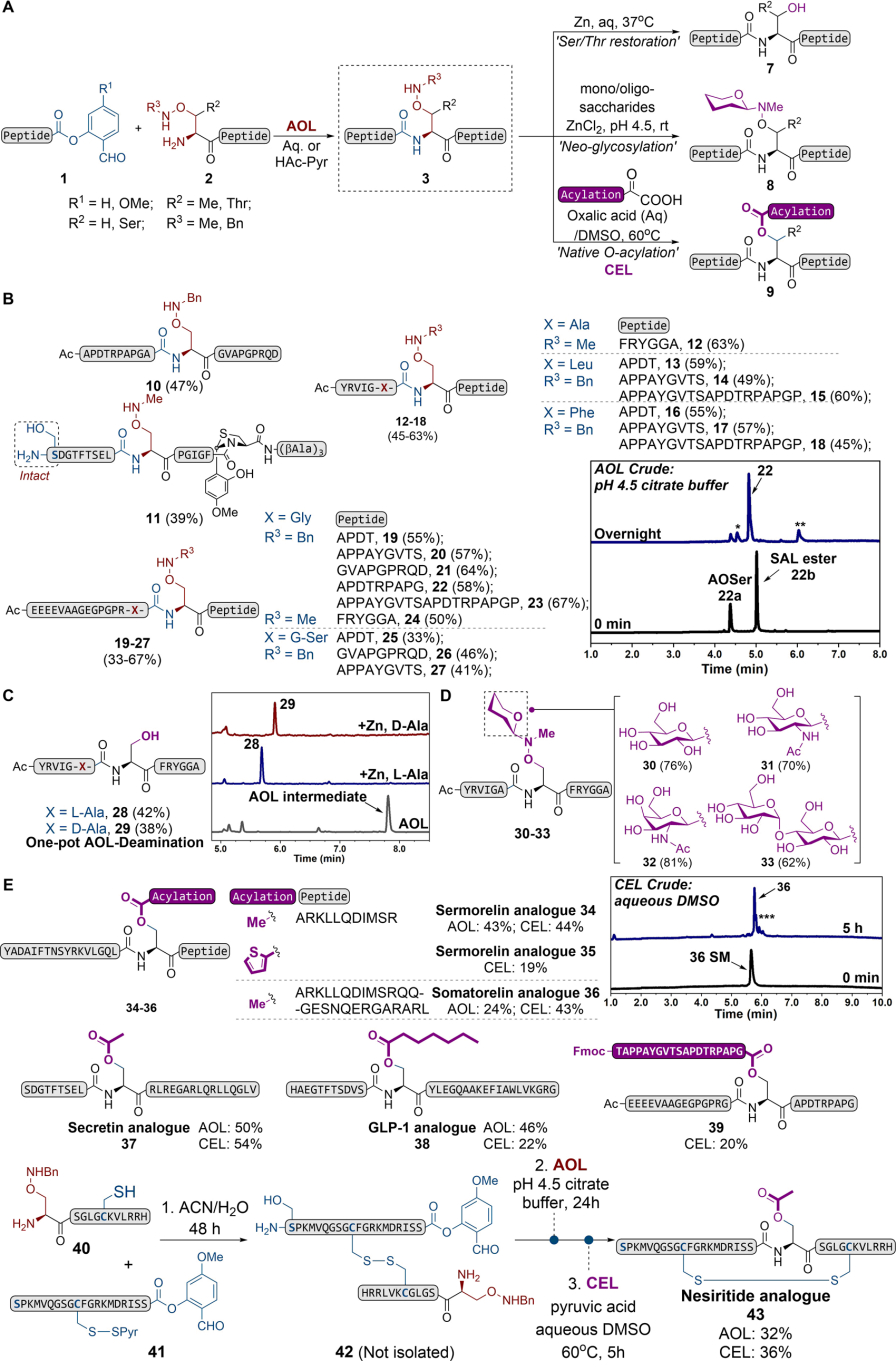
Scope and limitation of AOL and CEL. A. Overall scheme of AOL and postligational modifications, including CEL; B. Peptide substrate scope of AOL and reaction monitoring spectra of **23** by UPLC; C. Restoration of AOSer/Thr into Ser/Thr via reductive deamination by Zn, compatible with AOL crude in one pot; D. Selective neo-glycosylation at the AOSer site; E. Application of CEL on the chemoselective post-modification of peptide drugs and long-chain peptide esterification. *: Hydrolyzed SAL ester. **: SAL. ***: Deamination side product.

Subsequently, we continued to explore other modifications of aminooxy peptides, including the Zn-reduction of AOSer/AOThr into native serine and threonine, neo-glycosylation, and CEL discovered above (Figure 3C–3E). Due to the relatively weak N–O bond at the aminooxy group, it could be feasibly cleaved by metal-mediated reduction. Here, we found that in the presence of zinc in an aqueous ascorbate solution at 37 °C,^27^ the AOSer/AOThr could be successfully restored into Ser or Thr. Pleasingly, such reduction could also be applied to AOL crude in one pot, where the AOL intermediates were directly reduced into peptides **28** and **29** respectively with 38–42% isolated yield (Figure 3C). Meanwhile, the clean formation of products **28** and **29** with l-Ala and d-Ala at the C-terminus of the AOL site suggested that no detectable epimerization occurred during the ligation and Ser/Thr restoration.

In addition, the aminooxy group could also be utilized as a unique handle for site-specific bioconjugations, such as neo-glycosylation.^28^ Under pH 4.5 citrate buffer conditions with ZnCl_2_ as a catalyst, aminooxy-mediated β-selective glycosylation was carried out to generate the neoglycopeptides **30**–**33** with different mono/oligosaccharides at 62–81% HPLC yield (Figure 3D). ZnCl_2_ was found to be crucial for enhancing the neoglycosylation efficiency, probably through the activation of the glycan as well as the chelation of the aminooxy group. Such a method under mild conditions may enable precise postglycosylation to generate peptides and proteins with well-defined oligosaccharide patterns, overcoming the difficulties of complicated glycan building block preparation and sophisticated handling afterward.

With the combination of AOL and CEL, we were able to synthesize clinically relevant peptide drugs with late-stage site-specific O-acylation (Figure 3E). Sermorelin and somatorelin are synthetic growth hormone-releasing hormone (GHRH) analogues that stimulate endogenous growth hormone secretion for both diagnostic and therapeutic applications, yet investigation into their internal serine residue modifications has been barely reported.^29,30^ CEL provides a feasible way to access Sermorelin and somatorelin analogues **34–36** bearing different O-modifications with clean conversion and HPLC yield, including acetylation (**34**, 44%; **36**, 43%) and 2-thiophenecarboxylation (**35**, 19%). Secretin is another peptide hormone that stimulates pancreatic bicarbonate secretion and regulates gastric acid homeostasis, and it is clinically used in diagnostic testing of pancreatic exocrine function and certain endocrine tumors.^31^ O-acylated secretin analogue **37** was efficiently obtained through selective AOL in the presence of an N-terminal unprotected serine residue, followed by CEL with 50% and 54% isolated yield, respectively. As for GLP-1, it enhances glucose-dependent insulin secretion, inhibits glucagon release, slows gastric emptying, and promotes satiety, making it a key target for diabetes and obesity treatments.^32^ By CEL, an O-acylated analogue **38** with a medium carbon chain from 2-oxooctanoic acid could also be obtained in 22% HPLC isolated yield. This CEL method provides unique access to peptide drug analogues with different O-modifications to investigate their stability, half-life, and pharmacokinetic performance as novel therapeutic modalities. Strikingly, O-peptidylation was also achieved to generate peptide **39** with an O-link 20-mer MUC1 ester in 20% HPLC yield. This may also shed light on site-specific postmodified O-peptidylation, including ubiquitination, sumoylation, and other protein-size PTMs at unprotected peptides or proteins. Lastly, synthesis of Nesiritide by AOL and CEL was demonstrated. Nesiritide is a B-type natriuretic peptide used for the intravenous treatment of acute decompensated heart failure, where it promotes vasodilation, natriuresis, and reduction of cardiac preload and afterload to improve hemodynamic status.^33^ We designed and performed a proximity-induced intramolecular AOL through the disulfide bond formation between aminooxy peptide **40** and peptidyl SAL ester **41** to form **42**. Interestingly, we found part of the intermediate **42** had already undergone AOL under disulfide bond formation conditions, indicating the potential acceleration of AOL by tethering. Further incubation under citrate buffer advanced the AOL to completion, and the subsequent CEL with pyruvic acid afforded the Nesiritide analogue **43** with decent HPLC yields. This proximity-induced AOL method may enable chemoselective ligation under highly dilute conditions, which is suitable for synthesizing extremely hydrophobic proteins with poor solubility. Collectively, the above robust aminooxy-based strategies provide a general means to access site-specific Ser/Thr modifications on unprotected peptides, offering unexplored potential for their modification in peptide drug development, chemical biology research, and material science.

### Chemical Synthesis of Histone H2B with Site-Specific Glycosylation and Acylation

Histone H2B contains multiple serine and threonine residues, and their PTMs participate in complicated and dynamic PTM crosstalk, such as K-ubiquitination that regulates chromatin organization and DNA repair.^34^ We therefore targeted H2B S92GlcNAc and the dual S37/S92 O-acylation as representative glycosylation and acylation models to demonstrate the site-specific serine editing by AOL and CEL at the protein level (Figure 4).

**Figure 4:**
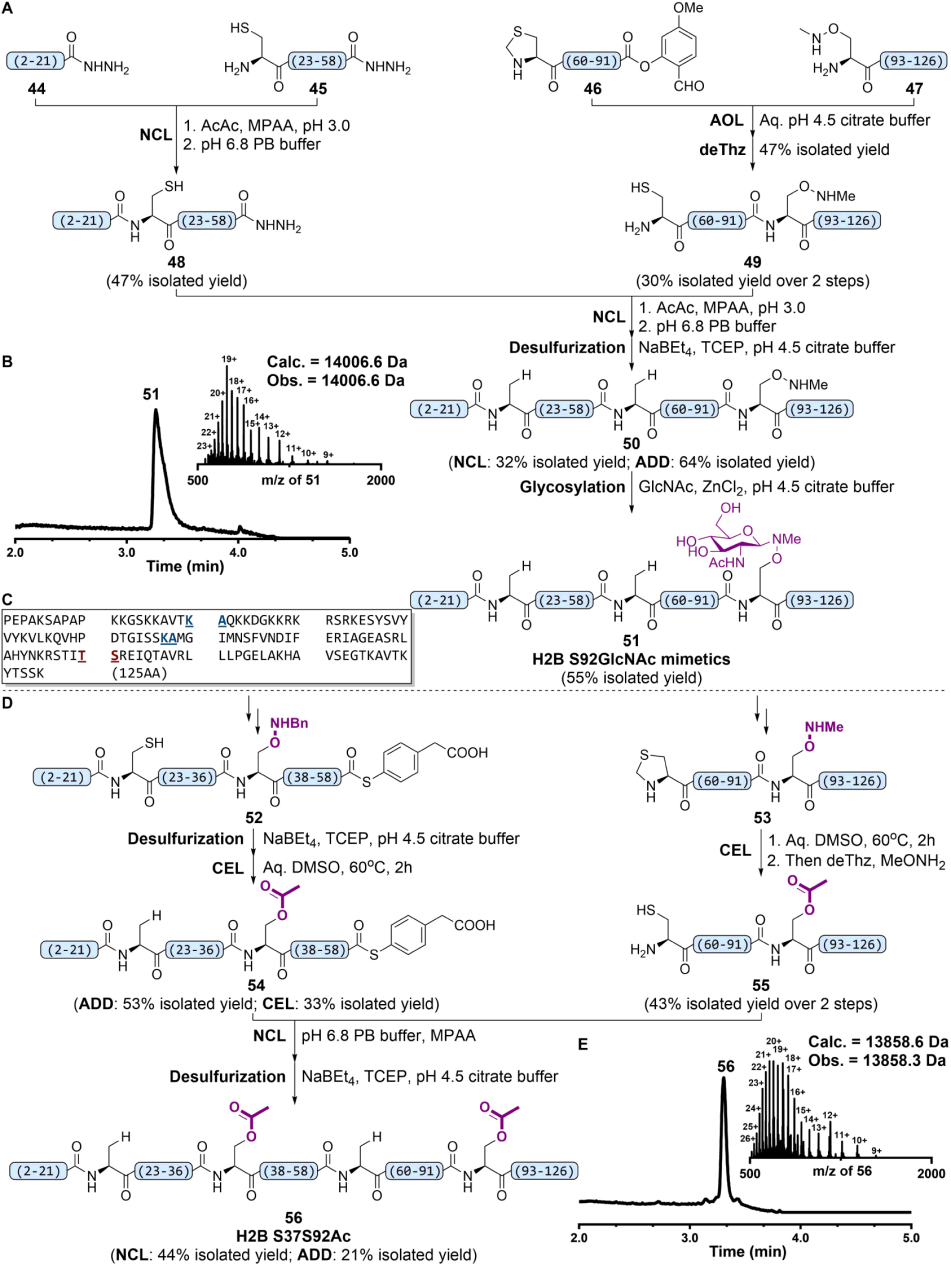
Chemical synthesis of histone H2B with site-specific O-glycosylation mimetic or O-acylation through AOL and CEL. A. Synthetic scheme of H2B S92GlcNAc mimetic **51**; B. UPLC-MS characterization of **51**; C. Amino acid sequence of **51**, with letters underlined in red or blue indicating the ligation sites; D. Synthetic scheme of H2B S37S92Ac **56**; E. UPLC-MS characterization of **56**.

First, the 125-AA H2B protein sequence was divided into four fragments to develop a convergent synthetic route, including peptidyl hydrazide **44**, **45**, peptidyl salicylaldehyde **46**, and N-terminal AOSer peptide **47**. Ala^22^ and Ala^59^ were temporarily mutated into Cys to enable the NCL (Figure 4A and 4C). Under activation by acetylacetone (AcAc),^35^ the peptidyl hydrazide **44** was converted into the peptide MPAA thioester under pH 3 conditions and then ligated with the N-terminal Cys of peptide **45** in pH 6.8 buffer conditions, generating ligated fragment **48** with a 47% isolated yield. Meanwhile, AOL was smoothly performed between the C-terminal SAL ester of **46** and the N-terminal AOSer of **47**, followed by the removal of Thz protection in one pot to produce ligation product **49** containing free N-terminal Cys in a 30% overall HPLC yield. With ligated fragments **48** and **49** in hand, we further carried out the final ligation to assemble them together, achieving a 32% isolated yield. NaBEt_4_-based add-and-done desulfurization (ADD)^36^ was performed as follows to restore all the mutated Cys into native Ala residues, yielding the H2B linear sequence **50** in a 64% isolated yield. It should be noted that the N–O bond at aminooxy serine can remain intact under ADD conditions, while under the conventional VA-044-mediated free radical desulfurization,^37^ messy results were observed, probably due to side reactions from the aminooxy group. Finally, ZnCl_2_-mediated glycosylation with N-acetylglucosamine (GlcNAc) was adopted to generate the H2B S92GlcNAc analogue **47** with a 55% isolation yield (Figure 4B).

Similarly, H2B with dual serine acetylation could be synthesized as well (Figure 4D and 4E). Peptidyl thioester **52** containing AOSer underwent desulfurization by ADD in 53% yield, followed by CEL to afford selectively O-acylated peptide **54** in 33% HPLC yield. On the other hand, the second CEL was carried out to install the acetylation on Ser^92^ of peptide **53** in aqueous DMSO conditions for 2 h. After Thz removal in one pot, fragment **55** was obtained in 43% isolated yield over two steps. At last, the linear H2B sequence was assembled by another NCL in 44% HPLC yield, and its Cys was restored back to Ala by ADD to produce the H2B S37S92Ac **56** successfully in 21% isolated yield (Figure 4E). In brief, convergent chemical syntheses of H2B proteins with homogeneous neoglycosylation or dual acetylation were developed in good yield, employing AOL and CEL together with NCL and NaBEt_4_-desulfurization. This strategy may provide efficient access to proteins with different O-modified PTMs by poststage modification of aminooxy peptide through CEL and others, omitting the need to prepare various preinstalled PTM building blocks and peptide fragments from scratch.

### Mechanistic Study of AOL and CEL

After examining the scope and application above, we further conducted a mechanistic study of AOL and CEL (Figure 5). For AOL, the proposed six-membered 1,2,4-oxadiazinane intermediate was confirmed by the detailed NMR characterization of a dipeptide model **61**, which was prepared from the AOL between SAL ester **57** and AOSer **58**, further stabilized by acetic anhydride treatment (Figure 5A). A twisted structure containing an *S*-configuration of the acetal, as well as a *trans* conformation of the amide bond, was identified by NOESY NMR spectroscopy. Interestingly, the ligation intermediate **59** was unstable and directly underwent spontaneous acidolysis to **60** under the HPLC conditions, which was different from the more stable oxazolidine and thiazolidine generated from STL^24^ and CPL.^38^ With the capping of the phenol group via acylation,^39–41^ the 1,2,4-oxadiazinane **61** could be prepared and isolated. More importantly, the six-membered ring formation was crucial for the following acyl transfer, and the direct aminolysis pathway, which could be present in imine-capture ligation,^22,42^ was also ruled out by the experiment of AOL between peptidyl SAL ester **62a** and unsubstituted aminooxy serine peptide **62b** (Figure 5B). Without the extra alkylation of the aminooxy group, the free aminooxy serine **62b** was rapidly conjugated to peptide **62a** through the formation of oxime **63** under pH 4.5 citrate buffer. After overnight incubation, oxime **63** remained the majority part of the reaction, and after zinc reduction, less than 5% of AOL product **64** was found. Hydrolyzed peptide SAL ester **65a** and deaminated AOSer peptide **66** were observed dominantly instead, with a detectable amount of amide **65b** as well, indicating the acyl transfer to the nitrogen of the aminooxy group. These data suggested the importance of 1,2,4-oxadiazinane formation in AOL to accelerate the ligated peptide formation.

**Figure 5:**
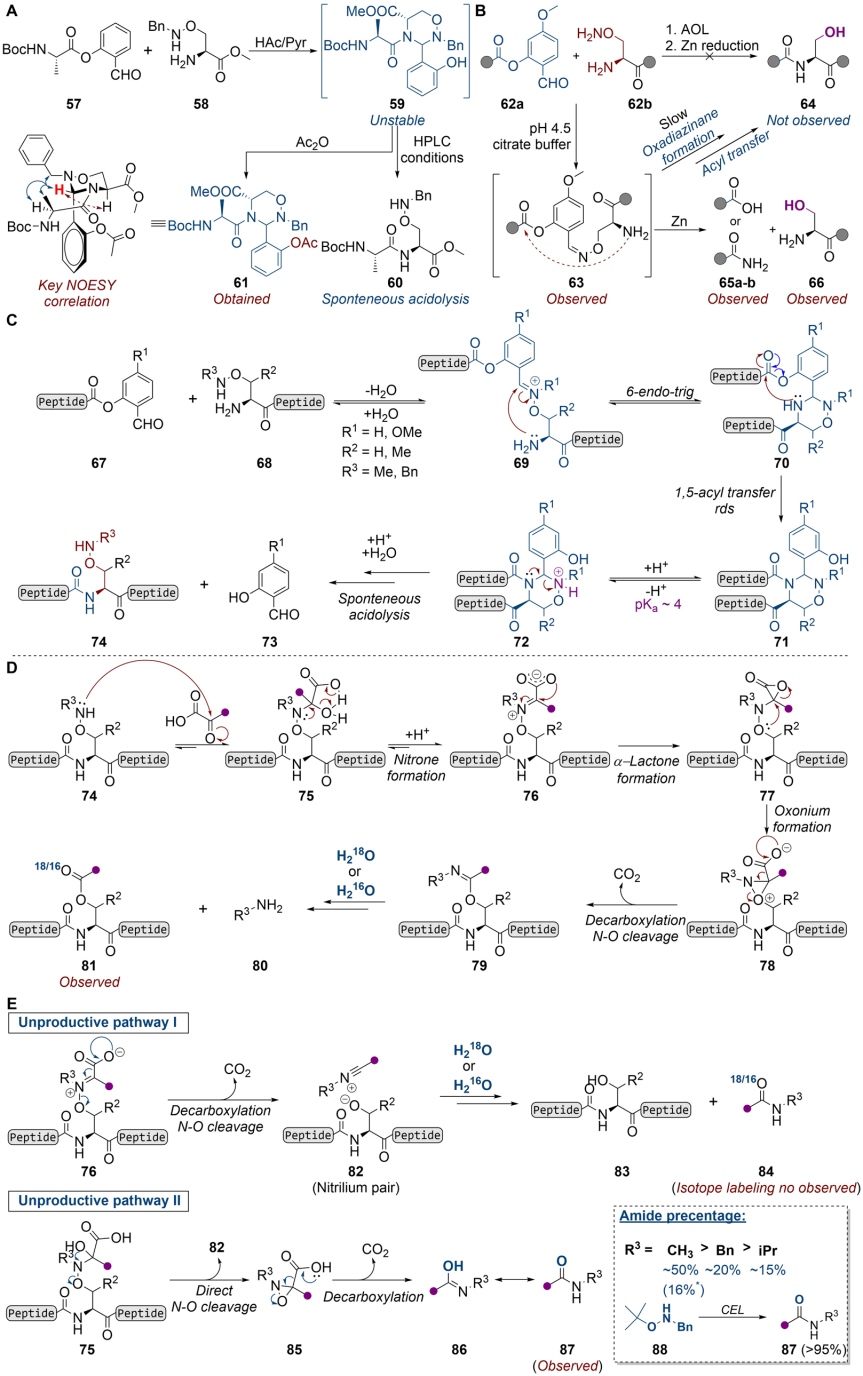
Mechanistic studies of AOL and CEL. A. Isolation and NMR analysis of the AOL intermediate, 1,2,4-oxadiazinane **61**. B. AOL with unsubstituted AOSer **62**. C. Proposed mechanism of AOL. D. Proposed mechanism of CEL and isotopic labeling with H_2_^16^O or H_2_^18^O. E. Comparison of two possible unproductive pathways for the generation of amide side products. *In the case of larger peptides, the amide percentage with R^3^ = CH_3_ is lower.

Combining these results, we proposed a detailed mechanism of AOL (Figure 5C). Initially, the aminooxy group of peptide **68** was captured by the SAL ester **67** to form intermediate **69** because of the stronger nucleophilicity of the aminooxy group than the N-terminal amine under pH 4.5 conditions. Enabled by the favorable *6-endo-trig* ring closure, 1,2,4-oxadiazinane **70** was then generated reversibly. Later, the proximity-induced irreversible 1,5-acyl transfer, which was believed to be the rate-determining step (RDS), occurred, giving the semistable AOL intermediate **71**. Subsequently, the aminooxy group could be protonated to form **72** under pH 4.5 conditions, triggering the following irreversible acidoylization process. Eventually, these two irreversible steps drove the AOL to produce SAL **73** and the ligated peptide **74** to completion.

As for CEL, its mechanism may be sophisticated (Figure 5D,E). Based on previous studies on KAHA ligation, the O-substituted hydroxylamine produced an amide product exclusively instead of giving an ester product.^26^ Although 5-oxoproline could convert into the ligated ester in type II KAHA ligation, the capture of the nitrilium intermediate by an intramolecular cyclic iminoether formation was identified as the key step.^43,44^ If following a similar mechanism, in CEL there would be an intermolecular process where it would be less likely to happen and would result in dominant amide formation as well (Figure 5E, unproductive pathway I). Therefore, we proposed an oxaziridine-mediated rearrangement process to explain the dominant ester formation in CEL (Figure 5D). First, the aminooxy peptide **74** was condensed with ketoacid to give nitrone **76** via a self-catalyzed dehydration of haminol **75**. Then, the formation of α-lactone **77** occurred,^45^ facilitating the conversion to oxaziridine **78** bearing an unstable oxonium. Such an unstable intermediate would undergo decarboxylation subsequently, resulting in the N–O cleavage to generate imidate **79**.

After hydrolysis, the ester product **81** was finally produced, accompanied by the amine **79** as a side product. The isotope labeling experiment using H_2_O and H_2_^18^O in the CEL reaction led to the observation of single ^18^O labeling ester, indicating that one of the oxygens came from the aminooxy group and the other came from the solvent H_2_O in the generated ester carbonyl group. Theoretically, if decarboxylation happened before the α-lactone **77** formation, peptide **76** could convert into the nitrilium pair **82**, which could further hydrolyze to ^18^O labeling **83**. Interestingly, we could not observe isotope labeling of these side products of CEL, implying that another unproductive pathway might exist. Instead, the direct N–O cleavage of the haminol intermediate **75** was regarded as the major unproductive pathway to generate **84** and oxaziridine **85**. After decarboxylation, the imidic acid **86** was formed and became the more stable amide form **87** through resonance without introducing external O from H_2_O. This pathway was supported by the increasing amide percentage in CEL with decreasing steric hindrance at the R^3^ site of the aminooxy group, as a less hindered R^3^ could facilitate the generation of **85**. Furthermore, when trifluoropyruvic acid was applied, exclusive amide formation was observed since the electron-withdrawing group hampered nitrone formation at haminol **75**, driving the production of **85** as well. A similar result was obtained if a bulky tBu group at the oxygen site of the aminooxy group was present, where **88** was only converted into amide. It should be noted that amide formation in CEL was also affected by the conformation of the peptide. For example, in the H2B synthesis, no amide product was found in the CEL steps even when methyl AOSer was adopted, indicating that on a protein scale, CEL might have better esterification efficiency.

## Conclusion

In conclusion, we developed the aminooxy ligation (AOL) and chemoselective ester ligation (CEL) based on AOSer/AOThr to achieve chemoselective and traceless Ser/Thr modification on unprotected peptides. Systematic investigations indicated a broad substrate scope, ranging from model peptides and therapeutic peptide drugs to proteins with precise PTM installation at a late stage. Subsequent mechanistic studies revealed the unique reactivity of aminooxy Ser/Thr in both the AOL and CEL. Future developments, including the incorporation of AOSer and AOThr into recombinant peptides and proteins by the ncAA expansion method, are in progress. This platform may open a new avenue for on-demand Ser/Thr modification on peptides and proteins, enabling exploration of their untapped potential in fundamental chemical biology research, therapeutic development, and novel materials discovery.

## Supplementary Material



## Notes

General information and additional experimental details; supporting figures and tables; NMR and HPLC spectra; peptide synthesis and related characterizations (PDF)

## ∥

Department of Medicine, The University of Chicago, 900 E. 57th Street, KCBD 4220A, Chicago, IL 60637, USA
