# Supplementary material for: Site-Specific and Programmable Editing of Serine and Threonine in Unprotected Peptides
Source: J Am Chem Soc. 2026 Jul 9;148(28):30418–28. doi: 10.1021/jacs.6c09445 (PMC13397880; doi:10.1021/jacs.6c09445)
Supplement: Supplementary file 1 [file ja6c09445_si_001.pdf]

Supporting Information

Site-specific and Programmable Editing of Serine and Threonine in Unprotected Peptides

Zhenquan Sun,[a, d, †] Percy Man-Kit Liao,[a, †] Adrian Kin Nam Chu,[a, †] Alvin Wai Leung Lam,[a] Yaoyue Zhang,[a] Jie Yu,[a] Xuechen Li*[a, b, c]

Content

1. General information ···· 3-9
2. Building block synthesis ···· 10-22
3. Discovery and development of AOL ···· 23-63
4. Discovery and development of CEL ···· 64-80
5. Scope and limitations of AOL and CEL ···· 81-156
6. Chemical synthesis of H2B ···· 157-186
7. Mechanistic study of AOL and CEL ···· 187-200
8. NMR spectra ···· 201-214
9. Reference ···· 215

1. General information

1.1. Materials and methods and abbreviations

All commercial materials (purchased from Aldrich, ChemImpex, Fluka and GL Biochem) were used without further purification. All solvents were reagent grade or HPLC grade (RCI or DUKSAN). Dry dichloromethane (CH2Cl_2) was distilled from calcium hydride (CaH2). All reversed-phase (RP) high-performance liquid chromatography (HPLC) separations involved a mobile phase of 0.1% trifluoroacetic acid (TFA) (v/v) in acetonitrile (CH_3CN)/0.1% TFA (v/v) in water (H2O) were performed with a Waters HPLC system equipped with a photodiode array detector (Waters 2996) using a Vydac 214TPTM C4 column (5 μm, 300 Å, 4.6 x 250 mm) at a flow rate of 0.6 mL/min for analytical HPLC and Vydac 214TPTM C4 column (10 μm, 300 Å, 22 x 250 mm) or Vydac 218TPTM C18 column (10 μm, 300 Å, 22 x 250 mm) at a flow rate of 10 mL/min for preparative HPLC. Low-resolution mass spectral (MS) analyses were performed with a Waters 3100 mass spectrometer using electrospray ionization (ESI, in positive mode unless otherwise specified). The results were analyzed with Waters Empower software. Calculated masses were based upon the most abundant isotope of a given ion. Analytical TLC was performed on E. Merck silica gel 60 F254 plates and visualized under UV light (254 nm) or by staining with ninhydrin or 5 % sulfuric acid in methanol. Silica flash column chromatography was performed on E. Merck 230-400 mesh silica gel 60. 1H and 13C nuclear magnetic resonance (NMR) spectra were recorded at 298 K on Bruker Avance DRX 300 FT-NMR Spectrometer at 75 MHz for 13C NMR or Bruker Avance DRX 400 FT-NMR spectrometer at 400 MHz for 1H NMR and 100 MHz for 13C NMR or Bruker Avance DRX 600 FT-NMR spectrometer at 150 MHz for 13C NMR. Chemical shifts are reported in parts per million (ppm)

and are referenced to solvent residual signals: $CDCl_3$ (δ 7.26 [1H]). 1H NMR data is reported as chemical shift (δ), relative integral, multiplicity (s = singlet, d = doublet, t = triplet, dd = doublet of doublet, td = triplet of doublet), coupling constant (J Hz). LCMS = Liquid chromatography mass-spectrometry; PG = protecting groups; SAL = salicylaldehyde; DMF = dimethylformamide; TIPS = triisopropylsilane; AO(Me) = Methyl substituted aminooxy group; AO(Bn) = Benzyl substituted aminooxy group.

1.2. Standard Protocol of Solid-Phase Peptide Synthesis (SPPS)

The solid phase peptide synthesis was carried out manually using 2-chloro-trityl resin (GL Biochem, loading capacity: 0.5 mmol/g). 2-chloro-trityl chloride resin was swollen in dry CH_2Cl_2 for 30 min and then it washed with CH_2Cl_2 (5 mL × 3). After that, a solution of Fmoc-Xaa-COOH (4.0 equiv. relative to resin loading capacity) and DIEA (8.0 equiv. relative to resin capacity) in CH_2Cl_2 was added and the resin was shaken at room temperature for 2 h to load the first amino acid. Then the resin was washed with DMF (5 mL × 3), CH_2Cl_2 (5 mL × 3) and subsequently treated with a solution of CH_2Cl_2/CH_3OH/DIEA (17:2:1, v/v/v, 5 mL) for 1 h to capping. The resin was washed with DMF (5 mL × 3), CH_2Cl_2 (5 mL × 3), and DMF (5 mL × 3). Finally, it was submitted to iterative peptide assembly (Fmoc-SPPS). The deblock solution was a mixture of 20/80 (v/v) of piperidine/DMF. The following Fmoc amino acids and Boc amino acids from GL Biochem were employed: Fmoc-Ala-COOH, Fmoc-Cys(Trt)-COOH, Fmoc-Asp(OtBu)-COOH, Fmoc-Phe-COOH, Fmoc-Gly-COOH, Fmoc-Glu(OtBu)-COOH, Fmoc-His(Trt)-COOH, Fmoc-Ile-COOH, Fmoc-Lys(Boc)-COOH, Fmoc-Leu-COOH, Fmoc-Met-COOH, Fmoc-Asn(Trt)-COOH, Fmoc-Pro-COOH, Fmoc-Gln(Trt)-COOH, Fmoc-Arg(Pbf)-COOH, Fmoc-Ser(tBu)-COOH, Fmoc-Thr(tBu)-COOH, Fmoc-Val-COOH, Fmoc-Trp(Boc)-COOH, Fmoc-Tyr(tBu)-

COOH, Fmoc-His(Boc)-COOH, Boc-Cys(Trt)-COOH, Boc-Ser(tBu)-COOH, Boc-Thr(tBu)-COOH, Boc-Leu-COOH· and Boc-Thz-COOH. The resin was washed with DMF (5 mL × 3), CH_2Cl_2 (5 mL × 3), and DMF (5 mL × 3). For the coupling step, a solution of Fmoc protected amino acid or Boc protected amino acid (4.0 equiv. according to the resin capacity), HATU (4.0 equiv.) and DIEA (10 equiv.) in DMF was gently agitated with the resin at room temperature for 1h. The resin was washed with DMF (5 mL × 3), CH_2Cl_2 (5 mL × 3), and DMF (5 mL × 3).

For Fmoc or Boc protected AO(Me) or AO(Bn) modified amino acids, 1.5 equiv. was added along with 1.5 equiv. HATU, 1.5 equiv. HOAt, and 3.0 equiv. DIEA in DMF at 0.1 M to the resin at room temperature and was allowed to react for overnight. The resin was subsequently washed with DMF (5 mL × 3), CH_2Cl_2 (5 mL × 3), and DMF (5 mL × 3).

1.3. Cleavage of fully protected peptide from 2-chloro-trityl chloride resin

The on-resin fully protected peptide, obtained as described in the Fmoc-SPPS section, was subjected to mild acidic cleavage cocktail (5-10 mL) of CH_2Cl_2/AcOH/trifluoroethanol (8/1/1, v/v/v), 3 times for 60 min each. Following filtration, the resulting cleavage solutions were combined and concentrated to give crude protected peptide bearing the free carboxylic acid at the C-terminus.

1.4. General procedure for synthesis of model C-terminus peptide SAL esters

1.4.1. Synthesis of C-terminus '1' L-Amino acid salicylaldehyde semicarbazone ester hydrochloride (HCl·H2N-Xaa-CO-SALoff)

BocHN-Xaa(PG)-COOH, (1.0 equiv.) was dissolved in DMF, followed by the addition of HATU (1.0 equiv.) and DIEA (2.0 equiv.). The solution was stirred for 2 min, then salicylaldehyde semicarbazone (1.0 equiv.) was added. The reaction mixture was stirred for overnight, after that, it was diluted with EtOAc and washed with 1 N HCl and brine. The organic layer was removed by reduced pressure evaporation, and the residue was purified by silica gel chromatography (CH_2Cl_2/EtOAc, 2:1) to give the desired BocHN Xaa(PG)-CO-SAL^{off} product. This product was treated with a solution of HCl/dioxane (4 N) for 30 min, and the solvent was removed by co-evaporation under reduced pressure with toluene. Without purification, this salt was directly used in the n+1 reaction

1.4.2. Synthesis of C-terminal Peptide SAL esters using n+1 strategy[1]

The fully protected peptidyl acid (1.0 equiv.) was dissolved in $CHCl_3$/trifluoroethanol (10 mM, 3/1, v/v), and then the corresponding amino L-Amino salicylaldehyde semicarbazone ester hydrochloride (HCl·H_2N-Xaa-CO-SAL^{off}) (6.0 equiv.) and Hydroxy-3,4-dihydro-4-oxo-1,2,3-benzotriazine (HOOBt) (3.0 equiv.) were then added. Finally, N-(3-dimethylaminopropyl)-N'-ethylcarbodiimide (EDC) (3.0 equiv.) was added. The reaction mixture was stirred for 3 h to form the crude protected C-terminal peptide SAL^{off} ester. After that, the solvent was removed under reduced pressure and the resulting residue was treated with TFA/H_2O (95:5, v/v) containing pyruvic acid (100 equiv.) for 3 h. After that, TFA was blown off and the oily residue was triturated with diethyl ether and centrifuged. The precipitate was pelleted, and the ether was

subsequently decanted. The resulting solid was purified by HPLC and lyophilization to give the peptide SAL esters as white solid.

1.5. Preparation of hydrazine 2-chlorotrityl chloride resin[2]

2CTC resin (1 g, loading = ~0.5 mmol/g) was swelled in 10 mL CH_2Cl_2/DMF (1/1, v/v). Then 10 mL $NH_2NH_2 \cdot H_2O$/DMF (1/20, v/v) was added. The reaction was conducted for 30 min. 10 mL of methanol/DMF (1/20, v/v) was added to quench the remaining 2-chlorotrityl chloride resin. After 30 min, the resin was washed with DMF and CH_2Cl_2 and ready for iterative peptide assembly (Fmoc-SPPS).

1.6. General procedure for aminooxy ligation (AOL)

1.6.1. Aminooxy Ligation in aqueous buffer

Peptide SAL ester (1.0 equiv.) and N-terminal Aminooxy peptide (1.1-1.2 equiv.) were incubated in 6 M Guanidine-HCl buffered with 0.5 M sodium citrate at pH 4 for AO(Me) and pH 4.5 for AO(Bn) at a concentration of 20 mM at room temperature. After the completion of the reaction, the reaction mixture is diluted with water or 6 M Guanidine-HCl for purification by HPLC and lyophilization to give the peptide as white solid.

1.6.2. Aminooxy Ligation in Pyridine/acetic acid

Peptide SAL ester (1.0 equiv.) and N-terminal Aminooxy peptide (1.1-1.2 equiv.)

were incubated in pyridine/acetic acid (1/3, v/v) at a concentration of 10 mM at room temperature. After the completion of the reaction, the solvent was blown off under a stream of condensed air. The residue was then treated with TFA/H_2O (97.5/2.5, v/v) at a concentration of 2 mM at room temperature for 0.5 h. After that, TFA was blown off and the residue was triturated with diethyl ether and centrifuged. The precipitate was pelleted and the ether was subsequently decanted, purified by HPLC and lyophilization to give the peptide as white solid.

1.7. General Procedure for CEL

Aminooxy peptide (1.0 equiv.) and target ketoacid (3.0-10.0 equiv.) were incubated in aqueous DMSO with 5-10% 0.01 M oxalic acid at a concentration of 20-40 mM at 60°C for 3 h. After the completion of the reaction, the reaction mixture is diluted with water or 6 M Guanidine-HCl for purification by HPLC and lyophilization to give the peptide as white solid.

1.8. General Procedure for aminooxy glycosylation

AO(Me) peptide (1.0 equiv.), zinc chloride (250.0 eq), and reducing sugar (500.0 eq) were incubated in 6 M Guanidine-HCl buffered with 0.1 M sodium citrate at pH 4 at a concentration of 1 mM relative to the AO(Me) peptide at room temperature for overnight. After the completion of the reaction, the reaction mixture is diluted with water or 6 M Guanidine-HCl for purification by HPLC and lyophilization to give the peptide as white solid.

1.9. General Procedure for Zinc cleavage of N-O bond to restore native serine

Modified from reported N-O cleavage method.[4] To a solution of aminooxy peptide (1.0 eq.) in 1.0 M ascorbic acid (1 mM), Zinc powder (100 eq.) was added, and the reaction mixture was incubated at 37 °C for few hours to overnight. The reaction was confirmed by UPLC-MS analysis.

1.10. General Procedure for Native Chemical Ligation

N-terminal peptidyl hydrazine (2.0 equiv.) was dissolved in aqueous buffer containing 6 M Guanidine-HCl and 0.2 M NaH_2PO_4 (pH = 3.0) (peptide final concentration is 20 mg/mL). After that, mercaptophenylacetic acid (MPAA, 10.0 equiv) and acetylacetone (AcAc, 5.0 equiv)[3] were added into the above mixture. The pH was adjusted to 2 and the reaction mixture was stirred under room temperature for 3 h, then C-terminal peptide (1.0 equiv) was added into the reaction mixture, and the pH of reaction mixture was then adjusted to 6.8 slowly with aqueous NaOH solution (1M) to initiate the Native Chemical Ligation at room temperature (C-terminal peptide final concentration is 3 mM). After the completion of the reaction, the reaction mixture is diluted with 6 M Guanidine-HCl for purification by HPLC and lyophilization to give the peptide as white solid

2. Building block synthesis of AOSer and AOThr

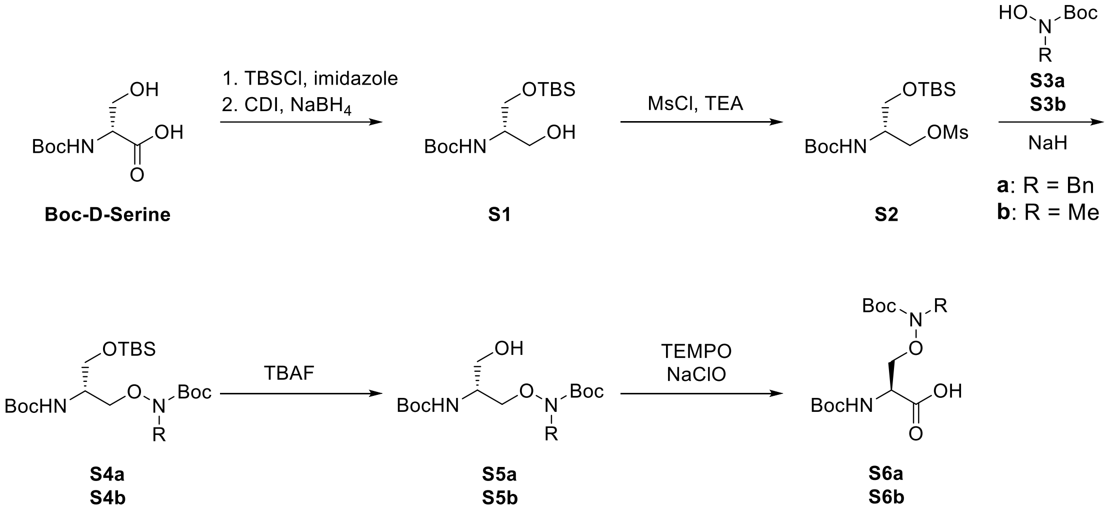

Scheme S1. Syntheses of AOSer and AOThr building blocks

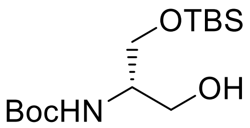

S1

To a solution of Boc-D-Serine (10.26 g, 50.0 mmol, 1.0 equiv.) dissolved in 80 mL of dry DMF, imidazole (9.00 g, 150.0 mmol, 3.0 equiv.) and tert-Butyldimethylsilyl chloride (7.93 g, 75.0 mmol, 1.5 equiv.) were added to the reaction mixture subsequently at room temperature. The above reaction mixture was stirred at room temperature for 14 h. After that, the reaction mixture was diluted with 400 mL of EA and washed with 1 N HCl (400.0 mL) x 2, and brine subsequently. The organic layer was dried with sodium sulfate and removed by reduced pressure evaporation. The reaction crude was then dissolved in 150 mL of THF, and 1,1-carbonyldiimidazole (10.81 g, 66.67 mmol, 1.33 equiv.) was added at room temperature and stirred at the same temperature for 10 minutes. The reaction mixture was then cooled to 0°C and $NaBH_4$ (3.15 g, 83.33 mmol, 1.67 equiv.) dissolved in 83 mL of H_2O (1 M) was

poured slowly into the reaction mixture and stirred at 0°C for 45 minutes. After that, the reaction mixture was diluted with 400 mL of EA and washed with 1 N HCl (100.0 mL) x 2, and brine subsequently. The organic layer was dried with sodium sulfate and removed by reduced pressure evaporation. The residue was purified by silica gel chromatography (Hexane/EtOAc, 10:1) to give the desired product **S1** (10.53 g, 68.9 %) as a colorless oil.

^{1}H NMR (500 MHz, $CDCl_3$) δ = 5.13 (s, 1H), 3.80 – 3.75 (m, 3H), 3.66 – 3.63 (m, 2H), 2.72 (br, 1H), 1.43 (s, 9H), 0.88 (s, 9H), 0.05 (s, 6H).
^{13}C NMR (126 MHz, $CDCl_3$) δ 156.05, 79.55, 77.29, 77.03, 76.78, 63.88, 52.67, 28.37, 25.82, 18.19, -5.56.
HRMS (ESI+) for $C_{14}H_{31}NO_4Si$ $[M+H]^+$ calcd 306.2095; found 306.2113.

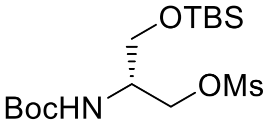

S2

To a solution of **S1** (6.10 g, 20.0 mmol, 1.0 equiv.) and triethylamine (4.2 mL, 30.0 mmol, 1.5 equiv.) dissolved in 50 mL of dry DCM, Methanesulfonyl chloride (1.9 mL, 24.0 mmol, 1.2 equiv.) was added dropwise to the reaction mixture at room temperature. The reaction mixture was stirred at room temperature for 15 minutes, then the DCM was removed by reduced pressure evaporation. The crude was then diluted with 250 mL of EA, washed with 1 N HCl (100.0 mL) x 3, and brine subsequently. The organic layer was dried with sodium sulfate and removed by reduced pressure evaporation. The reaction crude was used immediately in next step without purification to avoid degradation upon storage.

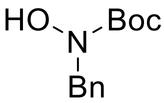

S3a

To a solution of N-Benzylhydroxylamine Hydrochloride (4.79 g, 30.0 mmol, 2.0 equiv.) dissolved in 12 mL of 1:1 THF/H_2O, K_2CO_3 (2.07 g, 15.0 mmol, 1.0 equiv.) and Boc_2O (7.6 mL, 33.0 mmol, 2.2 equiv.) in 9 mL of THF were added to the reaction mixture at 0°C. The above reaction mixture was stirred at 0°C for 2 h and then room temperature for 3 h. After that, the reaction mixture was diluted with 100 mL of EA and washed with 1 N HCl (100.0 mL) x 2, and brine subsequently. The organic layer was dried with sodium sulfate and removed by reduced pressure evaporation. The residue was purified by silica gel chromatography (Hexane/EtOAc, 15:1) to give the desired product **S3a** (5.09 g, 76.1%) as a pale yellow oil.

^{1}H NMR (500 MHz, $CDCl_3$) δ = 8.38 (br, 1H), 7.30 – 7.23 (m, 5H), 4.61 (s, 2H), 1.40 (s, 9H).

^{13}C NMR (126 MHz, $CDCl_3$) δ = 156.82, 136.59, 128.43, 128.07, 127.53, 82.12, 54.22, 28.30, 28.24.

HRMS (ESI+) for $C_{12}H_{17}NO_3$ $[M+Na]^+$ calcd 246.1101; found 246.1121.

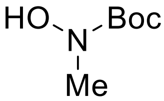

S3b

1b was synthesized using the same procedure for compound **1a** with N-methylhydroxylamine Hydrochloride (5.0 g, 60.0 mmol, 2.0 equiv.) as the starting material. The residue was purified by silica gel chromatography (Hexane/EtOAc, 15:1) to give the desired product **S3b** (4.31 g, 48.8%) as a pale yellow oil.

^{1}H NMR (600 MHz, $CDCl_3$) δ = 3.14 (s, 3H), 1.46 (s, 9H).

^{13}C NMR (151 MHz, $CDCl_3$) δ = 157.69, 81.77, 37.92, 28.28.

HRMS (ESI+) for $C_6H_{13}NO_3$ $[M+Na]^+$ calcd 170.0788; found 170.0809.

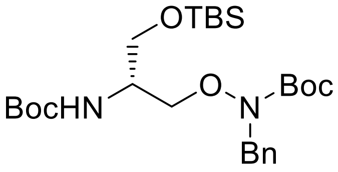

S4a

To a solution of **S3a** (5.02 g, 22.5 mmol, 1.5 equiv.) dissolved in 80 mL of anhydrous THF, NaH (0.90 g, 22.5 mmol, 1.5 equiv.) was added portion wise to the reaction mixture under Ar at 0°C. The reaction mixture was stirred at the same temperature for 15 minutes. Then **S2** (5.75 g, 15.0 mmol, 1.0 equiv.) dissolved in 40 mL of anhydrous THF was added to the reaction mixture. The reaction was stirred at room temperature for 16 h and was quenched by the addition of 22.5 mL of H_2O. After that, the reaction mixture was diluted with 400 mL of EA and washed with 1 N HCl (100.0 mL) x 2, and brine subsequently. The organic layer was dried with sodium sulfate and removed by reduced pressure evaporation. The residue was purified by silica gel chromatography (Hexane/EtOAc, 20:1) to give the desired product **S4a** (3.26 g, 42.6 %) as a pale yellow oil.

^1H NMR (500 MHz, $CDCl_3$) δ = 7.33 – 7.26 (m, 5H), 5.10 (br, 1H), 4.60 (s, 2H), 3.93 – 3.91 (m, 1H), 3.81 – 3.78 (dd, 1H), 3.73 (s, 1H), 3.64 – 3.61 (dd, 1H), 3.55 – 3.52 (dd, 1H), 1.49 (s, 9H), 1.43 (s, 9H), 0.86 (s, 9H), 0.02 (s, 6H).

^{13}C NMR (126 MHz, $CDCl_3$) δ = 156.38, 155.37, 136.73, 128.52, 128.48, 128.36, 127.53, 81.86, 73.67, 61.63, 53.46, 50.89, 28.40, 28.31, 28.23, 25.82, 25.79, 18.15, -5.54.

HRMS (ESI+) for $C_{26}H_{46}N_2O_6Si$ $[M+H]^+$ calcd 511.3198; found 511.3195.

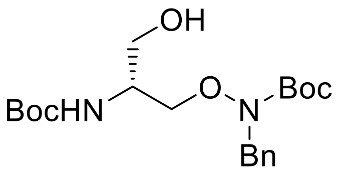

S5a

To a solution of **S4a** (1.82 g, 3.56 mmol, 1.0 equiv.) dissolved in 36 mL of THF, 1M Tetrabutylammonium fluoride in THF (4.27 mL, 4.27 mmol, 1.2 equiv.) was added to the reaction mixture at room temperature. The reaction was stirred at room temperature for 3 h. After that, the reaction mixture was diluted with 200 mL of EA and washed with 1 N HCl (100.0 mL) x 2, and brine subsequently. The organic layer was dried with sodium sulfate and removed by reduced pressure evaporation. The residue was purified by silica gel chromatography (Hexane/EtOAc, 5:1) to give the desired product **S5a** (1.32 g, 93.7 %) as a colorless oil.

^{1}H NMR (500 MHz, $CDCl_3$) δ = 7.36 – 7.28 (m, 5H), 5.21 (br, 1H), 4.59 (s, 2H), 3.92 –3.89 (dd, 1H), 3.82 – 3.77 (m, 2H), 3.72 (s, 1H), 3.58 – 3.56 (m, 2H), 1.48 (s, 9H), 1.43 (s, 9H).

^{13}C NMR (126 MHz, $CDCl_3$) δ = 156.52, 155.59, 136.40, 128.50, 128.43, 127.75, 82.54, 79.54, 74.31, 62.88, 53.83, 50.73, 28.34, 28.28, 28.24, 28.18.

HRMS (ESI+) for $C_{20}H_{32}N_2O_6$ $[M+H]^+$ calcd 397.2333; found 397.2350.

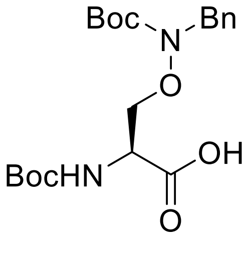

S6a

To a solution of **S5a** (1.32 g, 3.33 mmol, 1.0 equiv.) dissolved in 15 mL of ACN

and 15 mL of saturated KH_2PO_4 aqueous solution, sodium chlorite (602 mg, 6.66 mmol, 2.0 equiv.) and TEMPO (140 mg, 0.90 mmol, 0.27 equiv.) was added to the reaction mixture subsequently at room temperature. Then 13-16% aqueous sodium hypochlorite solution (1.7 mL, 0.5 mL/mmol) was added in one portion to the reaction mixture. The reaction was stirred at room temperature for 2 h. After that, the reaction mixture was diluted with 150 mL of EA and washed with 1 N HCl (100.0 mL), H_2O (30.0 mL), saturated NaS2O3 (10.0 mL), H_2O (30.0 mL), 1 N HCl (50.0 mL) and brine subsequently. The organic layer was dried with sodium sulfate and removed by reduced pressure evaporation to give the desired product **S6a** (1.29 g, 94.6 %) as a white solid.

^{1}H NMR (600 MHz, $CDCl_3$) δ = 7.33 – 7.28 (m, 5H), 5.73 – 5.71 (d, 2H), 4.65 – 4.51 (dd, 2H), 4.43 – 4.40 (dd, 2H), 4.17 – 4.14 (dd, 1H), 3.84 – 3.81 (dd, 1H), 1.47 (s, 9H), 1.44 (s, 9H)
^{13}C NMR (151 MHz, $CDCl_3$) δ = 172.98, 157.81, 155.46, 135.82, 128.55, 128.53, 128.48, 127.88, 83.74, 80.27, 73.86, 53.64, 52.16, 28.30, 28.17.
HRMS (ESI+) for $C_{20}H_{30}N_2O_7$ $[M+H]^+$ calcd 411.2126; found 411.2142.

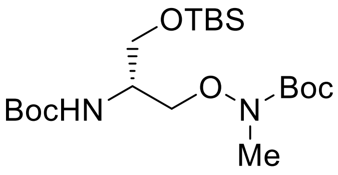

S4b

To a solution of **S3b** (4.42 g, 30.0 mmol, 1.5 equiv.) dissolved in 40 mL of anhydrous DMF, NaH (1.20 g, 30.0 mmol, 1.5 equiv.) was added portion wise to the reaction mixture under Ar at 0°C. The reaction mixture was stirred at the same temperature for 15 minutes. Then **S2** (7.67 g, 20.0 mmol, 1.0 equiv.) dissolved in 20 mL of anhydrous DMF was added to the reaction mixture. The reaction was stirred at room temperature for 16 h and was quenched by the

addition of 30 mL of H_2O. After that, the reaction mixture was diluted with 400 mL of EA and washed with 1 N HCl (100.0 mL) x 2, and brine subsequently. The organic layer was dried with sodium sulfate and removed by reduced pressure evaporation. The residue was purified by silica gel chromatography (Hexane/EtOAc, 30:1) to give the desired product **S4b** (3.97 g, 45.6 %) as a colorless oil.

^{1}H NMR (600 MHz, $CDCl_3$) δ = 5.17 (br, 1H), 4.03 – 4.00 (m, 1H), 3.86 – 3.84 (dd, 1H), 3.81 – 3.79 (m, 1H), 3.74 – 3.71 (dd, 1H), 3.63 – 3.60 (dd, 1H), 3.09 (s, 3H), 1.50 (s, 9H), 1.43 (s, 9H), 0.89 (s, 9H), 0.06 (s, 6H).
^{13}C NMR (151 MHz, $CDCl_3$) δ = 156.88, 155.49, 81.61, 72.69, 61.59, 50.78, 36.44, 28.39, 28.32, 25.85, 18.23, -5.40, -5.50.
HRMS (ESI+) for $C_{20}H_{42}N_2O_6Si$ $[M+H]^+$ calcd 435.2885; found 435.2896.

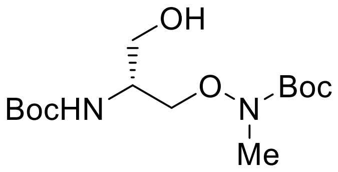

S5b

S5b was synthesized using the same procedure for compound **S5a** with **S4b** (3.96 g, 9.12 mmol, 1.0 equiv.) as the starting material. The residue was purified by silica gel chromatography (Hexane/EtOAc, 5:1) to give the desired product **S5b** (2.57 g, 87.8%) as a colorless oil.

^{1}H NMR (600 MHz, $CDCl_3$) δ = 5.29 (br, 1H), 4.03 – 4.00 (m, 1H), 3.94 – 3.91 (m, 1H), 3.87 – 3.84 (m, 1H), 3.78 (m, 1H), 3.66 – 3.63 (m, 1H), 3.16 (br, 1H), 3.10 – 3.09 (d, 3H), 1.48 – 1.47 (d, 9H), 1.43 (d, 9H).
^{13}C NMR (151 MHz, $CDCl_3$) δ = 156.96, 155.87, 82.30, 82.29, 79.71, 73.57, 62.92, 53.42, 50.75, 36.63, 28.35, 28.32, 28.24, 28.22, 28.10.

HRMS (ESI+) for $C_{14}H_{28}N_2O_6$ $[M+H]^+$ calcd 321.2020; found 321.2039.

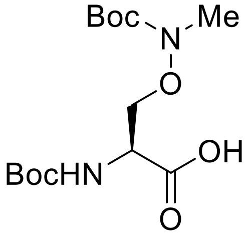

S6b

S6b was synthesized using the same procedure for compound **S6a** with **S5b** (2.56 g, 8.00 mmol, 1.0 equiv.) as the starting material to give the desired product **S6b** (2.30 g, 85.9%) without purification as a white solid after lyophilization.

^1H NMR (600 MHz, $CDCl_3$) δ = 5.69 – 5.68 (d, 2H), 4.45 – 4.41 (m, 1H), 4.19 – 4.16 (dd, 1H), 3.82 – 3.78 (dd, 1H), 3.13 (s, 3H), 1.51 (s, 9H), 1.44 (s, 9H).
^{13}C NMR (151 MHz, $CDCl_3$) δ = 171.76, 159.16, 155.18, 84.32, 80.33, 73.01, 51.60, 36.76, 28.30, 28.16.
HRMS (ESI+) for $C_{14}H_{26}N_2O_7$ $[M+H]^+$ calcd 335.3765; found 335.1830.

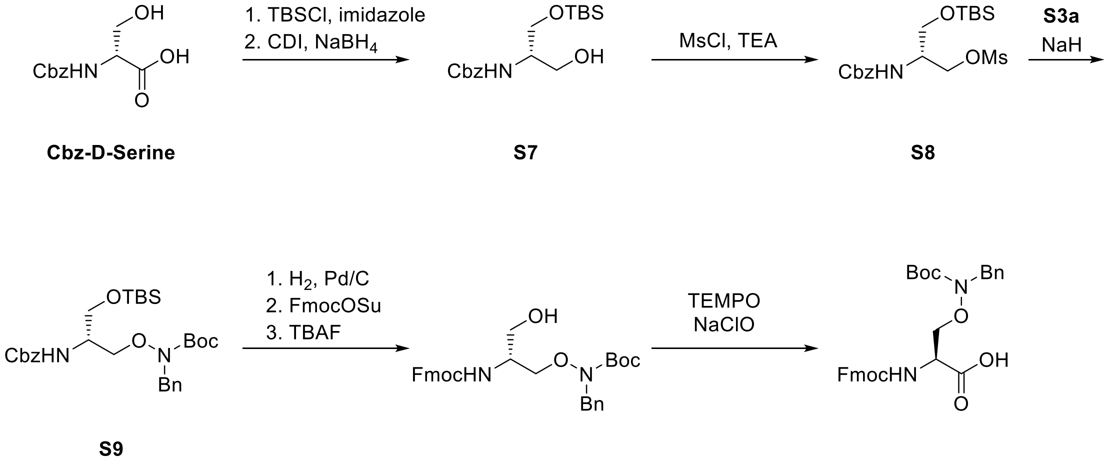

Scheme S2. Syntheses of Fmoc-protected AO building blocks

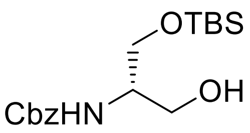

S7

To a solution of Cbz-D-Serine (3.59 g, 15.0 mmol, 1.0 equiv.) dissolved in 30 mL of dry DMF, imidazole (3.06 g, 45.0 mmol, 3.0 equiv.) and tert-Butyldimethylsilyl chloride (2.38 g, 22.5 mmol, 1.5 equiv.) were added to the reaction mixture subsequently at room temperature. The above reaction mixture was stirred at room temperature for 14 h. After that, the reaction mixture was diluted with 250 mL of EA and washed with 1 N HCl (150.0 mL) x 2, saturated $NaHCO_3$ (100.0 mL) x 2, 1 N HCl (150.0 mL) x 2 and brine subsequently. The organic layer was dried with sodium sulfate and removed by reduced pressure evaporation. The reaction crude was then dissolved in 45 mL of THF, and 1,1-carbonyldiimidazole (3.24 g, 20.0 mmol, 1.33 equiv.) was added at room temperature and stirred at the same temperature for 10 minutes. The reaction mixture was then cooled to 0°C and $NaBH_4$ (946 mg, 25.0 mmol, 1.67 equiv.) dissolved in 25 mL of H_2O (1 M) was poured slowly into the reaction mixture and stirred at 0°C for 45 minutes. After that, the reaction mixture was diluted with 250 mL of EA and washed with 1 N HCl (150.0 mL) x 2, and brine subsequently. The organic layer was dried with sodium sulfate and removed by reduced pressure evaporation. The residue was purified by silica gel chromatography (Hexane/EtOAc, 5:1) to give the desired product **7** (3.68 g, 72.1 %) as a colorless oil.

^{1}H NMR (600 MHz, $CDCl_3$) δ = 7.36 – 7.31 (m, 5H), 5.41 – 5.39 (d, 1H), 5.11 (s, 2H), 3.86 – 3.77 (m, 3H), 3.75 – 3.72 (m, 1H), 3.71 – 3.68 (dd, 1H), 2.41 (br, 1H), 0.88 (s, 9H), 0.06 – 0.05 (d, 6H).

^{13}C NMR (151 MHz, $CDCl_3$) δ = 156.46, 136.41, 128.55, 128.18, 128.14, 66.87,

63.91, 63.73, 53.03, 25.83, 18.20, -5.56, -5.58.

HRMS (ESI+) for $C_{17}H_{29}NO_4Si$ $[M+H]^+$ calcd 340.1939; found 340.1957.

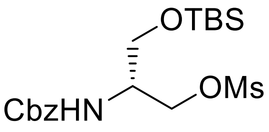

S8

8 was synthesized using the same procedure for compound **3** with **7** (3.67 g, 10.82 mmol, 1.0 equiv.) as the starting material. The residue was directly used in the next step immediately without further purification to avoid degradation.

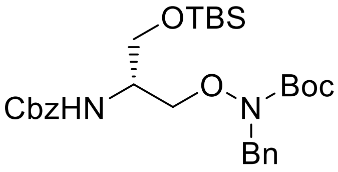

S9

9 was synthesized using the same procedure for compound **4a** with **8** (4.52 g, 10.82 mmol, 1.0 equiv.) and **1** (3.62 g, 16.23 mmol, 1.5 equiv.) as the starting material. The residue was purified by silica gel chromatography (Hexane/EtOAc, 15:1) to give the desired product **7** (2.22 g, 37.7 %) as a colorless oil.

^{1}H NMR (600 MHz, $CDCl_3$) δ = 7.35 – 7.30 (m, 10H), 5.12 – 5.07 (dd, 2H), 4.62, 4.56 (dd, 2H), 3.95 – 3.93 (m, 1H), 3.82 – 3.77 (m, 2H), 3.68 – 3.65 (dd, 1H), 3.57 – 3.54 (dd, 1H), 1.47 (s, 9H), 0.85 (s, 9H), 0.01 (s, 6H).

^{13}C NMR (151 MHz, $CDCl_3$) δ = 156.49, 156.06, 136.70, 136.65, 128.52, 128.46, 128.44, 128.02, 127.61, 82.03, 73.68, 66.56, 61.56, 53.73, 51.46, 28.28, 25.84, 18.19, -5.46, -5.53.

HRMS (ESI+) for $C_{29}H_{44}N_2O_6Si$ $[M+H]^+$ calcd 545.3401; found 545.3041.

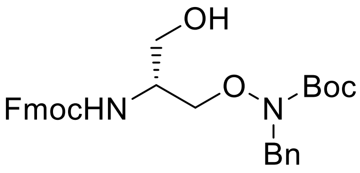

S10

To a solution of **9** (1.95 g, 3.59 mmol, 1.0 equiv.) dissolved in 50 mL of EA, 10% Pd/C (489 mg, 25% wt) were added at room temperature. The above reaction mixture was stirred at room temperature. After stirred under hydrogen (1 atm) for 3 h at room temperature, the resulting mixture was filtered and the organic solvent was removed by reduced pressure evaporation. The crude was then dissolved in 40 mL THF and 20 mL saturated $NaHCO_3$, Fmoc-OSu (1.82 g, 5.39 mmol, 1.5 equiv.) was added in one portion at room temperature. Ther reaction mixture was stirred at room temperature for 30 minutes. After that, the reaction mixture was diluted with 250 mL of EA and washed with 1 N HCl (150.0 mL) x 2, and brine subsequently. The organic layer was dried with sodium sulfate and removed by reduced pressure evaporation. The residue was redissolved in 35 mL THF, to which a mixture of 1M Tetrabutylammonium fluoride in THF (3.95 mL, 3.95 mmol, 1.1 equiv.) and acetic acid (0.41 mL, 7.18 mmol, 2.0 equiv.) was added at room temperature. The reaction was stirred at room temperature for 16 h. After that, the reaction mixture was diluted with 200 mL of EA and washed with 1 N HCl (100.0 mL) x 2, and brine subsequently. The organic layer was dried with sodium sulfate and removed by reduced pressure evaporation. The residue was purified by silica gel chromatography (Hexane/EtOAc, 2:1) to give the desired product **10** (1.27 g, 68.5 %) as a white solid.

^1H NMR (600 MHz, $CDCl_3$) δ = 7.77 – 7.76 (d, 2H), 7.61 – 7.60 (t, 2H), 7.41 – 7.39 (t, 2H), 7.34 – 7.28 (m, 7H), 5.68 – 5.66 (d, 1H), 4.59 (s, 2H), 4.40 – 4.34

(m, 2H), 4.22 – 4.19 (t, 1H), 3.95 – 3.92 (dd, 1H), 3.85 – 3.82 (m, 2H), 3.80, 3.80 – 3.77 (m, 1H), 3.63 – 3.60 (dd, 1H), 1.49 (s, 9H).

^{13}C NMR (151 MHz, $CDCl_3$) δ = 156.72, 156.44, 143.92, 143.90, 141.33, 136.34, 128.59, 128.45, 127.87, 127.71, 127.07, 127.06, 125.16, 125.13, 119.99, 82.81, 74.55, 66.86, 62.86, 54.14, 51.30, 47.22, 28.25.

HRMS (ESI+) for $C_{30}H_{34}N_2O_6$ $[M+H]^+$ calcd 519.2490; found 519.2491.

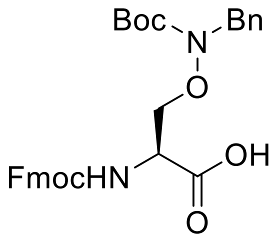

S11

11 was synthesized using the same procedure for compound **6a** with **10** (1.27 g, 2.45 mmol, 1.0 equiv.) as the starting material to give the desired product **11** (1.36 g, Quant. yield) without purification as a white solid.

^{1}H NMR (600 MHz, $CDCl_3$) δ = 7.77 – 7.76 (d, 2H), 7.62 – 7.60 (t, 2H), 7.42 – 7.39 (t, 2H), 7.35 – 7.28 (m, 7H), 6.20 (s, 1H), 4.69 – 4.54 (dd, 2H), 4.51 – 4.48 (m, 1H), 4.43 – 4.40 (dd, 1H), 4.37 – 4.34 (dd, 1H), 4.24 – 4.22 (t, 1H), 4.19 – 4.17 (dd, 1H), 3.86 – 3.83 (dd, 1H), 1.47 (s, 9H).

^{13}C NMR (151 MHz, $CDCl_3$) δ = 172.00, 158.43, 155.95, 143.82, 143.65, 141.33, 141.31, 135.60, 128.62, 128.38, 128.01, 127.77, 127.12, 125.19, 125.15, 120.03, 84.37, 73.76, 67.32, 53.86, 52.27, 47.08, 28.14.

HRMS (ESI+) for $C_{30}H_{32}N_2O_7$ $[M+H]^+$ calcd 533.2282; found 533.2285.

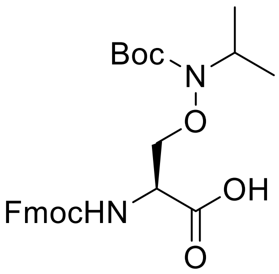

S12

12 was synthesized using the same procedure for compound **11** from compound **7**.

^{1}H NMR (600 MHz, $CDCl_3$) δ 7.77 – 7.76 (d, 2H), 7.63 – 7.60 (t, 2H), 7.41 – 7.39 (t, 2H), 7.32 – 7.30 (t, 2H), 4.55 – 4.51 (m, 1H), 4.42 – 4.33 (dt, 2H), 4.24 – 4.21 (dd, 2H), 4.18 – 4.13 (m, 1H), 3.97 – 3.94 (dd, 1H), 1.52 (s, 9H), 1.25 – 1.17 (dd, 6H).

^{13}C NMR (151 MHz, $CDCl_3$) δ 171.73, 155.98, 143.85, 143.64, 141.33, 141.30, 134.72, 129.11, 127.76, 127.10, 125.21, 125.15, 124.37, 120.33, 120.02, 67.30, 53.43, 52.41, 47.09, 28.26, 28.21, 19.30, 18.86.

HRMS (ESI+) for $C_{26}H_{32}N_2O_7$ $[M+H]^+$ calcd 485.2282; found 485.2291.

3. Discovery and development of AOL

3.1. Discovery of AOL

3.1.1. Preparation of peptidyl SAL ester

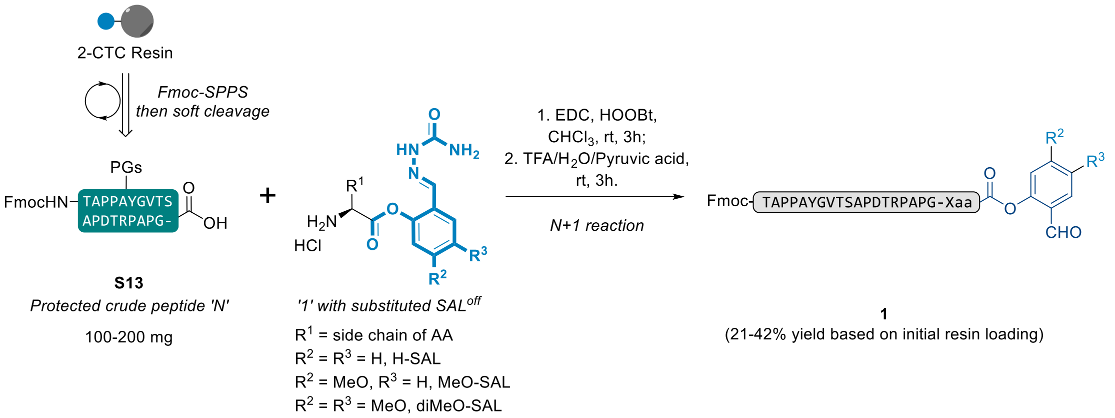

Figure S1. Synthetic route for model SAL ester **1**.

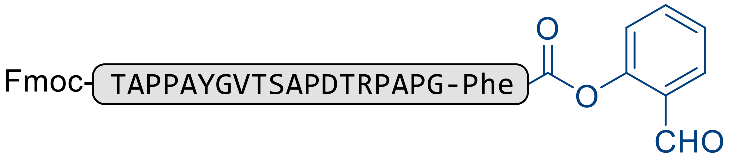

1 (Xaa = Phe, H-SAL)

SAL ester **1 (Xaa=Phe, H-SAL)** was synthesized according to the general SAL ester preparation method at 0.04~0.08 mmol scale. The crude peptide was purified by preparative reverse-phase HPLC (10-50% CH_3CN/H_2O over 30 min) and lyophilized to afford the desired SAL ester **1 (Xaa=Phe, H-SAL)** (36 mg, 32% yield).

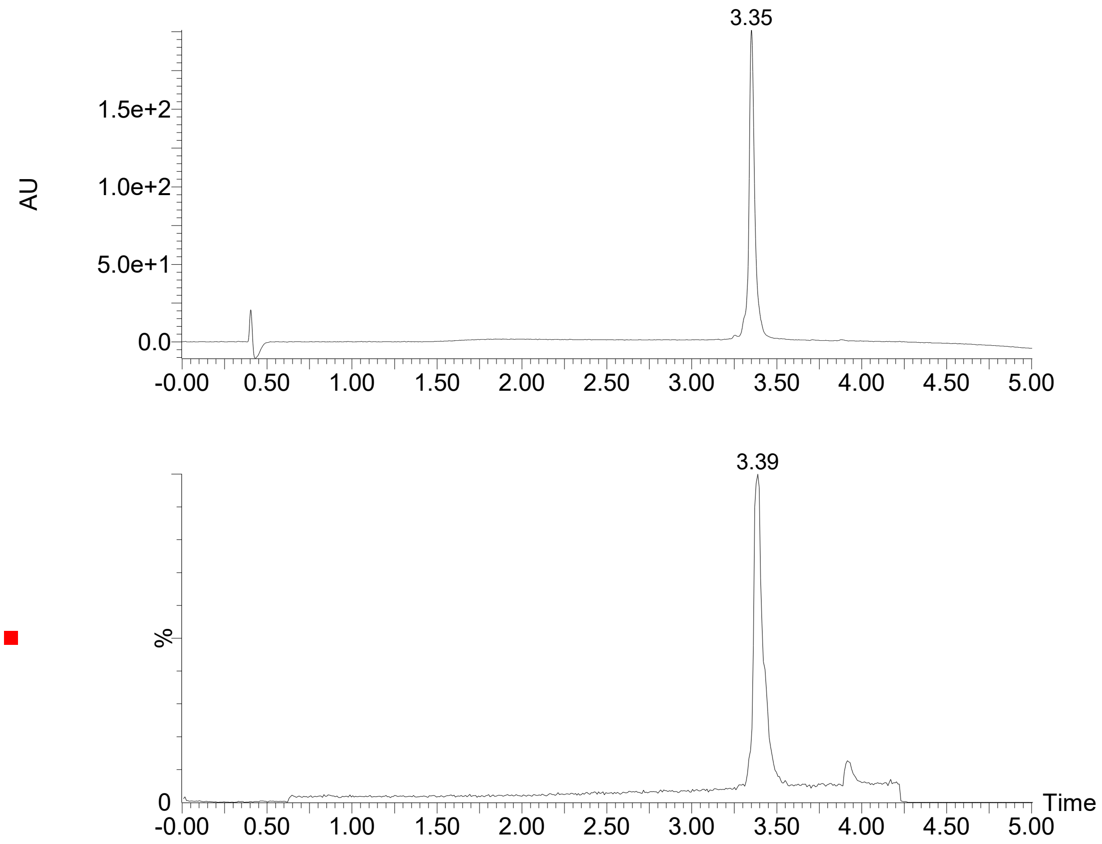

Figure S2. UV (190-400 nm) trace from UPLC-MS analysis of purified **1 (Xaa=Phe, H-SAL)** gradient 5-95% CH_3CN/H_2O containing 0.1% TFA over 5 min at a flow rate of 0.4 mL/min.

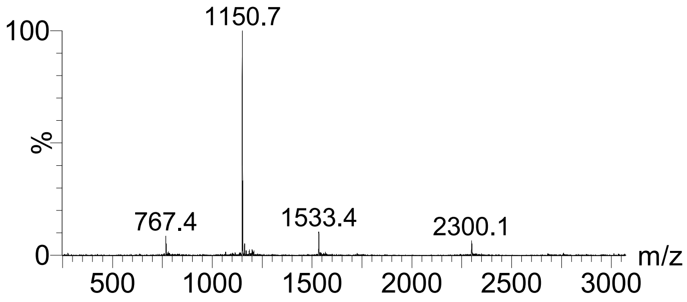

Figure S3. ESI-MS calcd. for C111H147N23O31 $[M+1H]^{1+}$ m/z = 2300.5, found 2300.1; $[M+2H]^{2+}$ m/z = 1150.8, found 1150.7; $[M+3H]^{3+}$ m/z = 767.5, found 767.4.

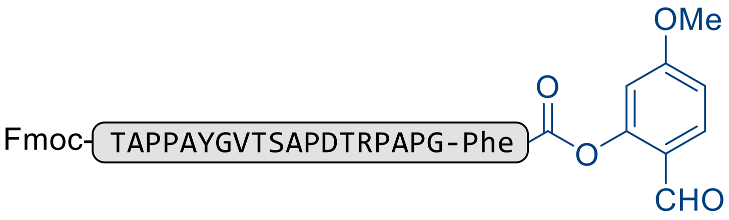

1 (Xaa = Phe, MeO-SAL)

SAL ester **1 (Xaa=Phe, MeO-SAL)** was synthesized according to the general SAL ester preparation method at 0.04~0.08 mmol scale. The crude peptide was purified by preparative reverse-phase HPLC (10-50% CH_3CN/H_2O over 30 min) and lyophilized to afford the desired SAL ester **1 (Xaa=Phe, MeO-SAL)** (33 mg, 29% yield).

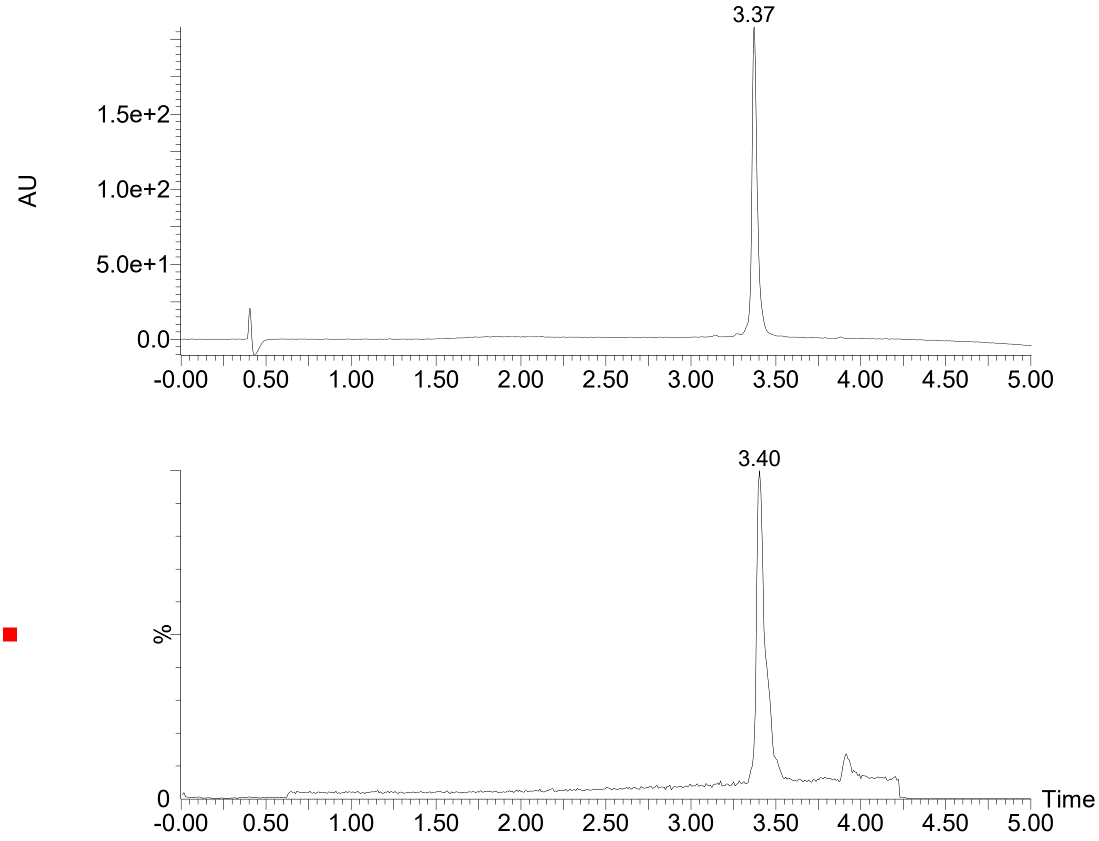

Figure S4. UV (190-400 nm) trace from UPLC-MS analysis of purified **1 (Xaa=Phe, MeO-SAL)** gradient 5-95% CH_3CN/H_2O containing 0.1% TFA over 5 min at a flow rate of 0.4 mL/min.

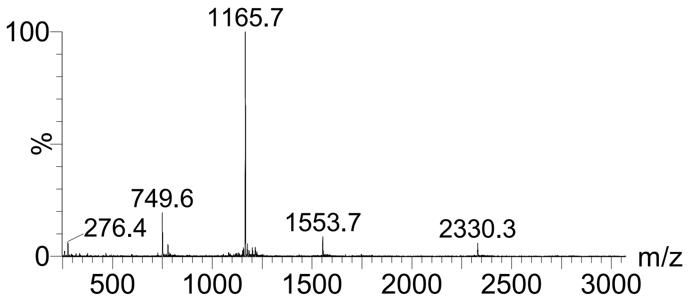

Figure S5. ESI-MS calcd. for C112H149N23O32 $[M+1H]^{1+}$ m/z = 2330.5, found 2330.3; $[M+2H]^{2+}$ m/z = 1165.8, found 1165.7.

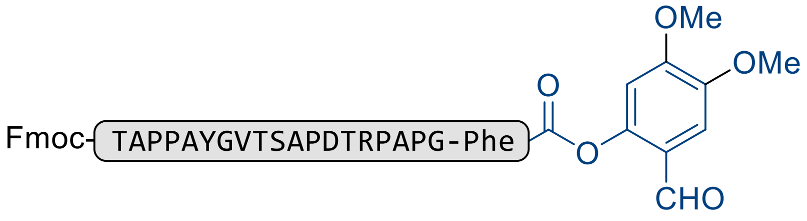

1 (Xaa = Phe, diMeO-SAL)

30 mg, 27%

SAL ester **1 (Xaa=Phe, diMeO-SAL)** was synthesized according to the general SAL ester preparation method at 0.04~0.08 mmol scale. The crude peptide was purified by preparative reverse-phase HPLC (10-50% CH_3CN/H_2O over 30 min) and lyophilized to afford the desired SAL ester **1 (Xaa=Phe, diMeO-SAL)** (30 mg, 27% yield).

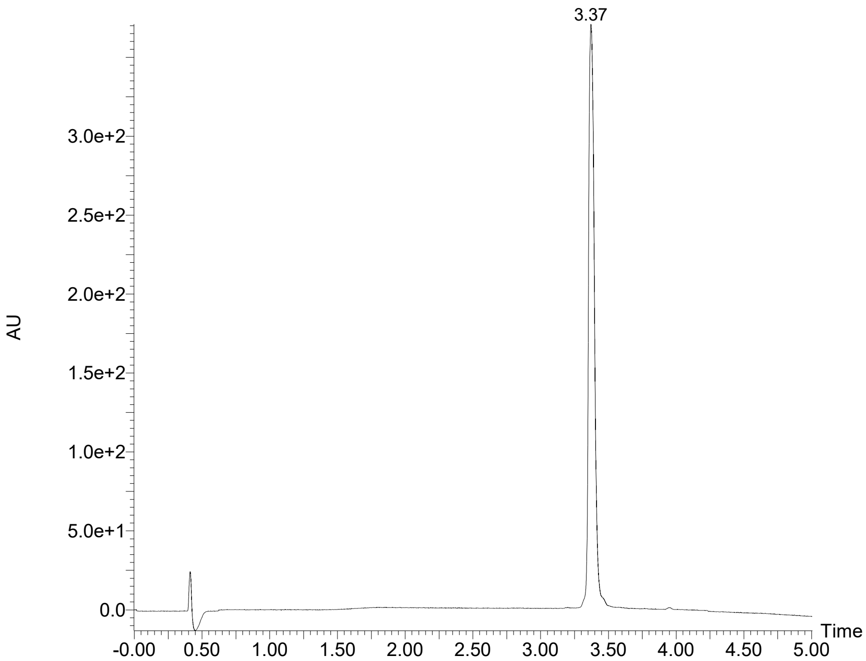

Figure S6. UV (190-400 nm) trace from UPLC-MS analysis of purified **1 (Xaa=Phe, diMeO-SAL)** gradient 5-95% CH_3CN/H_2O containing 0.1% TFA over 5 min at a flow rate of 0.4 mL/min.

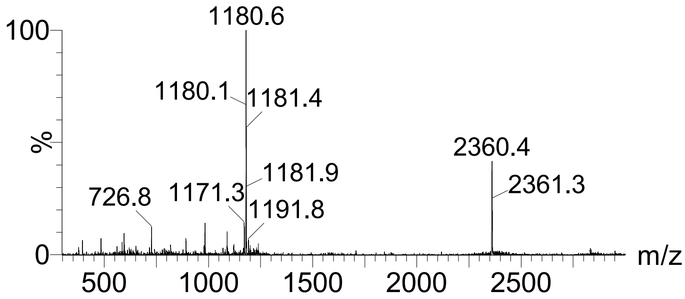

Figure S7. ESI-MS calcd. for C113H151N23O33 $[M+1H]^{1+}$ m/z = 2360.6, found 2360.4; $[M+2H]^{2+}$ m/z = 1180.8, found 1180.6.

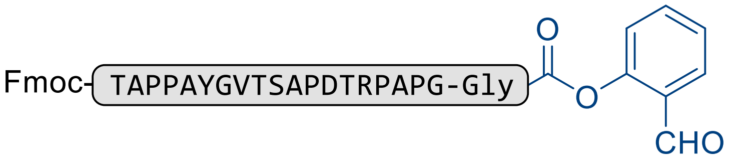

1 (Xaa = Gly, H-SAL)

SAL ester **1 (Xaa=Gly, H-SAL)** was synthesized according to the general SAL

ester preparation method at 0.04~0.08 mmol scale. The crude peptide was purified by preparative reverse-phase HPLC (10-50% CH_3CN/H_2O over 30 min) and lyophilized to afford the desired SAL ester **1 (Xaa=Gly, H-SAL)** (28 mg, 42% yield).

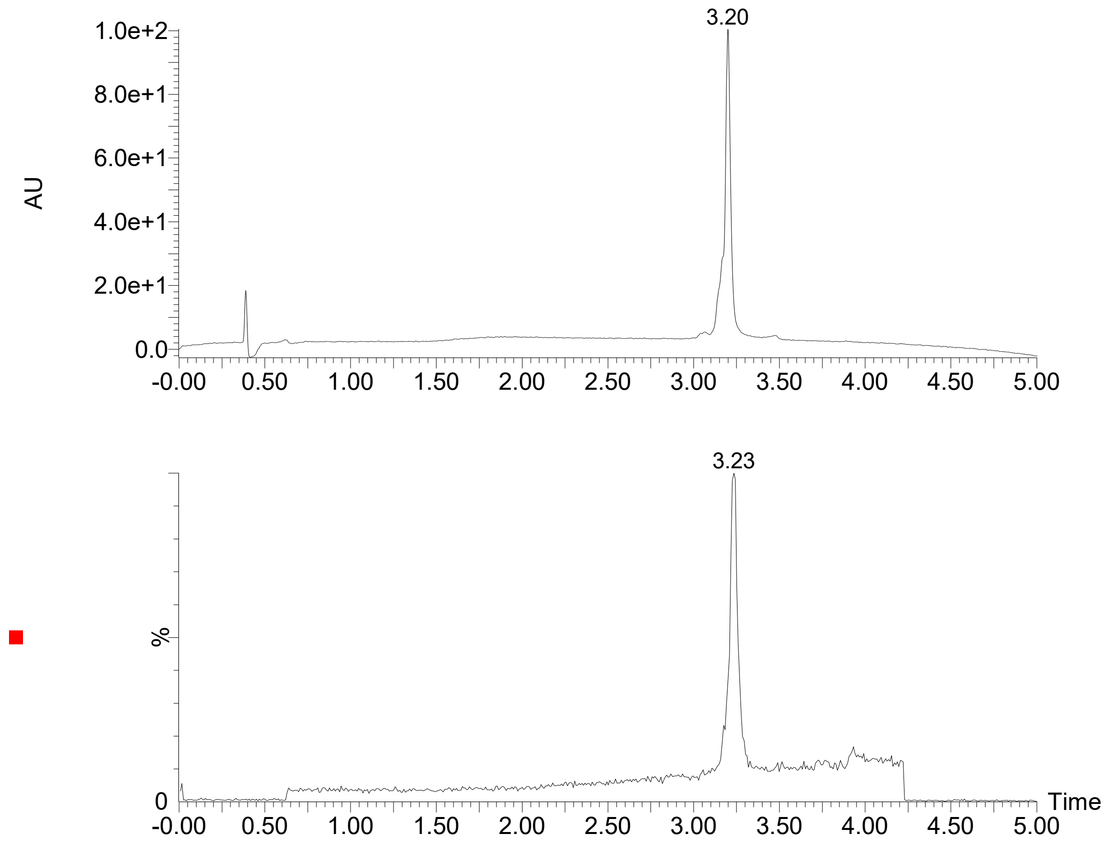

Figure S8. UV (190-400 nm) trace from UPLC-MS analysis of purified **1 (Xaa=Gly, H-SAL)** gradient 5-95% CH_3CN/H_2O containing 0.1% TFA over 5 min at a flow rate of 0.4 mL/min.

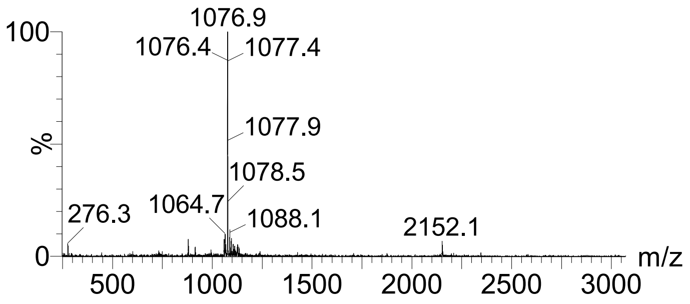

Figure S9. ESI-MS calcd. for C102H138N22O30 $[M+1H]^{1+}$ m/z = 2152.0, found 2152.1; $[M+2H]^{2+}$ m/z = 1076.5, found 1076.9.

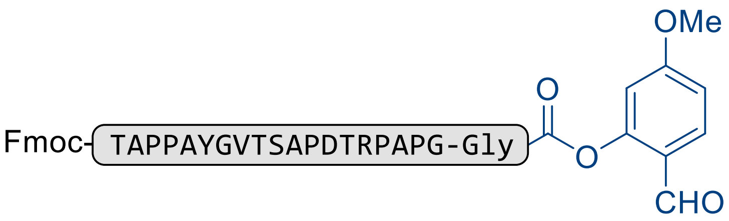

1 (Xaa = Gly, MeO-SAL)

26 mg, 39%

SAL ester **1 (Xaa=Gly, MeO-SAL)** was synthesized according to the general SAL ester preparation method at 0.04~0.08 mmol scale. The crude peptide was purified by preparative reverse-phase HPLC (10-50% CH_3CN/H_2O over 30 min) and lyophilized to afford the desired SAL ester **1 (Xaa=Gly, MeO-SAL)** (26 mg, 39% yield).

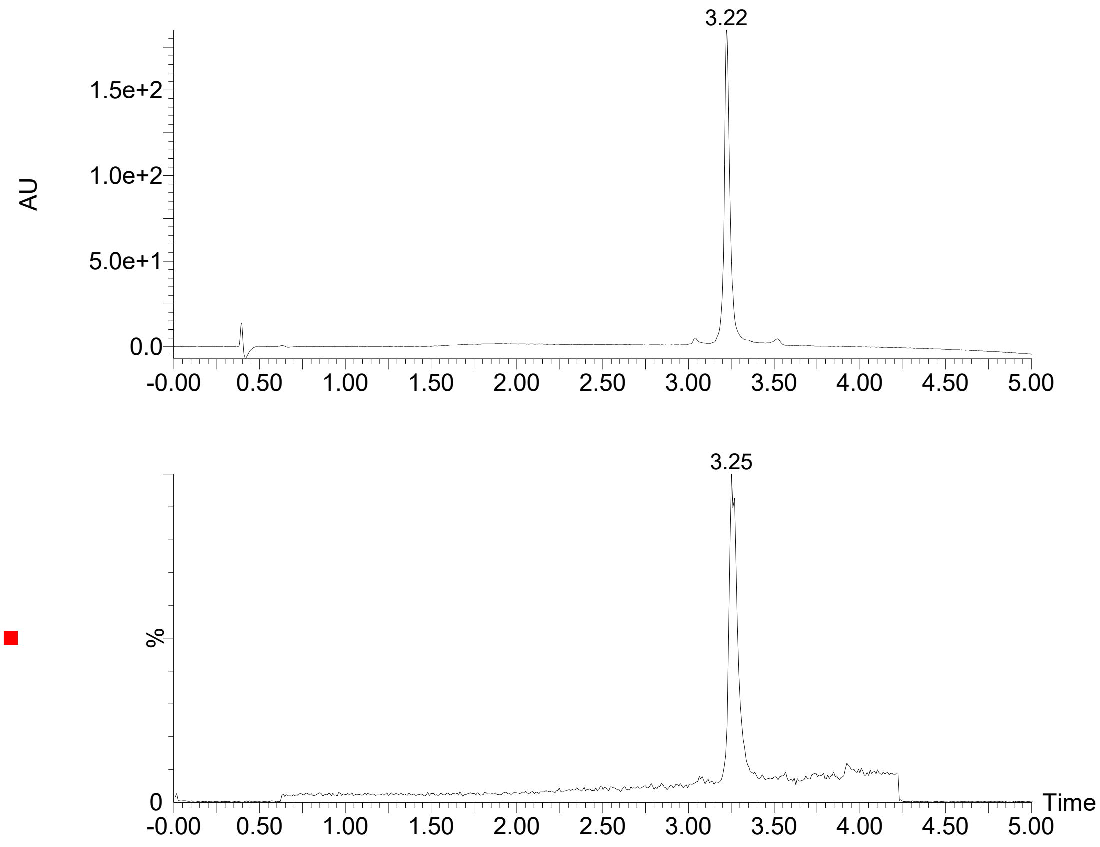

Figure S10. UV (190-400 nm) trace from UPLC-MS analysis of purified **1 (Xaa=Gly, MeO-SAL)** gradient 5-95% CH_3CN/H_2O containing 0.1% TFA over 5 min at a flow rate of 0.4 mL/min.

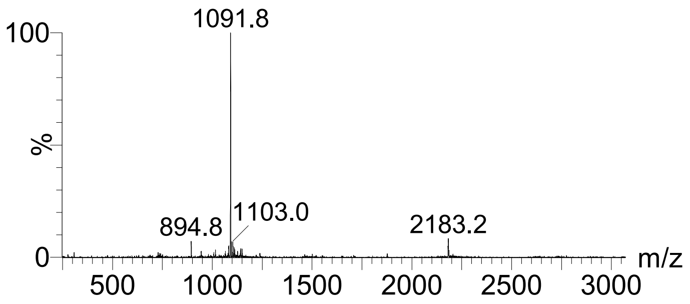

Figure S11. ESI-MS calcd. for C103H140N22O31 $[M+1H]^{1+}$ m/z = 2183.4, found 2183.2; $[M+2H]^{2+}$ m/z = 1092.2, found 1091.8.

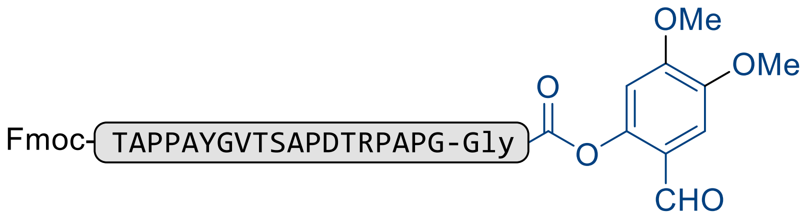

1 (Xaa = Gly, diMeO-SAL)

SAL ester **1 (Xaa=Gly, diMeO-SAL)** was synthesized according to the general SAL ester preparation method at 0.04~0.08 mmol scale. The crude peptide was purified by preparative reverse-phase HPLC (10-50% CH_3CN/H_2O over 30 min) and lyophilized to afford the desired SAL ester **1 (Xaa=Gly, diMeO-SAL)** (25 mg, 36% yield).

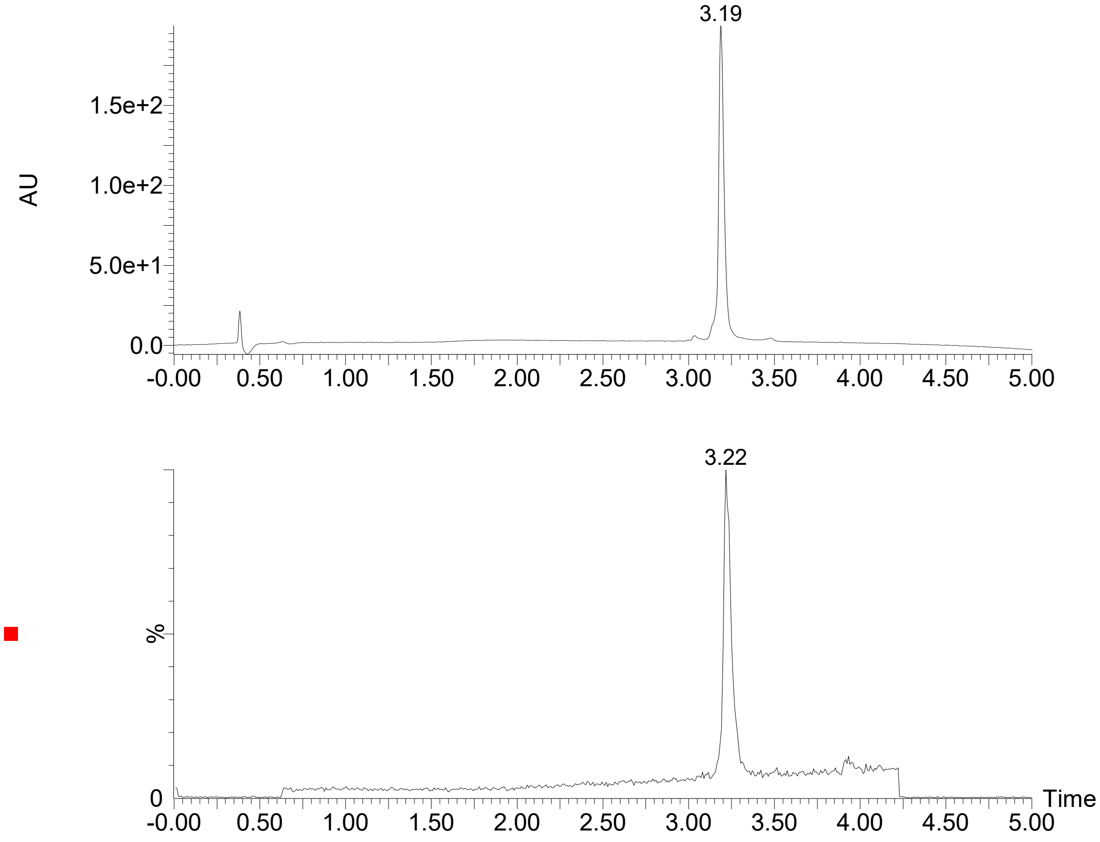

Figure S12. UV (190-400 nm) trace from UPLC-MS analysis of purified **1 (Xaa=Gly, diMeO-SAL)** gradient 5-95% CH_3CN/H_2O containing 0.1% TFA over 5 min at a flow rate of 0.4 mL/min.

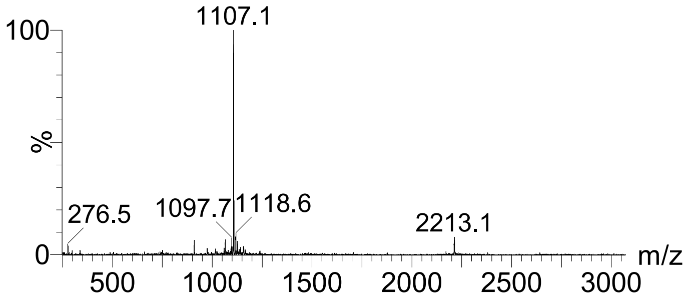

Figure S13. ESI-MS calcd. for C104H142N22O32 $[M+1H]^{1+}$ m/z = 2213.4, found 2213.1; $[M+2H]^{2+}$ m/z = 1107.2, found 1107.1.

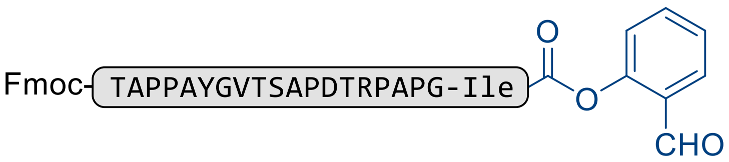

1 (Xaa = Ile, H-SAL)

SAL ester **1 (Xaa=Ile, H-SAL)** was synthesized according to the general SAL ester preparation method at 0.04~0.08 mmol scale. The crude peptide was purified by preparative reverse-phase HPLC (10-50% CH_3CN/H_2O over 30 min) and lyophilized to afford the desired SAL ester **1 (Xaa=Ile, H-SAL)** (39 mg, 35% yield).

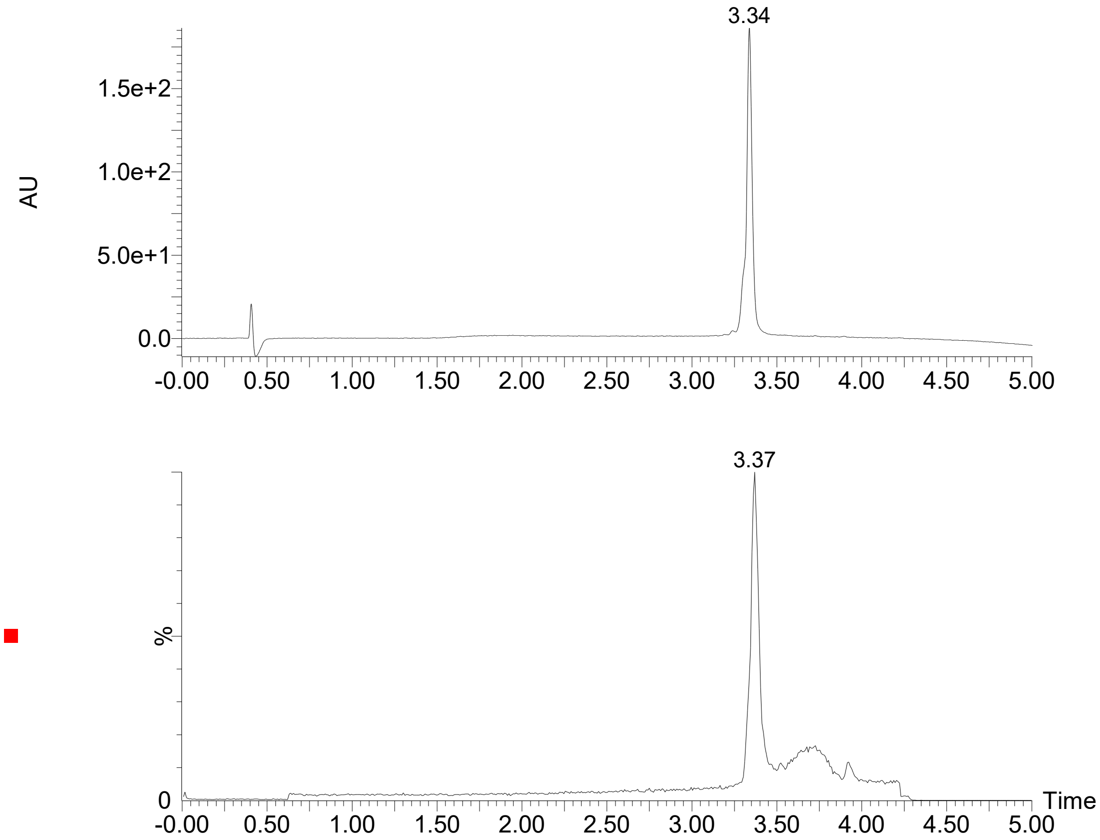

Figure S14. UV (190-400 nm) trace from UPLC-MS analysis of purified **1 (Xaa=Ile, H-SAL)** gradient 5-95% CH_3CN/H_2O containing 0.1% TFA over 5 min at a flow rate of 0.4 mL/min.

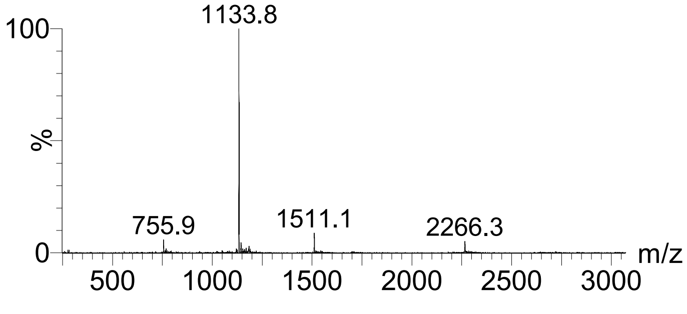

Figure S15. ESI-MS calcd. for C108H149N23O31 $[M+1H]^{1+}$ m/z = 2266.5, found 2266.3; $[M+2H]^{2+}$ m/z = 1133.7, found 1133.8; $[M+3H]^{3+}$ m/z = 756.2, found 755.9.

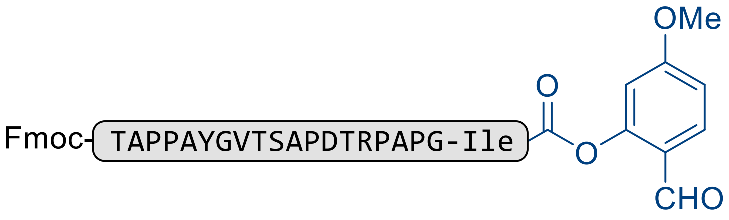

1 (Xaa = Ile, MeO-SAL)

SAL ester **1 (Xaa=Ile, MeO-SAL)** was synthesized according to the general SAL ester preparation method at 0.04~0.08 mmol scale. The crude peptide was purified by preparative reverse-phase HPLC (10-50% CH_3CN/H_2O over 30 min) and lyophilized to afford the desired SAL ester **1 (Xaa=Ile, MeO-SAL)** (34 mg, 30% yield).

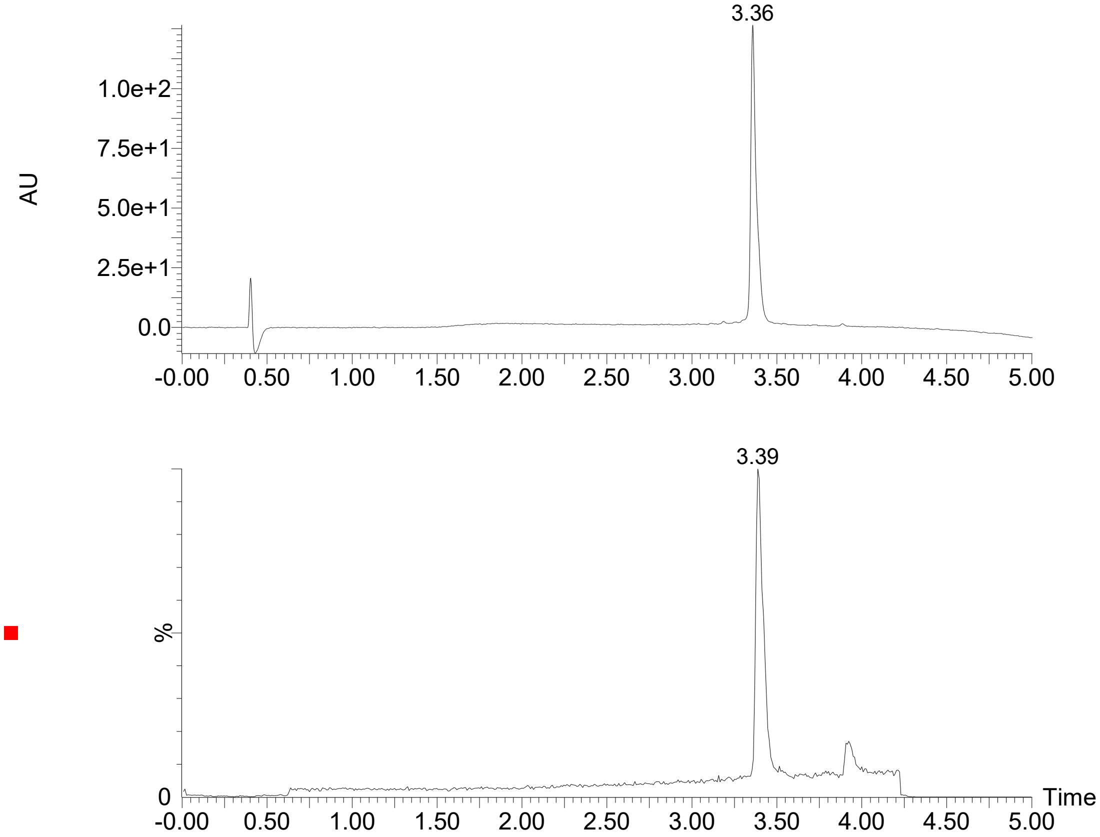

Figure S16. UV (190-400 nm) trace from UPLC-MS analysis of purified **1 (Xaa=Ile, MeO-SAL)** gradient 5-95% CH_3CN/H_2O containing 0.1% TFA

over 5 min at a flow rate of 0.4 mL/min.

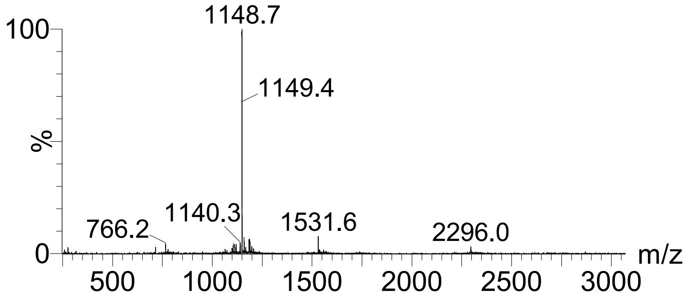

Figure S17. ESI-MS calcd. for C109H151N23O32 $[M+1H]^{1+}$ m/z = 2296.5, found 2296.0; $[M+2H]^{2+}$ m/z = 1148.8, found 1148.7; $[M+3H]^{3+}$ m/z = 766.2, found 766.2.

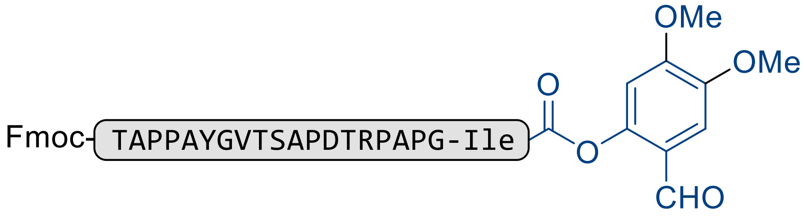

1 (Xaa = Ile, diMeO-SAL)

31 mg, 27%

SAL ester **1 (Xaa=Ile, diMeO-SAL)** was synthesized according to the general SAL ester preparation method at 0.04~0.08 mmol scale. The crude peptide was purified by preparative reverse-phase HPLC (10-50% CH_3CN/H_2O over 30 min) and lyophilized to afford the desired SAL ester **1 (Xaa=Ile, diMeO-SAL)** (31 mg, 27% yield).

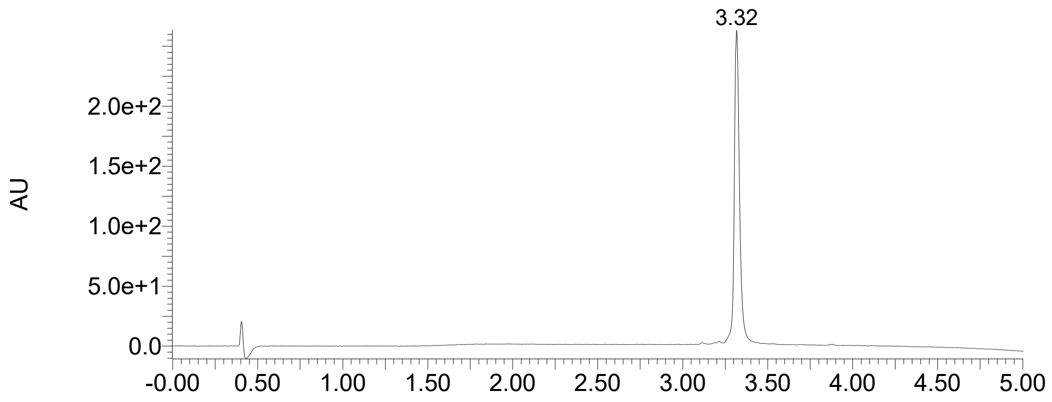

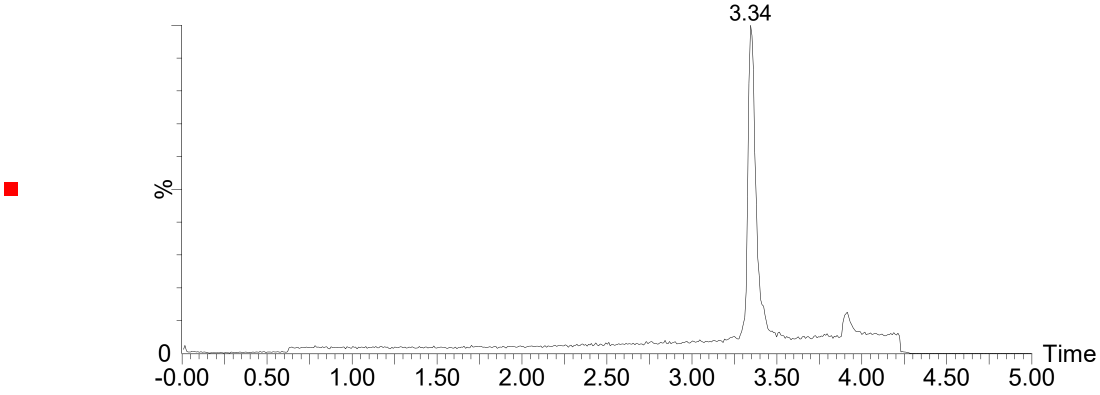

Figure S18. UV (190-400 nm) trace from UPLC-MS analysis of purified **1 (Xaa=Ile, diMeO-SAL)** gradient 5-95% CH_3CN/H_2O containing 0.1% TFA over 5 min at a flow rate of 0.4 mL/min.

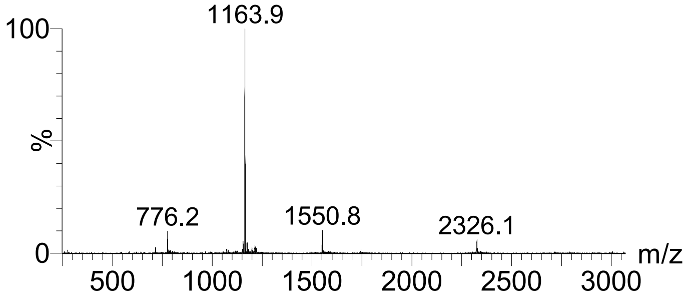

Figure S19. ESI-MS calcd. for $C_{110}H_{153}N_{23}O_{33}$ $[M+1H]^{1+}$ m/z = 2326.5, found 2326.1; $[M+2H]^{2+}$ m/z = 1163.8, found 1163.9; $[M+3H]^{3+}$ m/z = 776.2, found 776.2.

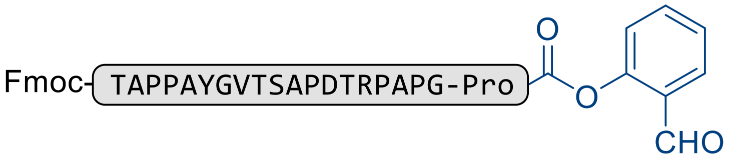

1 (Xaa = Pro, H-SAL)

SAL ester **1 (Xaa=Pro, H-SAL)** was synthesized according to the general SAL ester preparation method at 0.04~0.08 mmol scale. The crude peptide was purified by preparative reverse-phase HPLC (10-50% CH_3CN/H_2O over 30 min) and lyophilized to afford the desired SAL ester **1 (Xaa=Pro, H-SAL)** (21 mg, 32% yield).

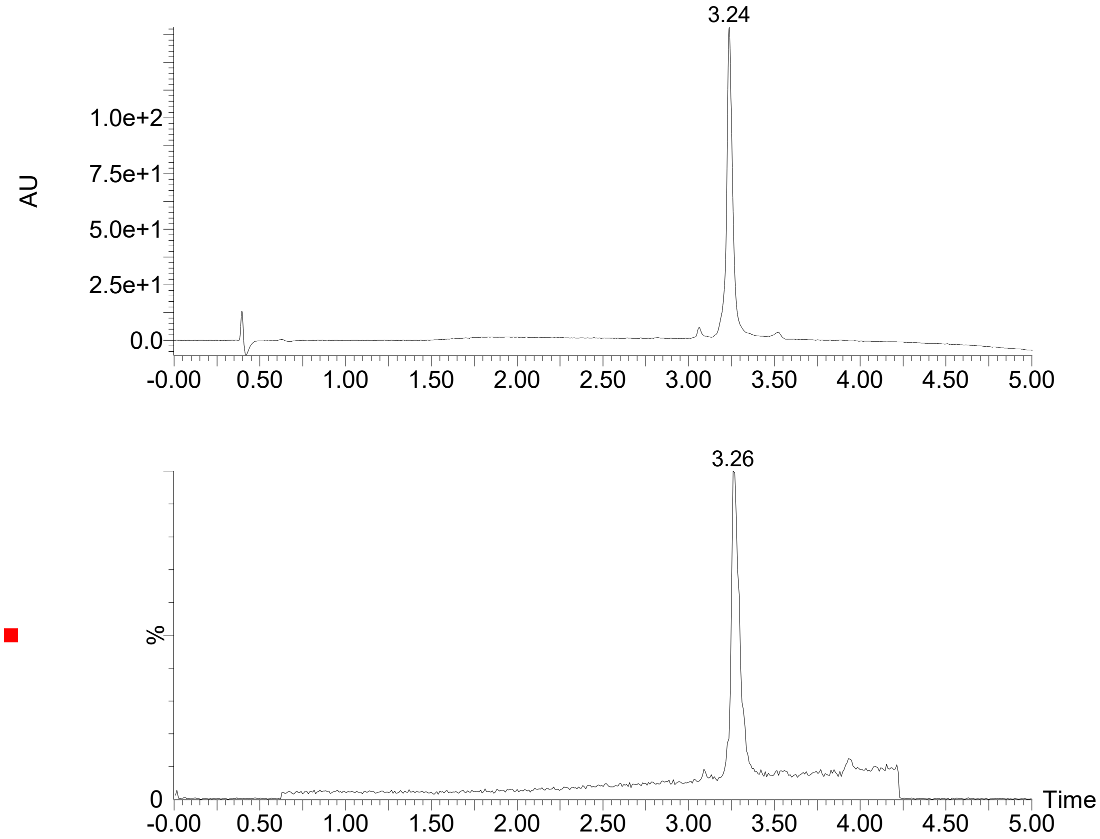

Figure S20. UV (190-400 nm) trace from UPLC-MS analysis of purified **1 (Xaa=Pro, H-SAL)** gradient 5-95% CH_3CN/H_2O containing 0.1% TFA over 5 min at a flow rate of 0.4 mL/min.

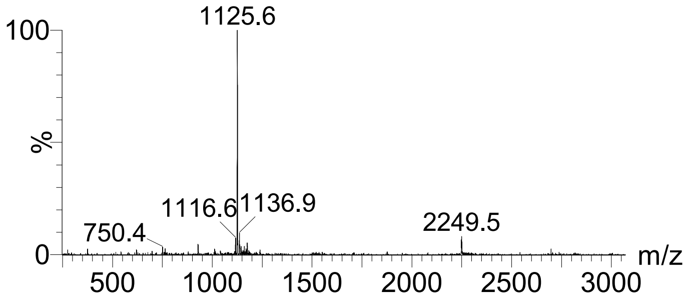

Figure S21. ESI-MS calcd. for C107H145N23O31 $[M+1H]^{1+}$ m/z = 2250.4, found 2249.5; $[M+2H]^{2+}$ m/z = 1125.7, found 1125.6; $[M+3H]^{3+}$ m/z = 750.8, found 750.4.

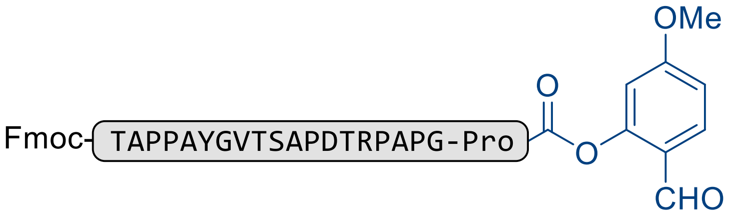

1 (Xaa = Pro, MeO-SAL)

SAL ester **1 (Xaa=Pro, MeO-SAL)** was synthesized according to the general SAL ester preparation method at 0.04~0.08 mmol scale. The crude peptide was purified by preparative reverse-phase HPLC (10-50% CH_3CN/H_2O over 30 min) and lyophilized to afford the desired SAL ester **1 (Xaa=Pro, MeO-SAL)** (26 mg, 38% yield).

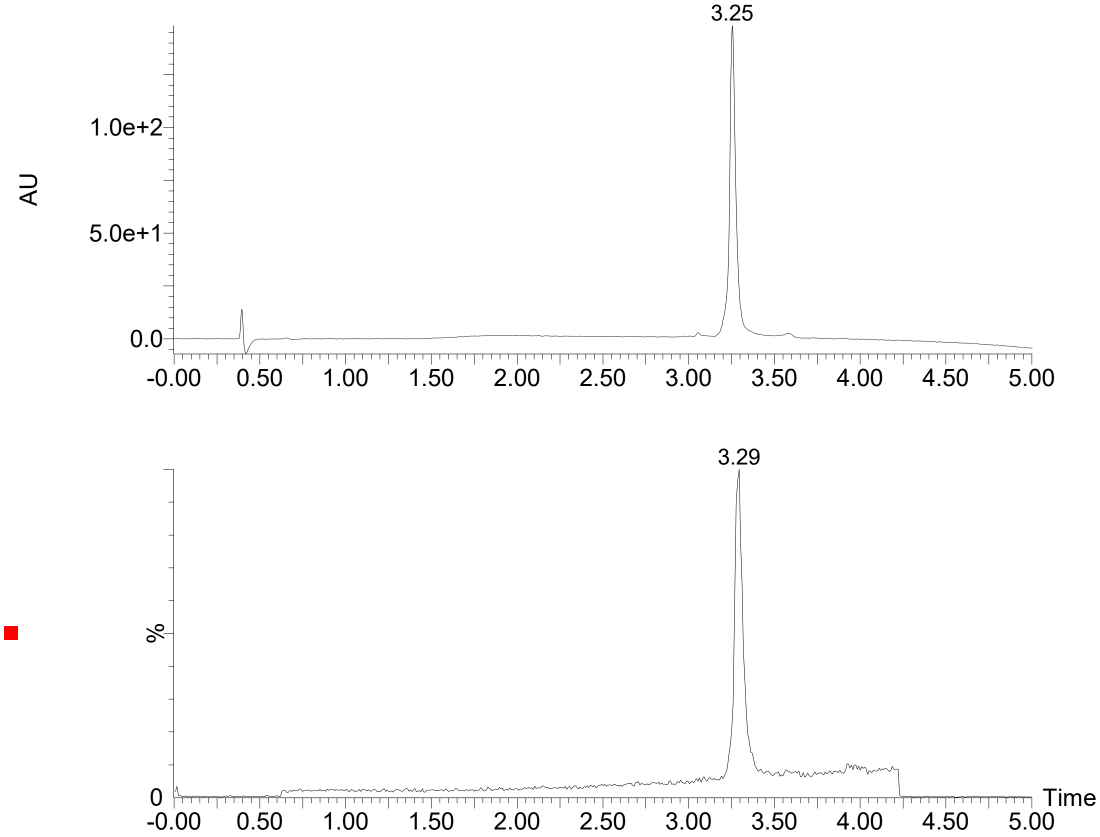

Figure S22. UV (190-400 nm) trace from UPLC-MS analysis of purified **1 (Xaa=Pro, MeO-SAL)** gradient 5-95% CH_3CN/H_2O containing 0.1% TFA over 5 min at a flow rate of 0.4 mL/min.

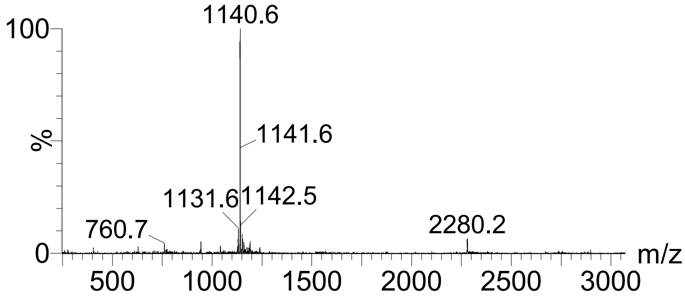

Figure S23. ESI-MS calcd. for C108H147N23O32 $[M+1H]^{1+}$ m/z = 2280.5, found 2280.2; $[M+2H]^{2+}$ m/z = 1140.7, found 1140.6; $[M+3H]^{3+}$ m/z = 760.8, found 760.7.

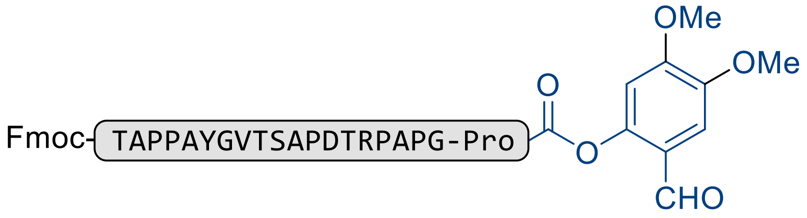

1 (Xaa = Pro, diMeO-SAL)

SAL ester **1 (Xaa=Pro, diMeO-SAL)** was synthesized according to the general SAL ester preparation method at 0.04~0.08 mmol scale. The crude peptide was purified by preparative reverse-phase HPLC (10-50% CH_3CN/H_2O over 30 min) and lyophilized to afford the desired SAL ester **1 (Xaa=Pro, diMeO-SAL)** (22 mg, 31% yield).

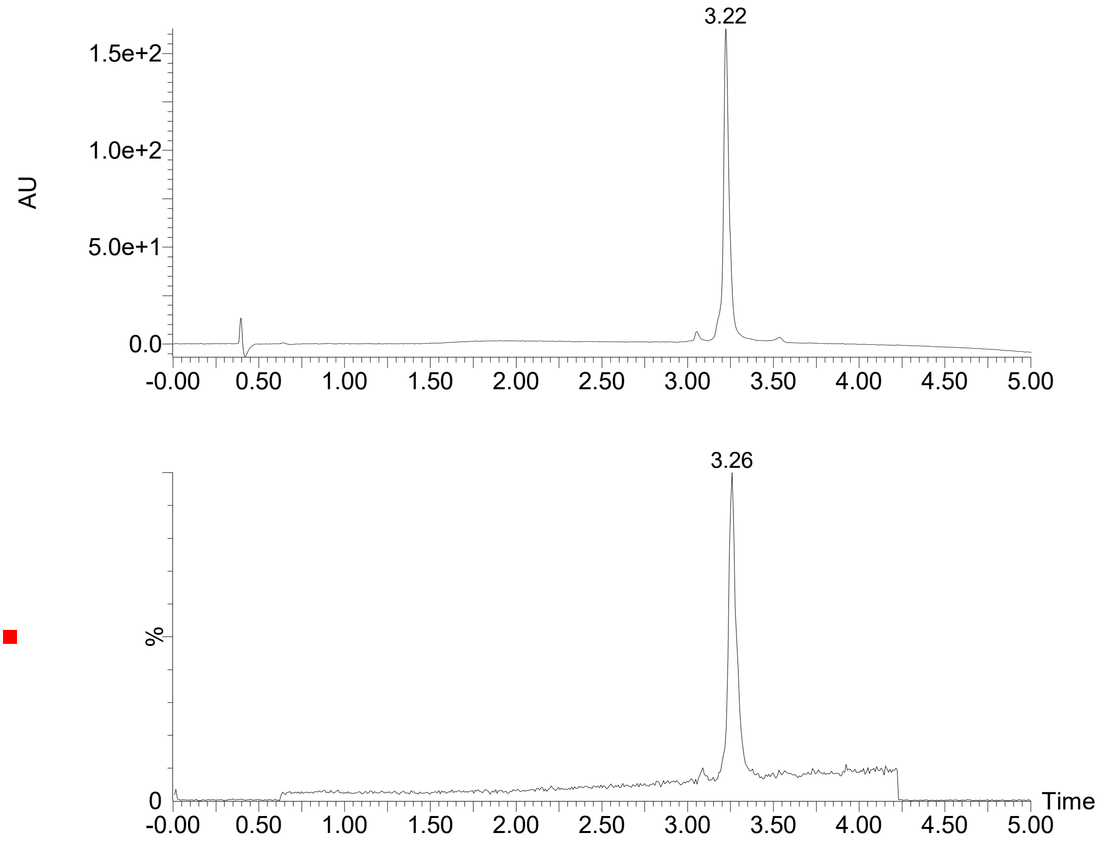

Figure S24. UV (190-400 nm) trace from UPLC-MS analysis of purified **1**

(Xaa=Pro, diMeO-SAL) gradient 5-95% CH_3CN/H_2O containing 0.1% TFA over 5 min at a flow rate of 0.4 mL/min.

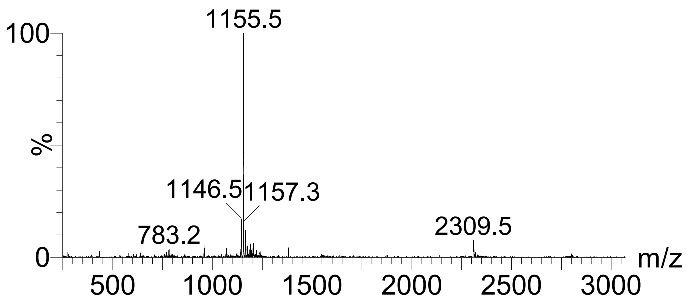

Figure S25. ESI-MS calcd. for C109H149N23O33 $[M+1H]^{1+}$ m/z = 2310.5, found 2309.5; $[M+2H]^{2+}$ m/z = 1155.7, found 1155.5.

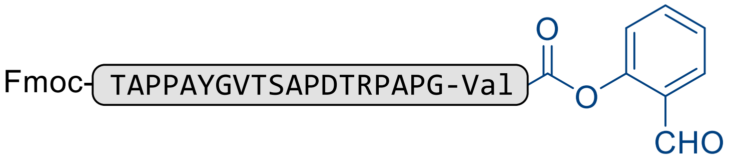

1 (Xaa = Val, H-SAL)

SAL ester **1 (Xaa=Val, H-SAL)** was synthesized according to the general SAL ester preparation method at 0.04~0.08 mmol scale. The crude peptide was purified by preparative reverse-phase HPLC (10-50% CH_3CN/H_2O over 30 min) and lyophilized to afford the desired SAL ester **1 (Xaa=Val, H-SAL)** (37 mg, 34% yield).

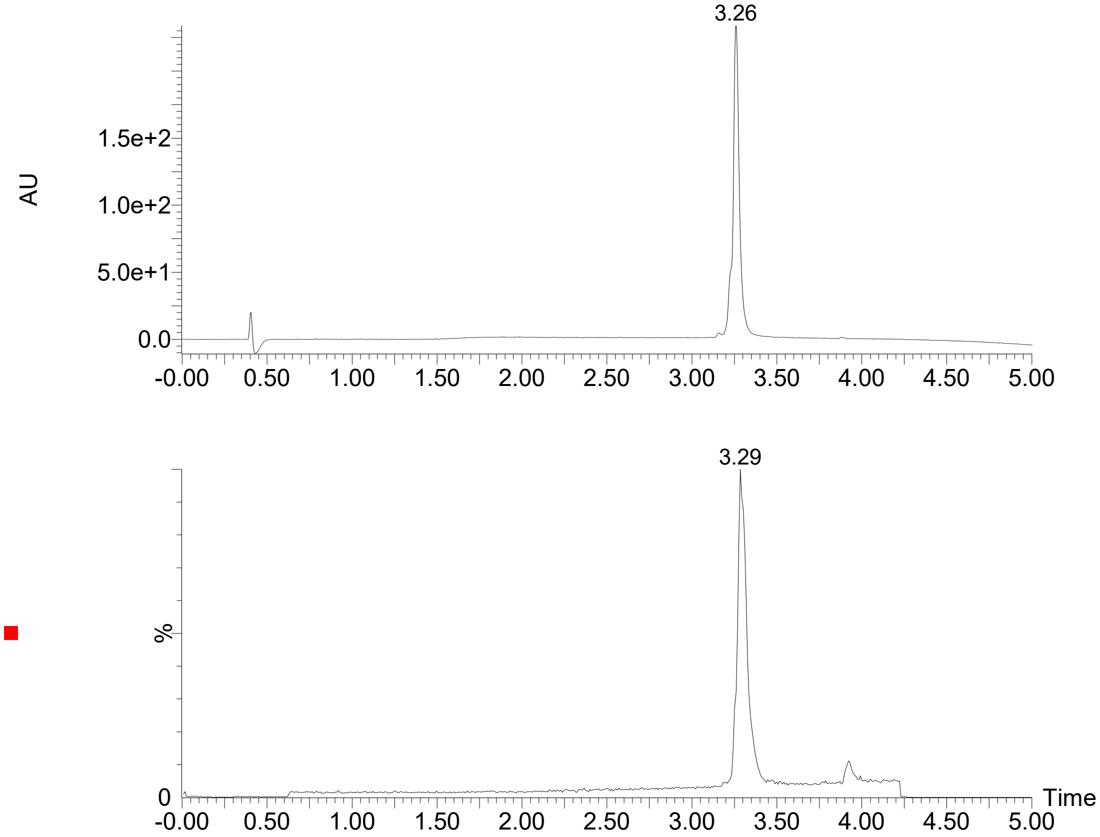

Figure S26. UV (190-400 nm) trace from UPLC-MS analysis of purified **1 (Xaa=Val, H-SAL)** gradient 5-95% CH_3CN/H_2O containing 0.1% TFA over 5 min at a flow rate of 0.4 mL/min.

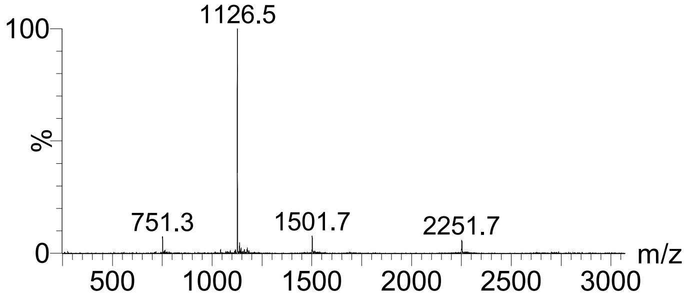

Figure S27. ESI-MS calcd. for C107H147N23O31 $[M+1H]^{1+}$ m/z = 2251.1, found 2251.7; $[M+2H]^{2+}$ m/z = 1126.0, found 1126.5; $[M+3H]^{3+}$ m/z = 751.0, found 751.3.

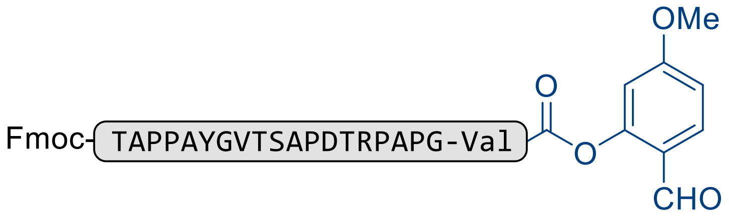

1 (Xaa = Val, MeO-SAL)

SAL ester **1 (Xaa=Val, MeO-SAL)** was synthesized according to the general SAL ester preparation method at 0.04~0.08 mmol scale. The crude peptide was purified by preparative reverse-phase HPLC (10-50% CH_3CN/H_2O over 30 min) and lyophilized to afford the desired SAL ester **1 (Xaa=Val, MeO-SAL)** (38 mg, 32% yield).

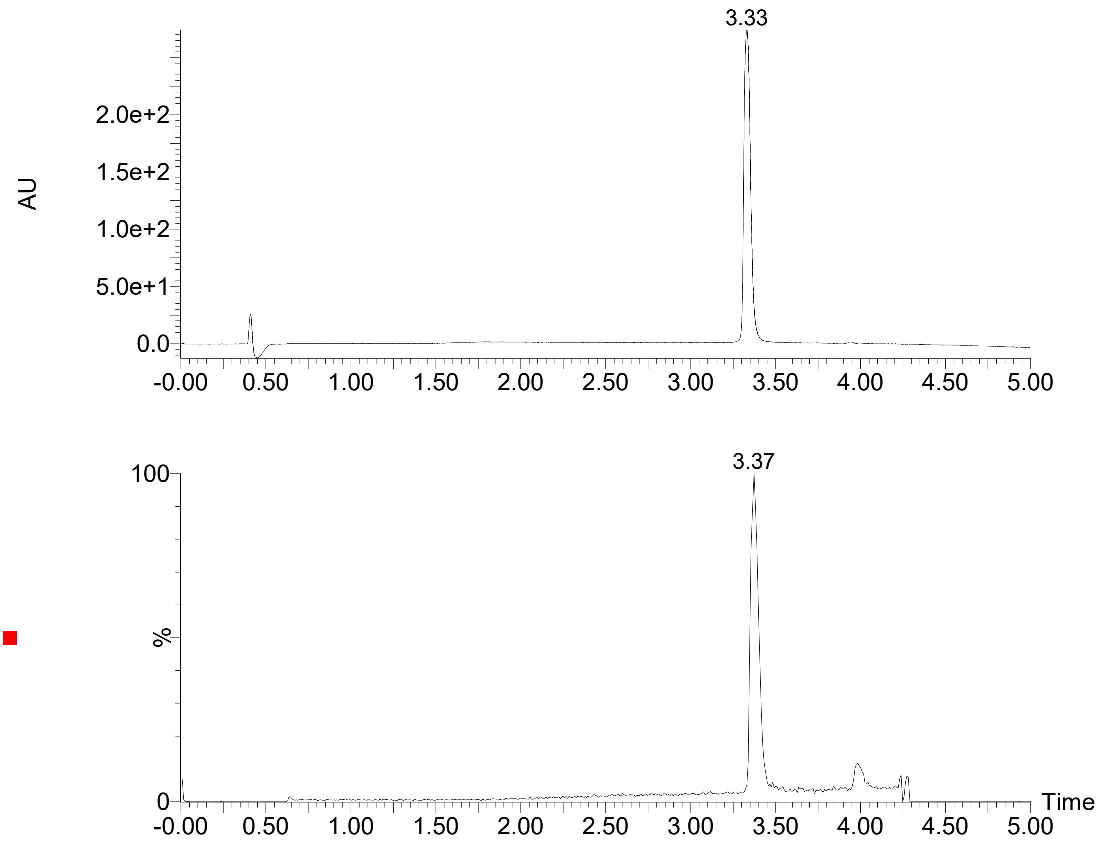

Figure S28. UV (190-400 nm) trace from UPLC-MS analysis of purified **1 (Xaa=Val, MeO-SAL)** gradient 5-95% CH_3CN/H_2O containing 0.1% TFA

over 5 min at a flow rate of 0.4 mL/min.

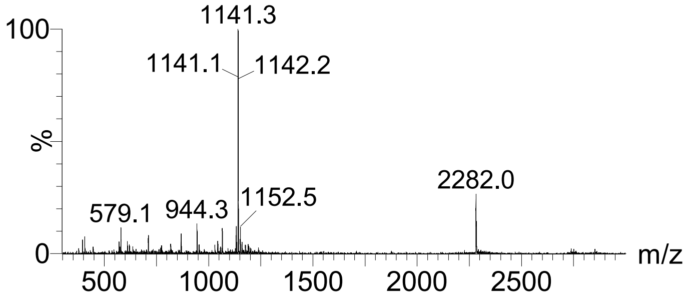

Figure S29. ESI-MS calcd. for C108H149N23O32 $[M+1H]^{1+}$ m/z = 2281.1, found 2282.0; $[M+2H]^{2+}$ m/z = 1141.0, found 1141.3.

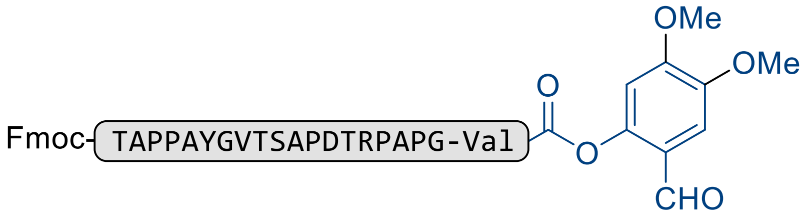

1 (Xaa = Val, diMeO-SAL)

SAL ester **1 (Xaa=Val, diMeO-SAL)** was synthesized according to the general SAL ester preparation method at 0.04~0.08 mmol scale. The crude peptide was purified by preparative reverse-phase HPLC (10-50% CH_3CN/H_2O over 30 min) and lyophilized to afford the desired SAL ester **1 (Xaa=Val, diMeO-SAL)** (37 mg, 33% yield).

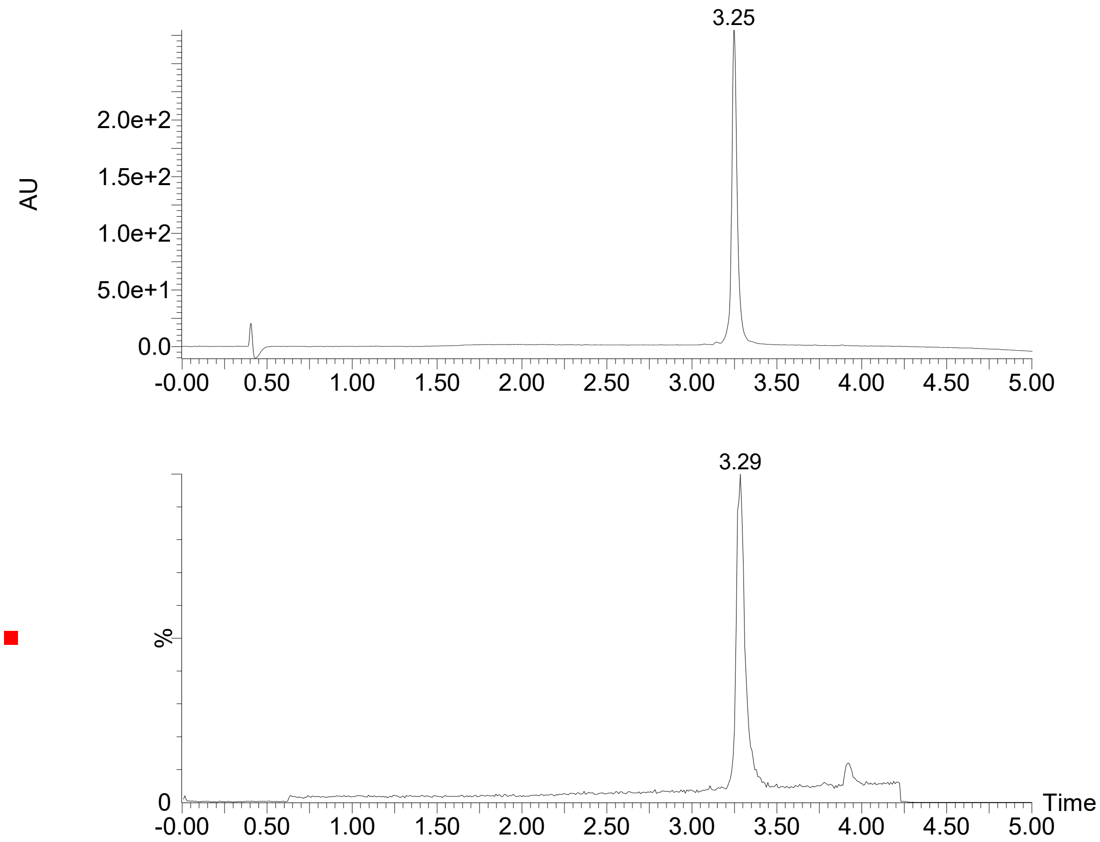

Figure S30. UV (190-400 nm) trace from UPLC-MS analysis of purified **1 (Xaa=Val, diMeO-SAL)** gradient 5-95% CH_3CN/H_2O containing 0.1% TFA over 5 min at a flow rate of 0.4 mL/min.

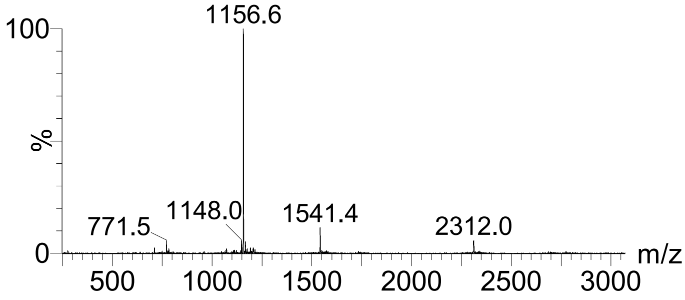

Figure S31. ESI-MS calcd. for C109H151N23O33 $[M+1H]^{1+}$ m/z = 2312.5, found 2312.0; $[M+2H]^{2+}$ m/z = 1156.8, found 1156.6; $[M+3H]^{3+}$ m/z = 771.5, found 771.5.

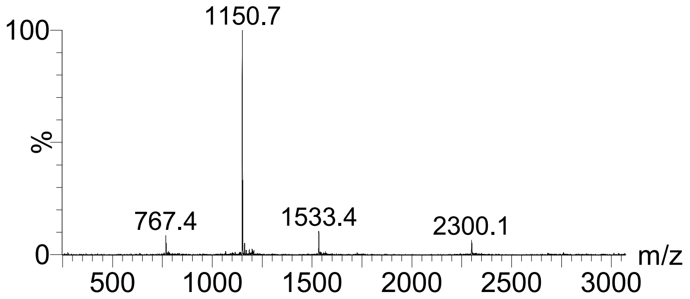

Figure S32. ESI-MS calcd. for C111H147N23O31 $[M+1H]^{1+}$ m/z = 2300.5, found 2300.1; $[M+2H]^{2+}$ m/z = 1150.8, found 1150.7; $[M+3H]^{3+}$ m/z = 767.5, found 767.4.

3.1.2. First trial of AOL

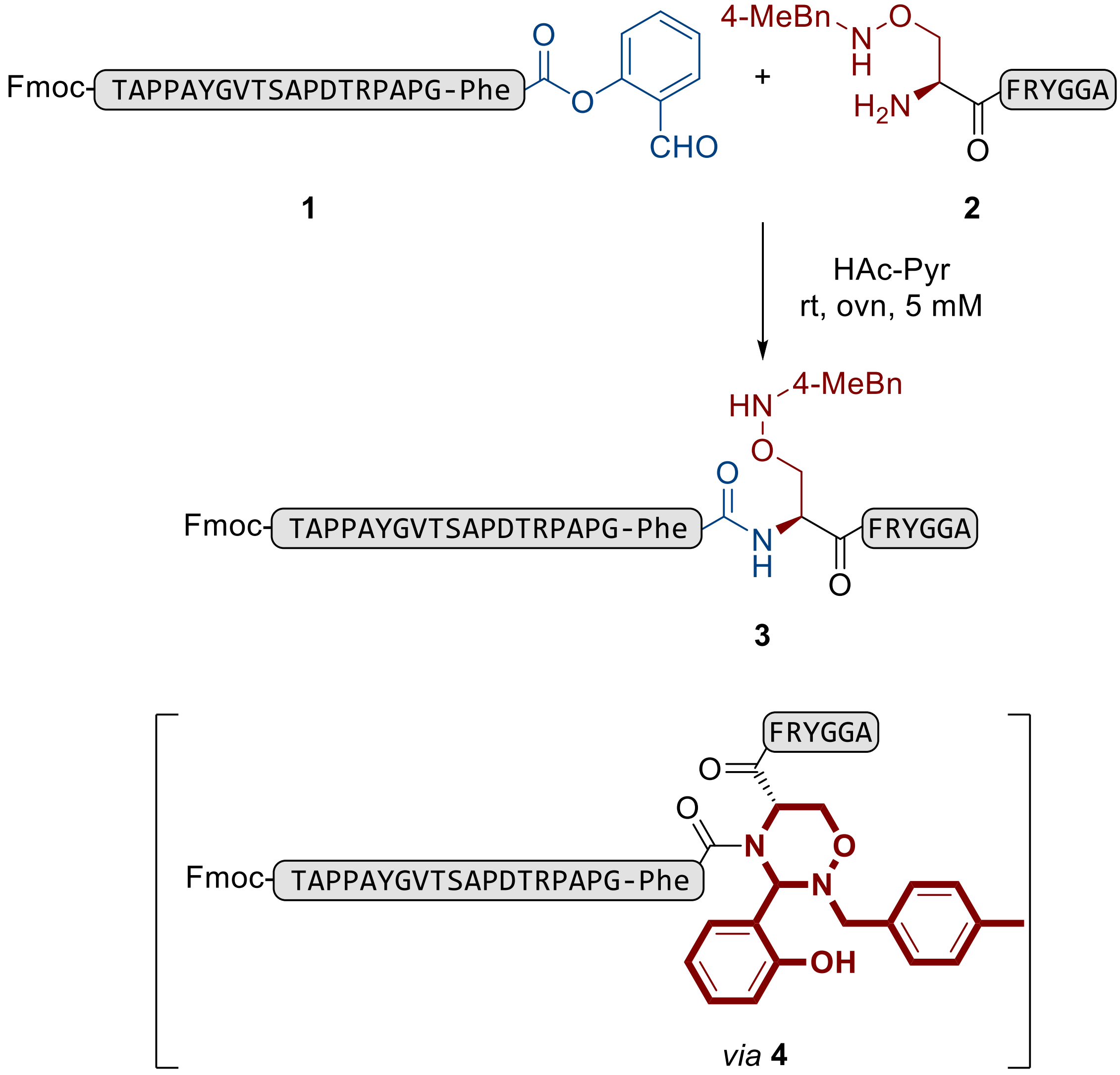

Scheme S3. First observation of AOL in Pyridine-acetic buffer.

Peptide **1 (Xaa=Phe, H-SAL)** (1.0 mg, 0.47 μmol, 1.0 eq.) was dissolved in pyridine-acetic acid buffer (1/1, mol) together with AO peptide **2 (R^3= 4-MethylBenzl, Ser)** (0.41 mg, , 0.47 μmol, 1.0 eq.) at 5 mM concentration. The reaction mixture was stirred under room temperature overnight. The reaction progress was monitored by UPLC-MS.

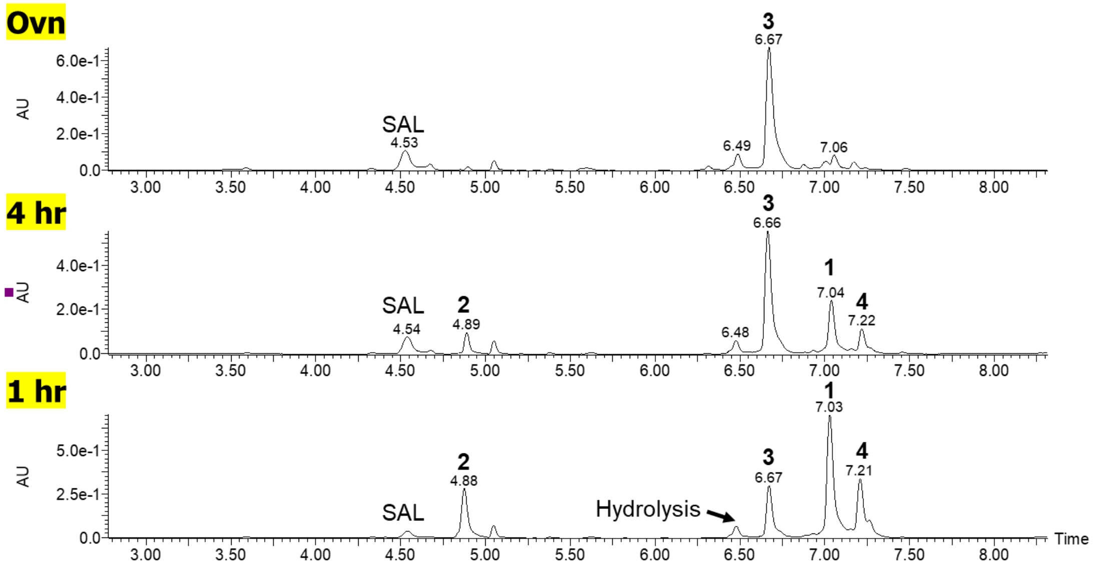

Figure S33. Reaction monitoring by UPLC-MS of first AOL attempt in pyridine-acetic acid buffer conditions for 1 hr, 4hr and overnight (bottom to top). Intermediate **4** was unstable and further converted to acidolysis product **3** by time.

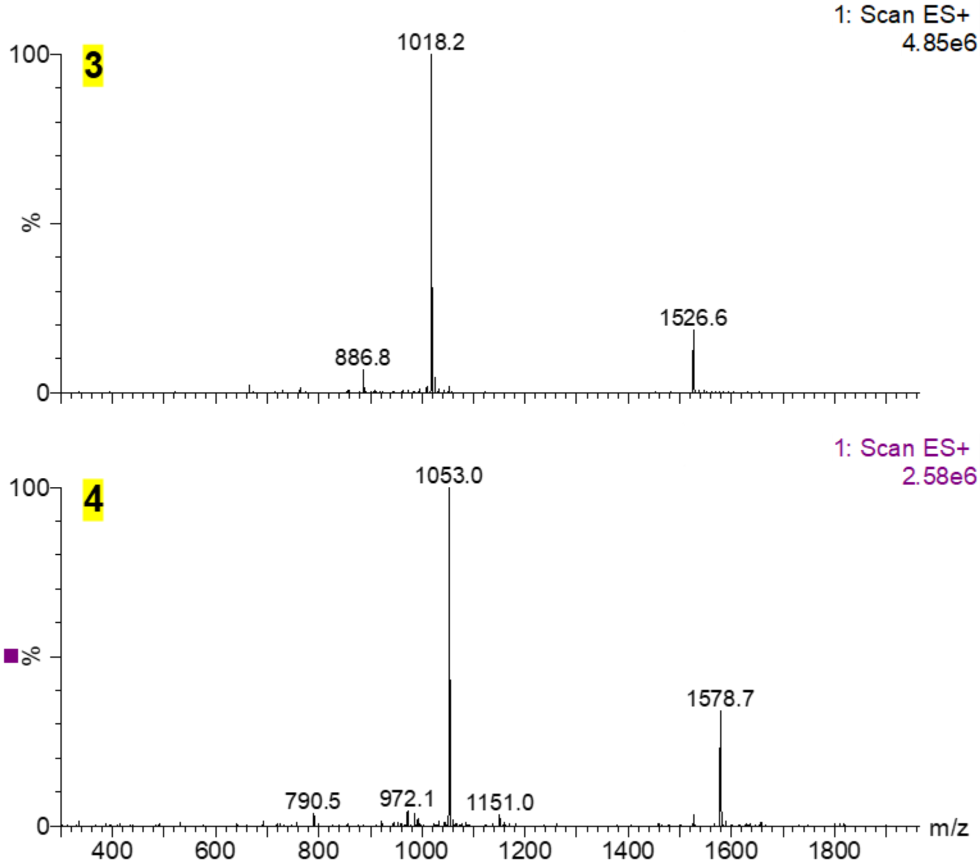

Figure S34. ESI-MS spectra of intermediate **4** (bottom). ESI-MS calcd. for

C153H203N35O39 $[M+2H]^{2+}$ m/z = 1579.2, found 1578.7; $[M+3H]^{3+}$ m/z = 1053.2, found 1053.0. ESI-MS spectra of acidolysis product **3** (top). ESI-MS calcd. for C146H199N35O38 $[M+2H]^{2+}$ m/z = 1527.2, found 1526.6; $[M+3H]^{3+}$ m/z = 1018.5, found 1018.2.

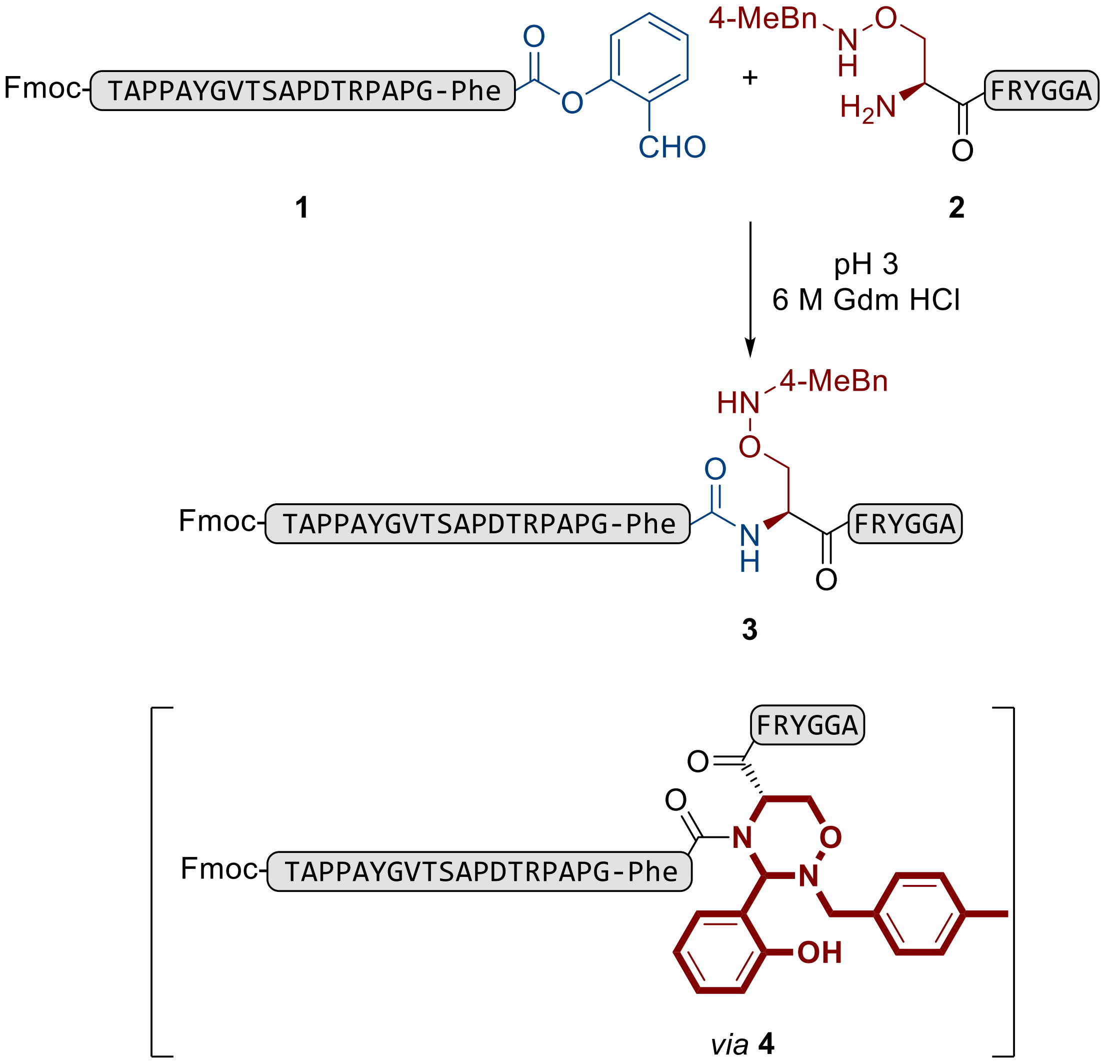

Scheme S4. Observation of AOL in aqueous buffer.

Peptide **1 (Xaa=Phe, H-SAL)** (0.84 mg, 0.37 µmol, 1.0 eq.) was dissolved in pH 3 PB buffer containing 6 M Gdm·HCl together with AO peptide **2 (R^3= 4-MethylBenzl, Ser)** (0.47 mg, , 0.54 µmol, 1.25 eq.) at 5 mM concentration. The reaction mixture was stirred under room temperature overnight. The reaction progress was monitored by UPLC-MS.

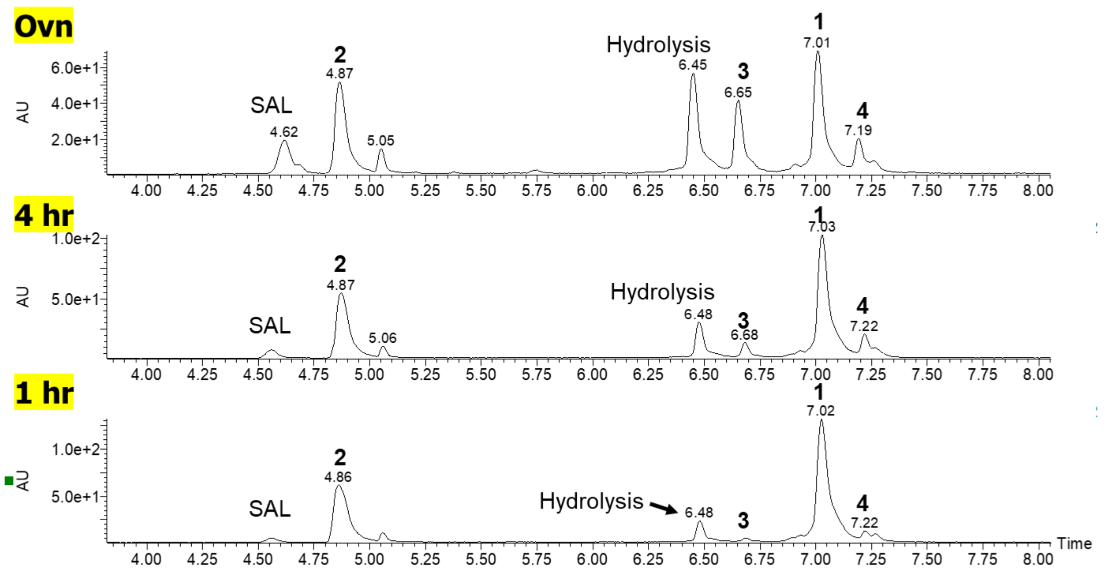

Figure S35. Reaction monitoring by UPLC-MS of the AOL attempt in pH 3 aqueous buffer conditions for 1 hr, 4hr and overnight (bottom to top). Intermediate **4** was unstable and gradually converted to acidolysis product **3** by time.

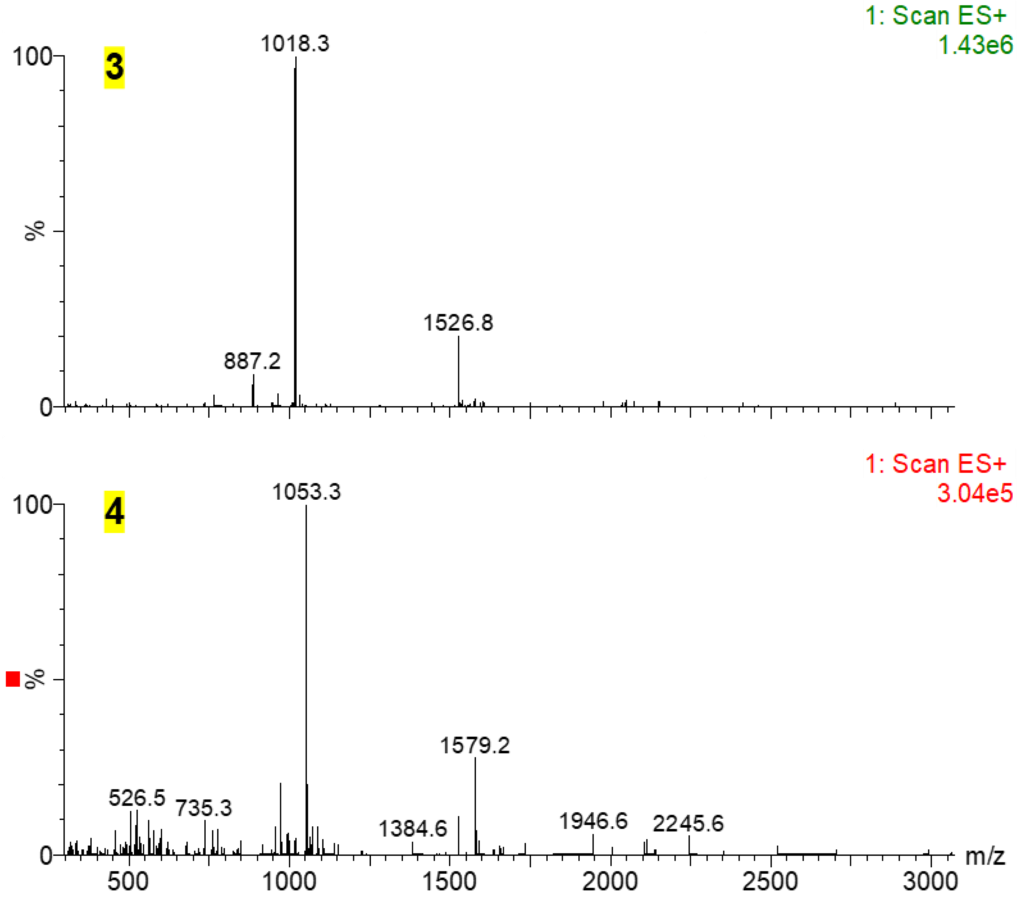

Figure S36. ESI-MS spectra of intermediate **4** (bottom). ESI-MS calcd. for C153H203N35O39 $[M+2H]^{2+}$ m/z = 1579.2, found 1579.2; $[M+3H]^{3+}$ m/z = 1053.2, found 1053.3. ESI-MS spectra of acidolysis product **3** (top). ESI-MS calcd. for C146H199N35O38 $[M+2H]^{2+}$ m/z = 1527.2, found 1526.8; $[M+3H]^{3+}$ m/z = 1018.5, found 1018.3.

3.2. Optimization of AOL

3.2.1. Condition screenings of AOL

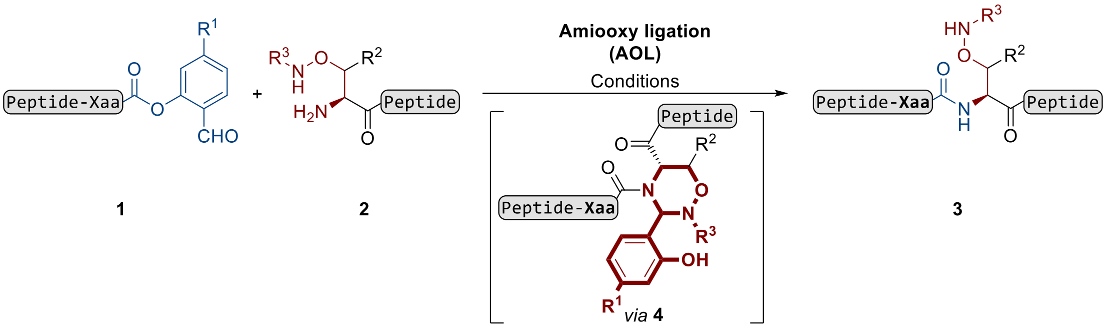

Scheme S5. Scheme of condition screenings of AOL

Peptide **1** (0.30~1.0 μmol, 1.0 eq.) was dissolved in various buffers with AO peptide **2** (0.45~1.5 μmol, 1.25 eq.) at 5 mM concentration. The reaction mixture was stirred under room temperature overnight. The reaction progress was monitored by UPLC-MS.

Entry	Xaa	R^1	R^2	R^3	Solvent	Conversion%[a]
1	Phe	H	H	Bn	Pyr/HAc (1:1, vol)	89%
2	Phe	H	H	Bn	10% Pyr/HAc (1:1, vol) in DMSO	87%
3	Phe	H	H	Bn	pH 4.5 citrate buffer with 6M GdmHCl	27%
4	Phe	H	H	Bn	pH 5.5 Acetate buffer with 6M	5%

					GdmHCl	
4	Phe	H	H	Bn	pH 6.2 PBS buffer with 6M GdmHCl	3%
5	Phe	MeO	H	Bn	Pyr/HAc (1:1, vol)	91%
6	Phe	MeO	H	Bn	10% Pyr/HAc (1:1, vol) in DMSO	89%
7	Phe	MeO	H	Bn	pH 4.5 citrate buffer with 6M GdmHCl	83%
8	Phe	MeO	H	Bn	pH 5.5 Acetate buffer with 6M GdmHCl	5%
9	Phe	diMeO	H	Bn	pH 6.2 PBS buffer with 6M GdmHCl	3%
10	Phe	diMeO	H	Bn	Pyr/HAc (1:1, vol)	90%
11	Phe	diMeO	H	Bn	10% Pyr/HAc (1:1, vol) in DMSO	18%
12	Phe	diMeO	H	Bn	pH 4.5 citrate buffer with 6M GdmHCl	55%
13	Phe	diMeO	H	Bn	pH 5.5 Acetate buffer with 6M GdmHCl	2%
14	Phe	diMeO	H	Bn	pH 6.2 PBS buffer with 6M GdmHCl	N.D.
15	Val	H	H	Bn	10% Pyr/HAc (1:1, vol) in DMSO	75%
16	Pro	H	H	Bn	10% Pyr/HAc (1:1, vol) in DMSO	73%
17	Ile	H	H	Bn	Pyr/HAc (1:1, vol)	86%

18	Val	H	H	Bn	Pyr/HAc (1:1, vol)	82%
19	Gly	H	H	Bn	Pyr/HAc (1:1, vol)	92%
20	Pro	H	H	Bn	Pyr/HAc (1:1, vol)	78%
21	Gly	MeO	H	Bn	pH 4.5 citrate buffer with 6M GdmHCl	89%
22	Ile	MeO	H	Bn	pH 4.5 citrate buffer with 6M GdmHCl	25%
23	Pro	MeO	H	Bn	pH 4.5 citrate buffer with 6M GdmHCl	28%
24	Pro	MeO	H	Me	pH 4.5 citrate buffer with 6M GdmHCl	75%
25	Phe	MeO	Me	Me	pH 4.5 citrate buffer with 6M GdmHCl	81%[b]
26	Phe	MeO	Me	Me	Pyr/HAc (1:1, vol)	91%[b]
27	Phe	MeO	H	H	pH 4.5 citrate buffer with 6M GdmHCl	<5%[c]
28	Phe	MeO	H	Ser in place of AOSer	pH 4.5 citrate buffer with 6M GdmHCl	N.D.[d]
28	Phe	MeO	H	Ser in place of AOSer	Pyr/HAc (1:1, vol)	92%

Notes: [a] Conversion analyzed by UPLC-MS; [b] Peptide **1** (Ac-YRVIG-Phe-) adopted; [c] Reaction halted at oxime stage; [d] no desired product detected.

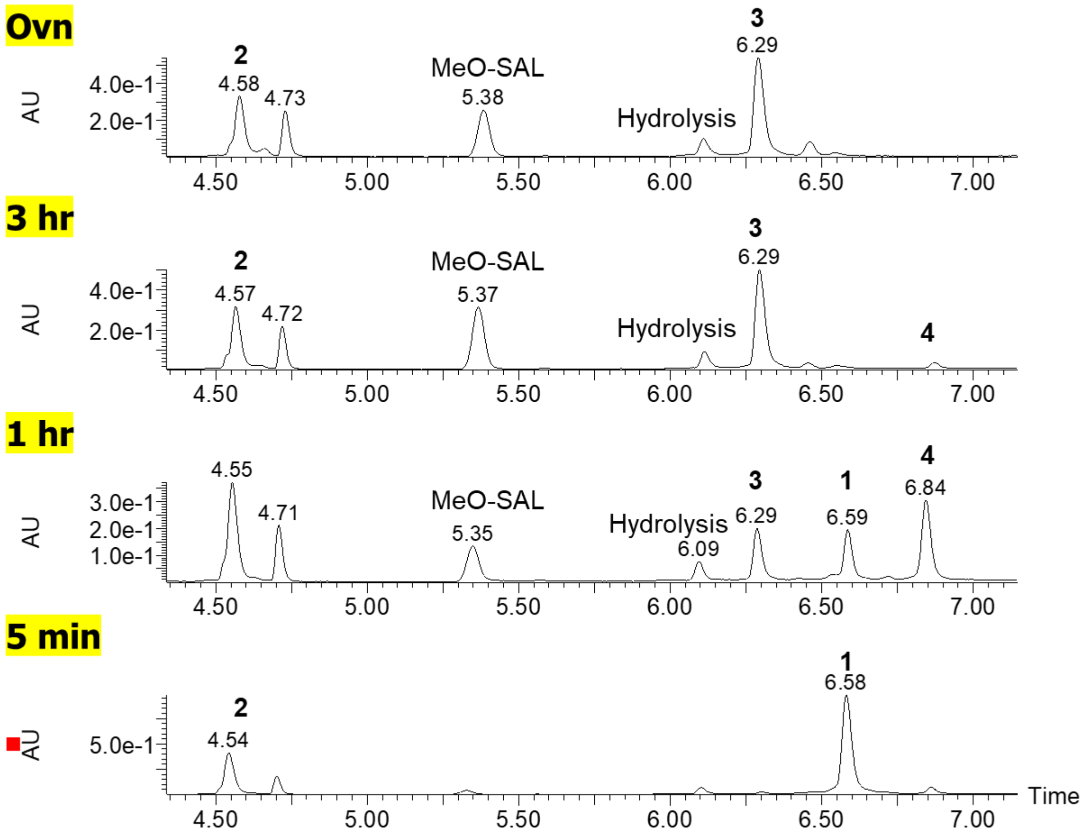

Figure S37. Reaction monitoring by UPLC-MS of the AOL between peptidyl SAL ester **1 (Xaa=Gly, MeO-SAL)** and AOSer **2 (R^3= Bn, Ser)** under pH 4.5 citrate buffer containing 6 M GdmHCl. Intermediate **4** was unstable and gradually converted to acidolysis product **3** by time.

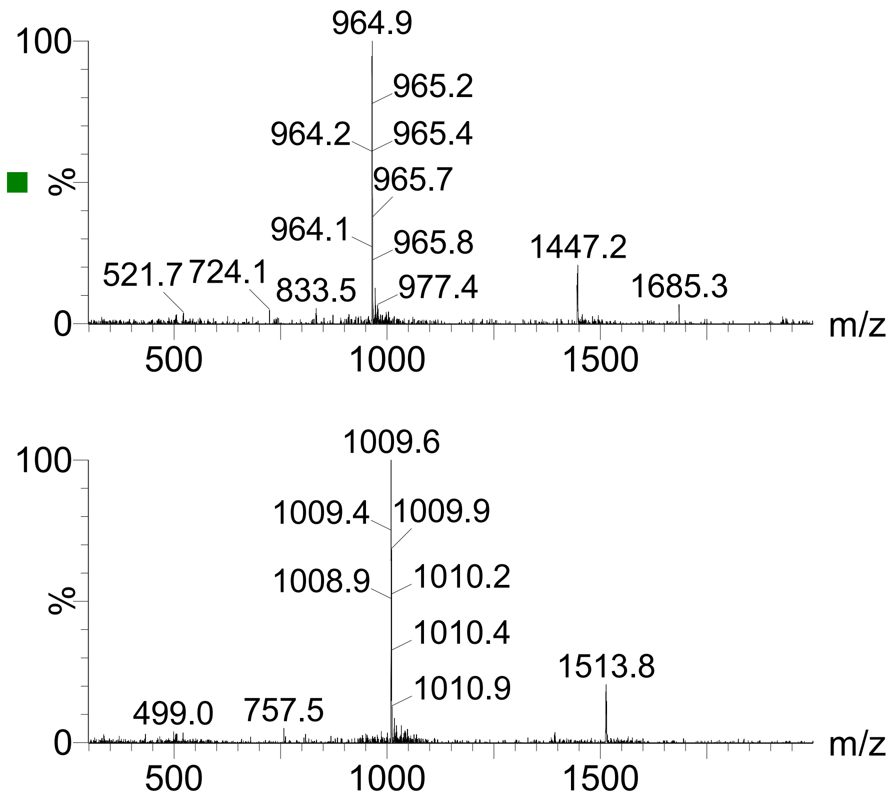

Figure S38. ESI-MS spectra of intermediate **4** (bottom). ESI-MS calcd. for C144H194N34O39 $[M+2H]^{2+}$ m/z = 1513.7, found 1513.8; $[M+3H]^{3+}$ m/z = 1009.4, found 1009.6. ESI-MS spectra of acidolysis product **3** (top). ESI-MS calcd. for C136H188N34O37 $[M+2H]^{2+}$ m/z = 1446.6, found 1447.2; $[M+3H]^{3+}$ m/z = 964.7, found 1964.9; $[M+4H]^{4+}$ m/z = 723.8, found 724.1.

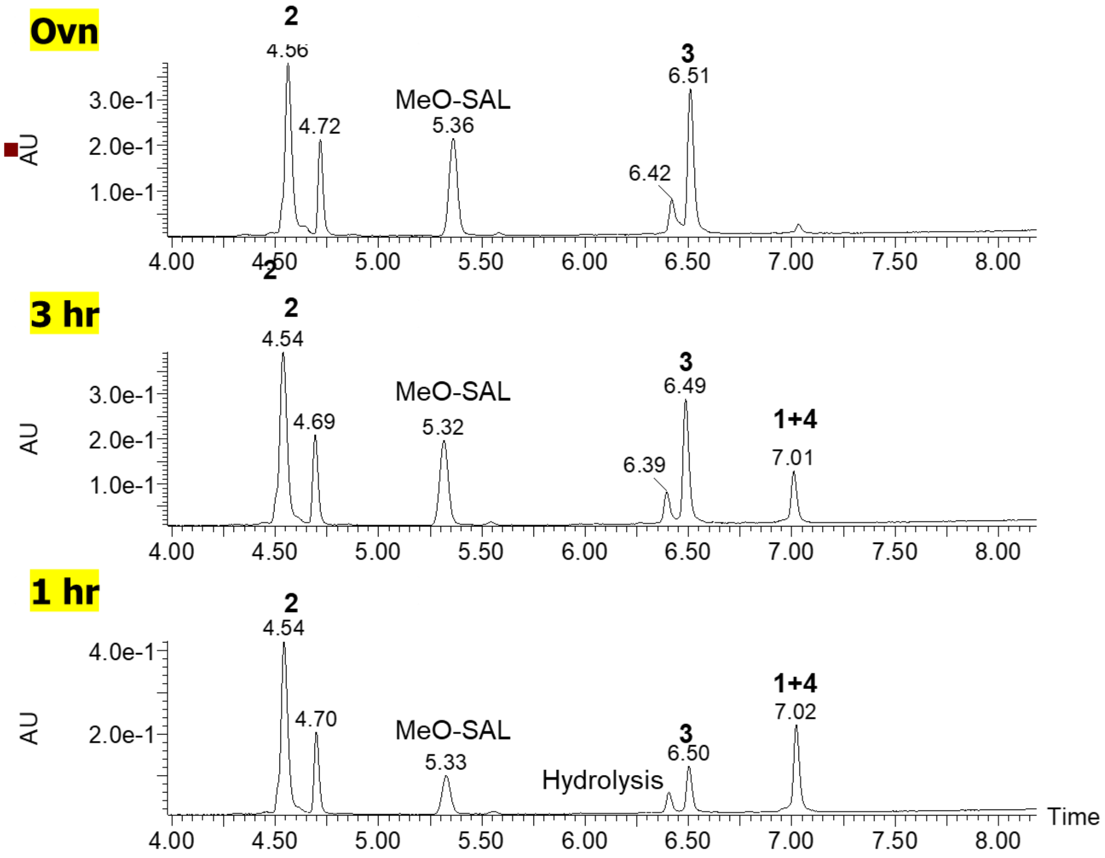

Figure S39. Reaction monitoring by UPLC-MS of the AOL between peptidyl SAL ester **1 (Xaa=Phe, MeO-SAL)** and AOSer **2 (R^3= Bn, Ser)** under pH 4.5 citrate buffer containing 6 M GdmHCl.

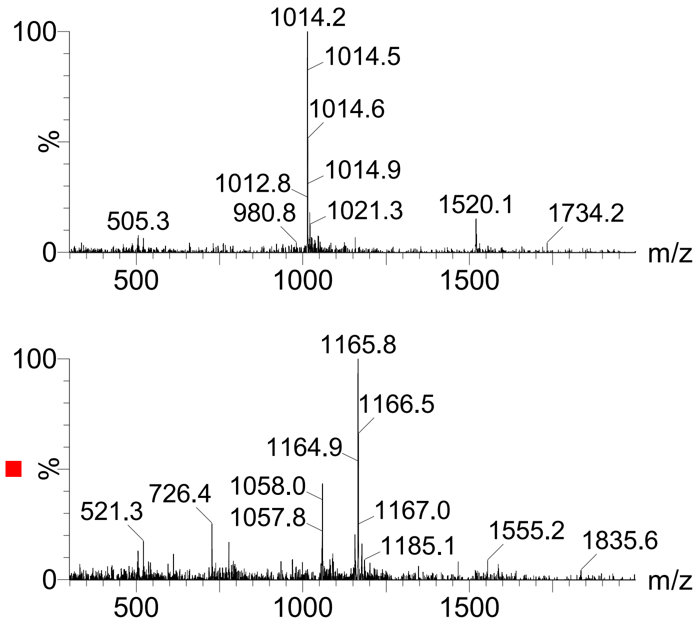

Figure S40. ESI-MS spectra of intermediate **4** (bottom) mixed with SAL ester **1**. ESI-MS calcd. for C153H203N35O40 $[M+3H]^{3+}$ m/z = 1058.5, found 1058.0. ESI-MS spectra of acidolysis product **3** (top). ESI-MS calcd. for C145H197N35O38 $[M+2H]^{2+}$ m/z = 1520.2, found 1520.1; $[M+3H]^{3+}$ m/z = 1013.8, found 1014.2.

3.2.2. Epimerization study of aminooxy ligation

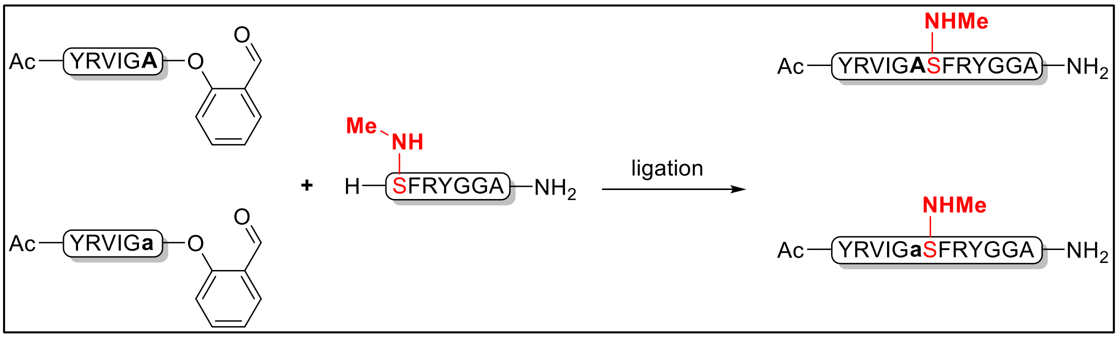

Scheme S6. Epimerization study of AOL

An epimerization study of aminooxy ligation at C-terminal was performed using Ac-YRVIGA-CO-SAL ester and Ac-YRVIGa-CO-SAL ester reacting with H-(AO, Me)FRYGGA-NH_2, respectively (see below). These ligated products were purified via preparative reverse phase HPLC (10-40% CH_3CN/H_2O over 45 min,

0.1% TFA) followed by lyophilization to obtain the pure ligated products respectively. No epimerization at C-terminal during ligation was observed.

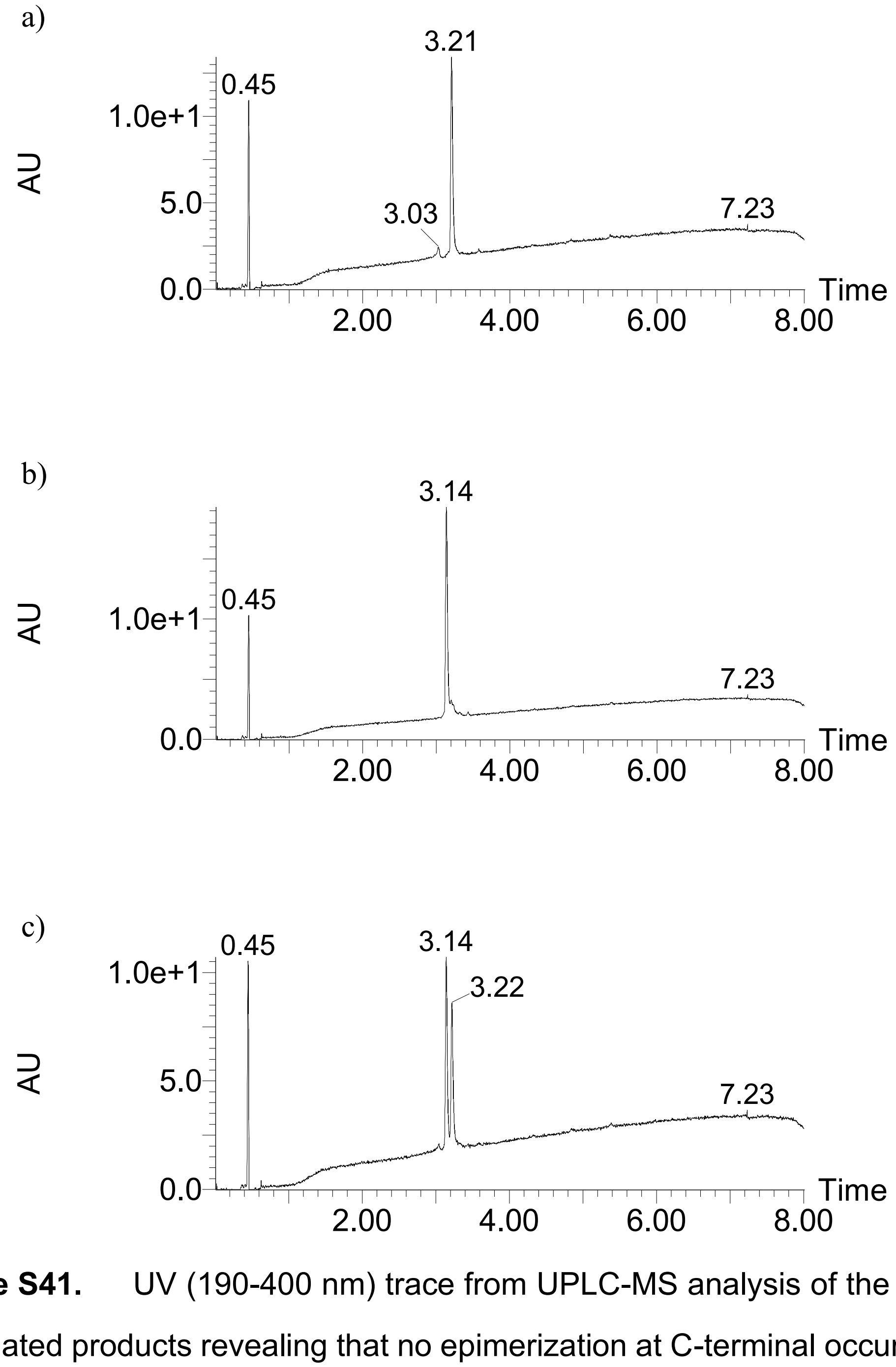

Figure S41. UV (190-400 nm) trace from UPLC-MS analysis of the purified ligated products revealing that no epimerization at C-terminal occurred. (a) purified Ac-YRVIG**A**(AO, Me)FRYGGA-NH_2; (b) purified Ac-YRVIG**a**(AO,

Me)FRYGGA-NH_2; (c) Co-injection of two ligation products gradient 5-75% CH_3CN/H_2O containing 0.1% TFA over 8 min at a flow rate of 0.4 mL/min.

3.3. Aminooxy ligation with thioester

3.3.1. Synthesis of model C-terminus peptide thioesters

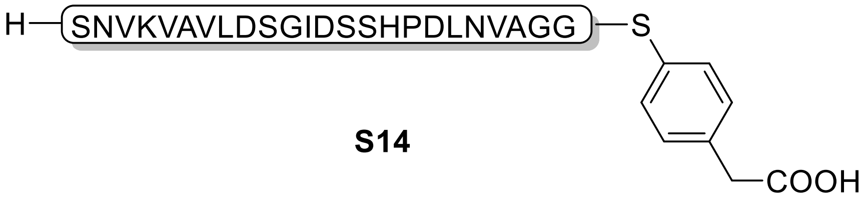

S14

H-SNVKVAVLDSGIDSSHPDLNVAGG-MPAA **S14** was synthesized according to the standard protocol of SPPS at 0.05 mmol scale. The crude peptide was treated with MPAA (42 mg, 0.25 mmol) and AcAc (12.5 mg, 0.125 mmol). The pH was adjusted to 2 and the reaction mixture was stirred under room temperature for 3 h and was purified by preparative reverse-phase HPLC (25-60% CH_3CN/H_2O over 45 min) and lyophilized to afford the desired peptide **S14** (48.4 mg, 38.7% yield).

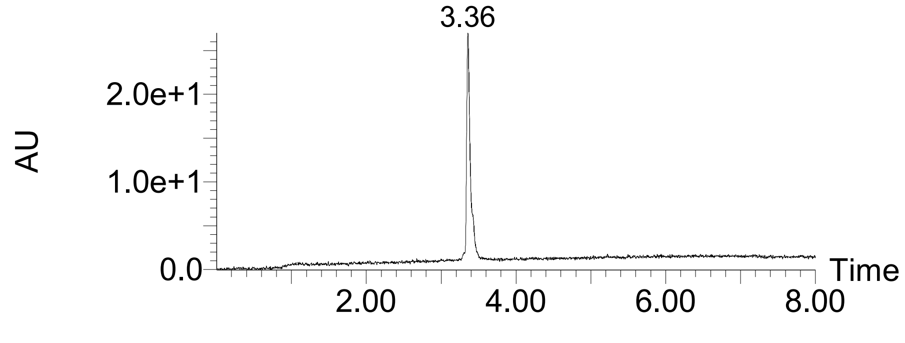

Figure S42. UV (190-400 nm) trace from UPLC-MS analysis of purified H-SNVKVAVLDSGIDSSHPDLNVAGG-MPAA **S14** gradient 5-95% CH_3CN/H_2O containing 0.1% TFA over 5 min at a flow rate of 0.4 mL/min.

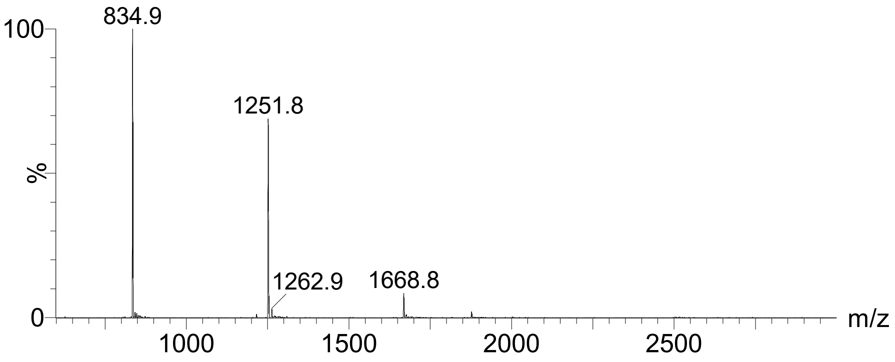

Figure S43. ESI-MS calcd. for $C_{107}H_{169}N_{29}O_{38}S$ $[M+2H]^{2+}$ m/z = 1251.9, found 1251.8; $[M+3H]^{3+}$ m/z = 834.9, found 834.9.

3.3.2. AOL with thioester

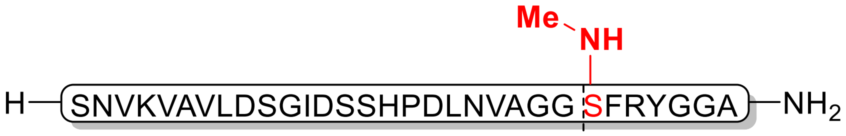

S16 *Ligation site*

The ligation between H-SNVKVAVLDSGIDSSHPDLNVAGG-MPAA **S14** (3 mg, 1.2 μmol) and H-(AO,Me)FRYGGA-NH_2 **S15** (1.9 mg, 2.4 μmol) was dissolved in 6 M Guanidine-HCl and 0.2 M NaH_2PO_4 buffer (5 mM), the pH was adjusted to 6.8 and the reaction was incubated at 25°C for 48 h. Purification via preparative reverse phase HPLC (10-45% CH_3CN/H_2O over 45 min, 0.1% TFA) followed by lyophilization afforded H-SNVKVAVLDSGIDSSHPDLNVAGG(AO,Me)FRYGGA-NH_2 **S16** (1.28 mg, 34.2 % yield) as white solids.

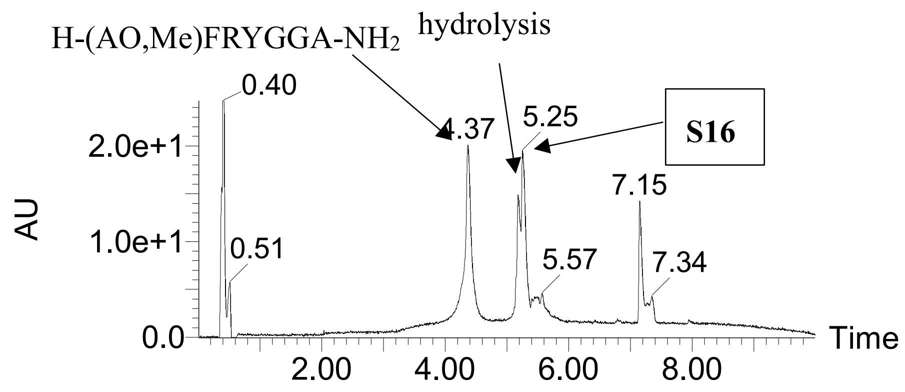

Figure S44. UV (190-400 nm) trace from UPLC-MS analysis of synthesis of **S16** gradient 5-75% CH_3CN/H_2O containing 0.1% TFA over 10 min at a flow rate of 0.4 mL/min.

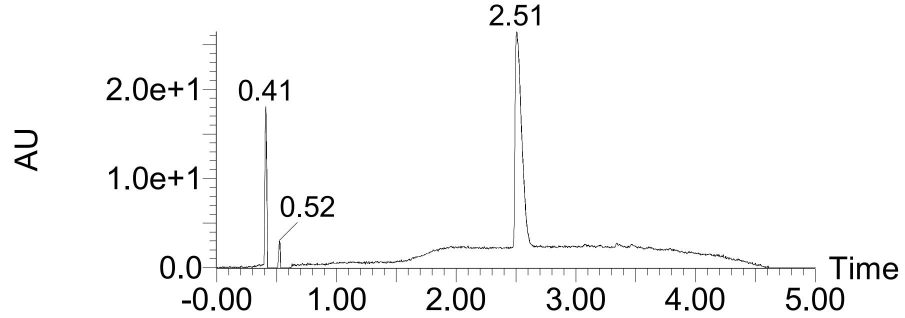

Figure S45. UV (190-400 nm) trace from UPLC-MS analysis of purified **S16** gradient 5-95% CH_3CN/H_2O containing 0.1% TFA over 5 min at a flow rate of 0.4 mL/min.

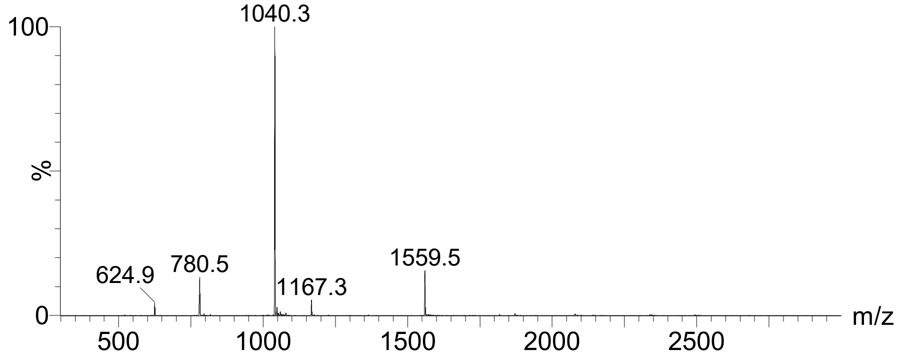

Figure S46. ESI-MS calcd. for $C_{134}H_{213}N_{41}O_{45}$ $[M+2H]^{2+}$ m/z = 1560.2, found 1559.5; $[M+3H]^{3+}$ m/z = 1040.3, found 1040.3; $[M+4H]^{4+}$ m/z = 780.6, found 780.5; $[M+5H]^{5+}$ m/z = 624.7, found 624.9.

3.3.3. Deamination of AOSer

H—(SNVKVAVLDSGIDSSHPDLNVAGG SFRYGGA)—NH_2

S17

H-SNVKVAVLDSGIDSSHPDLNVAGG(AO,Me)FRYGGA-NH_2 **S16** (0.13 mg, 0.0417 μmol) was treated with Zn powder (10 mg) in 1 M ascorbic acid aqueous solution and was incubated under room temperature for 8 h. Small amount of reaction mixture was aliquot and was analyzed by UPLC.

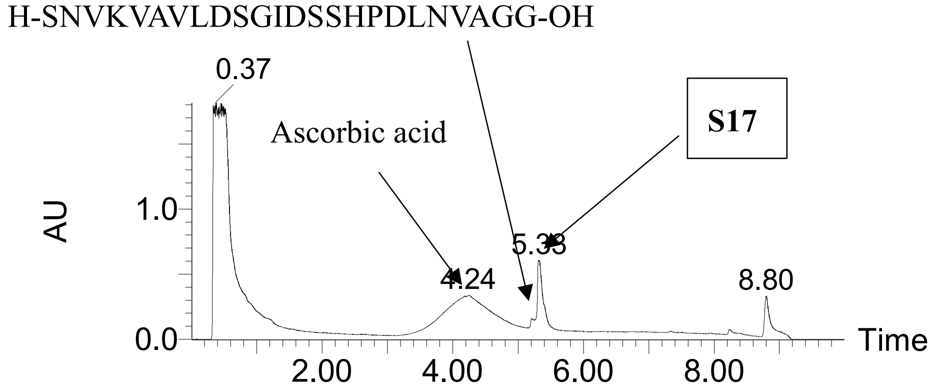

Figure S47. UV (206 nm) trace from UPLC-MS analysis of reaction of **S16** with Zn at 8 h gradient 5-75% CH_3CN/H_2O containing 0.1% TFA over 10 min at a flow rate of 0.4 mL/min.

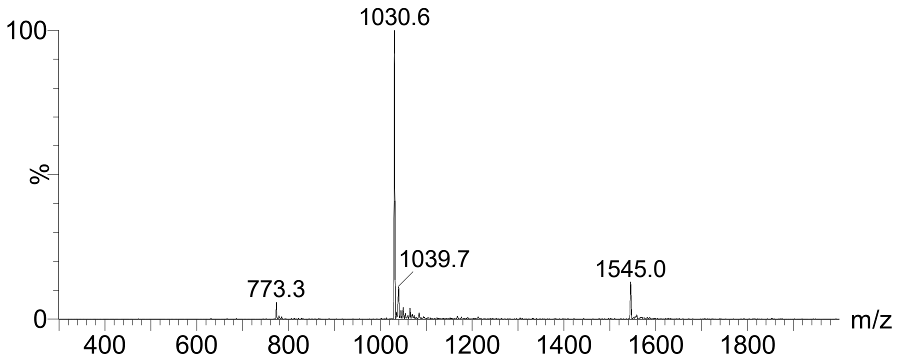

Figure S48. ESI-MS calcd. for $C_{133}H_{210}N_{40}O_{45}$ $[M+2H]^{2+}$ m/z = 1545.7, found 1545.0; $[M+3H]^{3+}$ m/z = 1030.8, found 1030.6; $[M+4H]^{4+}$ m/z = 773.3, found 773.3.

4. Discovery and development of CEL

4.1. Discovery of CEL

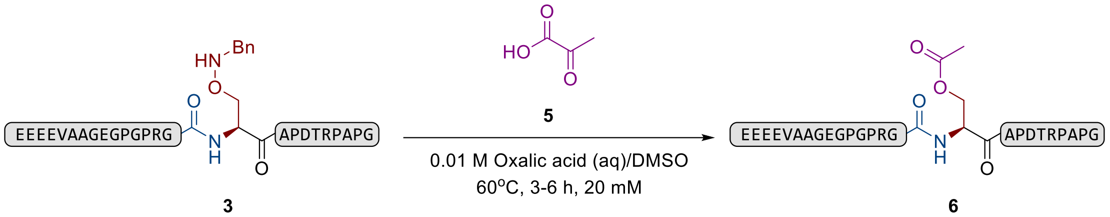

Scheme S7. Scheme of the first attempt to CEL.

AOSer-containing peptide **3** (0.2 mg, 67 nmol, 1.0 eq.) was first dissolved in 80% DMSO under 0.01 M oxalic acid aqueous solution at 20 mM concentration, together with pyruvic acid (5.0 eq., 100 mM). The reaction mixture was incubated in water bath under 60°C for 3 hr. Reaction progress was then monitored by UPLC-MS.

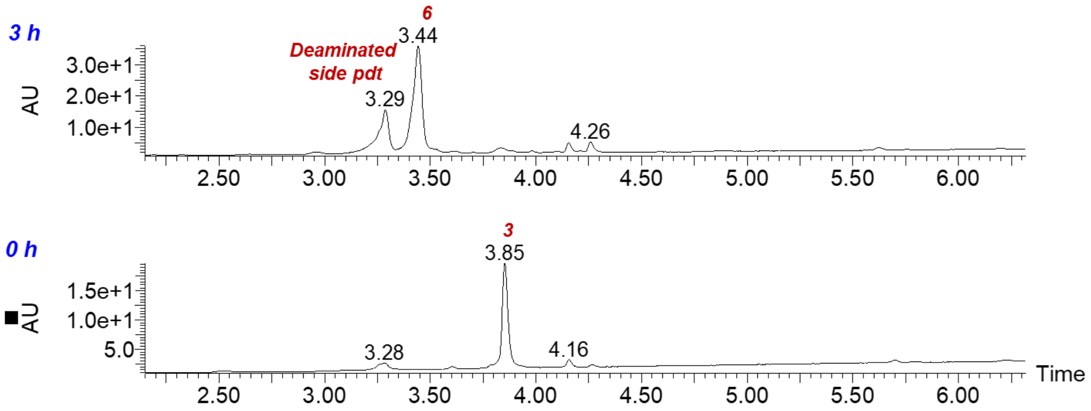

Figure S49. Reaction monitoring by UPLC-MS of first CEL attempt in aqueous oxalic acid/DMSO conditions for 0 hr and 3hr (bottom to top).

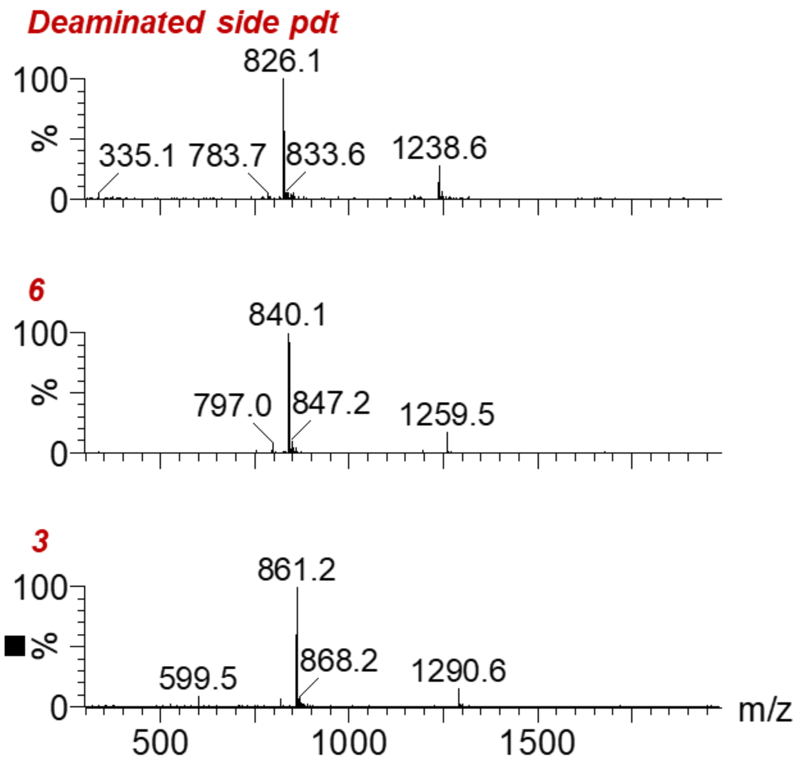

Figure S50. ESI-MS spectra of AOSer peptide **3**(bottom). ESI-MS calcd. for C109H166N32O41 $[M+2H]^{2+}$ m/z = 1291.3, found 1290.6; $[M+3H]^{3+}$ m/z = 861.2, found 861.2. ESI-MS spectra of ester peptide **6** (middle). ESI-MS calcd. for C104H161N31O42 $[M+2H]^{2+}$ m/z = 1259.8, found 1259.5; $[M+3H]^{3+}$ m/z = 840.2, found 840.1. ESI-MS spectra of deamination side product (top). ESI-MS calcd. for C102H159N31O41 $[M+2H]^{2+}$ m/z = 1238.8, found 1238.6; $[M+3H]^{3+}$ m/z = 826.2, found 826.1.

4.2. Optimization of CEL

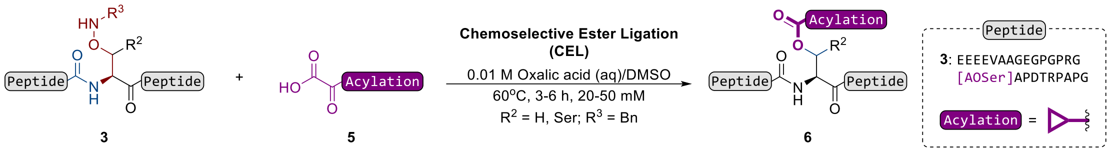

Entry	Deviation from above conditions	Conversion[a]
1	None	81%
2	ACN/H_2O (3/1) with 0.01 M Oxalic acid	55%
3	2.5% TFA in place of 0.01 M Oxalic acid	65%
4	Acylation = Me	75%
5	Acylation = Cyclohexyl	74%[b]
6	Acylation = Isopropyl	35%[b]
7	R^3 = Me	60%[c]
8	R^2 = R^3 = Me (Thr)	60%[c]
9	Ser in place of AOSer	N.D.[d]

Notes:
[a] Conversion was analyzed by UPLC-MS;
[b] Overnight reaction time required;
[c] AOL reaction crude applied;
[d] No desired pdt detected.

Scheme S8. Scheme of the CEL optimization.

AOSer-containing peptide **3** (0.5 mg, 200 nmol, 1.0 eq.) was first dissolved in solutions designed above at 20 mM concentration, together with ketoacids (5.0 eq., 100 mM). The reaction mixture was incubated in water bath under 60°C for 3 hr. Reaction progress was then monitored by UPLC-MS.

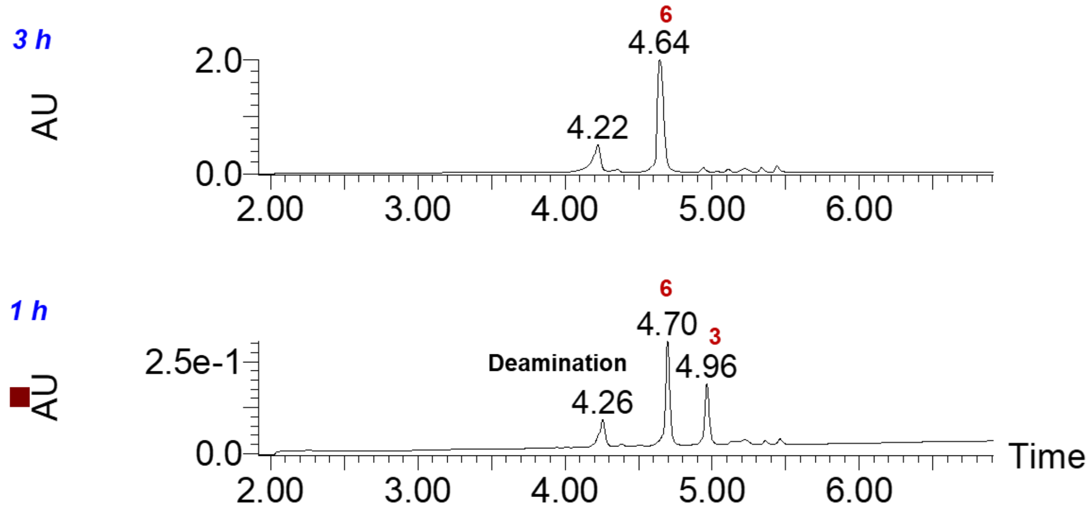

Figure S51. Reaction monitoring by UPLC-MS of CEL using substrate **3** under **entry 1** conditions (90% DMSO/0.01 M Oxalic acid) for 3 hr.

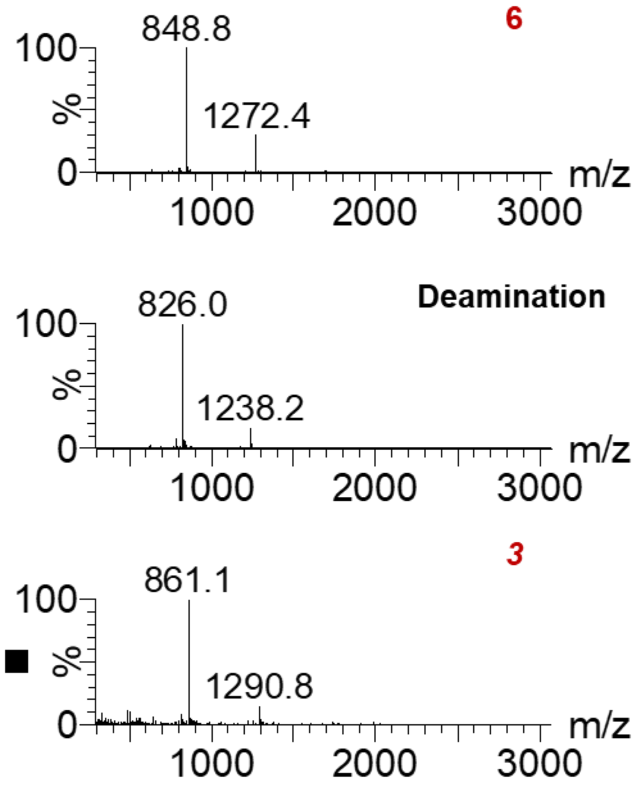

Figure S52. ESI-MS spectra of AOSer peptide **3** (bottom). ESI-MS calcd. for C109H166N32O41 $[M+2H]^{2+}$ m/z = 1291.3, found 1290.8; $[M+3H]^{3+}$ m/z = 861.2, found 861.1. ESI-MS spectra of deamination side product (middle). ESI-MS calcd. for C102H159N31O41 $[M+2H]^{2+}$ m/z = 1238.8, found 1238.6;

$[M+3H]^{3+}$ m/z = 826.2, found 826.1; ESI-MS spectra of ester peptide **6** (top). ESI-MS calcd. for C106H163N31O42 $[M+2H]^{2+}$ m/z = 1272.8, found 1272.4; $[M+3H]^{3+}$ m/z = 848.9, found 848.8.

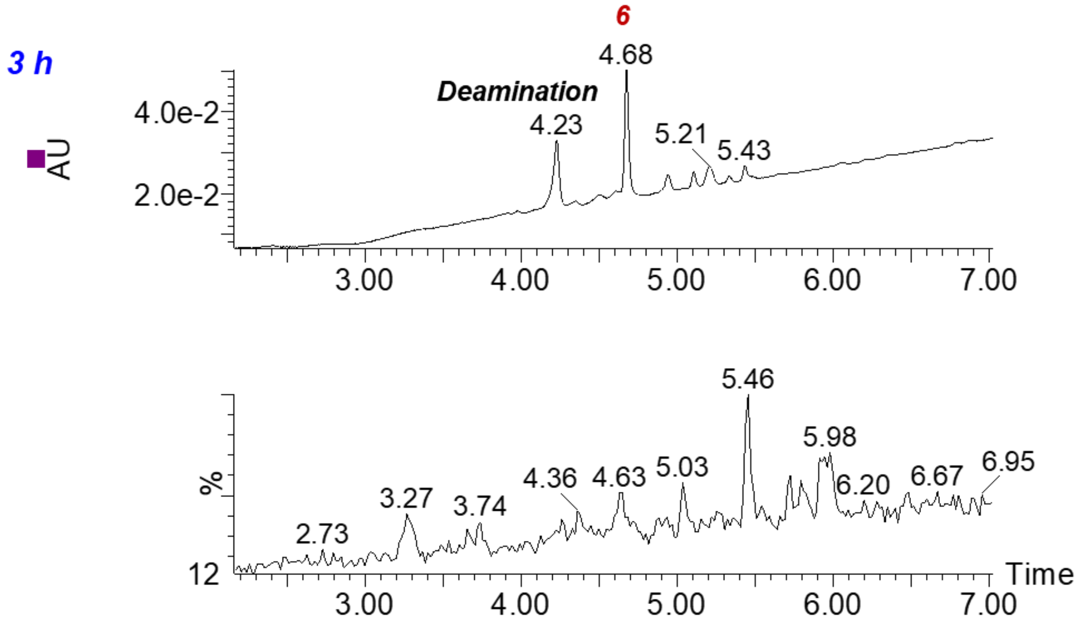

Figure S53. Reaction monitoring by UPLC-MS of CEL using substrate **3** under **entry 2** conditions (ACN/H2O, 3/1, v, with 0.01 M Oxalic acid) for 3 hr.

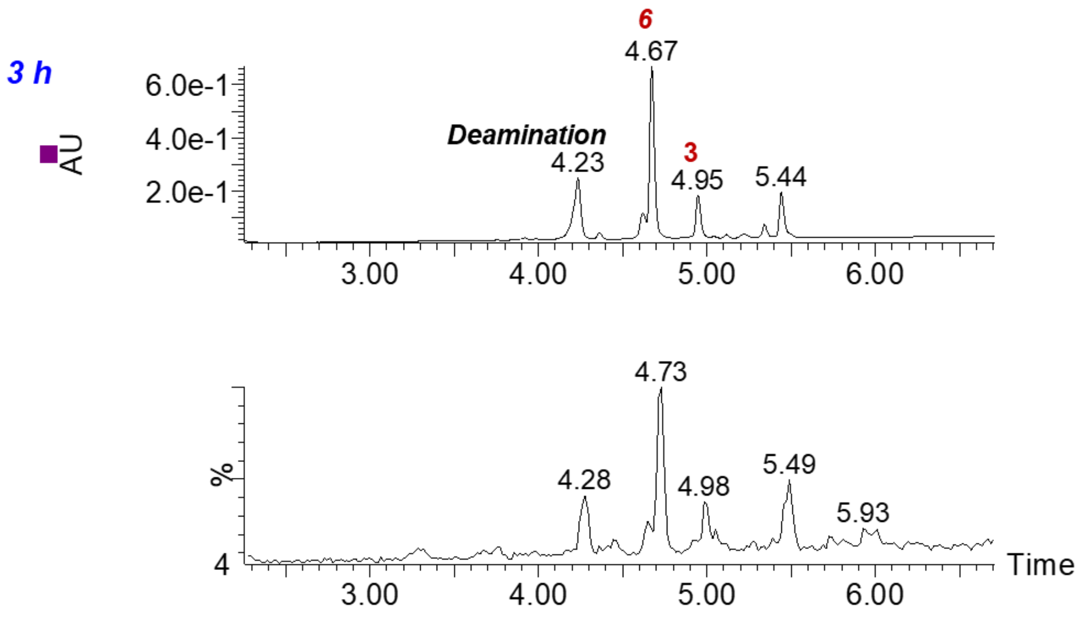

Figure S54. Reaction monitoring by UPLC-MS of CEL using substrate **3** under **entry 3** conditions (2.5% TFA, 90% DMSO/H2O) for 3 hr.

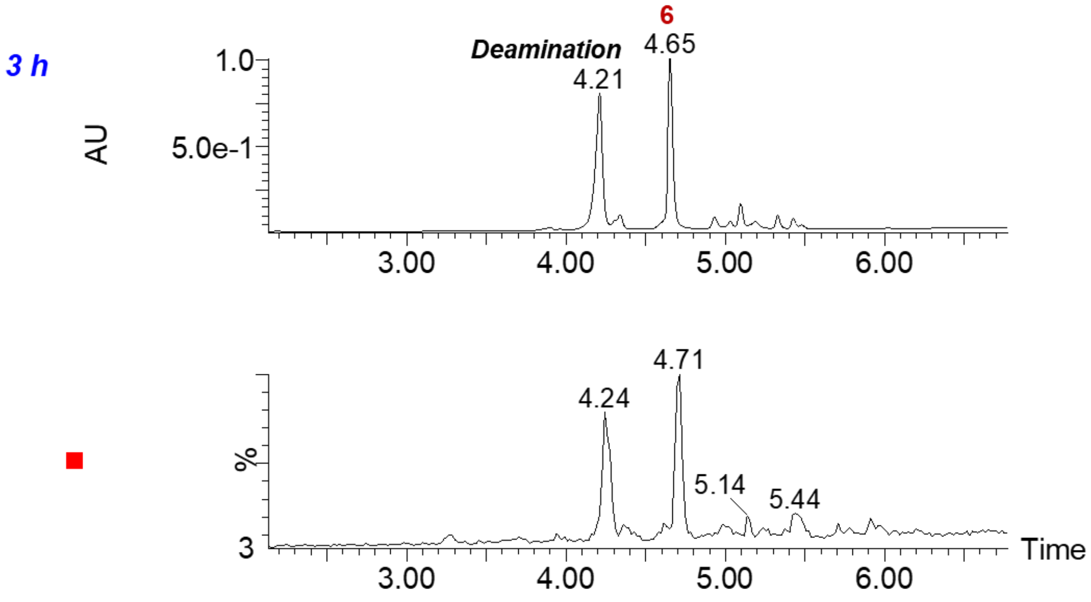

Figure S55. Reaction monitoring by UPLC-MS of CEL using substrate **3** under DMF containing 0.01 M Oxalic acid for 3 hr.

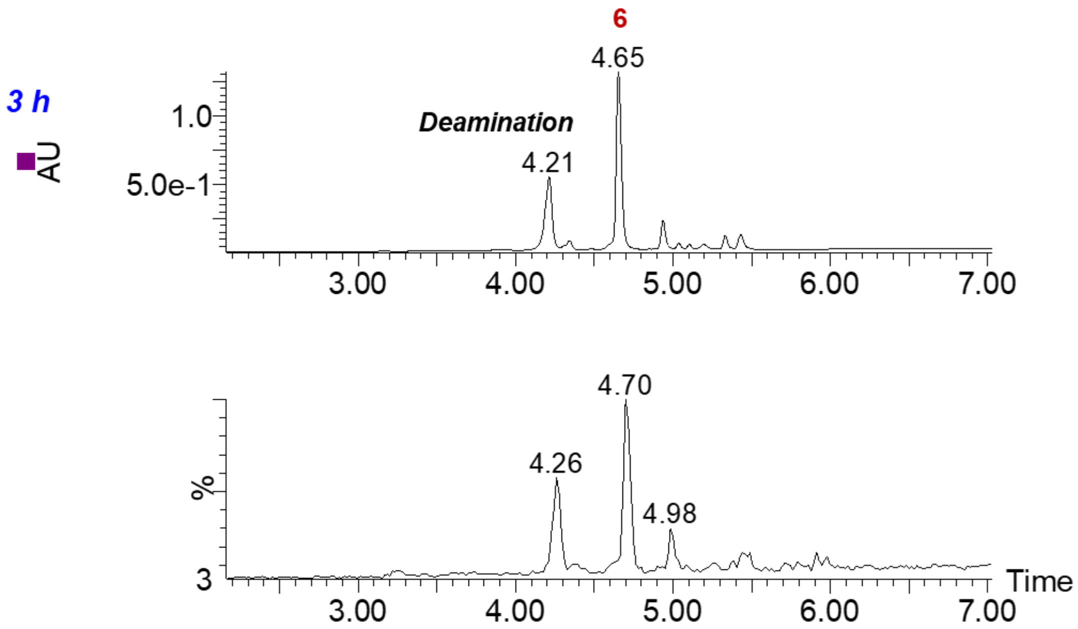

Figure S56. Reaction monitoring by UPLC-MS of CEL using substrate **3** under NMP containing 0.01 M Oxalic acid for 3 hr.

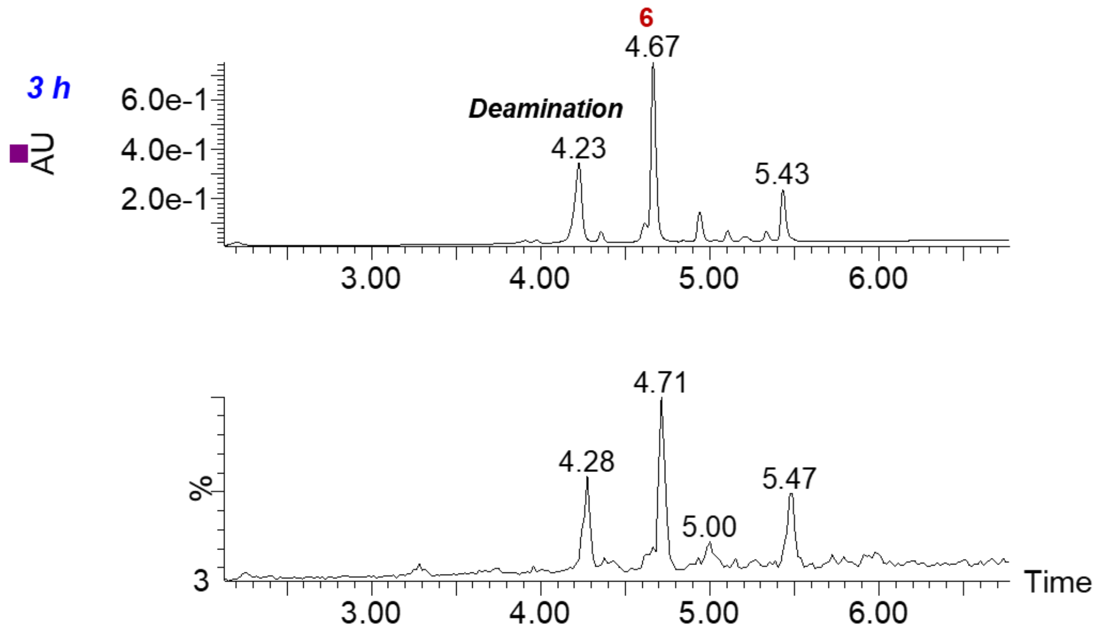

Figure S57. Reaction monitoring by UPLC-MS of CEL using substrate **3** under 50%DMSO/aqueous 0.01 M oxalic acid for 3 hr.

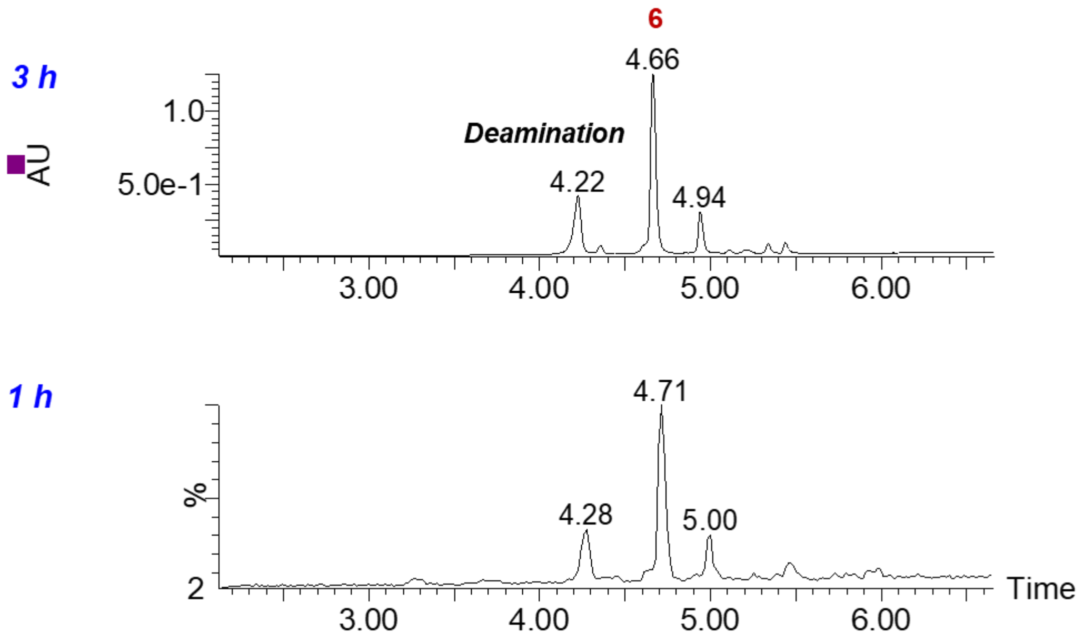

Figure S58. Reaction monitoring by UPLC-MS of CEL using substrate **3** under DMSO/IPA (8/2, v) containing 0.01 M oxalic acid for 3 hr.

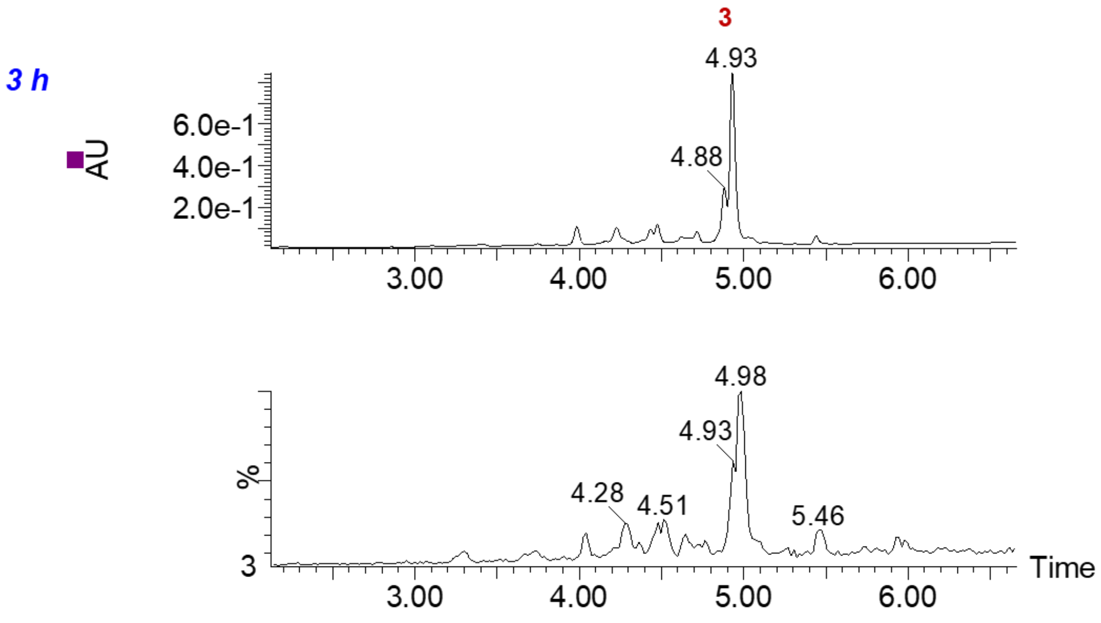

Figure S59. Reaction monitoring by UPLC-MS of CEL using substrate **3** under 6 M Gdm·HCl solution containing 0.01 M oxalic acid (pH = 2) for 3 hr.

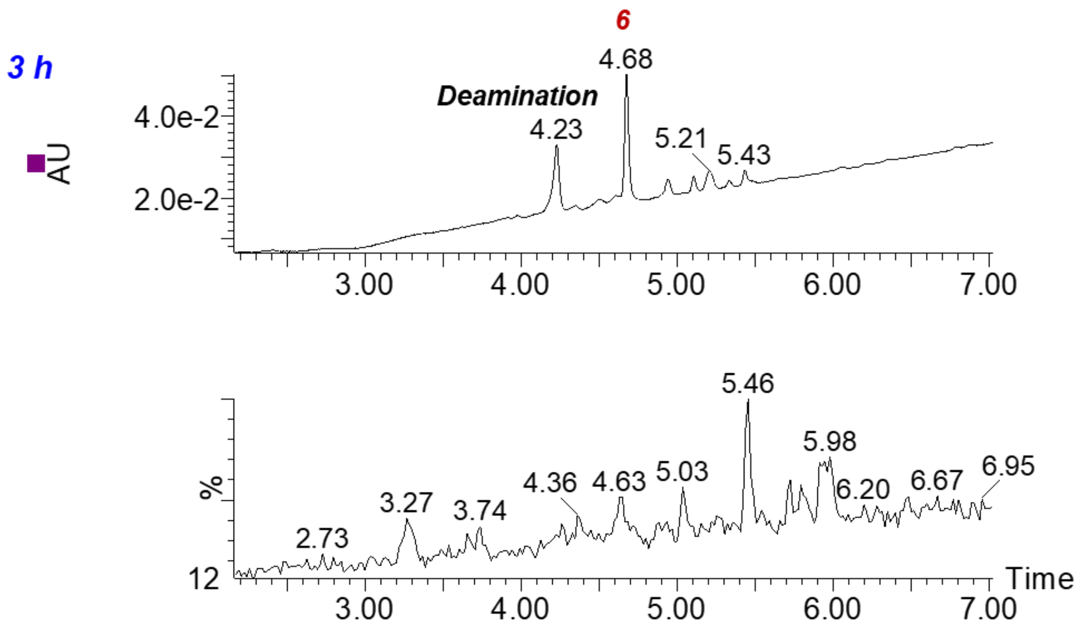

Figure S60. Reaction monitoring by UPLC-MS of CEL using substrate **3** under 50%DMSO/aqueous 0.01 M oxalic acid for 3 hr.

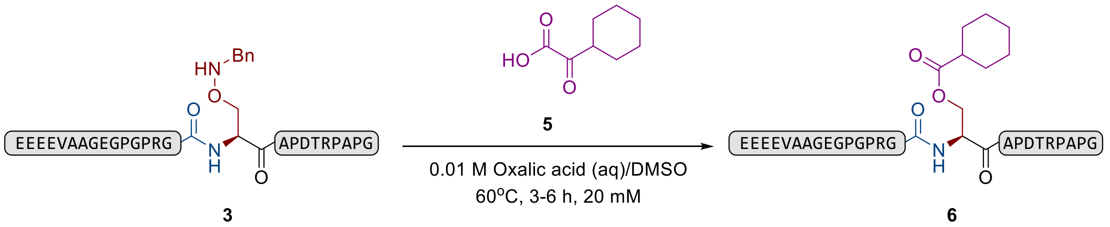

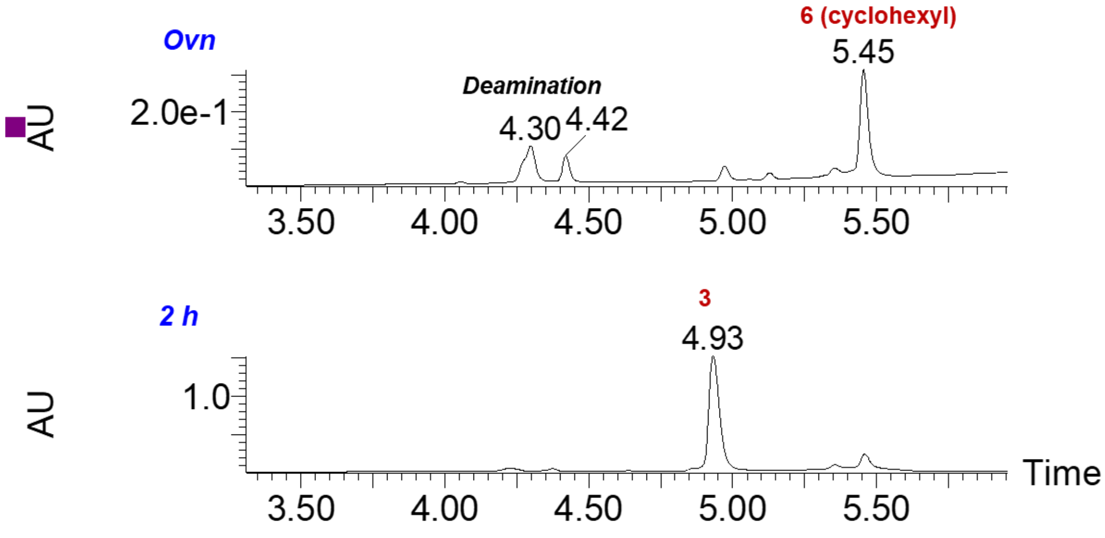

Figure S61. Reaction monitoring by UPLC-MS of CEL using substrate **3**

under **entry 5** (cyclohexyl) conditions overnight.

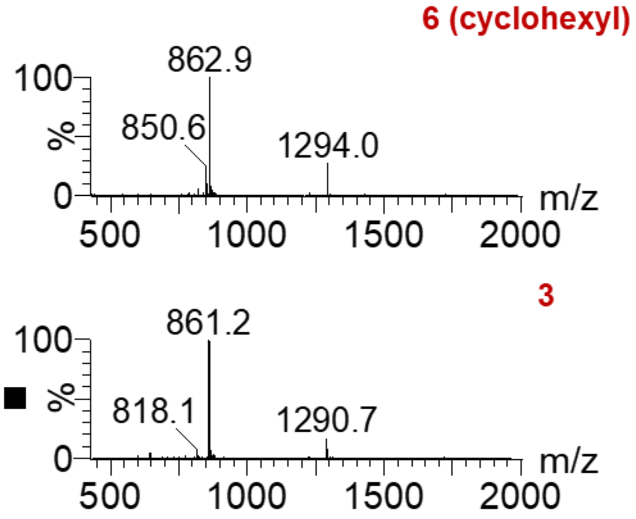

Figure S62. ESI-MS spectra of AOSer peptide **3** (bottom). ESI-MS calcd. for C109H166N32O41 $[M+2H]^{2+}$ m/z = 1291.3, found 1290.7; $[M+3H]^{3+}$ m/z = 861.2, found 861.2; ESI-MS spectra of ester peptide **6 (cyclohexyl)** (top). ESI-MS calcd. for C109H169N31O42 $[M+2H]^{2+}$ m/z = 1293.9, found 1294.0; $[M+3H]^{3+}$ m/z = 862.9, found 862.9.

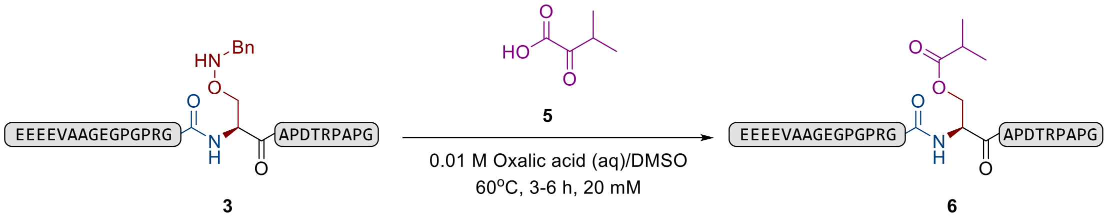

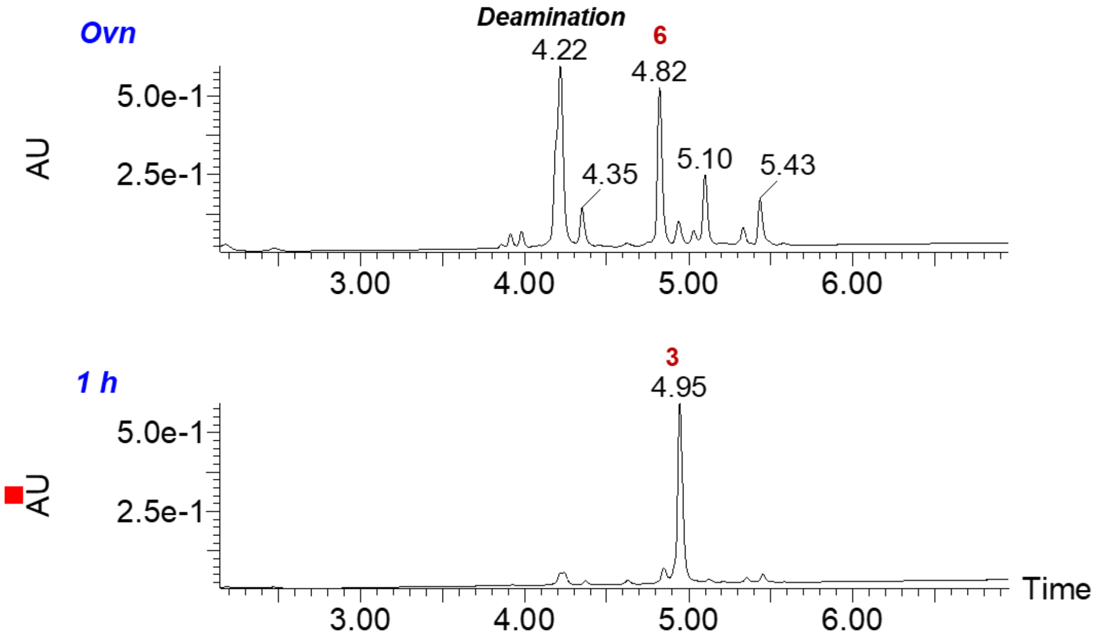

Figure S63. Reaction monitoring by UPLC-MS of CEL using substrate **3** under **entry 6** (isopropyl) conditions overnight.

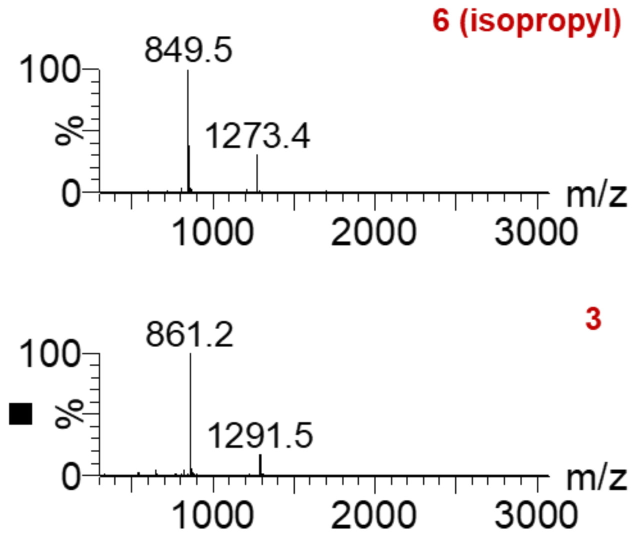

Figure S64. ESI-MS spectra of AOSer peptide **3** (bottom). ESI-MS calcd. for C109H166N32O41 $[M+2H]^{2+}$ m/z = 1291.3, found 1290.5; $[M+3H]^{3+}$ m/z = 861.2, found 861.2; ESI-MS spectra of ester peptide **6 (isopropyl)** (top). ESI-MS calcd. for C106H165N31O42 $[M+2H]^{2+}$ m/z = 1273.8, found 1273.4; $[M+3H]^{3+}$ m/z = 849.6, found 849.5.

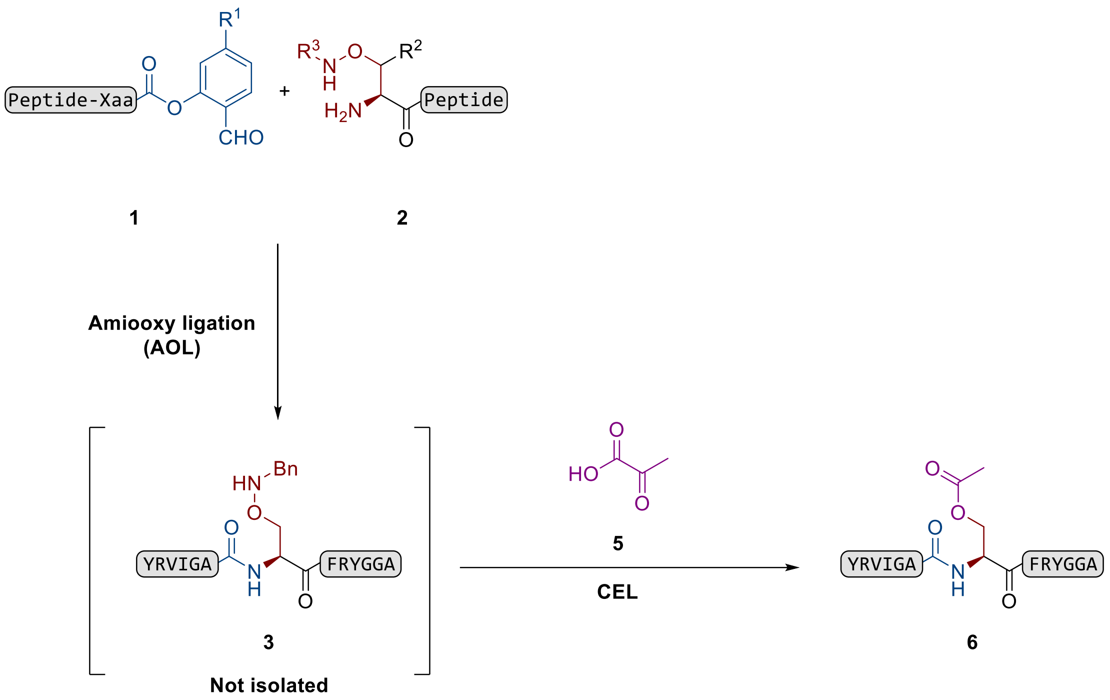

Scheme S9. Scheme of one-pot AOL-CEL for AO(Bn)Ser peptide.

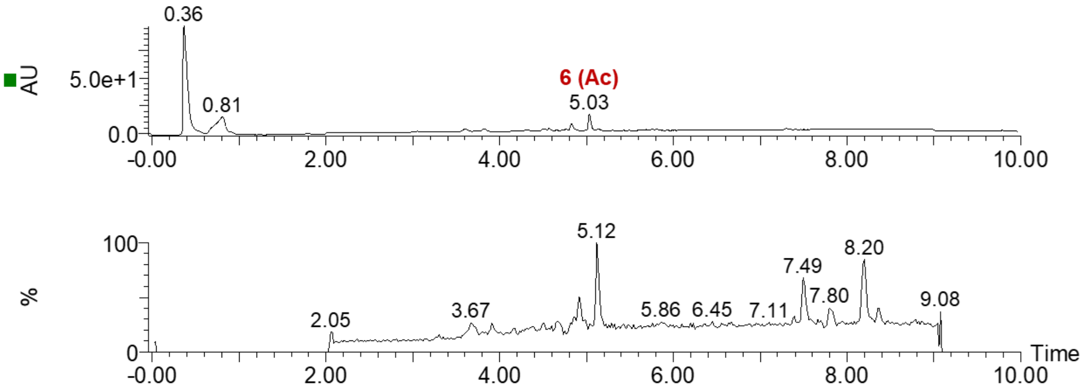

Figure S65. Reaction monitoring by UPLC-MS of one-pot AOL-CEL using substrate **3** conditions overnight.

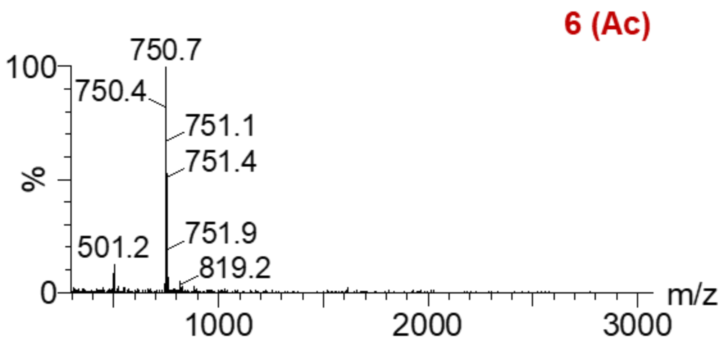

Figure S66. ESI-MS spectra of ester peptide **6 (Ac)** (top). ESI-MS calcd. for C69H102N20O18 $[M+2H]^{2+}$ m/z = 750.8, found 750.7; $[M+3H]^{3+}$ m/z = 500.9, found 501.2.

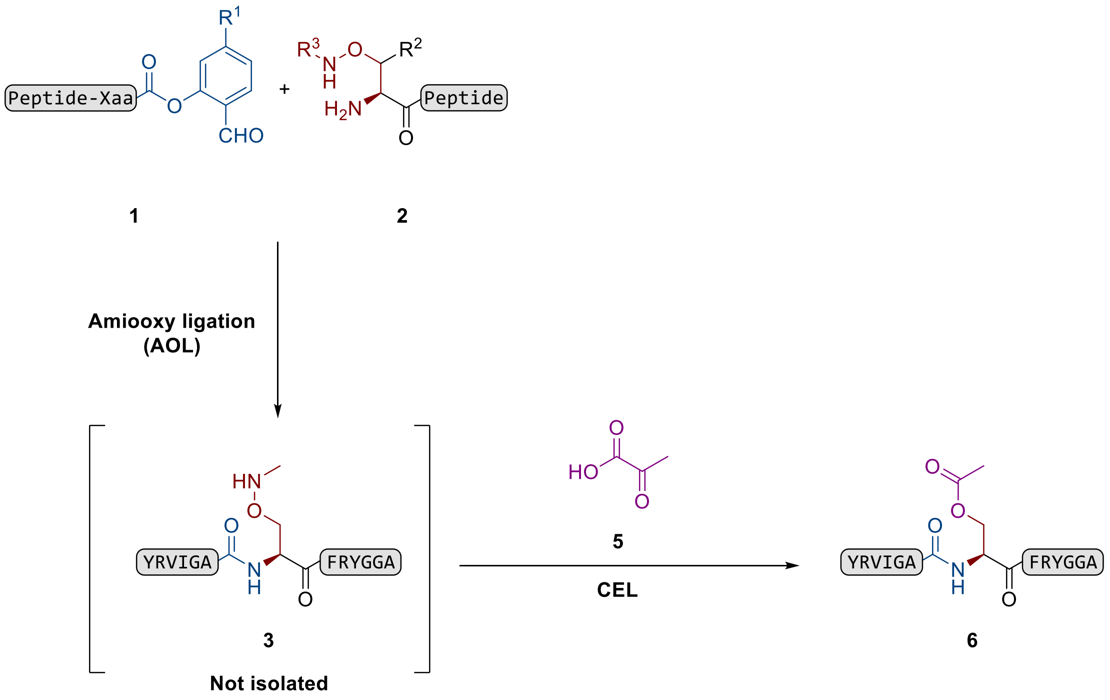

Scheme S10. Scheme of one-pot AOL-CEL for AO(Me)Ser peptide.

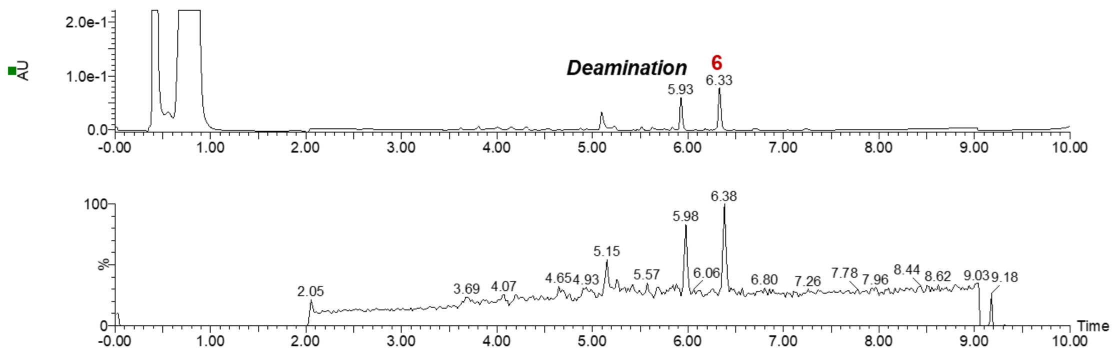

Figure S67. Reaction monitoring by UPLC-MS of one-pot AOL-CEL using substrate **3** under entry 7 conditions above overnight.

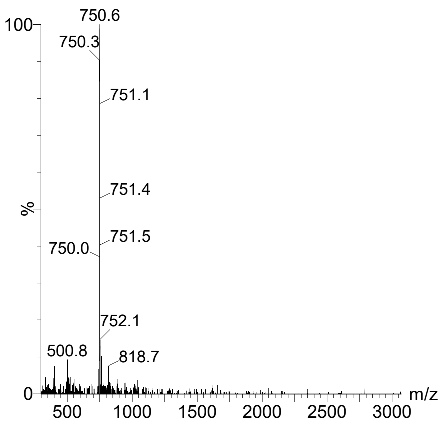

Figure S68. ESI-MS spectra of ester peptide **6 (Ac)** (top). ESI-MS calcd. for C69H102N20O18 $[M+2H]^{2+}$ m/z = 750.8, found 750.6; $[M+3H]^{3+}$ m/z = 500.9, found 500.8.

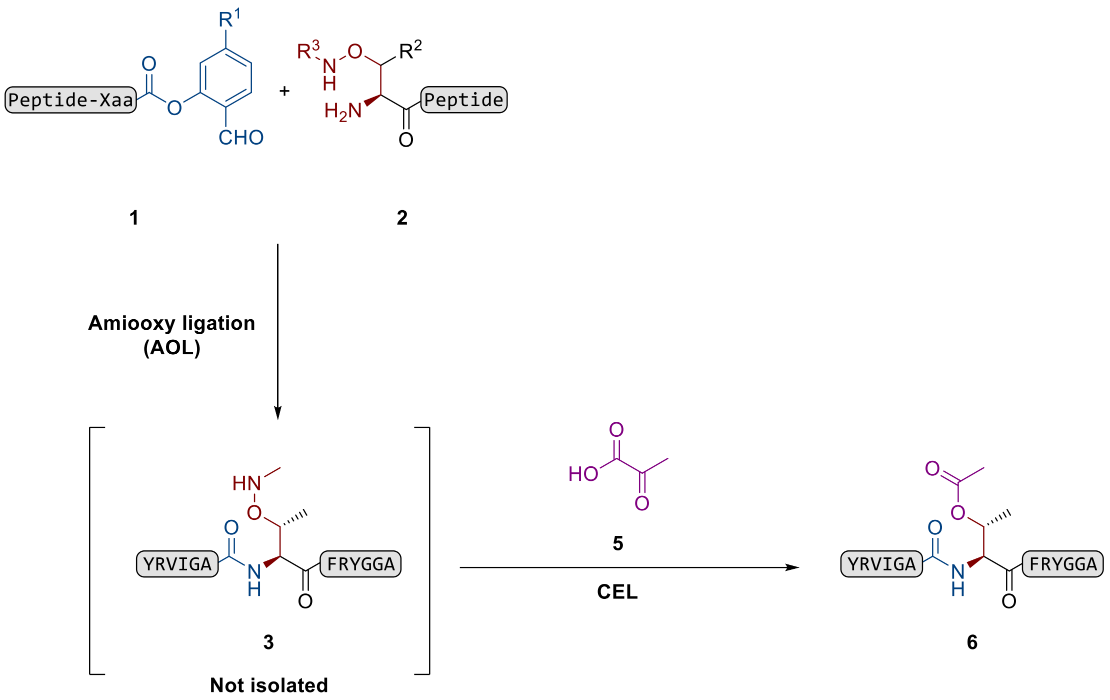

Scheme S11. Scheme of one-pot AOL-CEL for AO(Me)Thr peptide.

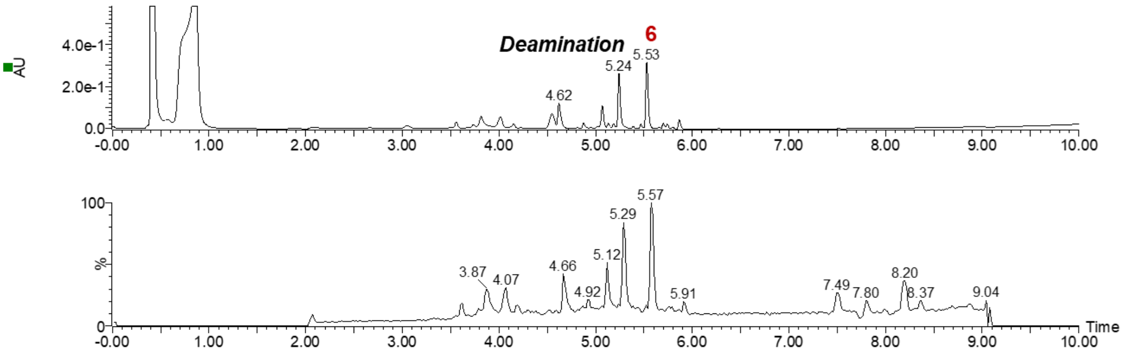

Figure S69. Reaction monitoring by UPLC-MS of one-pot AOL-CEL using substrate **3** under entry 7 conditions above overnight.

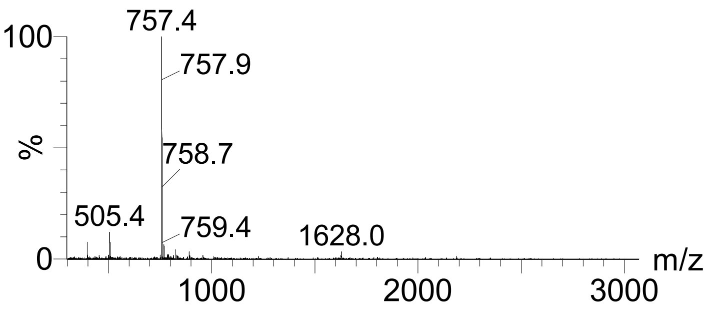

Figure S70. ESI-MS spectra of ester peptide **6 (Ac, Thr)** (top). ESI-MS calcd. for C70H104N20O18 $[M+2H]^{2+}$ m/z = 757.9, found 757.4; $[M+3H]^{3+}$ m/z = 505.6, found 505.4.

4.3. Confirmation of ester linkage in CEL

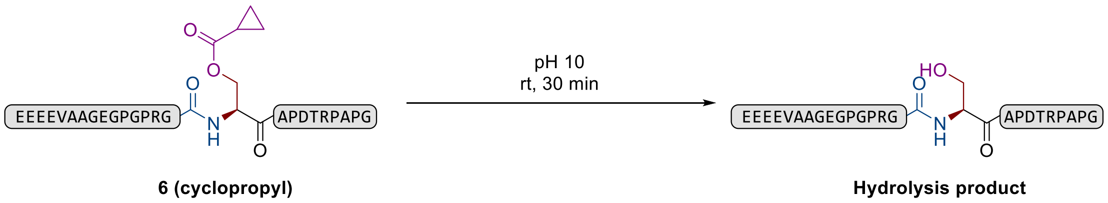

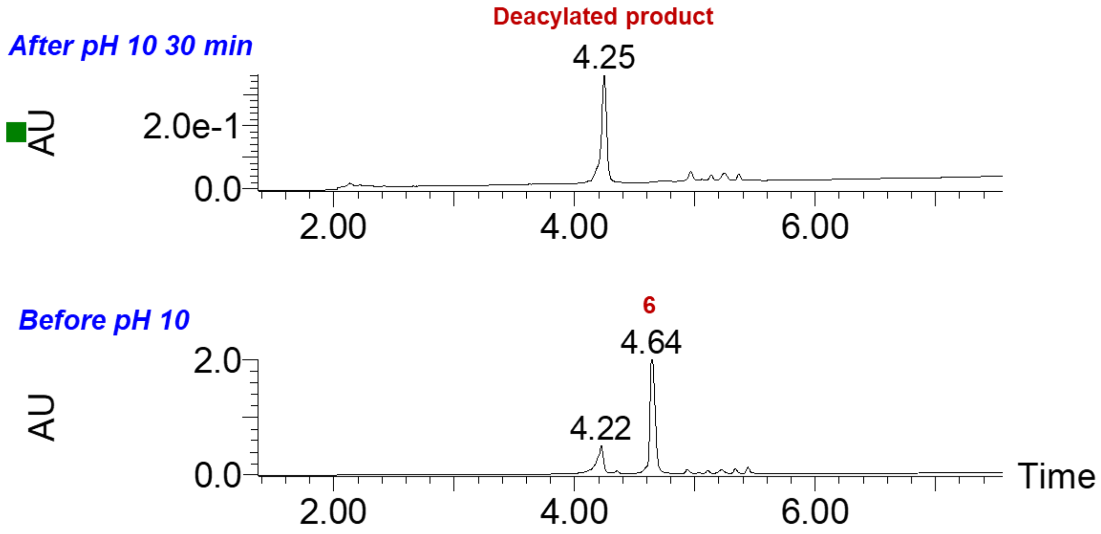

Figure S71. UPLC monitoring of peptide **6** hydrolysis. Confirmation of ester linkage of **6** by adjusting pH to 10 for 30 min.

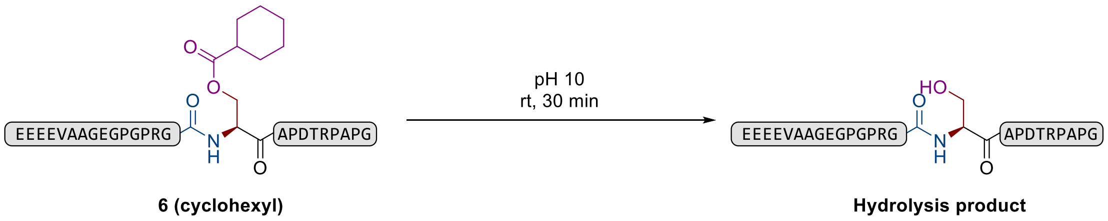

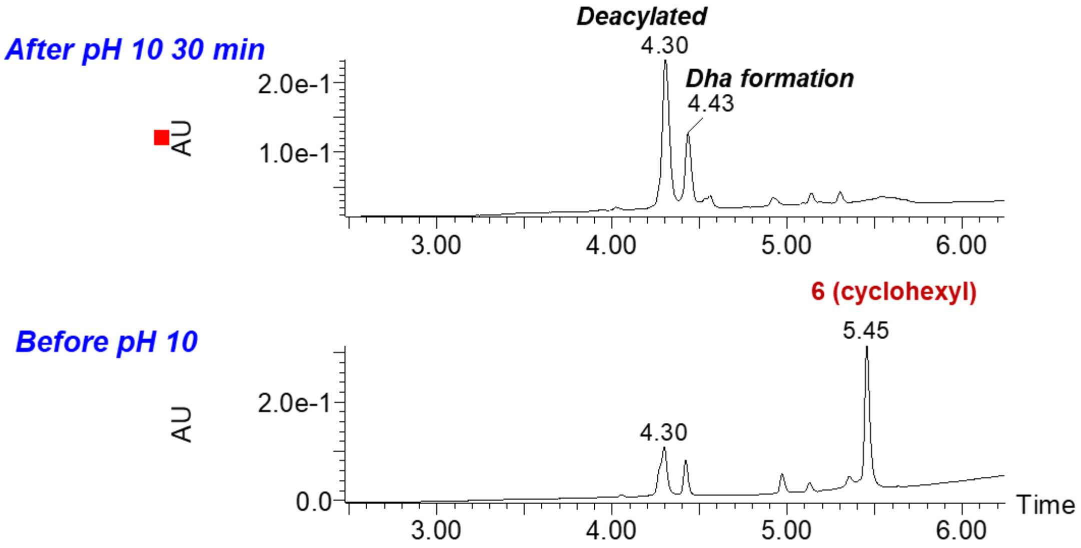

Figure S72. UPLC monitoring of peptide **6 (cyclohexyl)** hydrolysis. Confirmation of ester linkage of **6** by adjusting pH to 10 for 30 min.

5. Scope and limitations of AOL and CEL

5.1. Aminooxy ligation of model peptides

5.1.1. Synthesis of model N-terminal AO(Bn)/AO(Me) peptides

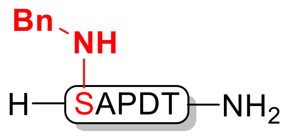

S18

H-(AO,Bn)APDT-NH_2 **S18** was synthesized according to the standard protocol of SPPS at 0.05 mmol scale. The crude peptide was purified by preparative reverse-phase HPLC (5-30% CH_3CN/H_2O over 45 min) and lyophilized to afford the desired peptide **S18** (29.0 mg, 97.6% yield).

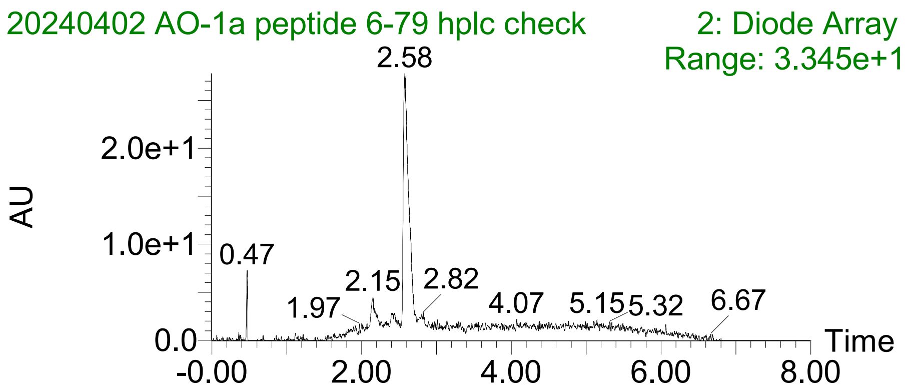

Figure S73. UV (190-400 nm) trace from UPLC-MS analysis of purified H-(AO,Bn)APDT-NH_2 **S18** gradient 5-95% CH_3CN/H_2O containing 0.1% TFA over 8 min at a flow rate of 0.4 mL/min.

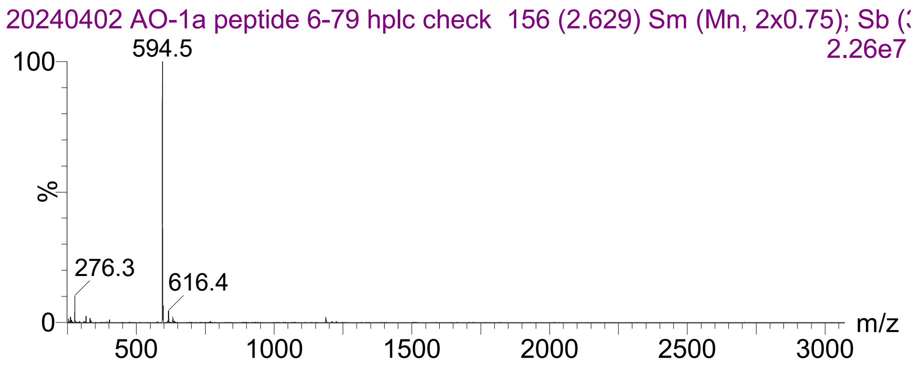

Figure S74. ESI-MS calcd. for $C_{26}H_{39}N_7O_9$ $[M+H]^+$ m/z = 594.6, found 594.5.

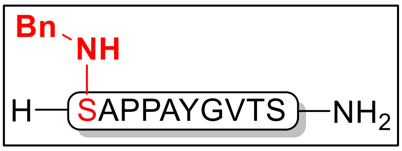

S19

H-(AO,Bn)APPAYGVTS -NH_2 **S19** was synthesized according to the standard protocol of SPPS at 0.05 mmol scale. The crude peptide was purified by preparative reverse-phase HPLC (10-40% CH_3CN/H_2O over 45 min) and lyophilized to afford the desired peptide **S19** (28.4 mg, 53.9% yield).

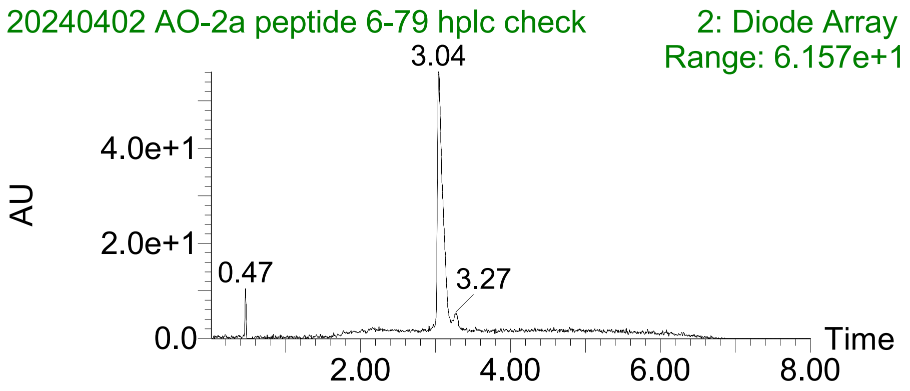

Figure S75. UV (190-400 nm) trace from UPLC-MS analysis of purified H-(AO,Bn)APPAYGVTS -NH_2 gradient 5-95% CH_3CN/H_2O containing 0.1% TFA over 8 min at a flow rate of 0.4 mL/min.

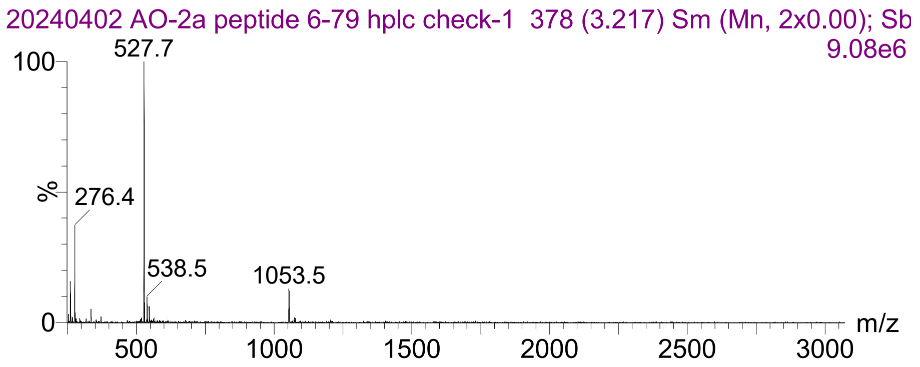

Figure S76. ESI-MS calcd. for $C_{49}H_{72}N_{12}O_{14}$ $[M+H]^+$ m/z = 1054.2, found 1053.5; $[M+2H]^{2+}$ m/z = 527.6, found 527.7.

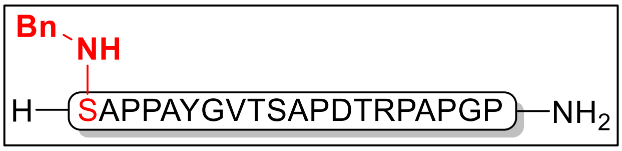

S20

H-(AO,Bn)APPAYGVTSAPDTRPAPGP-NH_2 **S20** was synthesized according to the standard protocol of SPPS at 0.05 mmol scale. The crude peptide was purified by preparative reverse-phase HPLC (10-40% CH_3CN/H_2O over 45 min) and lyophilized to afford the desired peptide **S20** (30.0 mg, 29.8% yield).

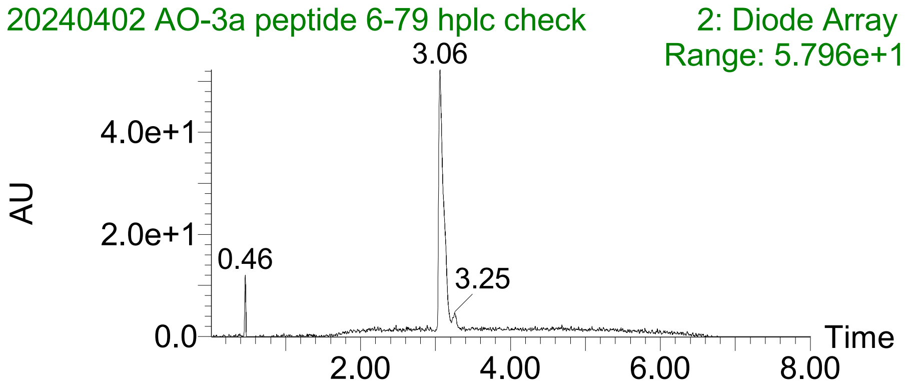

Figure S77. UV (190-400 nm) trace from UPLC-MS analysis of purified H-(AO,Bn)APPAYGVTSAPDTRPAPGP-NH_2 **S20** gradient 5-95% CH_3CN/H_2O containing 0.1% TFA over 8 min at a flow rate of 0.4 mL/min.

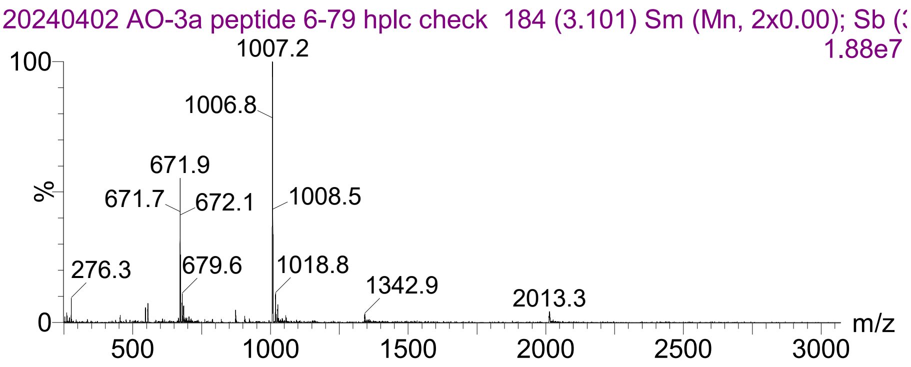

Figure S78. ESI-MS calcd. for $C_{91}H_{137}N_{25}O_{27}$ $[M+H]^+$ m/z = 2014.3, found 2013.3; $[M+2H]^{2+}$ m/z = 1007.7, found 1007.2; $[M+3H]^{3+}$ m/z = 672.1, found 671.9.

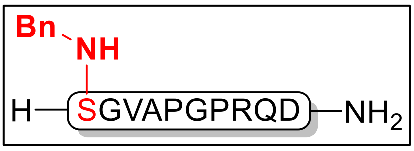

S21

H-(AO,Bn)GVAPGPRQD-NH_2 **S21** was synthesized according to the standard protocol of SPPS at 0.05 mmol scale. The crude peptide was purified by preparative reverse-phase HPLC (10-40% CH_3CN/H_2O over 45 min) and lyophilized to afford the desired peptide **S21** (29.5 mg, 54.3% yield).

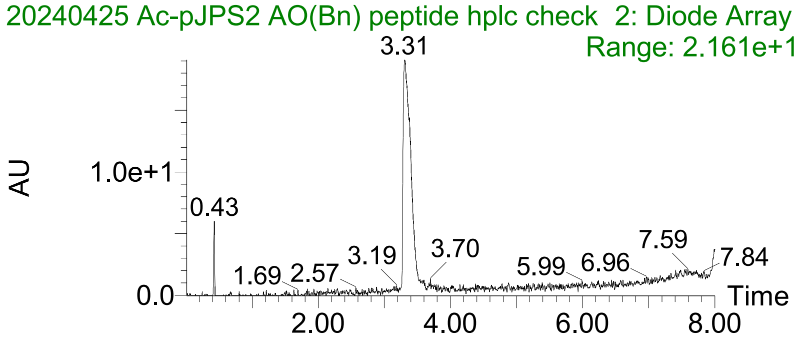

Figure S79. UV (190-400 nm) trace from UPLC-MS analysis of purified H-(AO,Bn)GVAPGPRQD-NH_2 **S21** gradient 5-95% CH_3CN/H_2O containing

0.1% TFA over 8 min at a flow rate of 0.4 mL/min.

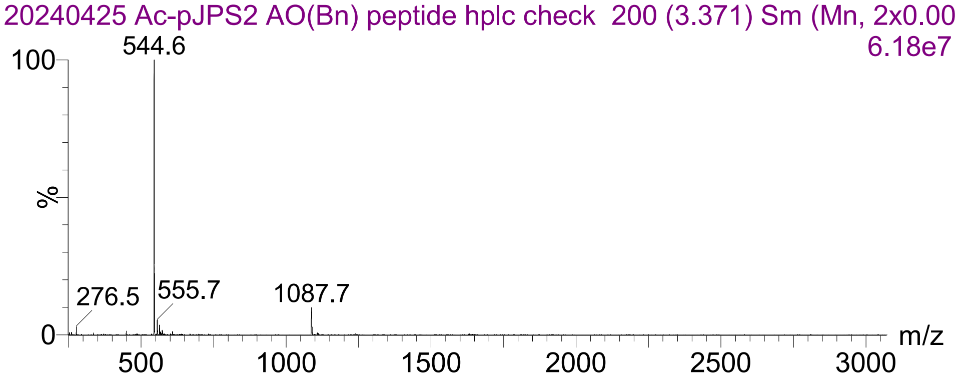

Figure S80. ESI-MS calcd. for $C_{91}H_{137}N_{25}O_{27}$ $[M+H]^{+}$ m/z = 1088.2, found 1087.7; $[M+2H]^{2+}$ m/z = 544.6, found 544.6.

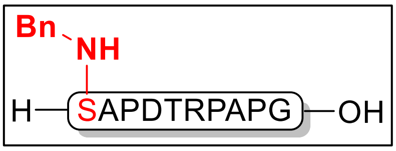

S22

H-(AO,Bn)APDTRPAPG-OH **S22** was synthesized according to the standard protocol of SPPS at 0.05 mmol scale. The crude peptide was purified by preparative reverse-phase HPLC (5-30% CH_3CN/H_2O over 45 min) and lyophilized to afford the desired peptide **S22** (35.5 mg, 65.3% yield).

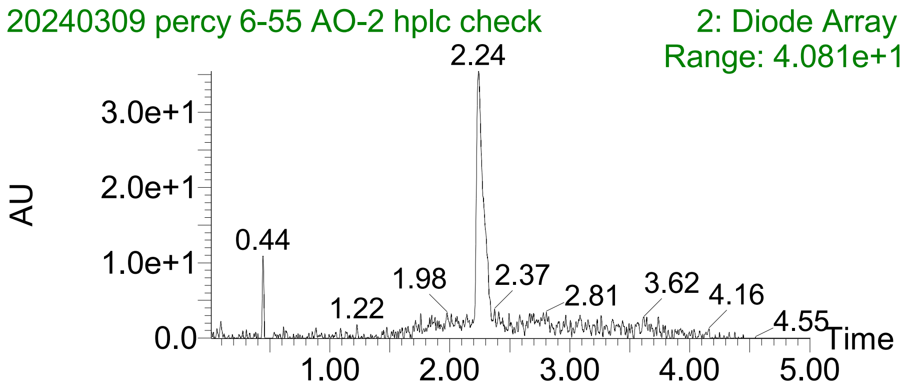

Figure S81. UV (190-400 nm) trace from UPLC-MS analysis of purified H-

(AO,Bn)APDTRPAPG-NH_2 **S22** gradient 5-95% CH_3CN/H_2O containing 0.1% TFA over 5 min at a flow rate of 0.4 mL/min.

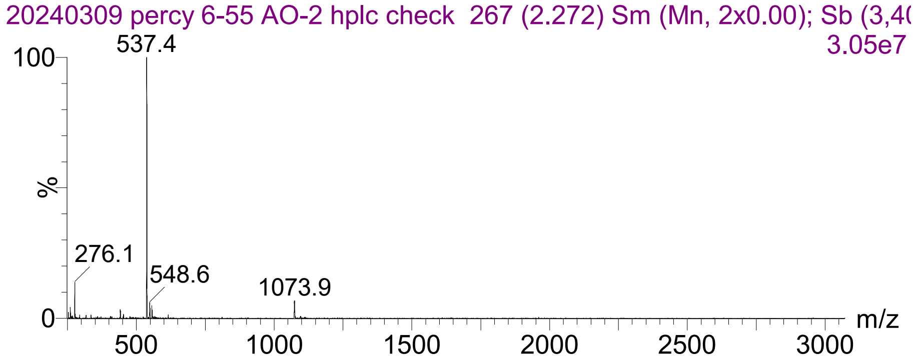

Figure S82. ESI-MS calcd. for $C_{47}H_{72}N_{14}O_{15}$ $[M+H]^+$ m/z = 1074.2, found 1073.9; $[M+2H]^{2+}$ m/z = 537.6, found 537.4.

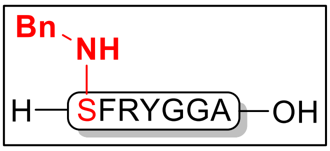

S23

H-(AO,Bn)FRYGGA-OH **S23** was synthesized according to the standard protocol of SPPS at 0.05 mmol scale. The crude peptide was purified by preparative reverse-phase HPLC (10-40% CH_3CN/H_2O over 45 min) and lyophilized to afford the desired peptide **S23** (27.5 mg, 63.9% yield).

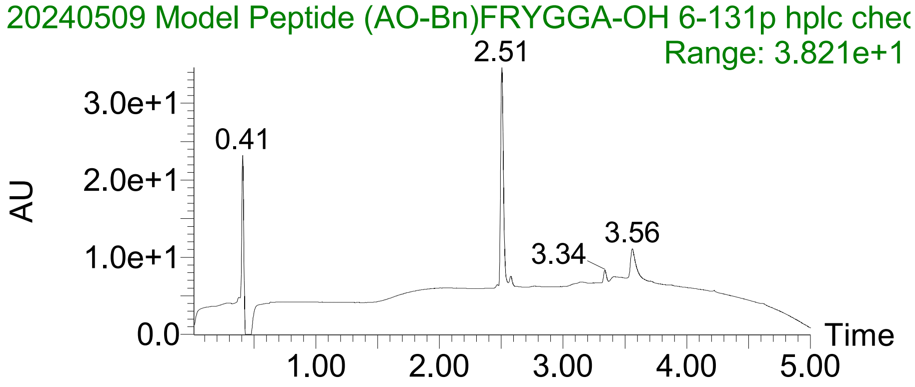

Figure S83. UV (190-400 nm) trace from UPLC-MS analysis of purified H-

(AO,Bn)FRYGGA-OH **S23** gradient 5-95% CH_3CN/H_2O containing 0.1% TFA over 5 min at a flow rate of 0.4 mL/min.

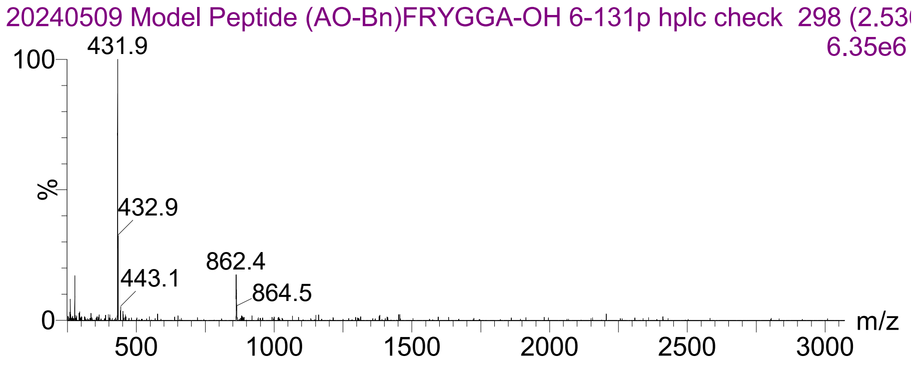

Figure S84. ESI-MS calcd. for $C_{54}H_{78}N_{14}O_{18}$ $[M+H]^+$ m/z = 862.0, found 862.4; $[M+2H]^{2+}$ m/z = 431.5, found 431.9.

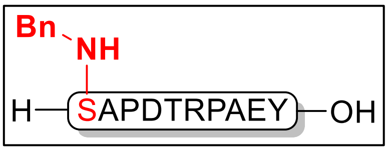

S24

H-(AO,Bn)APDTRPAEY-OH **S24** was synthesized according to the standard protocol of SPPS at 0.05 mmol scale. The crude peptide was purified by preparative reverse-phase HPLC (10-40% CH_3CN/H_2O over 45 min) and lyophilized to afford the desired peptide **S24** (38.3 mg, 63.2% yield).

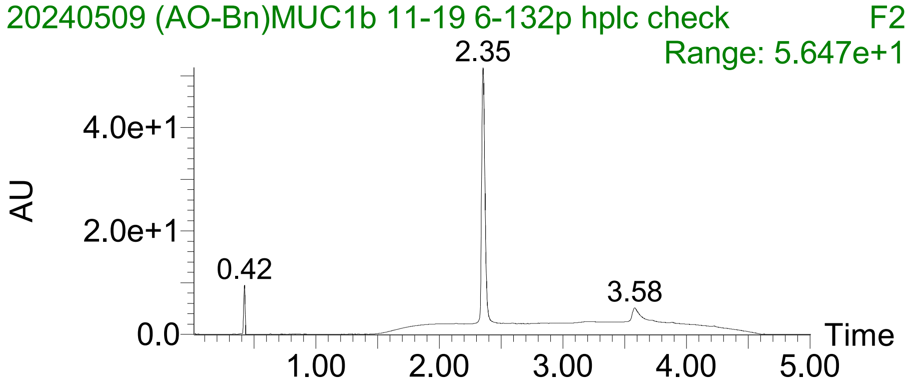

Figure S85. UV (190-400 nm) trace from UPLC-MS analysis of purified H-

(AO,Bn)APDTRPAEY-OH **S24** gradient 5-95% CH_3CN/H_2O containing 0.1% TFA over 5 min at a flow rate of 0.4 mL/min.

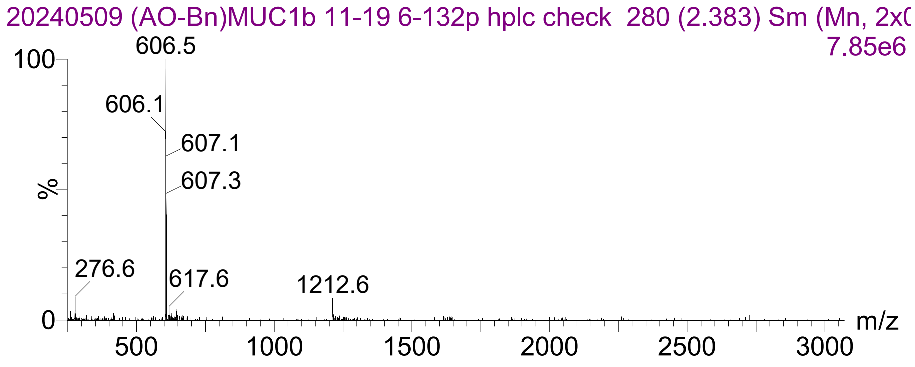

Figure S86. ESI-MS calcd. for $C_{54}H_{78}N_{14}O_{18}$ $[M+H]^+$ m/z = 1212.3, found 1212.6; $[M+2H]^{2+}$ m/z = 606.7, found 606.5.

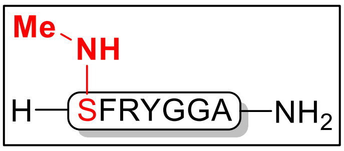

S15

H-(AO,Me)FRYGGA-NH_2 **S15** was synthesized according to the standard protocol of SPPS at 0.05 mmol scale. The crude peptide was purified by preparative reverse-phase HPLC (5-30% CH_3CN/H_2O over 45 min) and lyophilized to afford the desired peptide **S15** (17.8 mg, 45.3% yield).

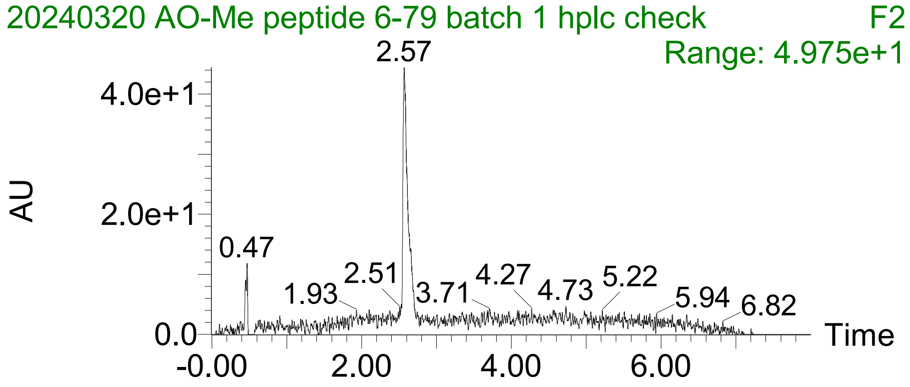

Figure S87. UV (190-400 nm) trace from UPLC-MS analysis of purified H-

(AO,Me)FRYGGA-NH_2 **S15** gradient 5-95% CH_3CN/H_2O containing 0.1% TFA over 8 min at a flow rate of 0.4 mL/min.

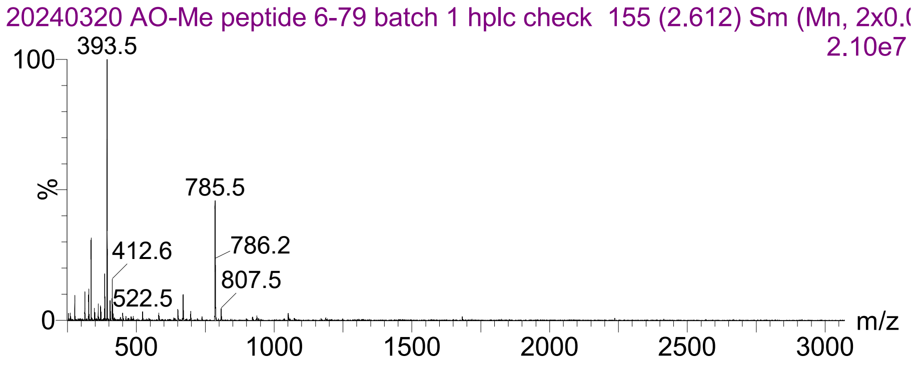

Figure S88. ESI-MS calcd. for $C_{35}H_{51}N_{11}O_{10}$ $[M+H]^+$ m/z = 786.9, found 785.5; $[M+2H]^{2+}$ m/z = 393.9, found 393.5.

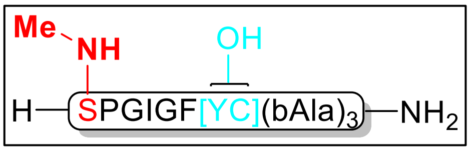

S25

H-(AO,Me)PGIGF[YC](β-Ala)$_3$-NH_2 **S25** was synthesized according to the standard protocol of SPPS at 0.05 mmol scale, where [YC] represents the NTD structure. The crude peptide was purified by preparative reverse-phase HPLC (15-45% CH_3CN/H_2O over 45 min) and lyophilized to afford the desired peptide **S25** (29.7 mg, 48.8% yield).

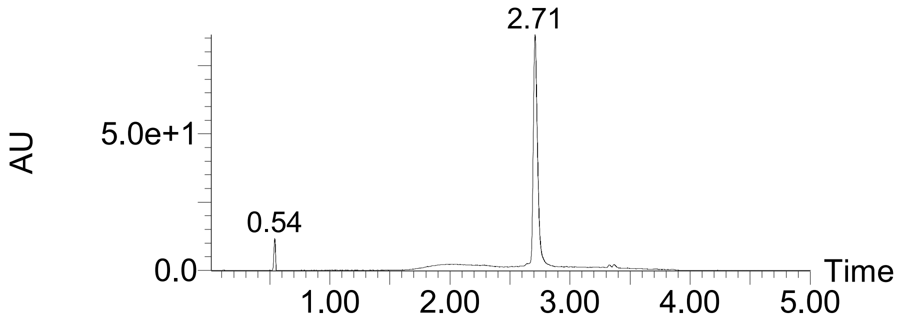

Figure S89. UV (190-400 nm) trace from UPLC-MS analysis of purified H-

(AO,Me)PGIGF[YC](β-Ala)$_3$-NH$_2$ **S25** gradient 5-95% CH_3CN/H_2O containing 0.1% TFA over 5 min at a flow rate of 0.4 mL/min.

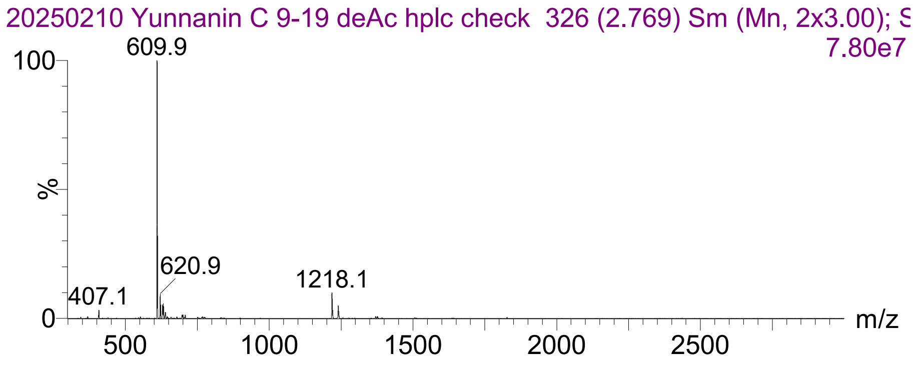

Figure S90. ESI-MS calcd. for $C_{70}H_{102}N_{18}O_{29}$ $[M+H]^+$ m/z = 1219.4, found 1218.1; $[M+2H]^{2+}$ m/z = 610.2, found 609.9; $[M+3H]^{3+}$ m/z = 407.1, found 407.1.

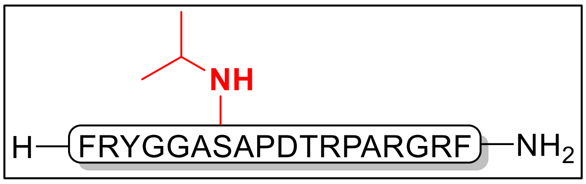

S26

H-FRYGGA(AO,iPr)APDTRPARGF-NH$_2$ **S26** was synthesized according to the standard protocol of SPPS at 0.05 mmol scale. The crude peptide was purified by preparative reverse-phase HPLC (5-30% CH_3CN/H_2O over 45 min) and lyophilized to afford the desired peptide **S26** (17.8 mg, 45.3% yield).

5.1.2. Synthesis of model C-terminus peptide SAL esters

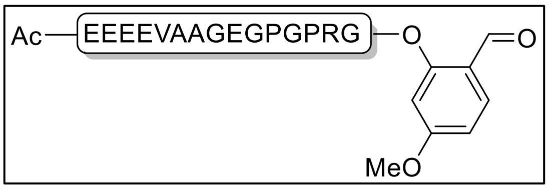

S27

Ac-EEEEVAAGEGPGPRG-CO-SAL **S27** was synthesized according to the general synthesis of C-terminal Peptide SAL esters using n+1 strategy at 0.031 mmol scale. The crude peptide was purified by preparative reverse-phase HPLC (5-30% CH_3CN/H_2O over 45 min) and lyophilized to afford the desired peptide **S27** (25.3 mg, 48.9% yield).

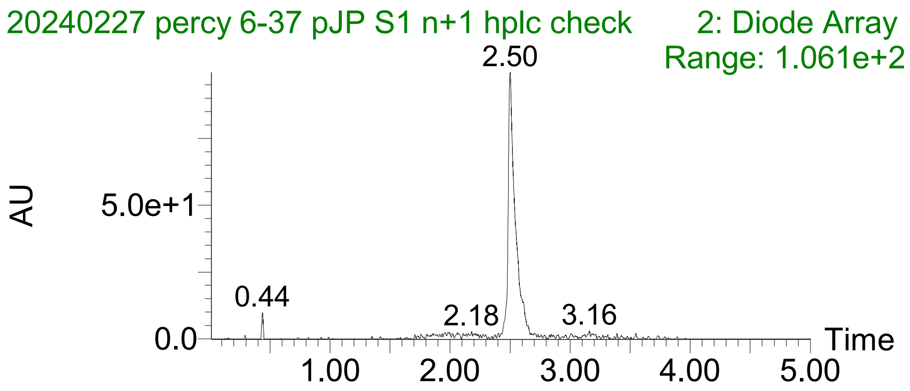

Figure S91. UV (190-400 nm) trace from UPLC-MS analysis of purified Ac-EEEEVAAGEGPGPRG-CO-SAL **S27** gradient 5-95% CH_3CN/H_2O containing 0.1% TFA over 5 min at a flow rate of 0.4 mL/min.

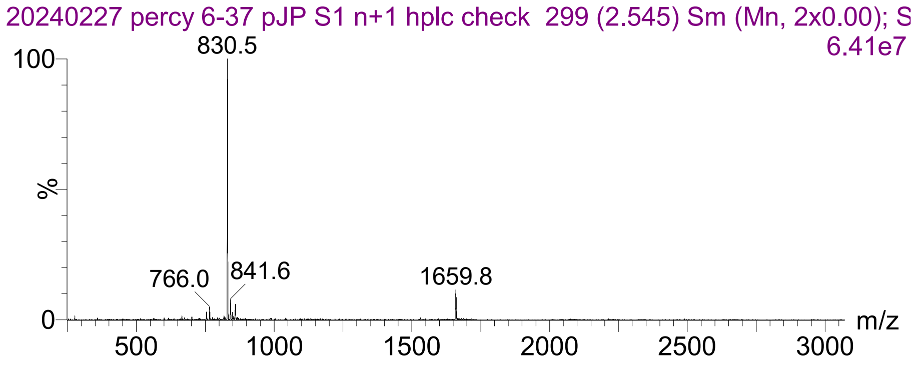

Figure S92. ESI-MS calcd. for $C_{70}H_{102}N_{18}O_{29}$ $[M+H]^+$ m/z = 1660.7, found 1659.8; $[M+2H]^{2+}$ m/z = 830.8, found 830.5.

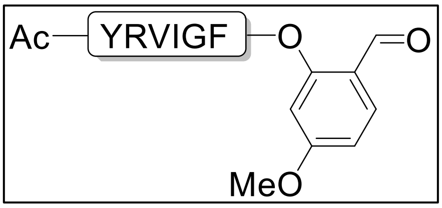

S28

Ac-YRVIGF-CO-SAL **S28** was synthesized according to the general synthesis of C-terminal Peptide SAL esters using n+1 strategy at 0.05 mmol scale. The crude peptide was purified by preparative reverse-phase HPLC (15-45% CH_3CN/H_2O over 45 min) and lyophilized to afford the desired peptide **S28** (10.6 mg, 22.4% yield).

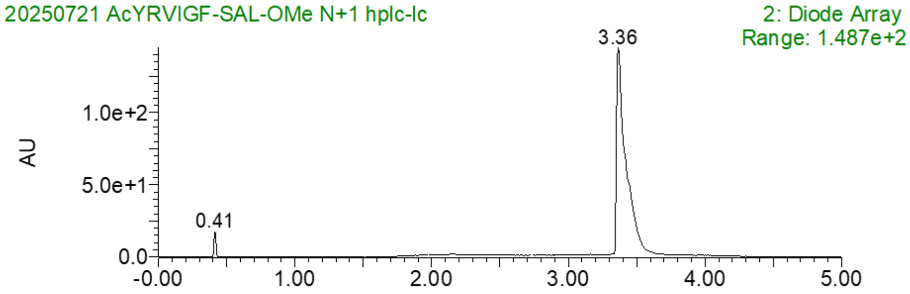

Figure S93. UV (190-400 nm) trace from UPLC-MS analysis of purified Ac-YRVIGF-CO-SAL **S28** gradient 5-95% CH_3CN/H_2O containing 0.1% TFA over 5 min at a flow rate of 0.4 mL/min.

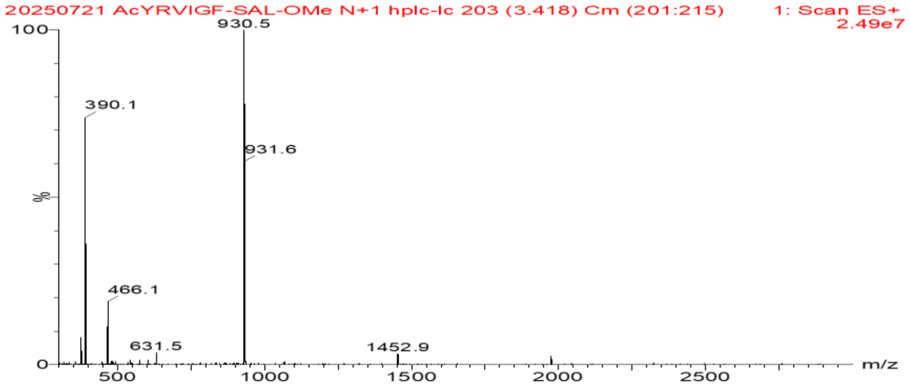

Figure S94. ESI-MS calcd. for $C_{47}H_{63}N_9O_{11}$ $[M+H]^+$ m/z = 931.0, found 930.5; $[M+2H]^{2+}$ m/z = 466.0, found 466.1.

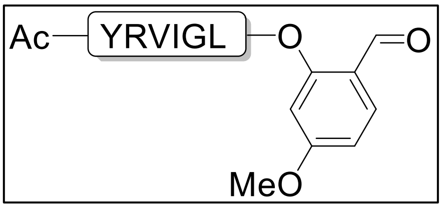

S29

Ac-YRVIGL-CO-SAL **S29** was synthesized according to the general synthesis of C-terminal Peptide SAL esters using n+1 strategy at 0.05 mmol scale. The crude peptide was purified by preparative reverse-phase HPLC (15-45% CH_3CN/H_2O over 45 min) and lyophilized to afford the desired peptide **S29** (23.9 mg, 50.7% yield).

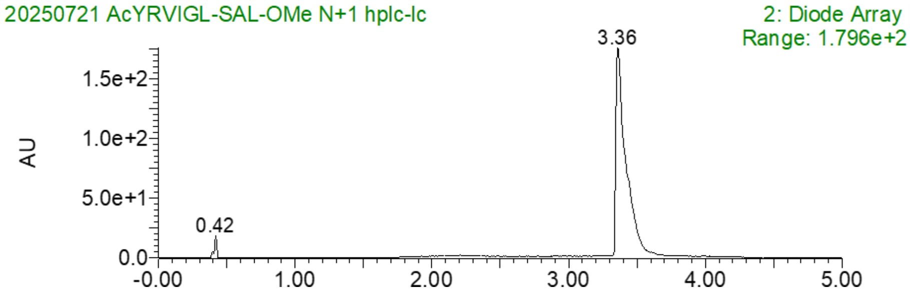

Figure S95. UV (190-400 nm) trace from UPLC-MS analysis of purified Ac-YRVIGL-CO-SAL **S29** gradient 5-95% CH_3CN/H_2O containing 0.1% TFA over 5 min at a flow rate of 0.4 mL/min.

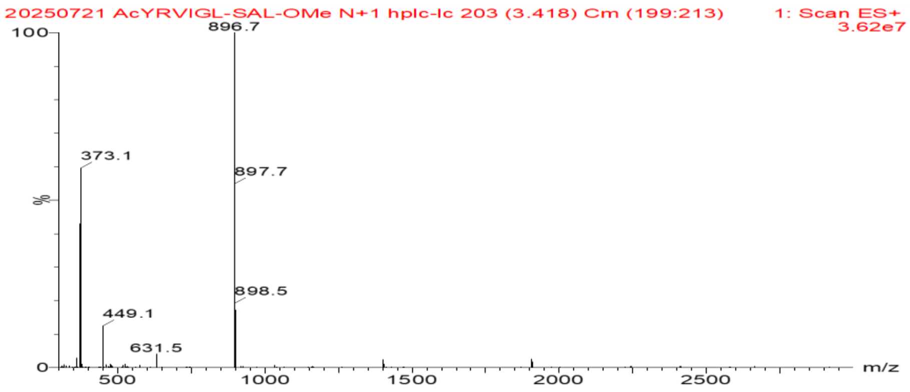

Figure S96. ESI-MS calcd. for $C_{44}H_{65}N_9O_{11}$ $[M+H]^+$ m/z = 897.0, found 896.7; $[M+2H]^{2+}$ m/z = 449.0, found 449.1.

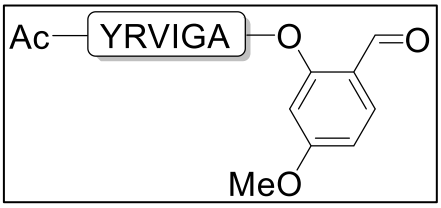

S30

Ac-YRVIGA-CO-SAL was synthesized according to the general synthesis of C-terminal Peptide SAL esters using n+1 strategy at 0.1 mmol scale. The crude peptide was purified by preparative reverse-phase HPLC (15-45% CH_3CN/H_2O over 45 min) and lyophilized to afford the desired peptide (53.5 mg, 66.2% yield).

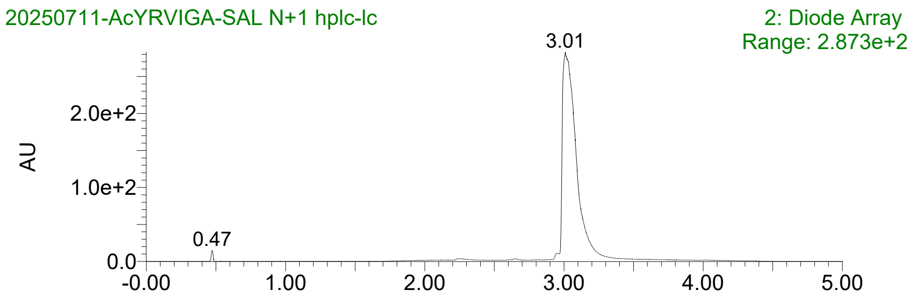

Figure S97. UV (190-400 nm) trace from UPLC-MS analysis of purified Ac-YRVIGA-CO-SAL **S30** gradient 5-95% CH_3CN/H_2O containing 0.1% TFA over 5 min at a flow rate of 0.4 mL/min.

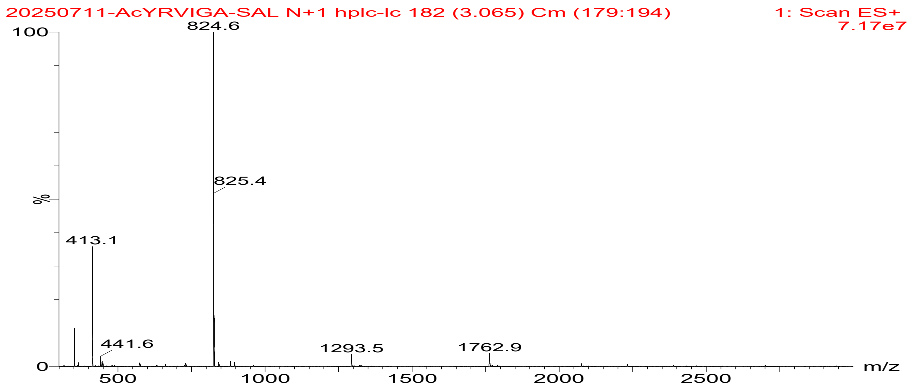

Figure S98. ESI-MS calcd. for $C_{40}H_{57}N_9O_{10}$ $[M+H]^+$ m/z = 824.9, found 824.6; $[M+2H]^{2+}$ m/z = 413.0, found 413.1.

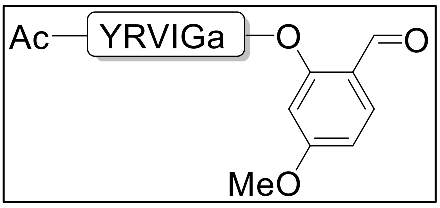

S31

Ac-YRVIGa-CO-SAL **S31** was synthesized according to the general synthesis of C-terminal Peptide SAL esters using n+1 strategy at 0.1 mmol scale. The crude peptide was purified by preparative reverse-phase HPLC (15-45% CH_3CN/H_2O over 45 min) and lyophilized to afford the desired peptide **S31** (72.1 mg, 83.4% yield).

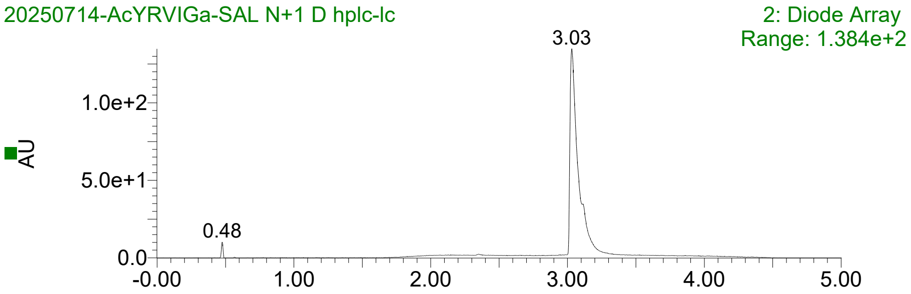

Figure S99. UV (190-400 nm) trace from UPLC-MS analysis of purified Ac-YRVIGa-CO-SAL gradient 5-95% CH_3CN/H_2O containing 0.1% TFA over 5 min at a flow rate of 0.4 mL/min.

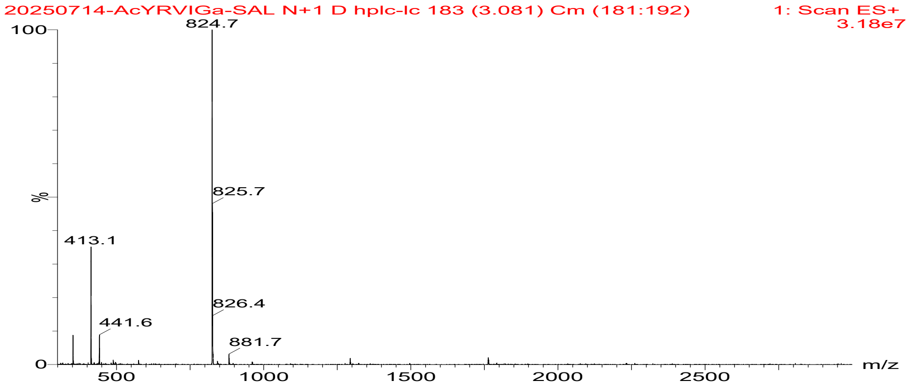

Figure S100. ESI-MS calcd. for $C_{40}H_{57}N_9O_{10}$ $[M+H]^+$ m/z = 824.9, found 824.7; $[M+2H]^{2+}$ m/z = 413.0, found 413.1.

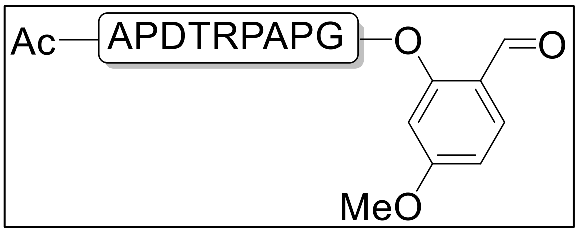

S32

Ac-APDTRPAPG-CO-SAL **S32** was synthesized according to the general synthesis of C-terminal Peptide SAL esters using n+1 strategy at 0.053 mmol scale. The crude peptide was purified by preparative reverse-phase HPLC (10-45% CH_3CN/H_2O over 45 min) and lyophilized to afford the desired peptide **S32** (40.3 mg, 67.2% yield).

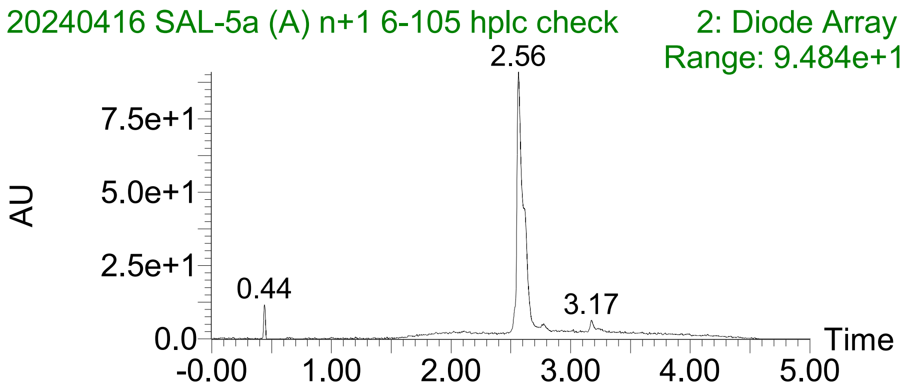

Figure S101. UV (190-400 nm) trace from UPLC-MS analysis of purified Ac-APDTRPAPG-CO-SAL **S32** gradient 5-95% CH_3CN/H_2O containing 0.1% TFA over 5 min at a flow rate of 0.4 mL/min.

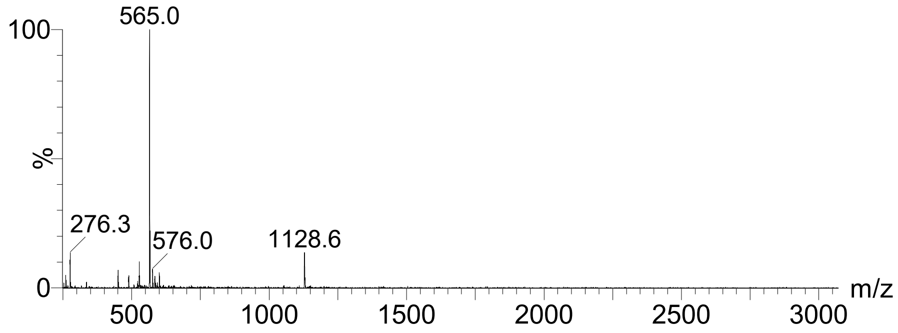

Figure S102. ESI-MS calcd. for $C_{50}H_{73}N_{13}O_{17}$ $[M+H]^+$ m/z = 1129.2, found 1128.6; $[M+2H]^{2+}$ m/z = 565.1, found 565.0.

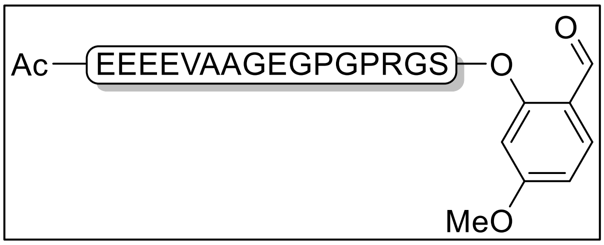

S33

Ac-EEEEVAAGEGPGPRGS-CO-SAL **S33** was synthesized according to the general synthesis of C-terminal Peptide SAL esters using n+1 strategy at 0.034 mmol scale. The crude peptide was purified by preparative reverse-phase HPLC (10-45% CH_3CN/H_2O over 45 min) and lyophilized to afford the desired peptide **S33** (40.3 mg, 67.2% yield).

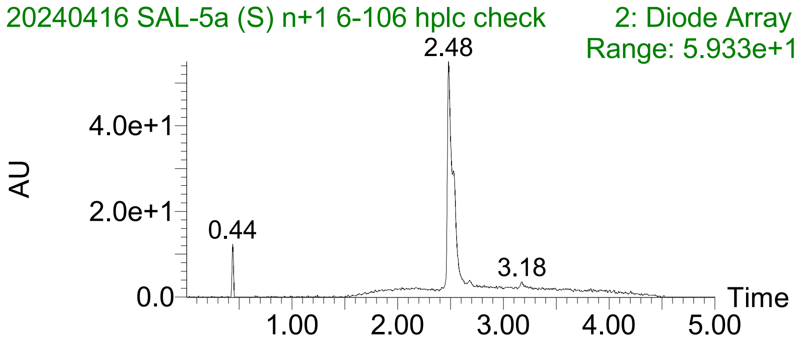

Figure S103. UV (190-400 nm) trace from UPLC-MS analysis of purified Ac-EEEEVAAGEGPGPRGS-CO-SAL **S33** gradient 5-95% CH_3CN/H_2O containing 0.1% TFA over 5 min at a flow rate of 0.4 mL/min.

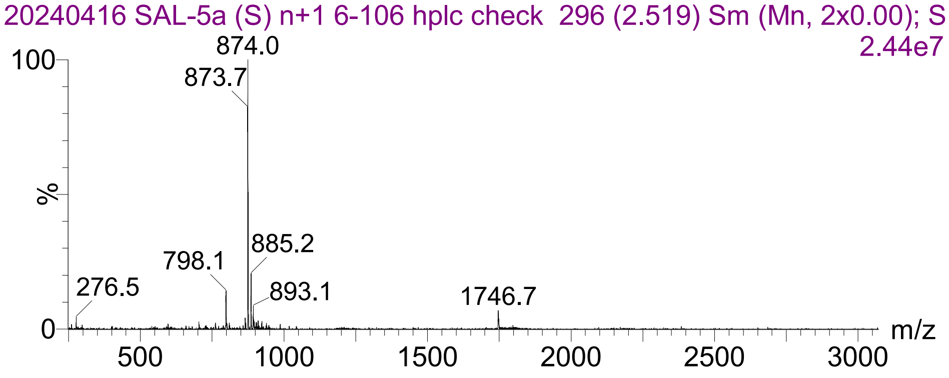

Figure S104. ESI-MS calcd. for $C_{73}H_{107}N_{19}O_{31}$ $[M+H]^+$ m/z = 1747.8, found 1746.7; $[M+2H]^{2+}$ m/z = 874.4, found 874.0.

5.1.3. Aminooxy ligation (AOL)

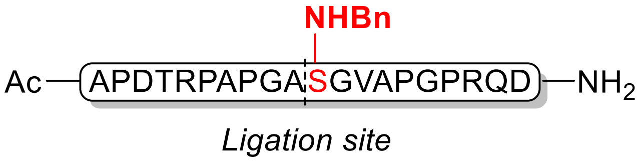

10

The ligation between Ac-APDTRPAPGA-CO-SAL (1.89 mg, 1.67 μmol) and H-(AO,Bn)GVAPGPRQD-NH_2 **S21** (2 mg, 1.84 μmol) was performed as described in the general procedure for aminooxy ligation in aqueous buffer at pH 4.5 for overnight. Purification via preparative reverse phase HPLC (10-35% CH_3CN/H_2O over 45 min, 0.1% TFA) followed by lyophilization afforded Ac-APDTRPAPGA(AO,Bn)GVAPGPRQD-NH_2 **10** (1.63 mg, 47.2% yield) as white solids.

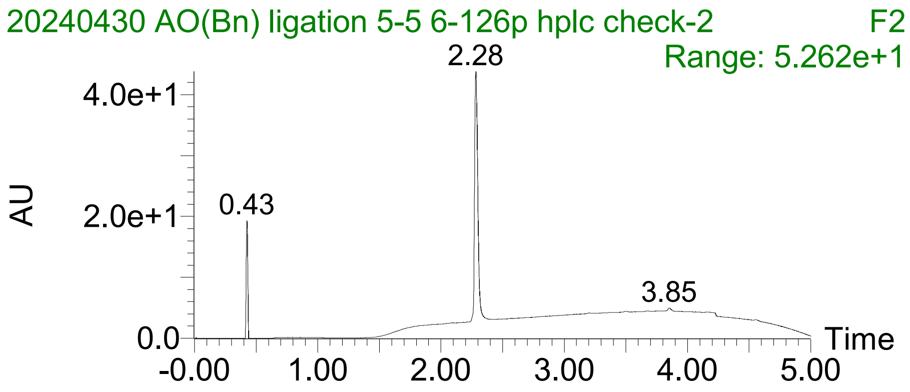

Figure S105. UV (190-400 nm) trace from UPLC-MS analysis of purified Ac-APDTRPAPGA(AO,Bn)GVAPGPRQD-NH_2 **10** gradient 5-95% CH_3CN/H_2O containing 0.1% TFA over 5 min at a flow rate of 0.4 mL/min.

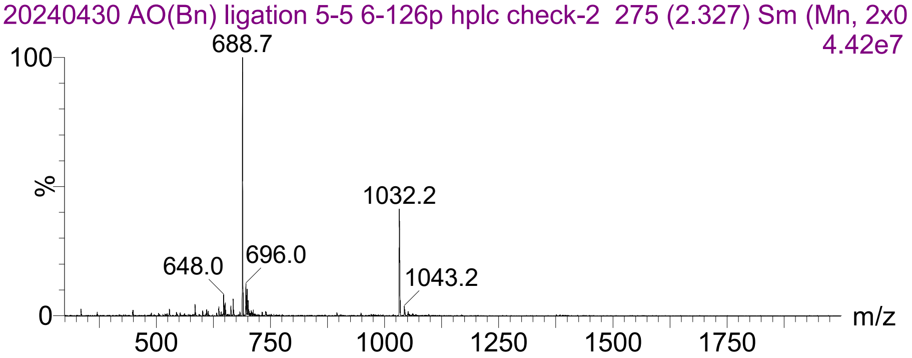

Figure S106. ESI-MS calcd. for $C_{89}H_{139}N_{29}O_{28}$ $[M+2H]^{2+}$ m/z = 1032.6, found 1032.2; $[M+3H]^{3+}$ m/z = 688.8, found 688.7.

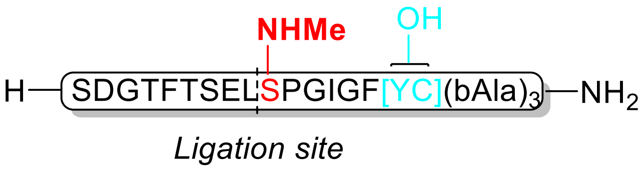

11

The ligation between H-SDGTFTSEL-CO-SAL (3 mg, 2.75 μmol) and H-(AO,Me)PGIGF[YC](β-Ala)$_3$-NH_2 **S25** (4.02 mg, 3.30 μmol) was performed as described in the general procedure for aminooxy ligation in aqueous buffer at pH 4.0 for overnight. Purification via preparative reverse phase HPLC (20-45% CH_3CN/H_2O over 45 min, 0.1% TFA) followed by lyophilization afforded H-SDGTFTSEL(AO,Me)PGIGF[YC](β-Ala)$_3$-NH_2 **11** (2.29 mg, 38.6% yield) as white solids.

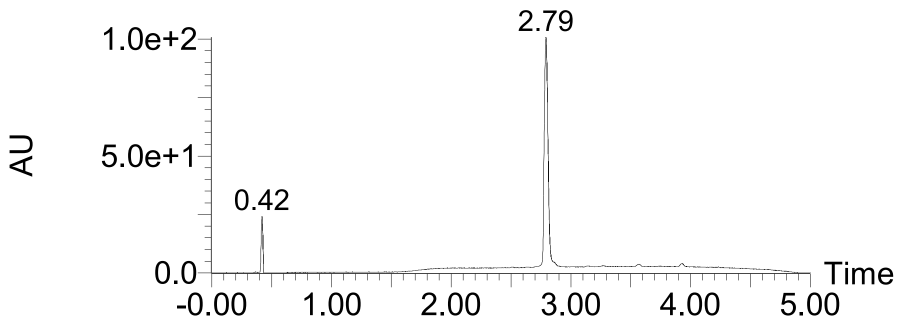

Figure S107. UV (190-400 nm) trace from UPLC-MS analysis of purified H-SDGTFTSEL(AO,Me)PGIGF[YC](β-Ala)$_3$-NH$_2$ **11** gradient 5-95% CH_3CN/H_2O containing 0.1% TFA over 5 min at a flow rate of 0.4 mL/min.

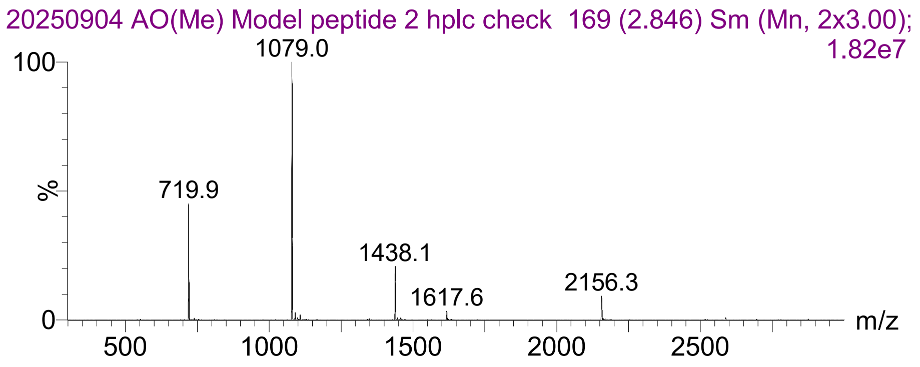

Figure S108. ESI-MS calcd. for $C_{112}H_{173}N_{35}O_{42}$ $[M+H]^+$ m/z = 2157.4, found 2156.3; $[M+2H]^{2+}$ m/z = 1079.2, found 1079.0; $[M+3H]^{3+}$ m/z = 719.8, found 719.9.

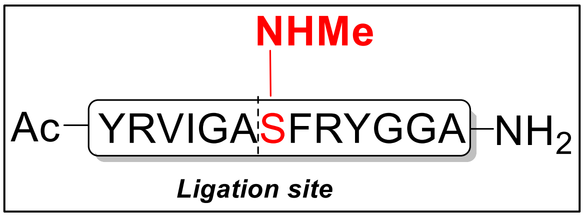

12

The ligation between Ac-YRVIGA-CO-SAL **S30** (4.4 mg, 5.33 μmol) and H-(AO,Me)FRYGGA-NH$_2$ **S15** (4.6 mg, 5.86 μmol) was performed as described in the general procedure for aminooxy ligation in Pyr/HOAc 1:3 for overnight.

Purification via preparative reverse phase HPLC (10-40% CH_3CN/H_2O over 45 min, 0.1% TFA) followed by lyophilization afforded Ac-YRVIGA(AO,Me)FRYGGA-NH_2 **12** (4.96 mg, 62.6% yield) as white solids.

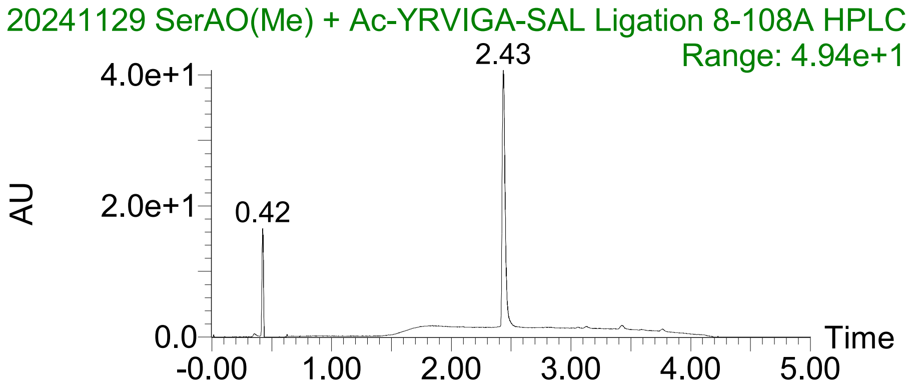

Figure S109. UV (190-400 nm) trace from UPLC-MS analysis of purified Ac-YRVIGA(AO,Me)FRYGGA-NH_2 **12** gradient 5-95% CH_3CN/H_2O containing 0.1% TFA over 5 min at a flow rate of 0.4 mL/min.

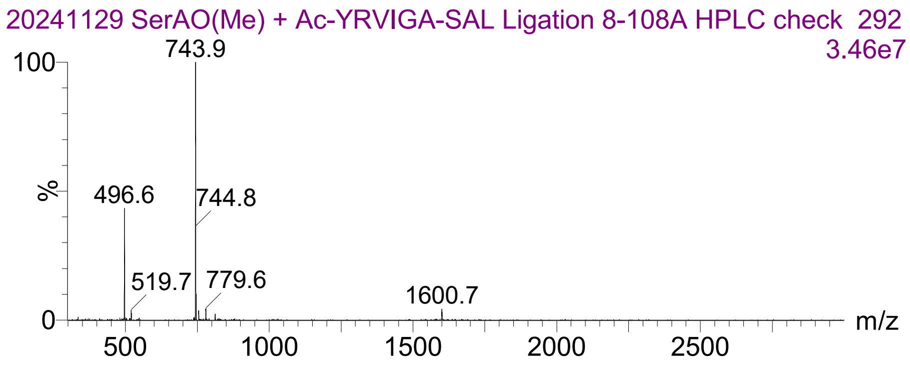

ESI-MS calcd. for $C_{68}H_{103}N_{21}O_{17}$ $[M+2H]^{2+}$ m/z = 744.4, found 743.9; $[M+3H]^{3+}$ m/z = 496.64, found 496.6.

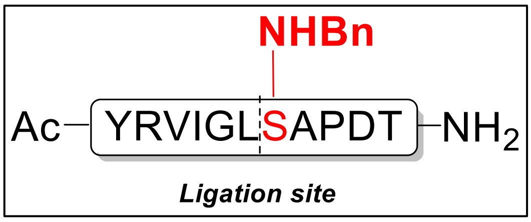

13

The ligation between Ac-YRVIGL-CO-SAL **S29** (1.34 mg, 1.5 μmol) and H-(AO,Bn)APDT-NH_2 (0.98 mg, 1.65 μmol) **S18** was performed as described in the general procedure for aminooxy ligation in aqueous buffer at pH 4.5 for overnight. Purification via preparative reverse phase HPLC (20-45% CH_3CN/H_2O over 45 min, 0.1% TFA) followed by lyophilization afforded Ac-YRVIGL(AO,Bn)APDT-NH_2 **13** (1.19 mg, 59.3% yield) as white solids.

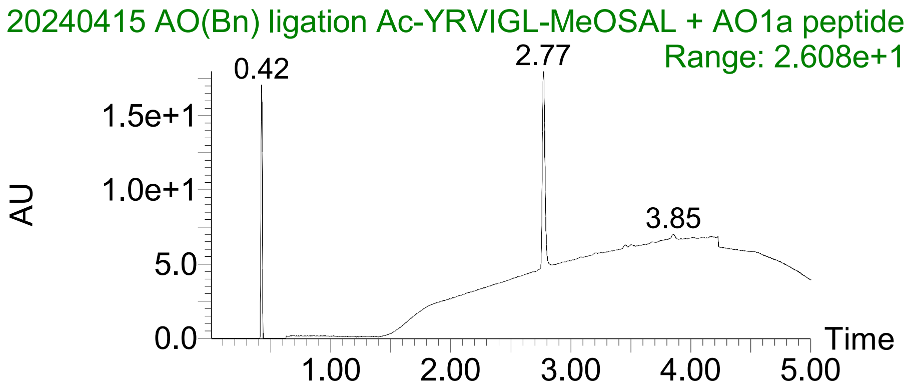

Figure S110. UV (190-400 nm) trace from UPLC-MS analysis of purified Ac-YRVIGL(AO,Bn)APDT-NH_2 **13** gradient 5-95% CH_3CN/H_2O containing 0.1% TFA over 5 min at a flow rate of 0.4 mL/min.

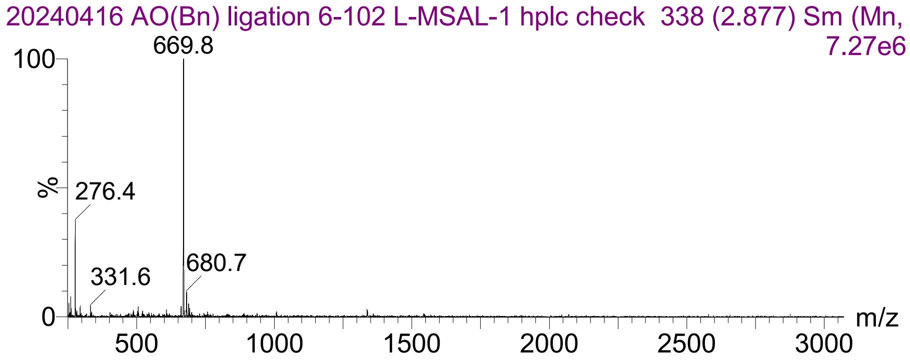

Figure S111. ESI-MS calcd. for $C_{62}H_{96}N_{16}O_{17}$ $[M+2H]^{2+}$ m/z = 669.8, found 669.8.

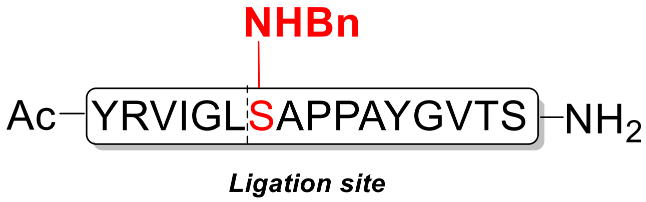

14

The ligation between Ac-YRVIGL-CO-SAL **S29** (1.34 mg, 1.5 μmol) and H-(AO,Bn)APPAYGVTS-NH_2 **S19** (1.74 mg, 1.65 μmol) was performed as described in the general procedure for aminooxy ligation in aqueous buffer at pH 4.5 for overnight. Purification via preparative reverse phase HPLC (20-45% CH_3CN/H_2O over 45 min, 0.1% TFA) followed by lyophilization afforded Ac-YRVIGL(AO,Bn)APPAYGVTS-NH_2 **14** (1.32 mg, 49.0% yield) as white solids.

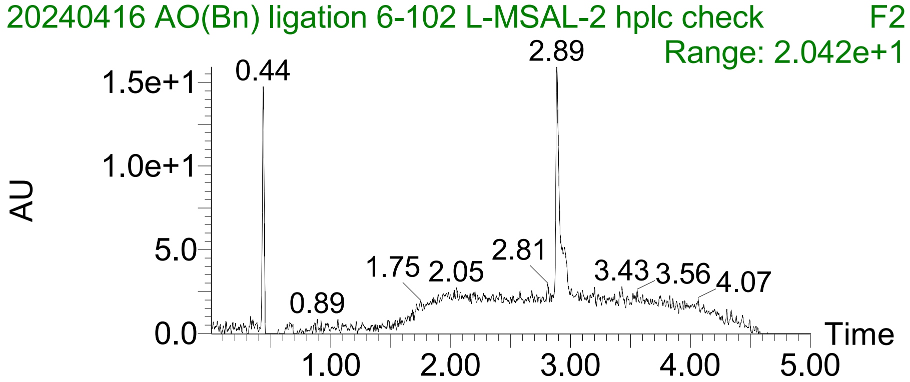

Figure S112. UV (190-400 nm) trace from UPLC-MS analysis of purified Ac-YRVIGL(AO,Bn)APPAYGVTS-NH_2 **14** gradient 5-95% CH_3CN/H_2O containing 0.1% TFA over 5 min at a flow rate of 0.4 mL/min.

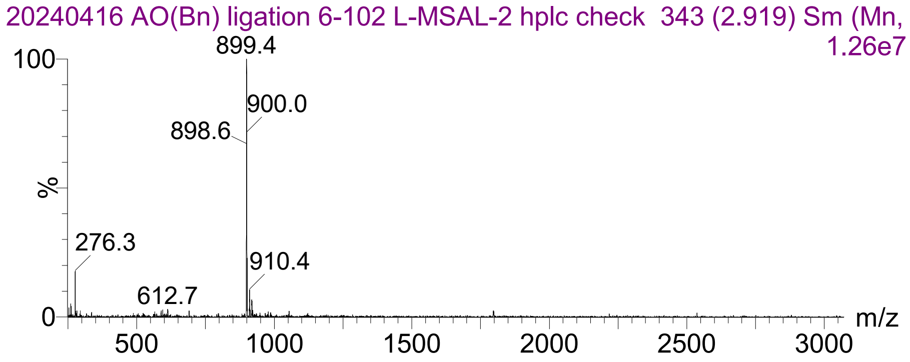

Figure S113. ESI-MS calcd. for $C_{85}H_{129}N_{21}O_{22}$ $[M+2H]^{2+}$ m/z = 899.5, found 899.4.

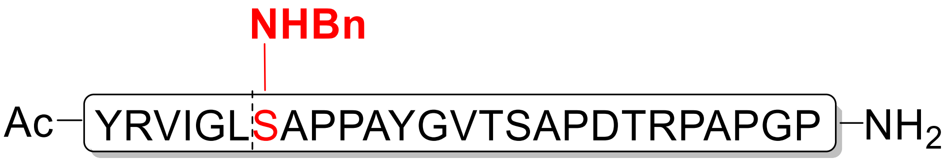

15

The ligation between Ac-YRVIGL-CO-SAL **S29** (1.34 mg, 1.5 μmol) and H-(AO,Bn)APPAYGVTSAPDTRPAPGP-NH_2 **S20** (3.32 mg, 1.65 μmol) was performed as described in the general procedure for aminooxy ligation in aqueous buffer at pH 4.5 for overnight. Purification via preparative reverse phase HPLC (20-45% CH_3CN/H_2O over 45 min, 0.1% TFA) followed by lyophilization afforded Ac-YRVIGL(AO,Bn)APPAYGVTSAPDTRPAPGP-NH_2 **15** (2.50 mg, 60.4% yield) as white solids.

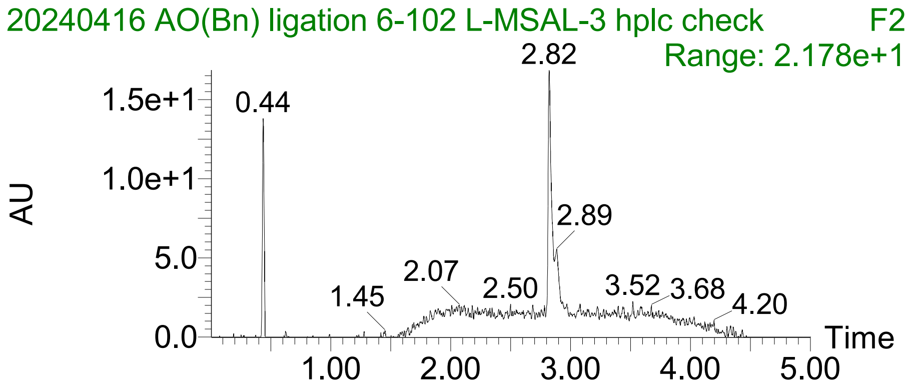

Figure S114. UV (190-400 nm) trace from UPLC-MS analysis of purified Ac-YRVIGL(AO,Bn)APPAYGVTSAPDTRPAPGP-NH_2 **15** gradient 5-95% CH_3CN/H_2O containing 0.1% TFA over 5 min at a flow rate of 0.4 mL/min.

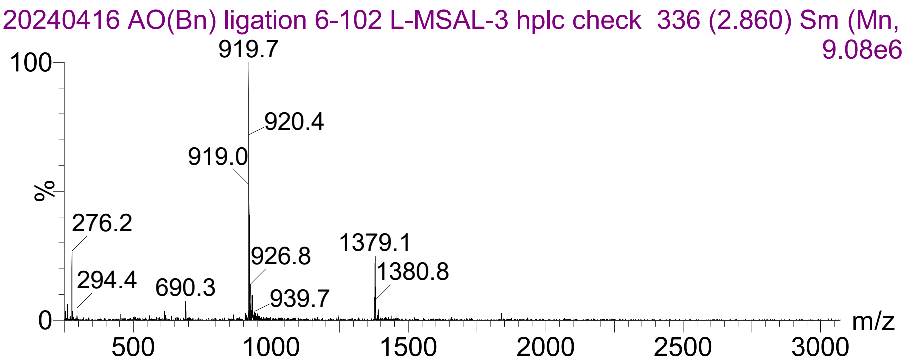

Figure S115. ESI-MS calcd. for $C_{127}H_{194}N_{34}O_{35}$ $[M+2H]^{2+}$ m/z = 1379.6, found 1379.1; $[M+3H]^{3+}$ m/z = 920.0, found 919.7; $[M+4H]^{4+}$ m/z = 690.2, found 690.3.

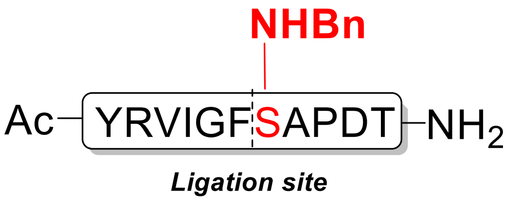

16

The ligation between Ac-YRVIGF-CO-SAL **S28** (1.4 mg, 1.5 μmol) and H-(AO,Bn)APDT-NH_2 **S18** (0.98 mg, 1.65 μmol) was performed as described in the general procedure for aminooxy ligation in aqueous buffer at pH 4.5 with 10% aqueous DMSO for overnight. Purification via preparative reverse phase HPLC (10-40% CH_3CN/H_2O over 45 min, 0.1% TFA) followed by lyophilization afforded Ac-YRVIGF(AO,Bn)APDT-NH_2 **16** (1.14 mg, 55.4% yield) as white solids.

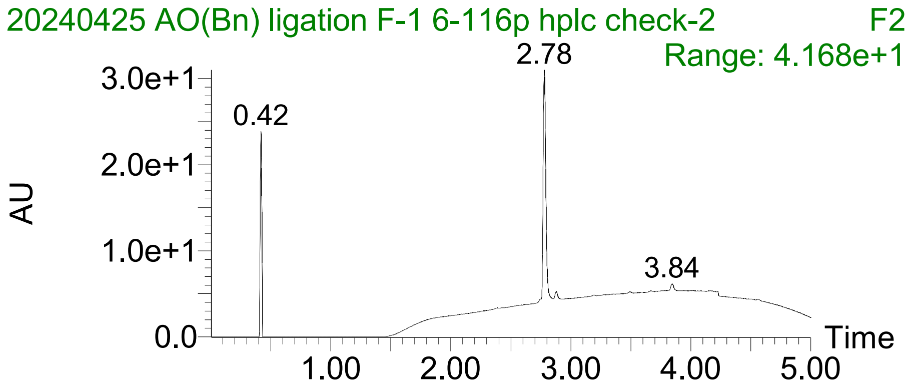

Figure S116. UV (190-400 nm) trace from UPLC-MS analysis of purified Ac-YRVIGF(AO,Bn)APDT-NH_2 **16** gradient 5-95% CH_3CN/H_2O containing 0.1% TFA over 5 min at a flow rate of 0.4 mL/min.

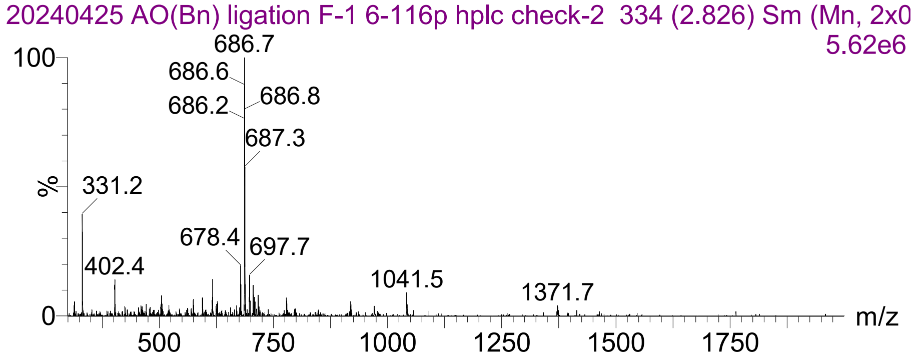

Figure S117. ESI-MS calcd. for $C_{65}H_{94}N_{16}O_{17}$ $[M+H]^+$ m/z = 1372.6, found 1371.7; $[M+2H]^{2+}$ m/z = 686.8, found 686.7.

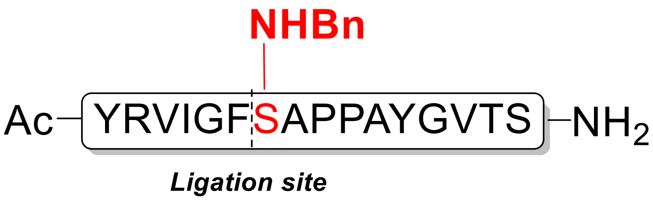

17

The ligation between Ac-YRVIGF-CO-SAL **S28** (1.4 mg, 1.5 μmol) and H-(AO,Bn)APPAYGVTS-NH_2 (1.74 mg, 1.65 μmol) **S19** was performed as described in the general procedure for aminooxy ligation in aqueous buffer at pH 4.5 for overnight. Purification via preparative reverse phase HPLC (10-40%

CH$_3$CN/H$_2$O over 45 min, 0.1% TFA) followed by lyophilization afforded Ac-YRVIGF(AO,Bn)APPAYGVTS-NH$_2$ **17** (8.03 mg, 50.2% yield) as white solids.

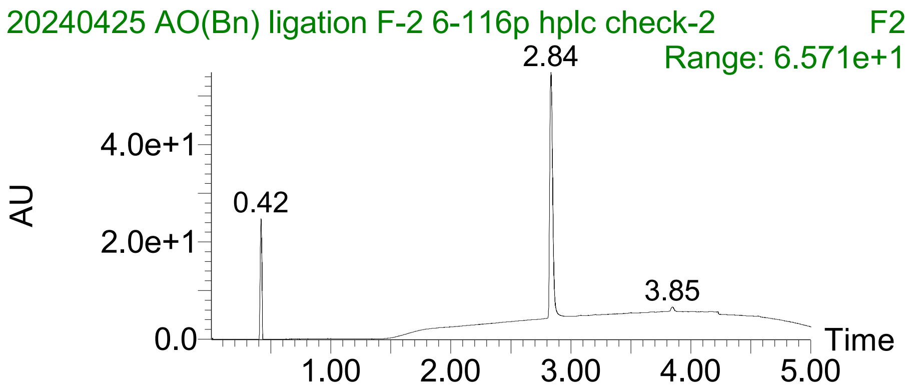

Figure S118. UV (190-400 nm) trace from UPLC-MS analysis of purified Ac-YRVIGF(AO,Bn)APPAYGVTS-NH$_2$ **17** gradient 5-95% CH$_3$CN/H$_2$O containing 0.1% TFA over 5 min at a flow rate of 0.4 mL/min.

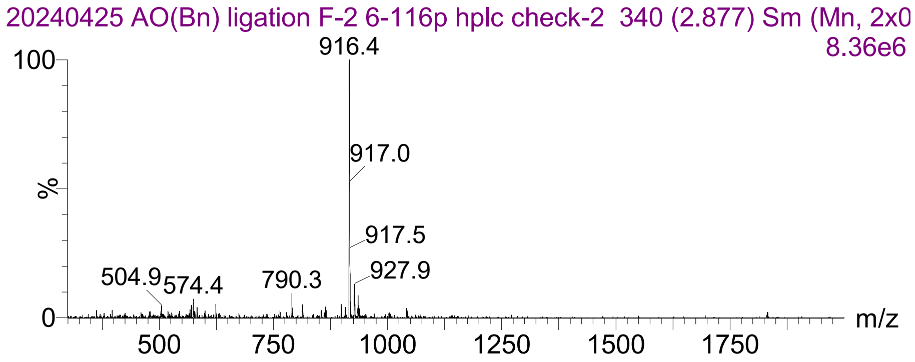

Figure S119. ESI-MS calcd. for $C_{88}H_{127}N_{21}O_{22}$ $[M+2H]^{2+}$ m/z = 916.6, found 916.4.

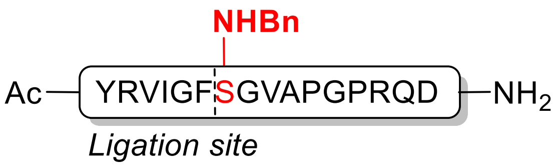

18

The ligation between Ac-YRVIGF-CO-SAL **S28** (1.56 mg, 1.67 μmol) and H-(AO,Bn)GVAPGPRQD-NH$_2$ **S21** (2 mg, 1.84 μmol) was performed as described in the

general procedure for aminooxy ligation in aqueous buffer at pH 4.5 with 10% aqueous DMSO for overnight. Purification via preparative reverse phase HPLC (15-45% CH_3CN/H_2O over 45 min, 0.1% TFA) followed by lyophilization afforded Ac-YRVIGF(AO,Bn)GVAPGPRQD-NH_2 **18** (1.08 mg, 34.6% yield) as white solids.

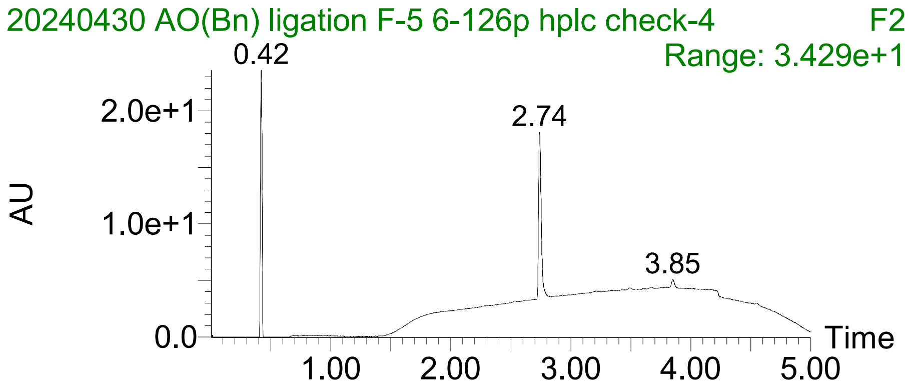

Figure S120. UV (190-400 nm) trace from UPLC-MS analysis of purified Ac-YRVIGF(AO,Bn)GVAPGPRQD-NH_2 **18** gradient 5-95% CH_3CN/H_2O containing 0.1% TFA over 5 min at a flow rate of 0.4 mL/min.

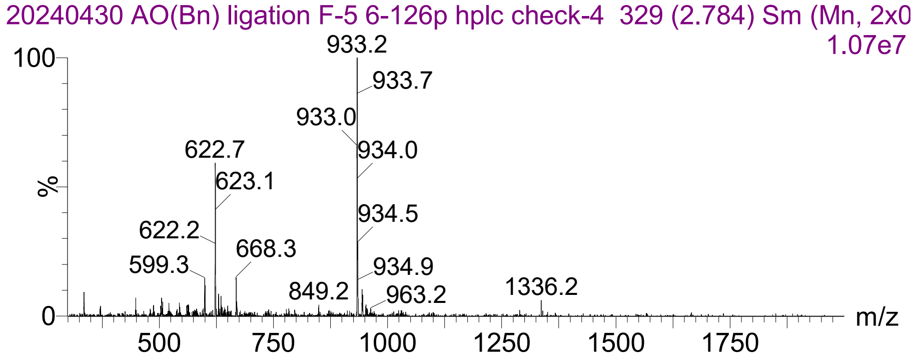

Figure S121. ESI-MS calcd. for $C_{86}H_{129}N_{25}O_{22}$ $[M+2H]^{2+}$ m/z = 933.6, found 933.2; $[M+3H]^{3+}$ m/z = 622.7, found 622.7.

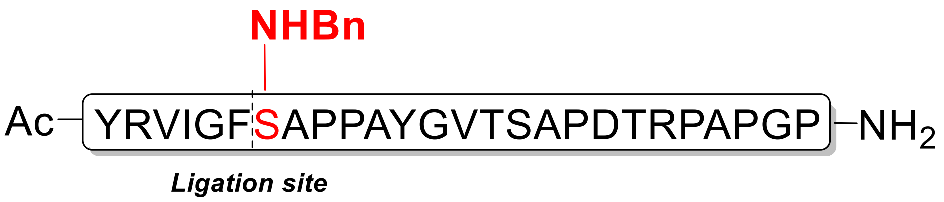

19

The ligation between Ac-YRVIGF-CO-SAL **S28** (1.4 mg, 1.5 μmol) and H-(AO,Bn)APPAYGVTSAPDTRPAPGP-NH_2 **S20** (3.32 mg, 1.65 μmol) was performed as described in the general procedure for aminooxy ligation in aqueous buffer at pH 4.5 for overnight. Purification via preparative reverse phase HPLC (20-45% CH_3CN/H_2O over 45 min, 0.1% TFA) followed by lyophilization afforded Ac-YRVIGF(AO,Bn)APPAYGVTSAPDTRPAPGP-NH_2 **19** (1.88 mg, 44.9% yield) as white solids.

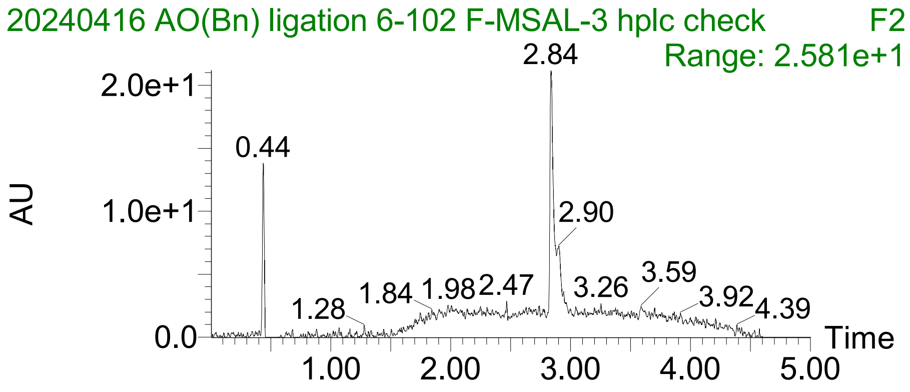

Figure S122. UV (190-400 nm) trace from UPLC-MS analysis of purified Ac-YRVIGF(AO,Bn)APPAYGVTSAPDTRPAPGP-NH_2 **19** gradient 5-95% CH_3CN/H_2O containing 0.1% TFA over 5 min at a flow rate of 0.4 mL/min.

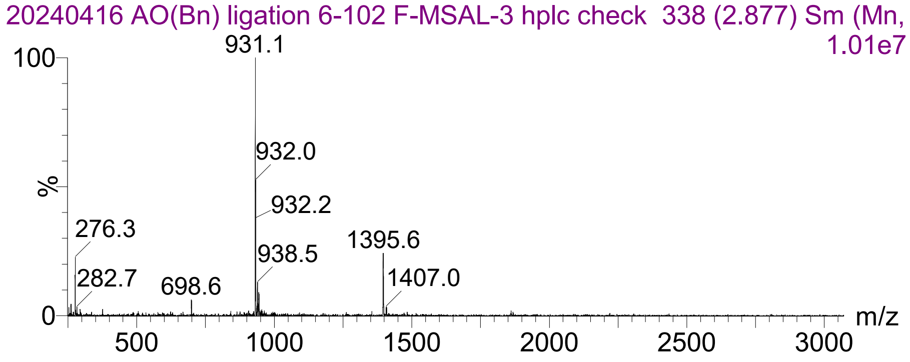

Figure S123. ESI-MS calcd. for $C_{130}H_{192}N_{34}O_{35}$ $[M+2H]^{2+}$ m/z = 1396.6, found 1395.6; $[M+3H]^{3+}$ m/z = 931.1, found 931.1; $[M+4H]^{4+}$ m/z = 698.8, found 698.6.

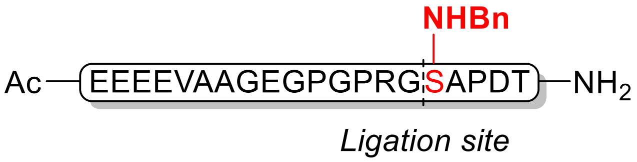

20

The ligation between Ac-EEEEVAAGEGPGPRG-CO-SAL **S27** (2 mg, 1.21 μmol) and H-(AO,Bn)APDT-NH_2 **S18** (0.79 mg, 1.33 μmol) was performed as described in the general procedure for aminooxy ligation in aqueous buffer at pH 4.5 for overnight. Purification via preparative reverse phase HPLC (10-30% CH_3CN/H_2O over 45 min, 0.1% TFA) followed by lyophilization afforded Ac-EEEEVAAGEGPGPRG(AO,Bn)APDT-NH_2 **20** (1.39 mg, 54.9% yield) as white solids.

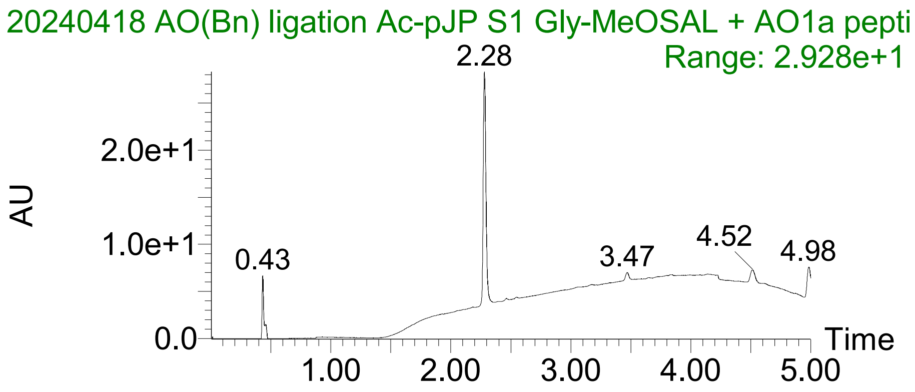

Figure S124. UV (190-400 nm) trace from UPLC-MS analysis of purified Ac-EEEEVAAGEGPGPRG(AO,Bn)APDT-NH_2 **20** gradient 5-95% CH_3CN/H_2O containing 0.1% TFA over 5 min at a flow rate of 0.4 mL/min.

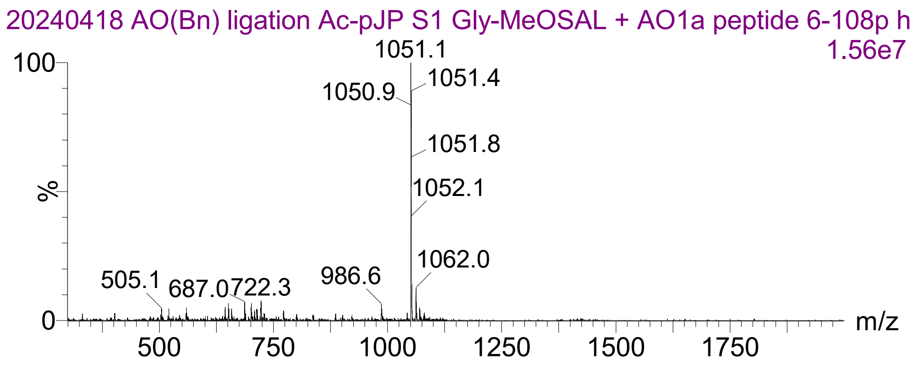

Figure S125. ESI-MS calcd. for $C_{88}H_{133}N_{25}O_{35}$ $[M+2H]^{2+}$ m/z = 1051.6, found 1051.5.

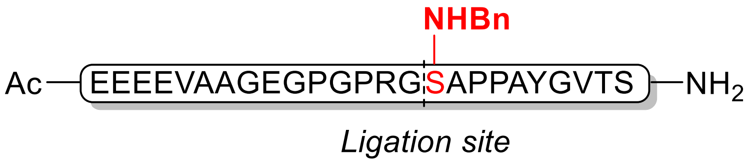

21

The ligation between Ac-EEEEVAAGEGPGPRG-CO-SAL **S27** (2 mg, 1.21 μmol) and H-(AO,Bn)APPAYGVTS-NH2 **S19** (1.40 mg, 1.33 μmol) was performed as described in the general procedure for aminooxy ligation in aqueous buffer at pH 4.5 for overnight. Purification via preparative reverse phase HPLC (10-40% CH_3CN/H_2O over 45 min, 0.1% TFA) followed by lyophilization afforded Ac-EEEEVAAGEGPGPRG(AO,Bn)APPAYGVTS-NH2 **21** (1.75 mg, 56.7% yield) as white solids.

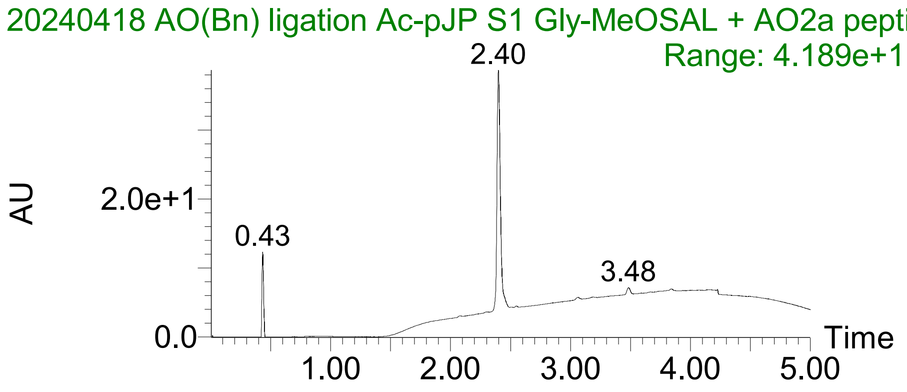

Figure S126. UV (190-400 nm) trace from UPLC-MS analysis of purified Ac-EEEEVAAGEGPGPRG(AO,Bn)APPAYGVTS-NH_2 **21** gradient 5-95% CH_3CN/H_2O containing 0.1% TFA over 5 min at a flow rate of 0.4 mL/min.

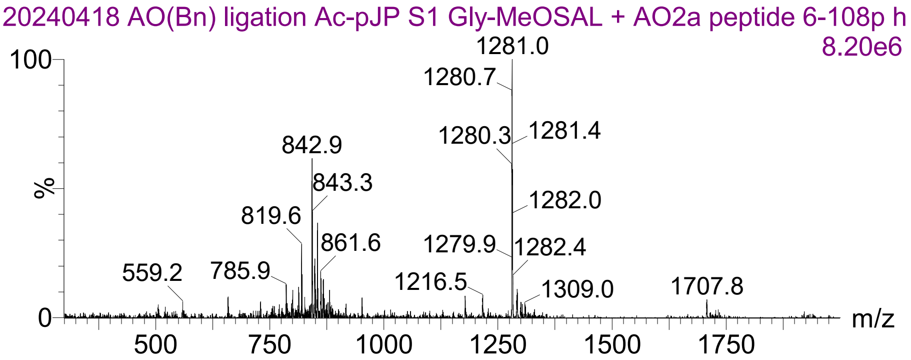

Figure S127. ESI-MS calcd. for $C_{111}H_{166}N_{30}O_{40}$ $[M+2H]^{2+}$ m/z = 1281.4, found 1281.0. $[M+3H]^{3+}$ m/z = 854.6, found 854.7.

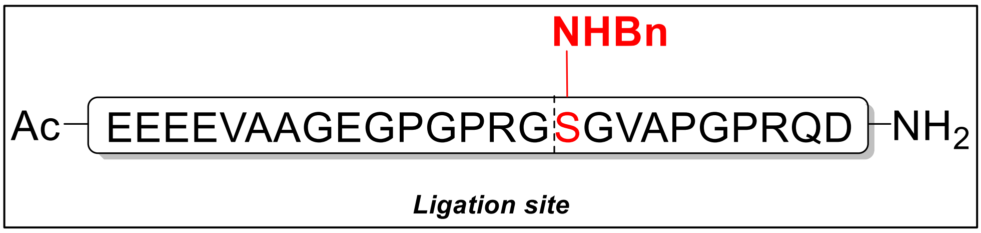

22

The ligation between Ac-EEEEVAAGEGPGPRG-CO-SAL **S27** (2.78 mg, 1.67 μmol) and H-(AO,Bn)GVAPGPRQD-NH_2 **S21** (2 mg, 1.84 μmol) was performed as described in the general procedure for aminooxy ligation in aqueous buffer at pH 4.5 for overnight. Purification via preparative reverse phase HPLC (5-30% CH_3CN/H_2O over 45 min, 0.1% TFA) followed by lyophilization afforded Ac-EEEEVAAGEGPGPRG(AO,Bn)GVAPGPRQD-NH_2 **22** (2.77 mg, 63.8% yield) as white solids.

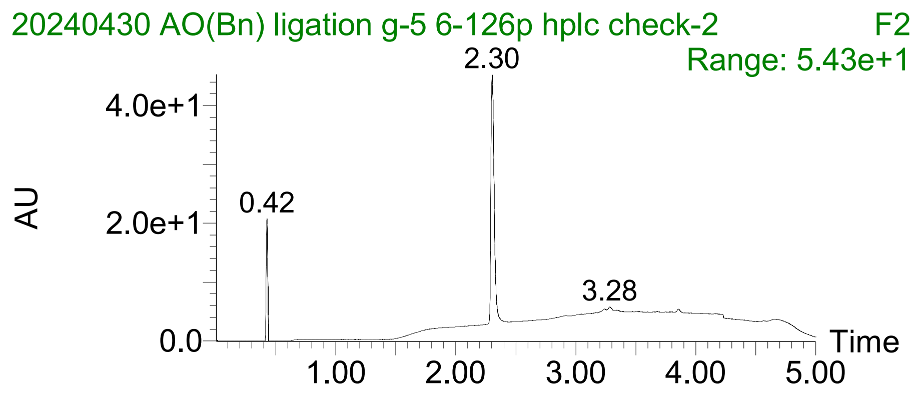

Figure S128. UV (190-400 nm) trace from UPLC-MS analysis of purified Ac-EEEEVAAGEGPGPRG(AO,Bn)GVAPGPRQD-NH_2 **22** gradient 5-95% CH_3CN/H_2O containing 0.1% TFA over 5 min at a flow rate of 0.4 mL/min.

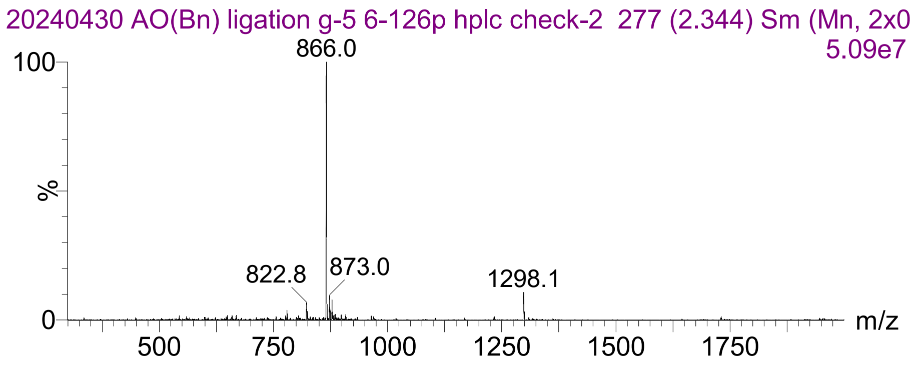

Figure S129. ESI-MS calcd. for $C_{109}H_{168}N_{34}O_{40}$ $[M+2H]^{2+}$ m/z = 1298.4, found 1298.1; $[M+3H]^{3+}$ m/z = 865.9, found 866.0.

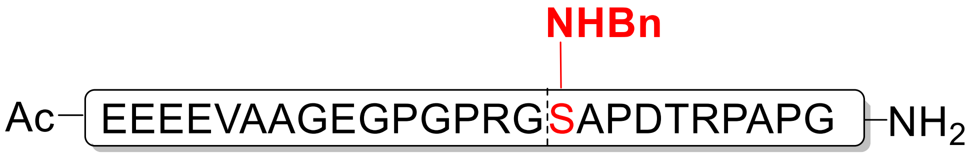

Ligation site

23

The ligation between Ac-EEEEVAAGEGPGPRG-CO-SAL **S27** (14.1 mg, 8.47 μmol) and H-(AO,Bn)APDTRPAPG-OH **S22** (10 mg, 9.32 μmol) was performed as described in the general procedure for aminooxy ligation in aqueous buffer

at pH 4.5 for overnight. Purification via preparative reverse phase HPLC (5-40% CH_3CN/H_2O over 45 min, 0.1% TFA) followed by lyophilization afforded Ac-EEEEVAAGEGPGPRG(AO,Bn)APDTRPAPG-OH **23** (12.64 mg, 57.8% yield) as white solids.

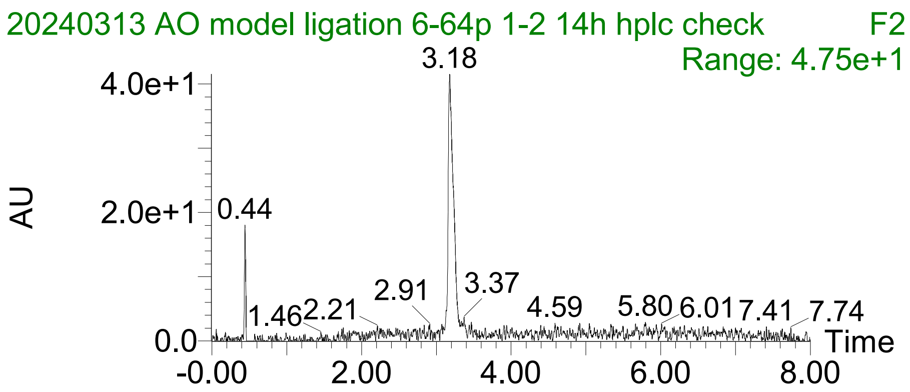

Figure S130. UV (190-400 nm) trace from UPLC-MS analysis of purified Ac-EEEEVAAGEGPGPRG(AO,Bn)APDTRPAPG-OH **23** gradient 5-95% CH_3CN/H_2O containing 0.1% TFA over 8 min at a flow rate of 0.4 mL/min.

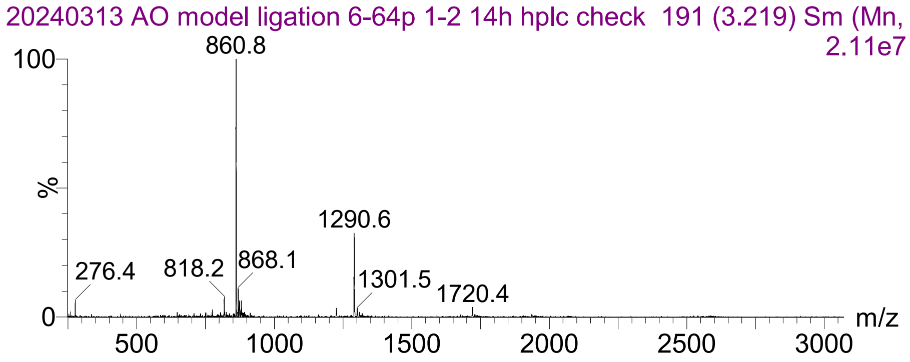

Figure S131. ESI-MS calcd. for $C_{109}H_{166}N_{32}O_{41}$ $[M+2H]^{2+}$ m/z = 1291.4, found 1290.6; $[M+3H]^{3+}$ m/z = 861.2, found 860.8.

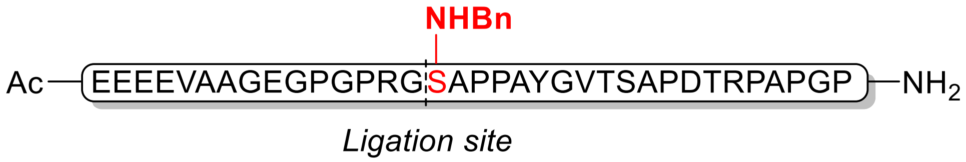

24

The ligation between Ac-EEEEVAAGEGPGPRG-CO-SAL **S27** (2 mg, 1.21 μmol) and H-(AO,Bn)APPAYGVTSAPDTRPAPGP-NH_2 **S20** (2.67 mg, 1.33 μmol) was performed as described in the general procedure for aminooxy ligation in aqueous buffer at pH 4.5 for overnight. Purification via preparative reverse phase HPLC (10-40% CH_3CN/H_2O over 45 min, 0.1% TFA) followed by lyophilization afforded Ac-EEEEVAAGEGPGPRG(AO,Bn)APPAYGVTSAPDTRPAPGP-NH_2 **24** (2.85 mg, 67.2% yield) as white solids.

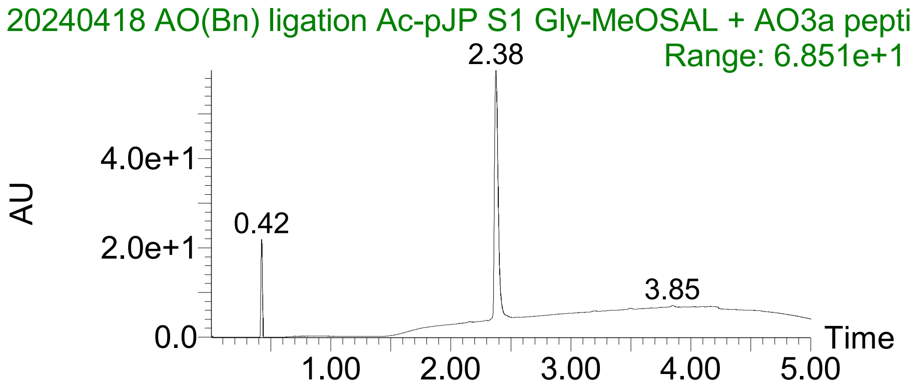

Figure S132. UV (190-400 nm) trace from UPLC-MS analysis of purified Ac-EEEEVAAGEGPGPRG(AO,Bn)APPAYGVTSAPDTRPAPGP-NH_2 **24** gradient 5-95% CH_3CN/H_2O containing 0.1% TFA over 5 min at a flow rate of 0.4 mL/min.

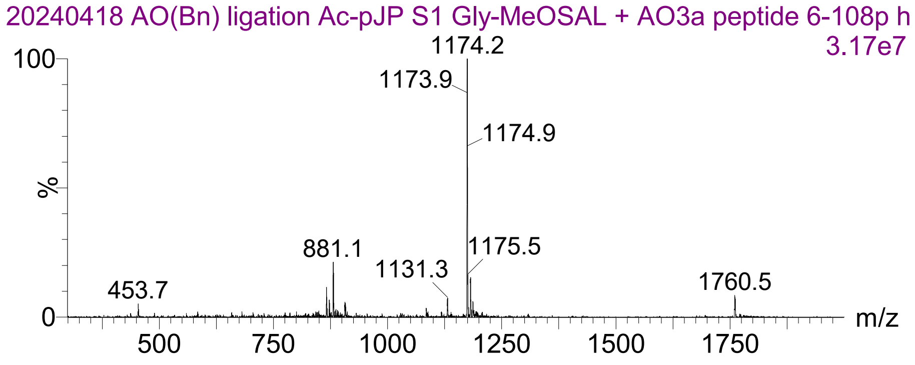

Figure S133. ESI-MS calcd. for $C_{153}H_{231}N_{43}O_{53}$ $[M+2H]^{2+}$ m/z = 1761.4, found 1760.5. $[M+3H]^{3+}$ m/z = 1174.6, found 1174.2; $[M+4H]^{4+}$ m/z = 881.2, found

881.1.

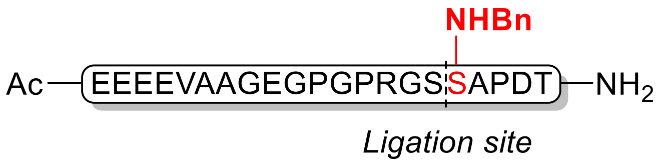

25

The ligation between Ac-EEEEVAAGEGPGPRGS-CO-SAL **S33** (2.62 mg, 1.5 μmol) and H-(AO,Bn)APDT-NH_2 **S18** (0.98 mg, 1.65 μmol) was performed as described in the general procedure for aminooxy ligation in aqueous buffer at pH 4.5 for overnight. Purification via preparative reverse phase HPLC (10-30% CH_3CN/H_2O over 45 min, 0.1% TFA) followed by lyophilization afforded Ac-EEEEVAAGEGPGPRGS(AO,Bn)APDT-NH_2 **25** (1.09 mg, 33.2% yield) as white solids.

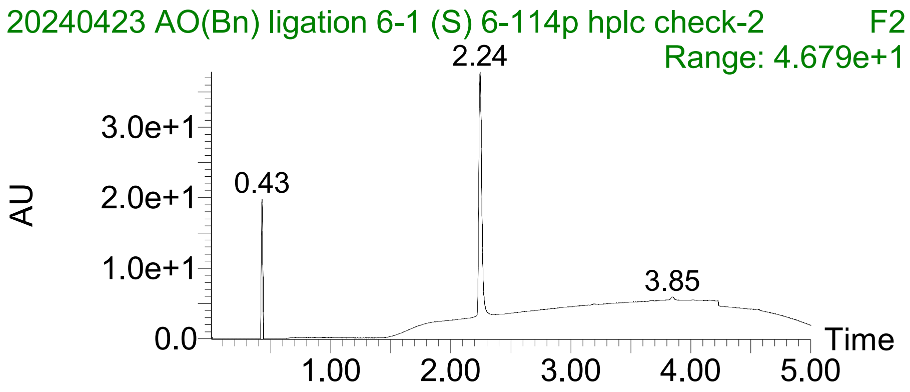

Figure S134. UV (190-400 nm) trace from UPLC-MS analysis of purified Ac-EEEEVAAGEGPGPRGS(AO,Bn)APDT-NH_2 **25** gradient 5-95% CH_3CN/H_2O containing 0.1% TFA over 5 min at a flow rate of 0.4 mL/min.

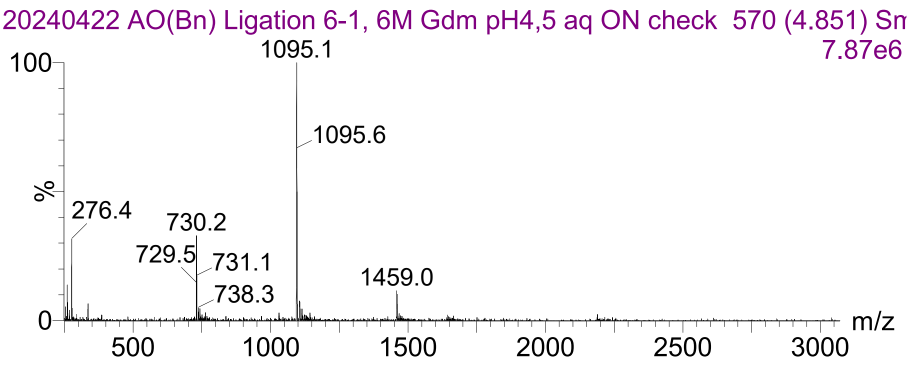

Figure S135. ESI-MS calcd. for $C_{91}H_{138}N_{26}O_{37}$ $[M+2H]^{2+}$ m/z = 1095.1, found 1095.1; $[M+3H]^{3+}$ m/z = 730.4, found 730.2.

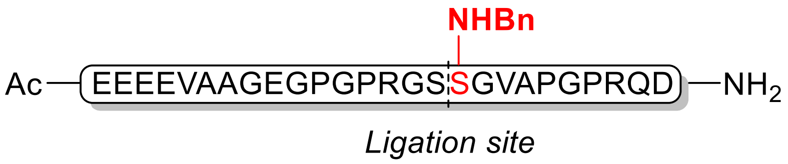

26

The ligation between Ac-EEEEVAAGEGPGPRGS-CO-SAL **S33** (2.92 mg, 1.67 μmol) and H-(AO,Bn)GVAPGPRQD-NH_2 **S21** (2 mg, 1.84 μmol) was performed as described in the general procedure for aminooxy ligation in aqueous buffer at pH 4.5 for overnight. Purification via preparative reverse phase HPLC (10-35% CH_3CN/H_2O over 45 min, 0.1% TFA) followed by lyophilization afforded Ac-EEEEVAAGEGPGPRGS(AO,Bn)GVAPGPRQD-NH_2 **26** (1.85 mg, 41.2% yield) as white solids.

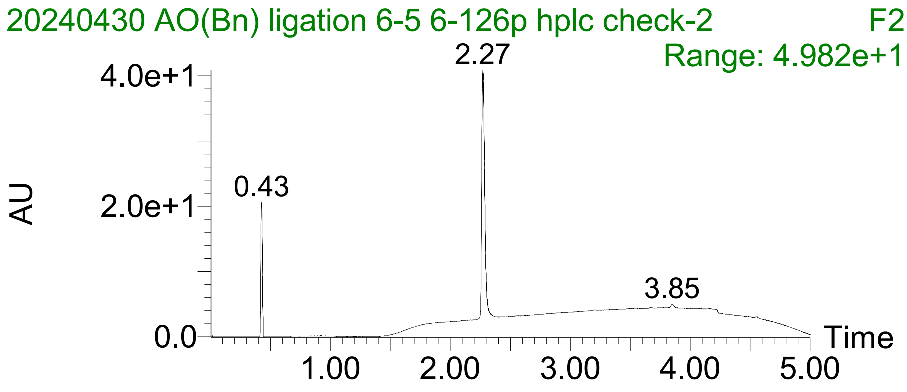

Figure S136. UV (190-400 nm) trace from UPLC-MS analysis of purified Ac-EEEEVAAGEGPGPRGS(AO,Bn)GVAPGPRQD-NH_2 gradient 5-95% CH_3CN/H_2O containing 0.1% TFA over 5 min at a flow rate of 0.4 mL/min.

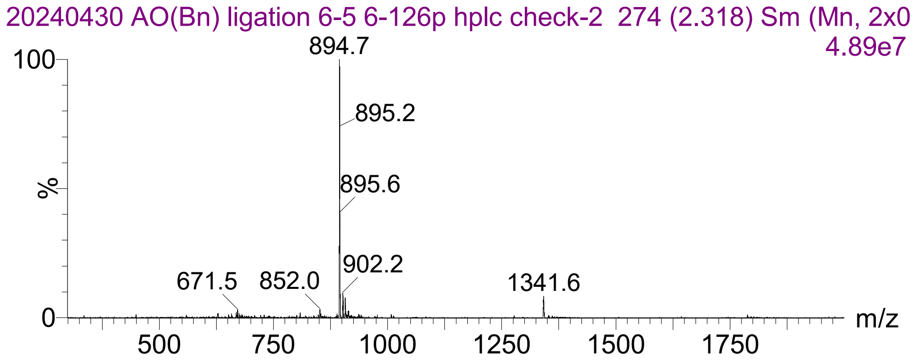

Figure S137. ESI-MS calcd. for $C_{112}H_{173}N_{35}O_{42}$ $[M+2H]^{2+}$ m/z = 1341.9, found 1341.6; $[M+3H]^{3+}$ m/z = 894.9, found 894.7; $[M+4H]^{4+}$ m/z = 671.5, found 671.5.

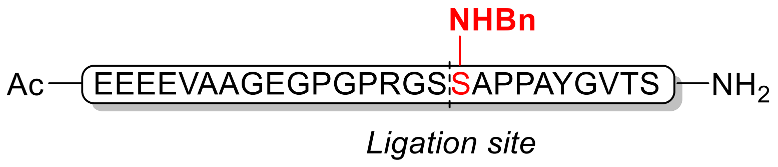

27

The ligation between Ac-EEEEVAAGEGPGPRGS-CO-SAL **S33** (2.62 mg, 1.5 μmol) and H-(AO,Bn)APPAYGVTS-NH_2 **S19** (1.74 mg, 1.65 μmol) was performed as described in the general procedure for aminooxy ligation in aqueous buffer at pH 4.5 for overnight. Purification via preparative reverse phase HPLC (10-40% CH_3CN/H_2O over 45 min, 0.1% TFA) followed by lyophilization afforded Ac-EEEEVAAGEGPGPRGS(AO,Bn)APPAYGVTS-NH_2 **27** (1.81 mg, 45.6% yield) as white solids.

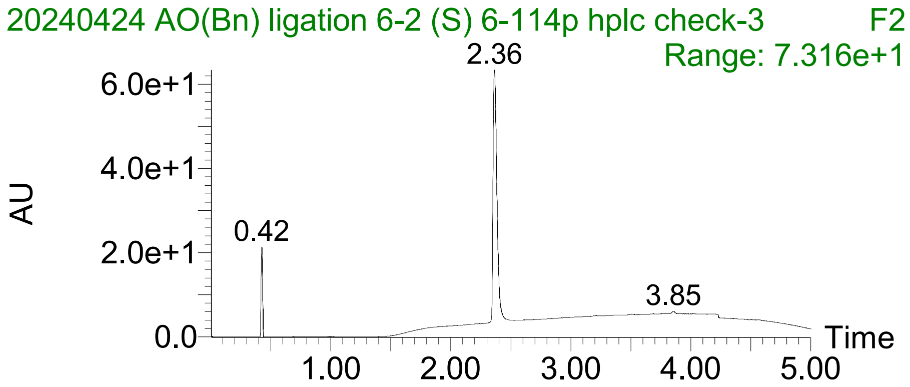

Figure S138. UV (190-400 nm) trace from UPLC-MS analysis of purified Ac-EEEEVAAGEGPGPRGS(AO,Bn)APPAYGVTS-NH$_2$ **27** gradient 5-95% CH_3CN/H_2O containing 0.1% TFA over 5 min at a flow rate of 0.4 mL/min.

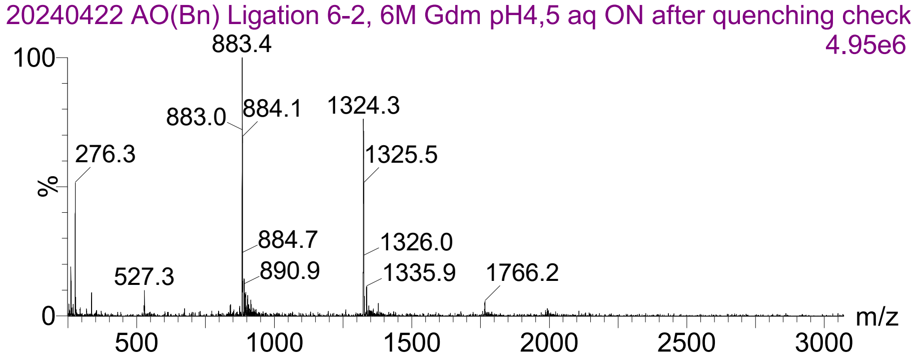

Figure S139. ESI-MS calcd. for $C_{114}H_{171}N_{31}O_{42}$ $[M+2H]^{2+}$ m/z = 1324.9, found 1324.3; $[M+3H]^{3+}$ m/z = 883.6, found 883.4.

5.1.4. Aminooxy glycosylation

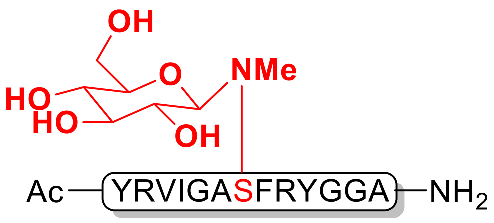

30

Ac-YRVIGA(AO, Me, Glc)FRYGGA-NH2 **30** was synthesized according to the general aminooxy glycosylation method. Ac-YRVIGA(AO, Me)FRYGGA-NH2 **12** (4 mg, 2.72 μmol) was incubated with 0.25 M glucose and 0.1 M $ZnCl_2$ in 6 M Guanidine buffer buffered with 0.2 M sodium citrate at pH 4 at 1 mM, room temperature for 96 h. The crude peptide was purified by preparative reverse-phase HPLC (10-40% CH_3CN/H_2O over 45 min) and lyophilized to afford the desired Glc peptide **30** (3.39 mg, 76.3% yield).

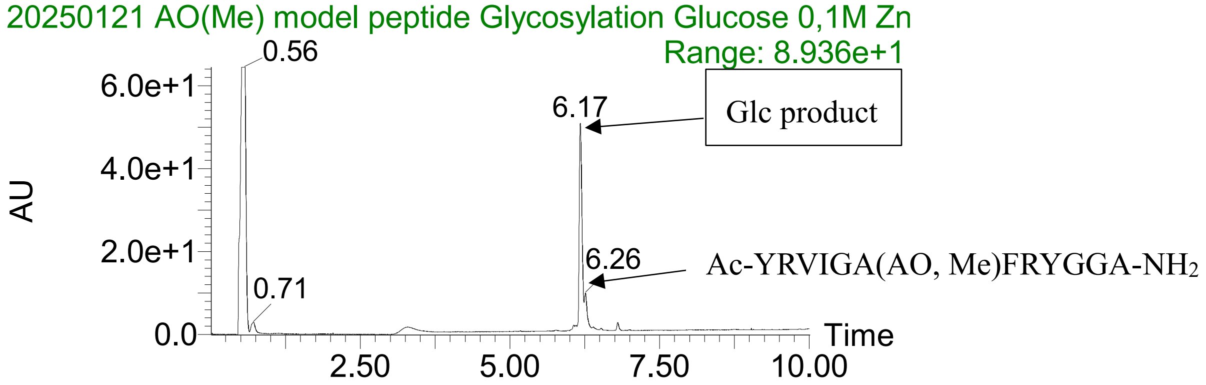

Figure S140. Representative UV (190-400 nm) trace from UPLC-MS analysis of synthesis of Ac-YRVIGA(AO, Me, Glc)FRYGGA-NH2 **30** gradient 10-40% CH_3CN/H_2O containing 0.1% TFA over 10 min at a flow rate of 0.4 mL/min.

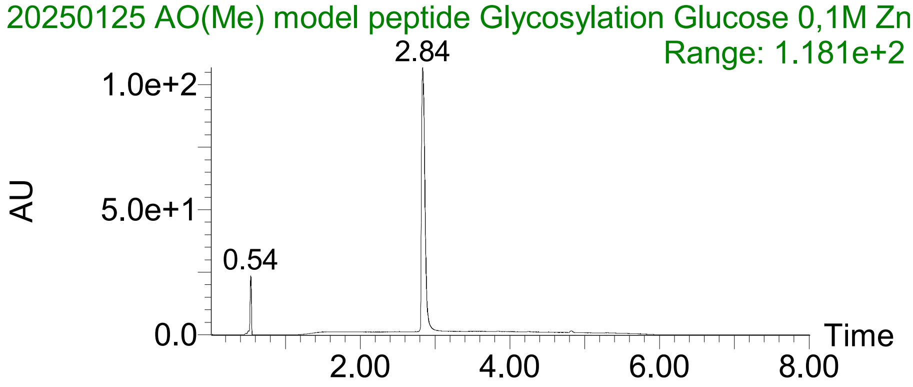

Figure S141. UV (190-400 nm) trace from UPLC-MS analysis of purified Ac-

YRVIGA(AO, Me, Glc)FRYGGA-NH_2 **30** gradient 5-95% CH_3CN/H_2O containing 0.1% TFA over 8 min at a flow rate of 0.4 mL/min.

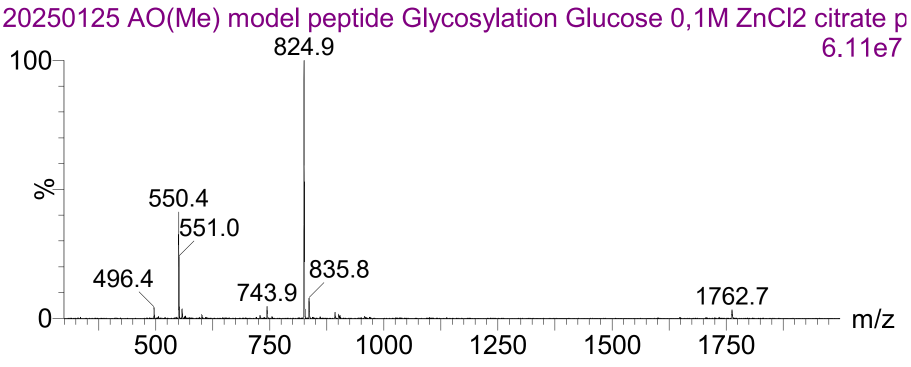

Figure S142. ESI-MS calcd. for $C_{75}H_{115}N_{21}O_{21}$ $[M+2H]^{2+}$ m/z = 825.4, found 824.9; $[M+3H]^{3+}$ m/z = 550.6, found 550.4.

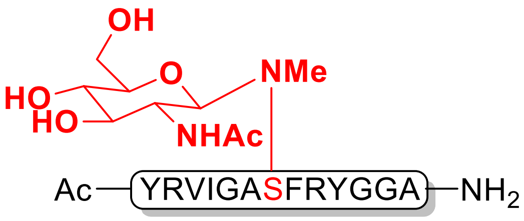

31

Ac-YRVIGA(AO, Me, GlcNAc)FRYGGA-NH_2 **31** was synthesized according to the general aminooxy glycosylation method. Ac-YRVIGA(AO, Me)FRYGGA-NH_2 **12** (2 mg, 1.36 μmol) was incubated with 0.25 M GlcNAc and 0.1 M $ZnCl_2$ in 6 M Guanidine buffer buffered with 0.2 M sodium citrate at pH 4 at 1 mM, room temperature for 96 h. The crude peptide was purified by preparative reverse-phase HPLC (10-40% CH_3CN/H_2O over 45 min) and lyophilized to afford the desired GlcNAc peptide **31** (1.59 mg, 69.8% yield).

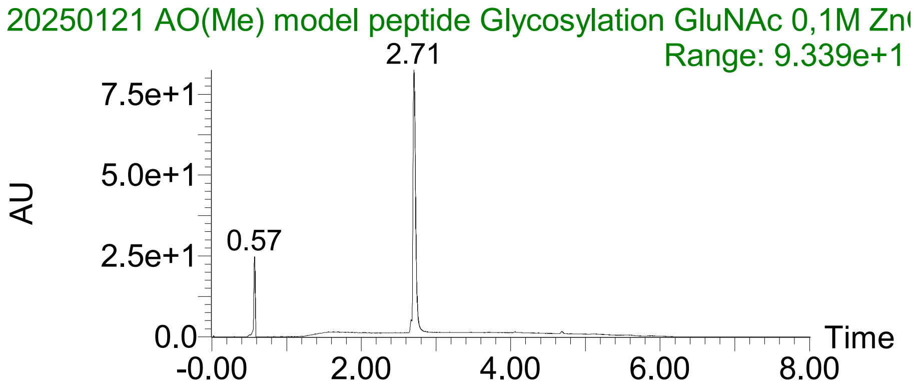

Figure S143. UV (190-400 nm) trace from UPLC-MS analysis of purified Ac-YRVIGA(AO, Me, GlcNAc)FRYGGA-NH_2 **31** gradient 5-95% CH_3CN/H_2O containing 0.1% TFA over 8 min at a flow rate of 0.4 mL/min.

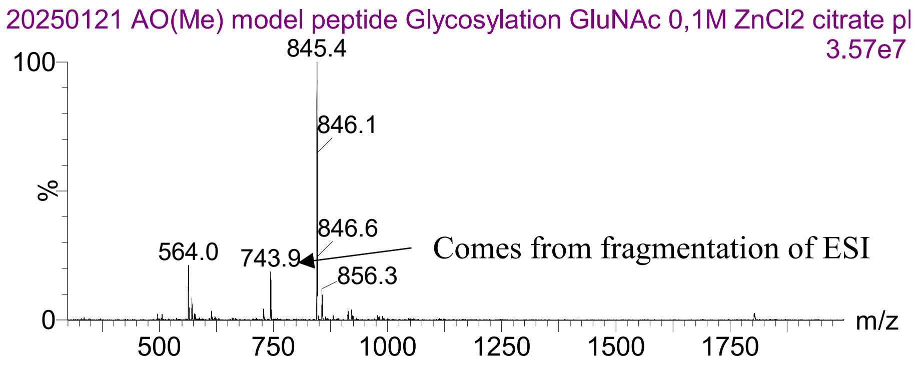

Figure S144. ESI-MS calcd. for $C_{77}H_{118}N_{22}O_{21}$ $[M+2H]^{2+}$ m/z = 846.0, found 845.4; $[M+3H]^{3+}$ m/z = 564.6, found 564.0.

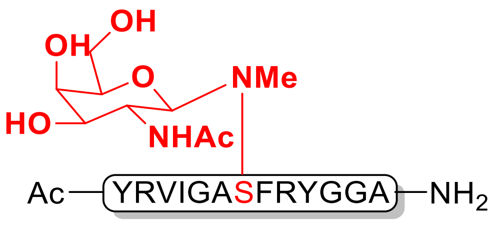

32

Ac-YRVIGA(AO, Me, GalNAc)FRYGGA-NH_2 **32** was synthesized according to the general aminooxy glycosylation method. Ac-YRVIGA(AO, Me)FRYGGA-NH_2 **12** (2 mg, 1.36 μmol) was incubated with 0.25 M GalNAc and 0.1 M $ZnCl_2$

in 6 M Guanidine buffer buffered with 0.2 M sodium citrate at pH 4 at 1 mM, room temperature for 96 h. The crude peptide was purified by preparative reverse-phase HPLC (10-40% CH_3CN/H_2O over 45 min) and lyophilized to afford the desired GalNAc peptide **32** (1.85 mg, 81.2% yield).

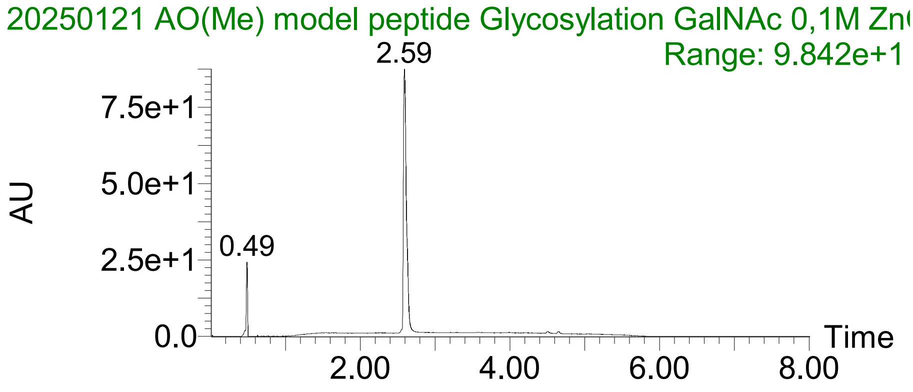

Figure S145. UV (190-400 nm) trace from UPLC-MS analysis of purified Ac-YRVIGA(AO, Me, GalNAc)FRYGGA-NH_2 **32** gradient 5-95% CH_3CN/H_2O containing 0.1% TFA over 8 min at a flow rate of 0.4 mL/min.

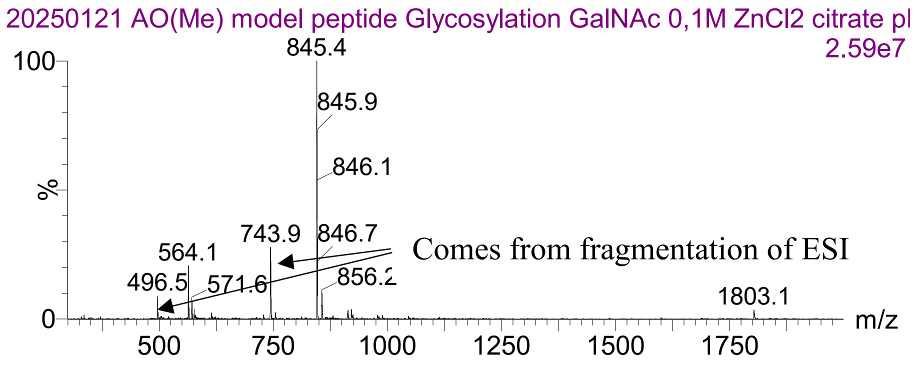

Figure S146. ESI-MS calcd. for $C_{77}H_{118}N_{22}O_{21}$ $[M+2H]^{2+}$ m/z = 846.0, found 845.4; $[M+3H]^{3+}$ m/z = 564.3, found 564.1.

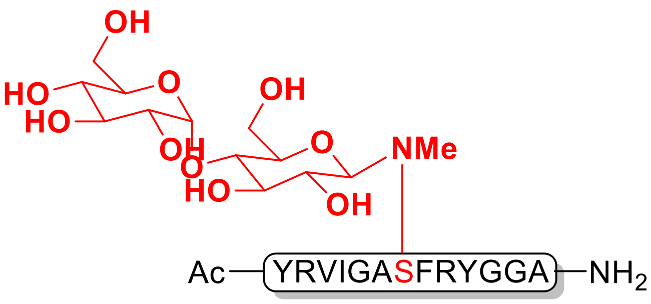

33

Ac-YRVIGA(AO, Me, Mal)FRYGGA-NH_2 **33** was synthesized according to the general aminooxy glycosylation method. Ac-YRVIGA(AO, Me)FRYGGA-NH_2 **12** (2 mg, 2.72 μmol) was incubated with 0.25 M Maltose and 0.1 M $ZnCl_2$ in 6 M Guanidine buffer buffered with 0.2 M sodium citrate at pH 4 at 1 mM, room temperature for 96 h. The crude peptide was purified by preparative reverse-phase HPLC (10-40% CH_3CN/H_2O over 45 min) and lyophilized to afford the desired Mal peptide **33** (1.52 mg, 61.6% yield).

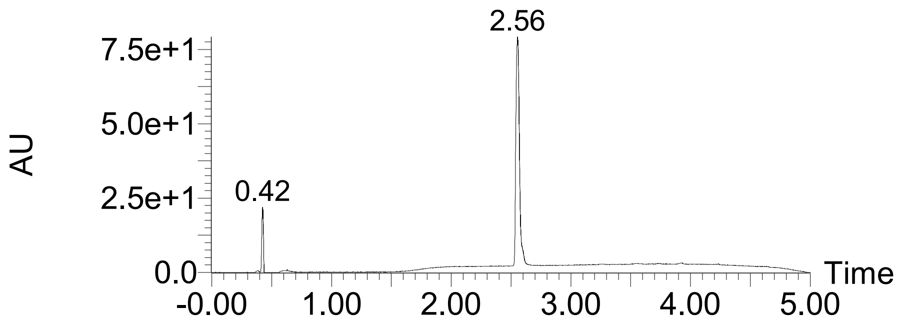

Figure S147. UV (190-400 nm) trace from UPLC-MS analysis of purified Ac-YRVIGA(AO, Me, Mal)FRYGGA-NH_2 **33** gradient 5-95% CH_3CN/H_2O containing 0.1% TFA over 5 min at a flow rate of 0.4 mL/min.

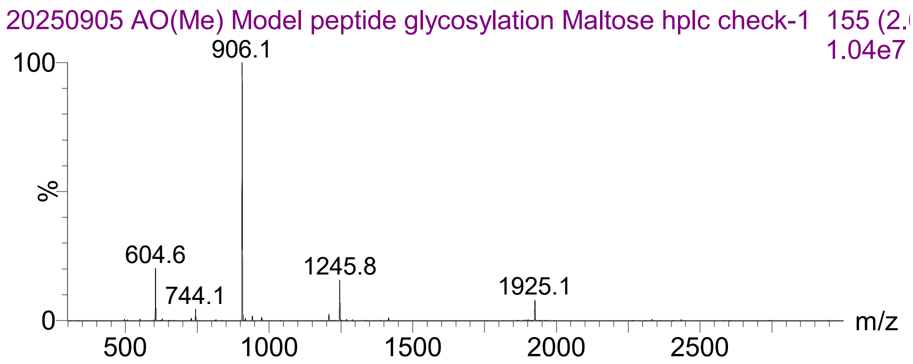

Figure S148. ESI-MS calcd. for $C_{80}H_{123}N_{21}O_{27}$ $[M+2H]^{2+}$ m/z = 906.5, found 906.1; $[M+3H]^{3+}$ m/z = 604.7, found 604.6.

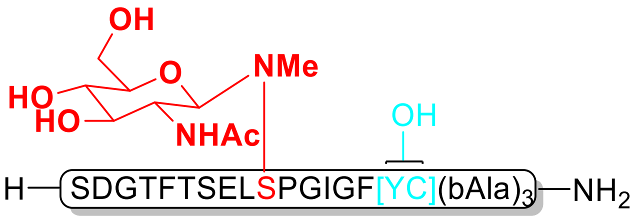

S34

H-SDGTFTSEL(AO, Me, GlcNAc)PGIGF[YC](βAla)$_3$-NH$_2$ **S34** was synthesized according to the general aminooxy glycosylation method. H-SDGTFTSEL(AO, Me)PGIGF[YC](βAla)$_3$-NH$_2$ **11** (2 mg, 0.928 μmol) was incubated with 0.25 M GlcNAc and 0.1 M $ZnCl_2$ in 6 M Guanidine buffer buffered with 0.2 M sodium citrate at pH 4 at 1 mM, room temperature for 96 h. The crude peptide was purified by preparative reverse-phase HPLC (20-45% CH_3CN/H_2O over 45 min) and lyophilized to afford the desired GlcNAc peptide **S34** (1.2 mg, 54.8% yield).

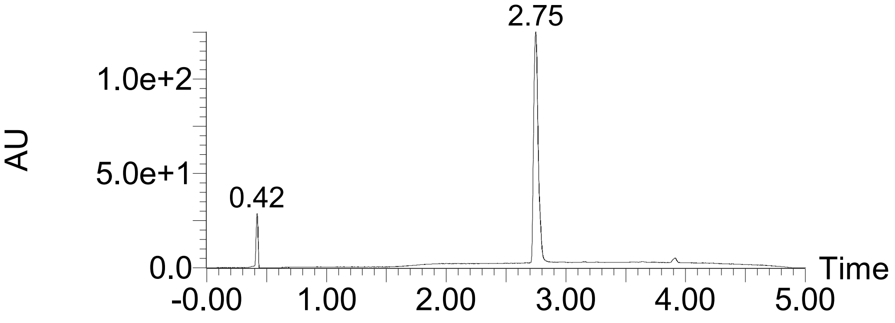

Figure S149. UV (190-400 nm) trace from UPLC-MS analysis of purified H-SDGTFTSEL(AO, Me, GlcNAc)PGIGF[YC](βAla)$_3$-NH$_2$ **S34** gradient 5-95% CH_3CN/H_2O containing 0.1% TFA over 5 min at a flow rate of 0.4 mL/min.

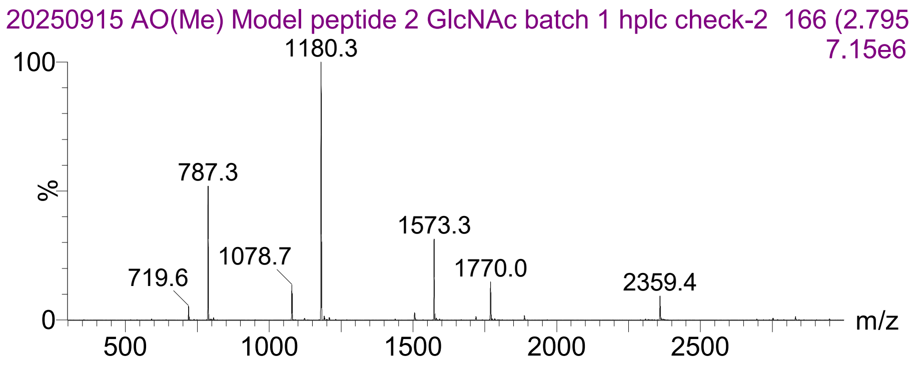

Figure S150. ESI-MS calcd. for $C_{105}H_{151}N_{23}O_{37}S$ $[M+H]^{+}$ m/z = 2360.6, found 2359.4; $[M+2H]^{2+}$ m/z = 1180.8, found 1180.3; $[M+3H]^{3+}$ m/z = 787.5, found 787.3.

5.1.5. Applications of AOL & CEL in peptide drug synthesis and modification.

5.1.5.1. Synthesis of Sermorelin analogue **34** and **35**

Synthesis of Sermorelin (1-17) SAL ester **S35**

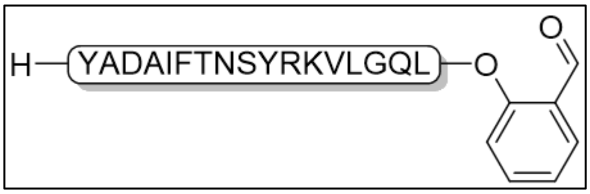

S35

Sermoerlin (1-17) SAL ester **S35** was synthesized according to the general SAL ester preparation method at 0.042 mmol scale. The crude peptide was purified by preparative reverse-phase HPLC (25-60% CH_3CN/H_2O over 45 min) and lyophilized to afford the desired SAL ester **S35** (42.5 mg, 49% yield).

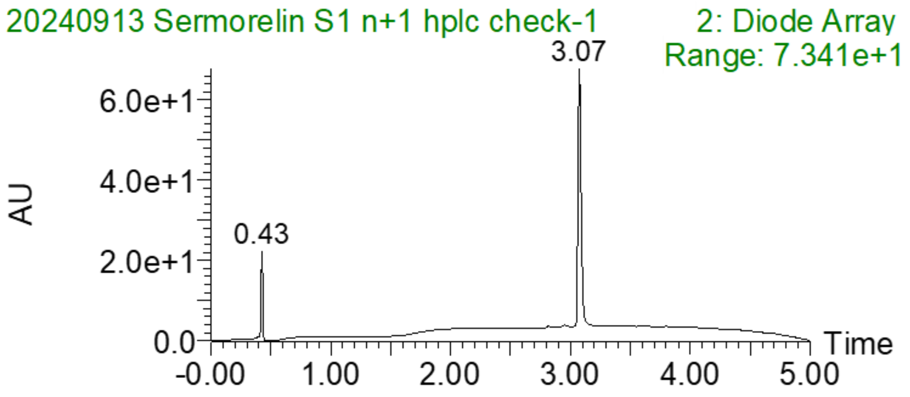

Figure S151. UV (190-400 nm) trace from UPLC-MS analysis of purified Sermorelin (1-17) SAL ester **S35** gradient 5-95% CH_3CN/H_2O containing 0.1% TFA over 5 min at a flow rate of 0.4 mL/min.

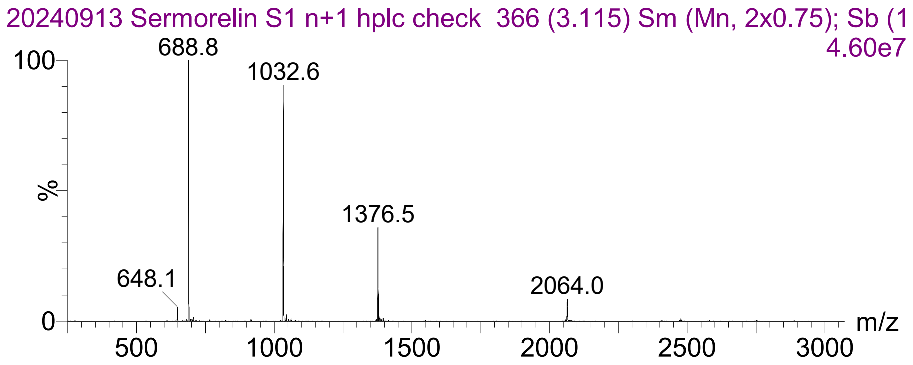

Figure S152. ESI-MS calcd. for $C_{97}H_{143}N_{23}O_{27}$ $[M+H]^+$ m/z = 2064.4, found 2064.0; $[M+2H]^{2+}$ m/z = 1033.2, found 1032.7; $[M+3H]^{3+}$ m/z = 689.1, found 688.8.

Synthesis of Sermorelin (18-29, AO) with N-terminal AO(Bn) **S36**

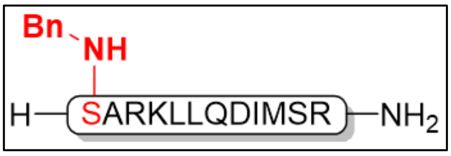

S36

Sermorelin (18-29, AO) with N-terminal AO(Bn) **S36** was synthesized according to the standard protocol of SPPS at 0.05 mmol scale. The crude peptide was purified by preparative reverse-phase HPLC (15-45% CH_3CN/H_2O over 45 min) and lyophilized to afford the desired peptide **S36** (42.7 mg, 56.2% yield).

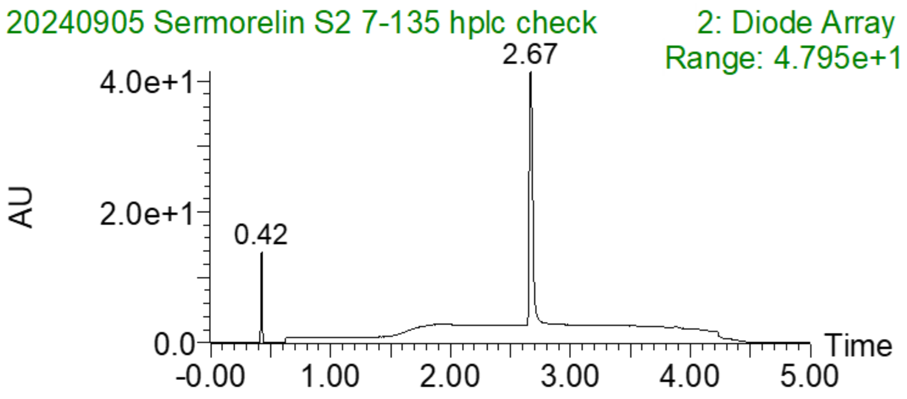

Figure S153. UV (190-400 nm) trace from UPLC-MS analysis of purified Sermorelin (18-29, AO) with N-terminal AO(Bn) **S36** gradient 5-95% CH_3CN/H_2O containing 0.1% TFA over 5 min at a flow rate of 0.4 mL/min.

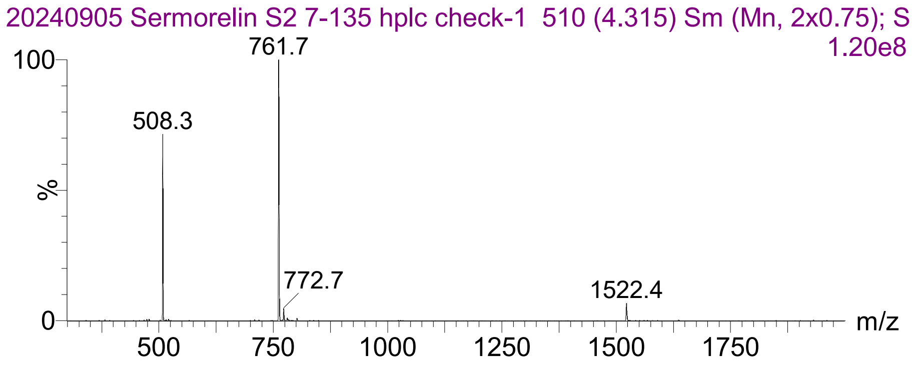

Figure S154. ESI-MS calcd. for $C_{66}H_{116}N_{22}O_{17}S$ $[M+H]^+$ m/z = 1521.9, found 1522.4; $[M+2H]^{2+}$ m/z = 762.0, found 761.7; $[M+3H]^{3+}$ m/z = 508.3, found

508.3.

Synthesis of Sermorelin (1-29, AO) ***S37*** *by aminooxy ligation*

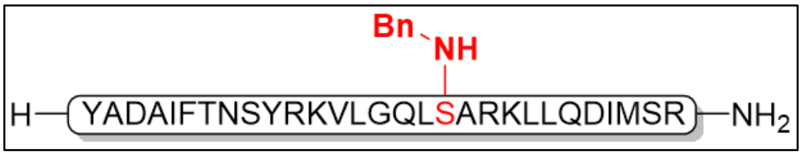

S37

The ligation between Sermorelin (1-17, AO) SAL ester **S35** (5 mg, 2.42 μmol) and Sermorelin (18-29, AO) **S36** (7.4 mg, 4.85 μmol) was performed as described in the general procedure for aminooxy ligation in Pyridine/Acetic acid 1:3 at 20 mM for overnight. Purification via preparative reverse phase HPLC (25-50% CH_3CN/H_2O over 45 min, 0.1% TFA) followed by lyophilization afforded Sermorelin (1-29, AO) **S37** (3.61 mg, 43.0% yield) as white solids.

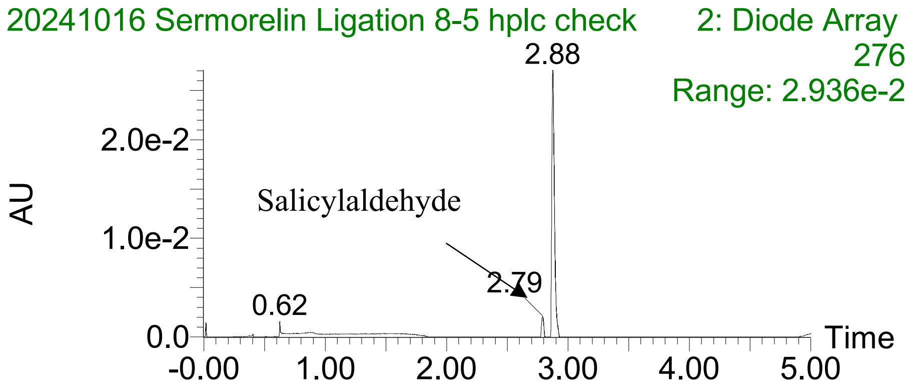

Figure S155. UV (190-400 nm) trace from UPLC-MS analysis of purified Sermorelin (1-29, AO) **S37** gradient 5-95% CH_3CN/H_2O containing 0.1% TFA over 5 min at a flow rate of 0.4 mL/min.

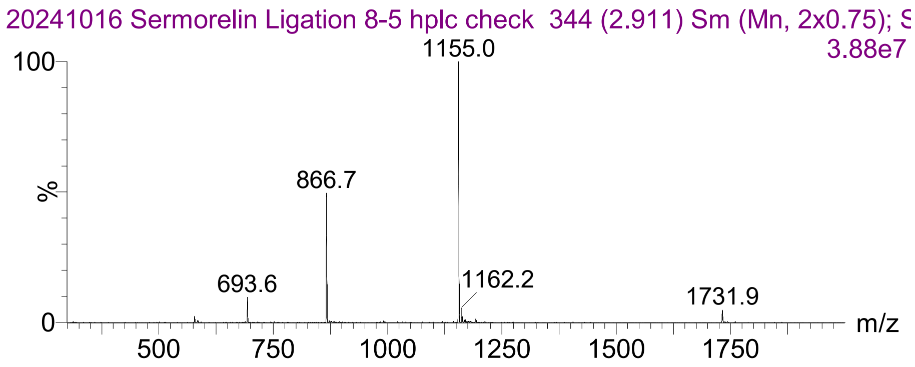

Figure S156. ESI-MS calcd. for $C_{156}H_{253}N_{45}O_{42}S$ $[M+2H]^{2+}$ m/z = 1732.5, found 1731.9; $[M+3H]^{3+}$ m/z = 1155.4, found 1155.0; $[M+4H]^{4+}$ m/z = 866.8, found 866.7; $[M+5H]^{5+}$ m/z = 693.6, found 693.6.

*Sermorelin (1-29) analogue **34** by CEL*

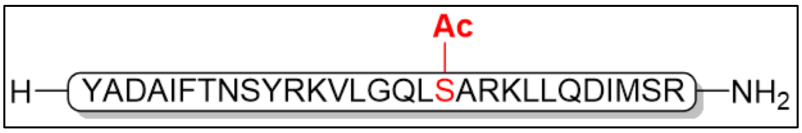

34

Sermorelin analogue **34** was synthesized according to the general CEL method. Sermorelin (1-29, AO) **S37** (3 mg, 0.867 μmol) was incubated with pyruvic acid (0.31 μL, 4.33 μmol) in aqueous DMSO with 0.01M oxalic acid at 40 mM, 60°C for 5 h. The crude peptide was purified by preparative reverse-phase HPLC (25-45% CH_3CN/H_2O over 45 min) and lyophilized to afford the desired **34** (1.30 mg, 44.1% yield).

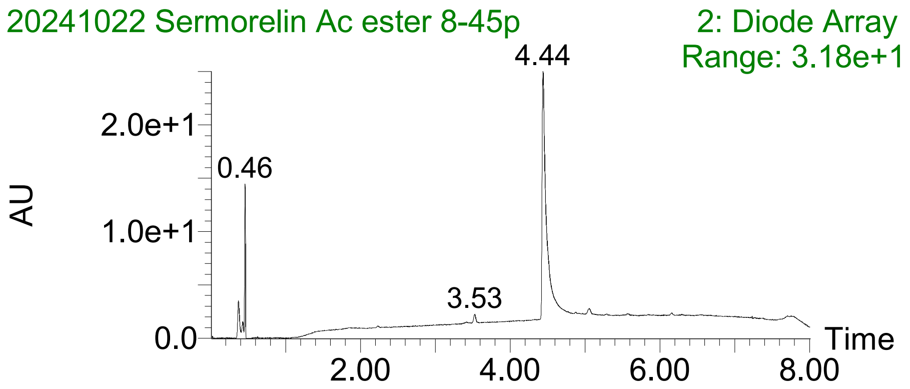

Figure S157. UV (190-400 nm) trace from UPLC-MS analysis of purified Sermorelin analogue **34** gradient 5-75% CH_3CN/H_2O containing 0.1% TFA over 8 min at a flow rate of 0.4 mL/min.

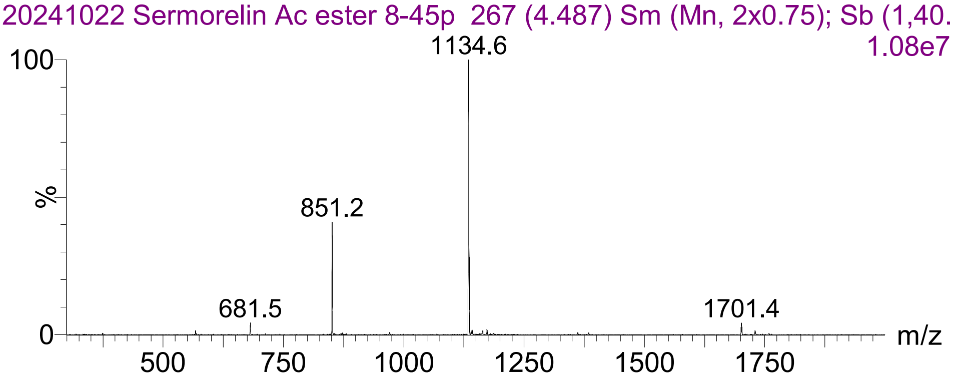

Figure S158. ESI-MS calcd. for $C_{151}H_{248}N_{44}O_{43}S$ $[M+2H]^{2+}$ m/z = 1701.0, found 1701.4; $[M+3H]^{3+}$ m/z = 1134.3, found 1143.6; $[M+4H]^{4+}$ m/z = 851.0, found 851.2; $[M+5H]^{5+}$ m/z = 681.0, found 681.5.

*Synthesis of Sermorelin (1-29) analogue **35** by CEL*

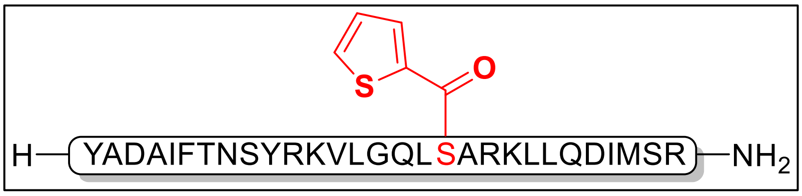

35

Sermorelin (1-29) analogue **35** was synthesized according to the general CEL method. Sermorelin (1-29, AO) **S37** (1 mg, 0.289 μmol) was incubated with 2-Oxo-2-(thiophen-2-yl) acetic acid (0.45 mg, 2.89 μmol) in aqueous DMSO with 0.1M oxalic acid at 40 mM, 60°C for overnight. The crude peptide was purified by preparative reverse-phase HPLC (25-50% CH_3CN/H_2O over 45 min) and lyophilized to afford the desired thiazole ester **35** (0.19 mg, 19.0% yield).

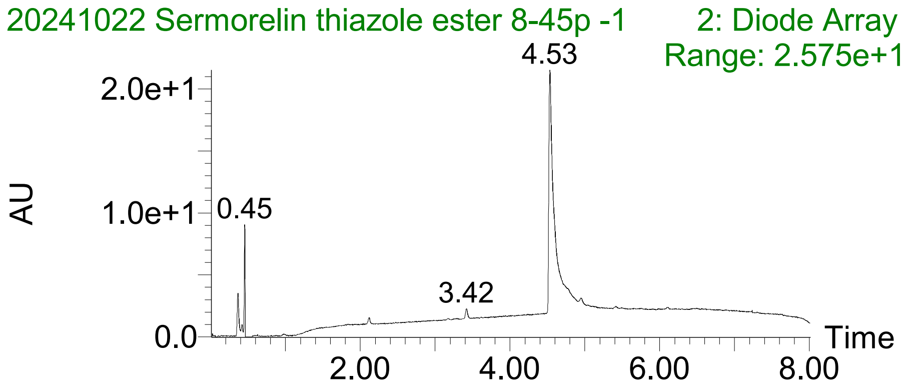

Figure S159. UV (190-400 nm) trace from UPLC-MS analysis of purified Sermorelin (1-29, thiazole ester) **35** gradient 5-75% CH_3CN/H_2O containing 0.1% TFA over 8 min at a flow rate of 0.4 mL/min.

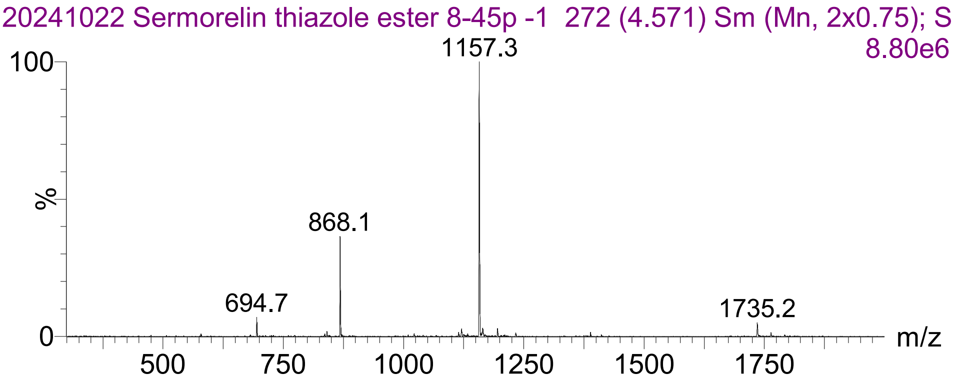

Figure S160. ESI-MS calcd. for $C_{154}H_{248}N_{44}O_{43}S_2$ $[M+2H]^{2+}$ m/z = 1735.3, found 1735.2; $[M+3H]^{3+}$ m/z = 1157.2, found 1157.3; $[M+4H]^{4+}$ m/z = 868.2, found 868.1; $[M+5H]^{5+}$ m/z = 694.7, found 694.7.

5.1.5.2. Synthesis of Somatorelin analogue **36**

Synthesis of Somatorelin (1-17) SAL ester **S38**

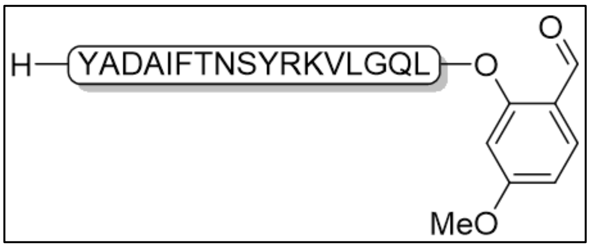

Somatorelin (1-17) SAL ester **S38** was synthesized according to the general

SAL ester preparation method at 0.05 mmol scale. The crude peptide was purified by preparative reverse-phase HPLC (10-50% CH_3CN/H_2O over 30 min) and lyophilized to afford the desired SAL ester **S38** (31.1 mg, 29.7% yield).

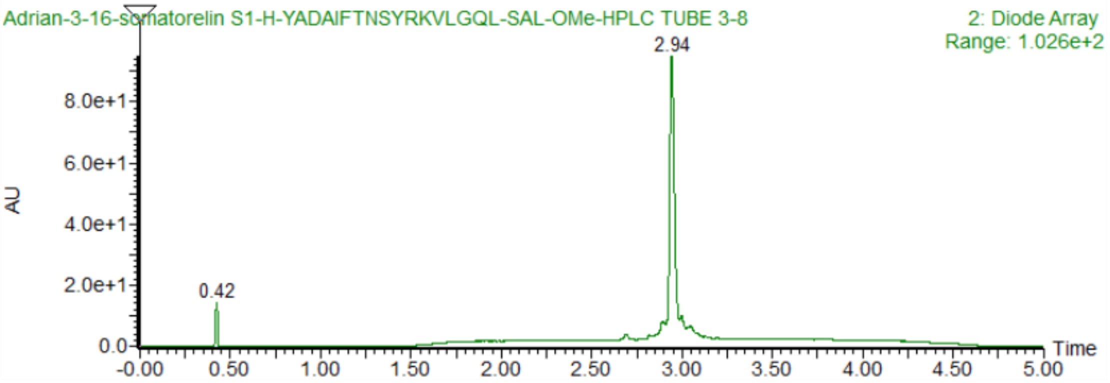

Figure S161. UV (190-400 nm) trace from UPLC-MS analysis of purified Somatorelin (1-17) SAL ester **S38** gradient 5-95% CH_3CN/H_2O containing 0.1% TFA over 5 min at a flow rate of 0.4 mL/min.

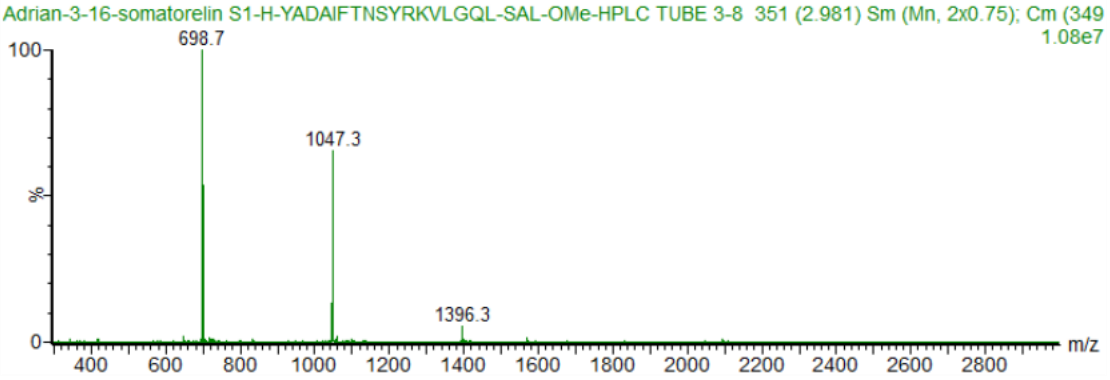

Figure S162. ESI-MS calcd. for $C_{98}H_{145}N_{23}O_{28}$ $[2M+3H]^{3+}$ m/z = 1396.6, found 1396.3; $[M+2H]^{2+}$ m/z = 1047.9, found 1047.3; $[M+3H]^{3+}$ m/z = 698.9, found 698.7.

Synthesis of Somatorelin (21-44, AO) with N-terminal AO(Bn) **S39**

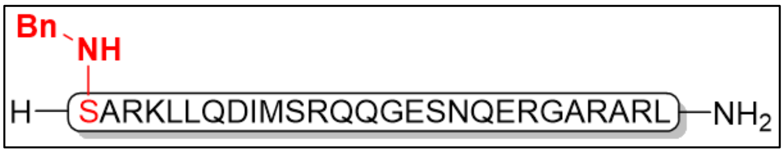

S39

Somatorelin (21-44, AO) with N-terminal AO(Bn) **S39** was synthesized according to the standard protocol of SPPS at 0.05 mmol scale. The crude peptide was purified by preparative reverse-phase HPLC (10-45% CH_3CN/H_2O over 45 min) and lyophilized to afford the desired peptide **S39** (75.8 mg, 47.3% yield).

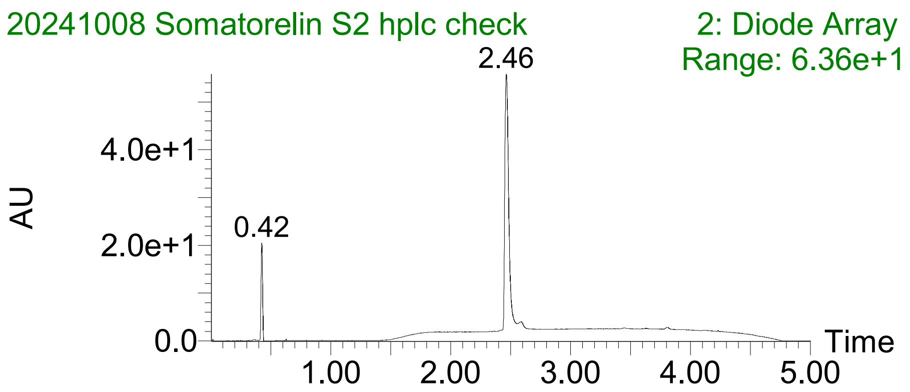

Figure S163. UV (190-400 nm) trace from UPLC-MS analysis of purified Somatorelin (21-44, AO) with N-terminal AO(Bn) **S39** gradient 5-95% CH_3CN/H_2O containing 0.1% TFA over 5 min at a flow rate of 0.4 mL/min.

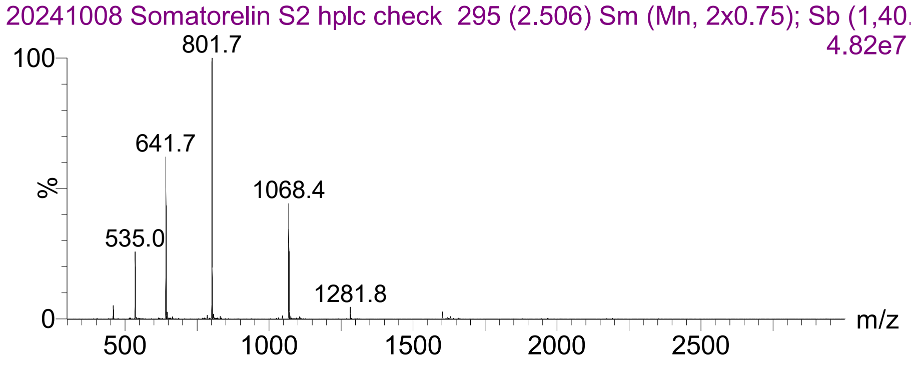

Figure S164. ESI-MS calcd. for $C_{132}H_{227}N_{49}O_{42}S$ $[M+3H]^{3+}$ m/z = 1069.2 found 1068.4; $[M+4H]^{4+}$ m/z = 802.2, found 801.7; $[M+5H]^{5+}$ m/z = 641.9, found 641.7; $[M+6H]^{6+}$ m/z = 535.1, found 535.0.

Synthesis of Somatorelin (1-44, AO) **S40**

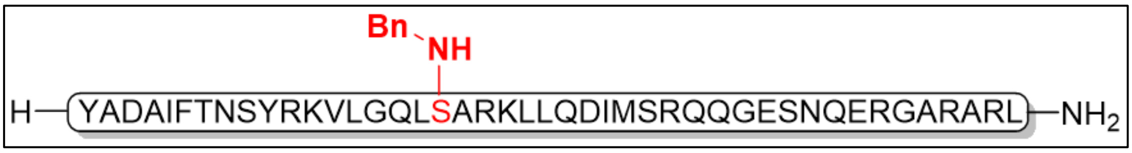

S40

The ligation between Somatorelin (1-17) SAL ester **S38** (6 mg, 2.87 μmol) and Somatorelin (21-44, AO) **S39** (11.1 mg, 3.44 μmol) was performed as described in the general procedure for aminooxy ligation in aqueous buffer at pH 4.5 for overnight. Purification via preparative reverse phase HPLC (20-50% CH_3CN/H_2O over 45 min, 0.1% TFA) followed by lyophilization afforded Somatorelin (1-44, AO) **S40** (3.51 mg, 23.8% yield) as white solids.

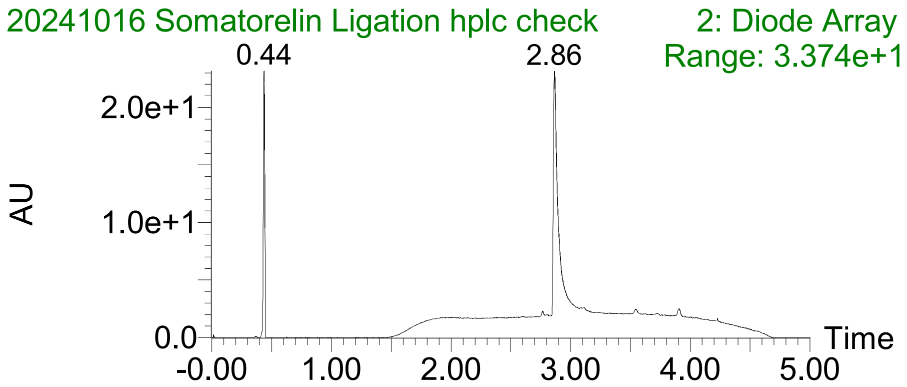

Figure S165. UV (190-400 nm) trace from UPLC-MS analysis of purified Somatorelin (1-44, AO) **S40** gradient 5-95% CH_3CN/H_2O containing 0.1% TFA over 5 min at a flow rate of 0.4 mL/min.

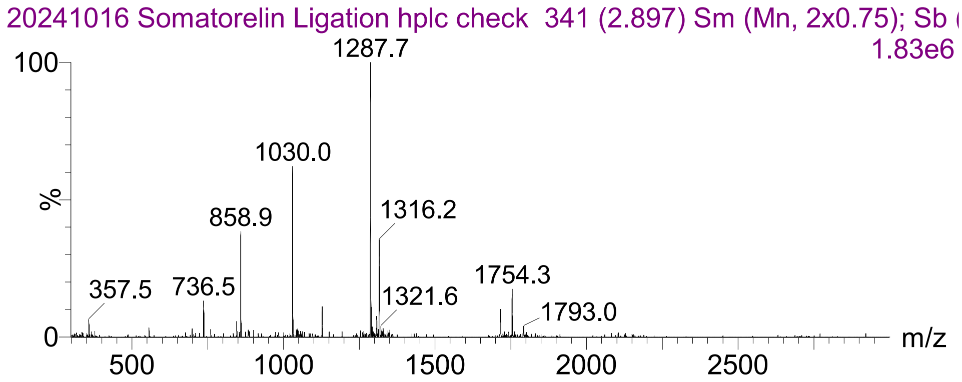

Figure S166. ESI-MS calcd. for $C_{222}H_{364}N_{72}O_{67}S$ $[M+3H]^{3+}$ m/z = 1716.3 found 1716.6; $[M+4H]^{4+}$ m/z = 1287.5, found 1287.7; $[M+5H]^{5+}$ m/z = 1030.2, found 1030.0; $[M+6H]^{6+}$ m/z = 858.6, found 858.9; $[M+7H]^{7+}$ m/z = 736.1, found 736.5.

Synthesis of Somatorelin analogue ***36*** *by CEL modification*

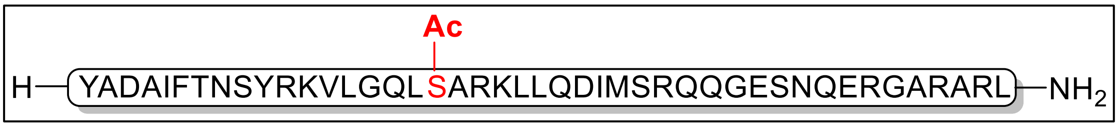

36

Somatorelin (1-44, Ac ester) **36** was synthesized according to the general CEL method. Somatorelin (1-44, AO) (2.3 mg, 0.447 μmol) was incubated with pyruvic acid (0.157 μL, 2.24 μmol) in aqueous DMSO with 0.01M oxalic acid and 0.1M DMS at 40 mM, 60°C for 5 h. The crude peptide was purified by preparative reverse-phase HPLC (20-45% CH_3CN/H_2O over 45 min) and lyophilized to afford the desired Ac ester **36** (0.98 mg, 43.1% yield).

Ac ester product

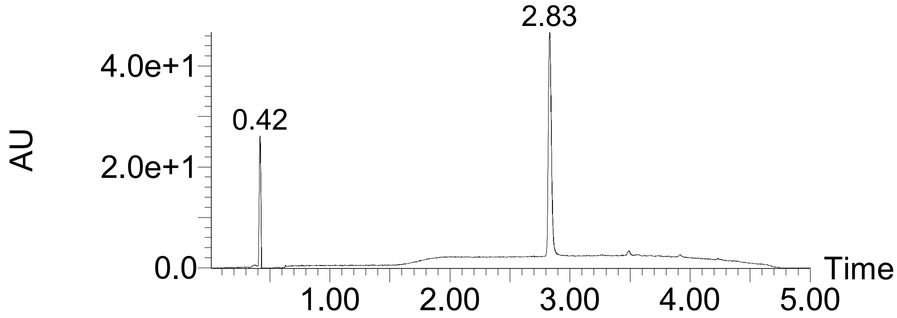

Figure S167. UV (190-400 nm) trace from UPLC-MS analysis of purified Somatorelin (1-44, Ac ester) **36** gradient 5-95% CH_3CN/H_2O containing 0.1% TFA over 5 min at a flow rate of 0.4 mL/min.

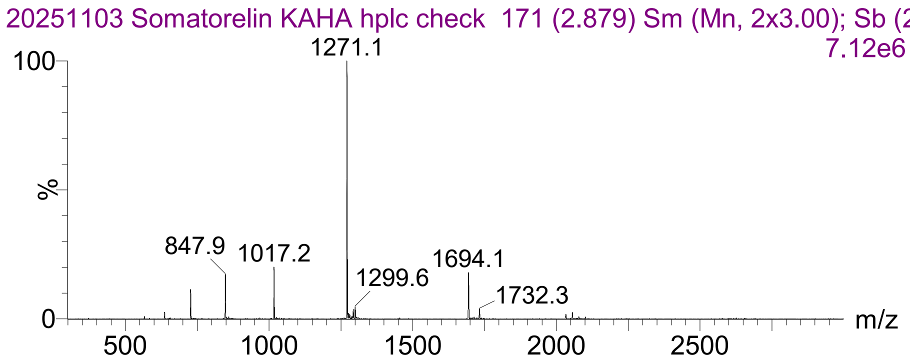

Figure S168. ESI-MS calcd. for $C_{217}H_{359}N_{71}O_{68}S$ $[M+3H]^{3+}$ m/z = 1695.2 found 1694.1; $[M+4H]^{4+}$ m/z = 1271.7, found 1271.1; $[M+5H]^{5+}$ m/z = 1017.5, found 1017.2; $[M+6H]^{6+}$ m/z = 848.1, found 847.9; $[M+7H]^{7+}$ m/z = 727.1, found 726.9.

5.1.5.3. Synthesis of Secretin analogue **37**

Synthesis of Secretin (1-9) SAL ester **S41**

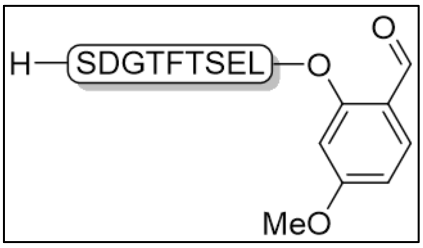

S41

Secretin (1-9) SAL ester **S41** was synthesized according to the general SAL ester preparation method at 0.135 mmol scale. The crude peptide was purified by preparative reverse-phase HPLC (25-50% CH_3CN/H_2O over 45 min) and lyophilized to afford the desired SAL ester **S41** (63.3 mg, 43.0% yield).

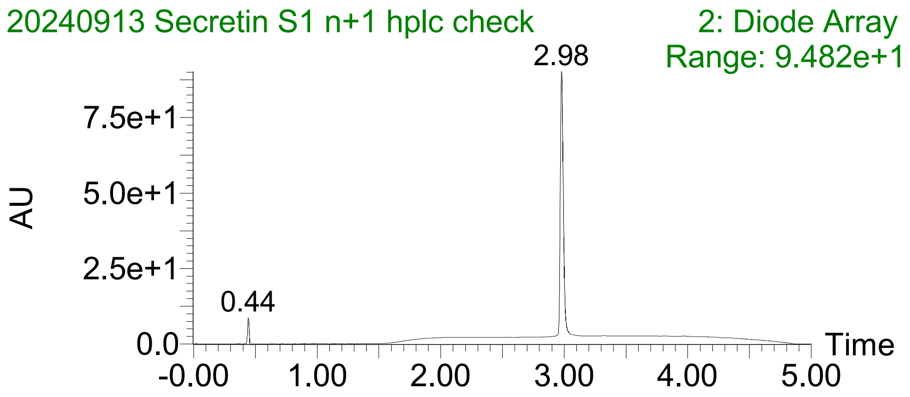

Figure S169. UV (190-400 nm) trace from UPLC-MS analysis of purified Secretin (1-9) SAL ester **S41** gradient 5-95% CH_3CN/H_2O containing 0.1% TFA over 5 min at a flow rate of 0.4 mL/min.

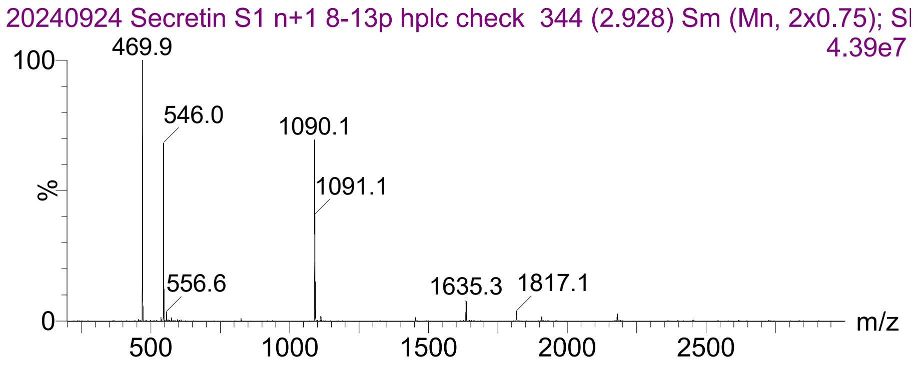

Figure S170. ESI-MS calcd. for $C_{48}H_{67}N_9O_{20}$ $[M+H]^+$ m/z = 1091.1, found 1090.1; $[M+2H]^{2+}$ m/z = 546.0, found 545.9.

Synthesis of Secretin (10-26, AO) with N-terminal AO(Bn) **S42**

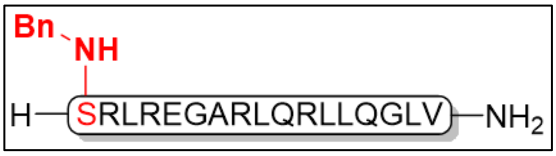

S42

Secretin (10-26, AO) with N-terminal AO(Bn) **S42** was synthesized according to the standard protocol of SPPS at 0.05 mmol scale. The crude peptide was purified by preparative reverse-phase HPLC (20-50% CH_3CN/H_2O over 45 min) and lyophilized to afford the desired peptide **S42** (64.2 mg, 62.1% yield).

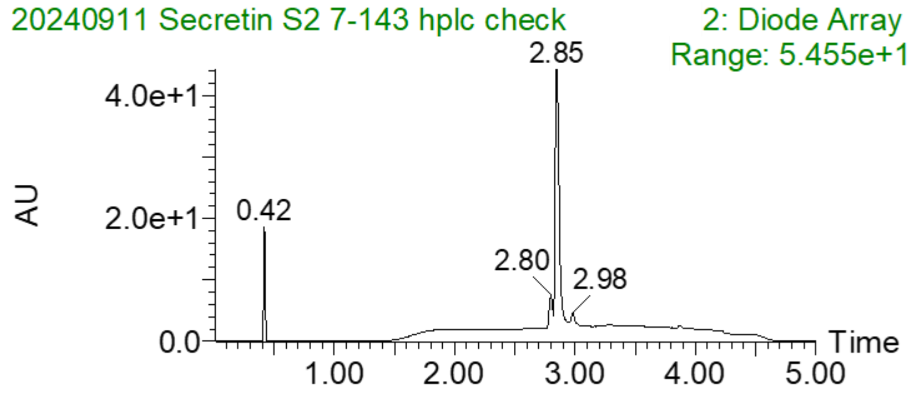

Figure S171. UV (190-400 nm) trace from UPLC-MS analysis of purified Secretin (10-26, AO) **S42** gradient 5-95% CH_3CN/H_2O containing 0.1% TFA over 5 min at a flow rate of 0.4 mL/min.

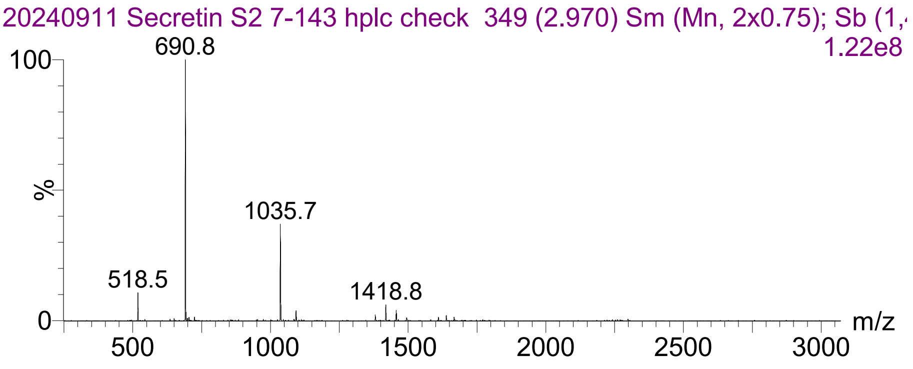

Figure S172. ESI-MS calcd. for $C_{91}H_{161}N_{33}O_{22}$ $[M+2H]^{2+}$ m/z = 1035.7, found 1035.7; $[M+3H]^{3+}$ m/z = 690.8, found 690.8; $[M+4H]^{4+}$ m/z = 518.4, found

518.5.

Synthesis of Secretin (1-26, AO) **S43**

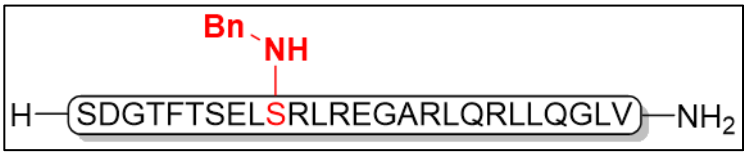

S43

The ligation between Secretin (1-9) SAL ester **S41** (5.8 mg, 5.32 μmol) and Secretin (10-26, AO) **S42** (16.5 mg, 7.98 μmol) was performed as described in the general procedure for aminooxy ligation in aqueous buffer at pH 4.5 for overnight. Purification via preparative reverse phase HPLC (25-50% CH_3CN/H_2O over 45 min, 0.1% TFA) followed by lyophilization afforded Secretin (1-26, AO) **S43** (8.03 mg, 50.2% yield) as white solids.

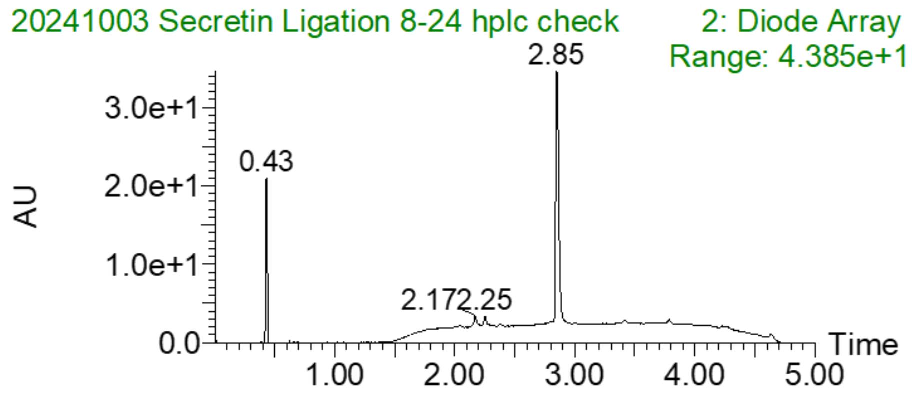

Figure S173. UV (190-400 nm) trace from UPLC-MS analysis of purified Secretin (1-26, AO) **S43** gradient 5-95% CH_3CN/H_2O containing 0.1% TFA over 5 min at a flow rate of 0.4 mL/min.

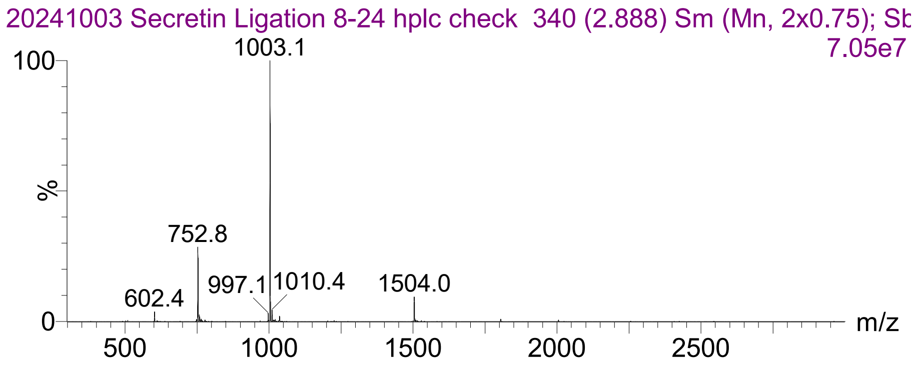

Figure S174. ESI-MS calcd. for $C_{131}H_{220}N_{42}O_{39}$ $[M+2H]^{2+}$ m/z = 1504.7, found 1503.9; $[M+3H]^{3+}$ m/z = 1003.5, found 1003.1; $[M+4H]^{4+}$ m/z = 752.9, found 752.7.

Synthesis of Secretin analogue ***37*** *by CEL*

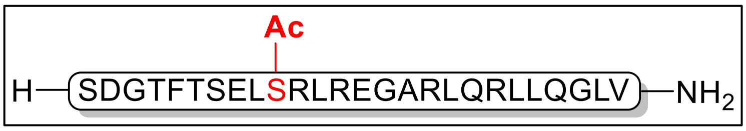

37

Secretin analogue **37** was synthesized according to the general CEL method. Secretin (1-26, AO) **S43** (5 mg, 1.66 μmol) was incubated with pyruvic acid (0.59 μL, 8.32 μmol) in aqueous DMSO with 0.1M oxalic acid at 40 mM, 60°C for 5 h. The crude peptide was purified by preparative reverse-phase HPLC (25-45% CH_3CN/H_2O over 45 min) and lyophilized to afford the desired Ac ester **37** (2.67 mg, 54.5% yield).

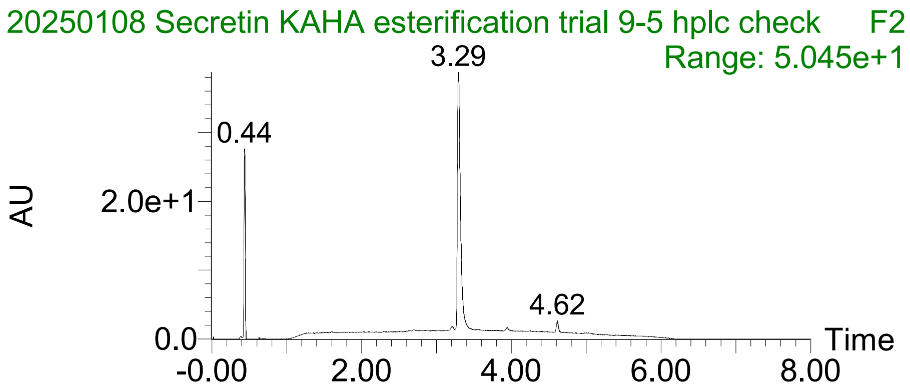

Figure S175. UV (190-400 nm) trace from UPLC-MS analysis of purified **37** gradient 5-95% CH_3CN/H_2O containing 0.1% TFA over 8 min at a flow rate of 0.4 mL/min.

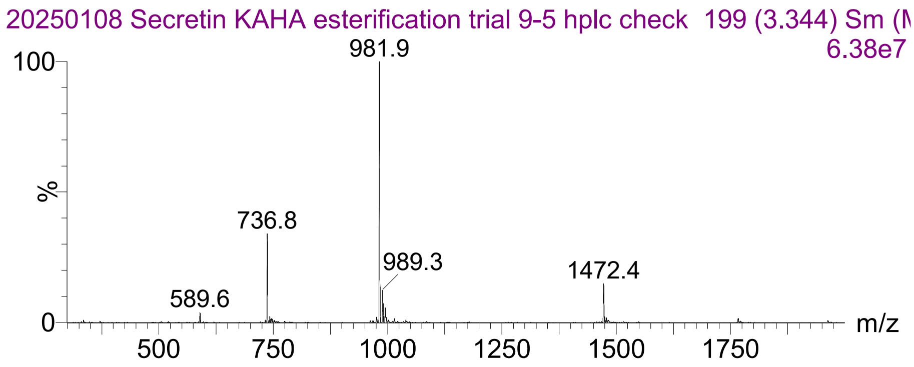

Figure S176. ESI-MS calcd. for $C_{126}H_{215}N_{41}O_{40}$ $[M+2H]^{2+}$ m/z = 1473.2, found 1472.4; $[M+3H]^{3+}$ m/z = 982.5, found 981.9; $[M+4H]^{4+}$ m/z = 737.1, found 736.8; $[M+5H]^{5+}$ m/z = 589.9, found 589.6.

5.1.5.4. Synthesis of GLP-1 analogue **38**

Synthesis of GLP-1 (1-11) SAL ester **S44**

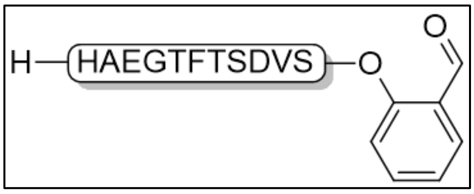

GLP-1 (1-11) SAL ester **S44** was synthesized according to the general SAL ester preparation method at 0.1 mmol scale. The crude peptide was purified by preparative reverse-phase HPLC (10-40% CH_3CN/H_2O over 45 min) and lyophilized to afford the desired SAL ester **S44** (44.4 mg, 35.4% yield).

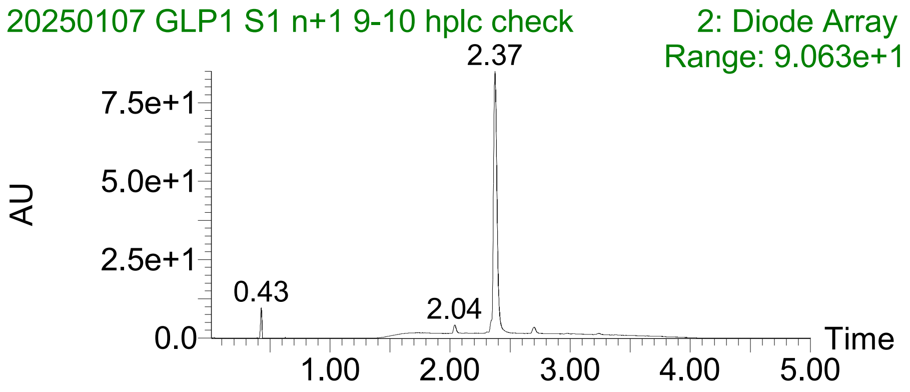

Figure S177. UV (190-400 nm) trace from UPLC-MS analysis of purified

GLP-1 (1-11) SAL ester **S44** gradient 5-95% CH_3CN/H_2O containing 0.1% TFA over 5 min at a flow rate of 0.4 mL/min.

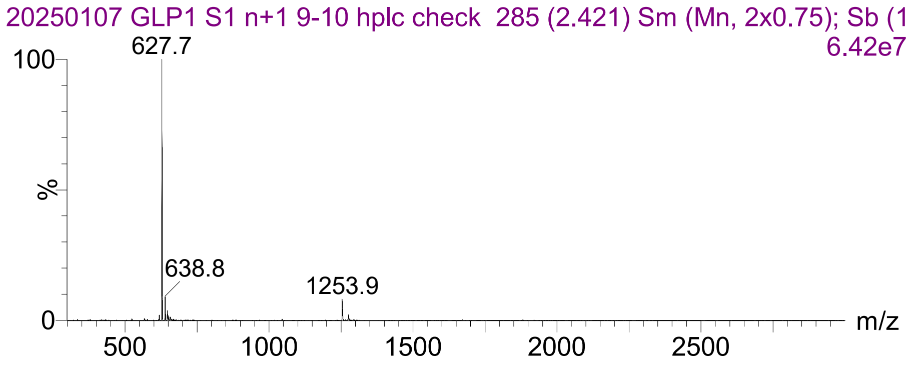

Figure S178. ESI-MS calcd. for $C_{55}H_{75}N_{13}O_{21}$ $[M+H]^+$ m/z = 1255.3 found 1253.9; $[M+2H]^{2+}$ m/z = 627.6, found 627.7.

Synthesis of GLP-1 (12-31, AO) with N-terminal AO(Bn) **S45**

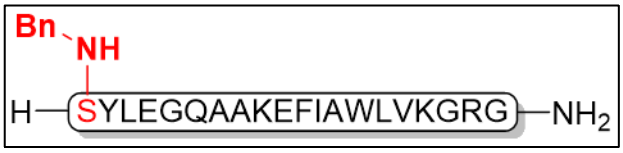

GLP-1 (12-31, AO) with N-terminal AO(Bn) **S45** was synthesized according to the standard protocol of SPPS at 0.05 mmol scale. The crude peptide was purified by preparative reverse-phase HPLC (25-60% CH_3CN/H_2O over 45 min) and lyophilized to afford the desired peptide **S45** (48.0 mg, 41.2% yield).

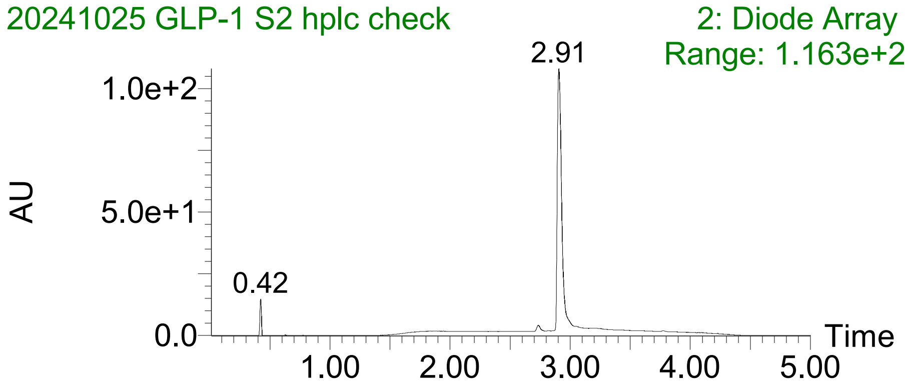

Figure S179. UV (190-400 nm) trace from UPLC-MS analysis of purified GLP-1 (12-31, AO) **S45** with N-terminal AO(Bn) gradient 5-95%

CH_3CN/H_2O containing 0.1% TFA over 5 min at a flow rate of 0.4 mL/min.

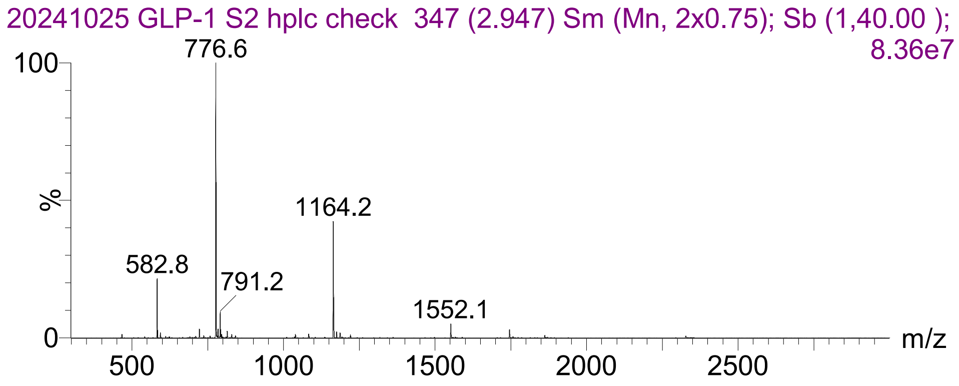

Figure S180. ESI-MS calcd. for $C_{110}H_{166}N_{28}O_{28}$ $[M+2H]^{2+}$ m/z = 1165.4 found 1164.2; $[M+3H]^{3+}$ m/z = 777.2, found 776.6; $[M+4H]^{4+}$ m/z = 583.2, found 582.8.

Synthesis of GLP-1 (1-31, AO) **S46**

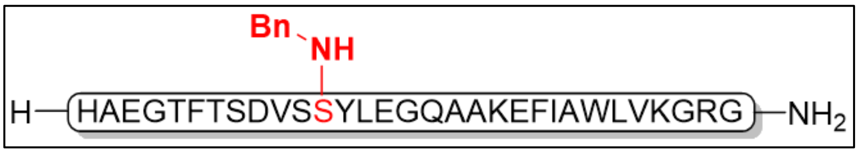

The ligation between GLP-1 (1-11, AO) SAL ester **S44** (1 mg, 0.797 μmol) and GLP-1 (12-31, AO) **S45** (1.95 mg, 0.837 μmol) was performed as described in the general procedure for aminooxy ligation in Collidine/Acetic acid 1:6 at 10 mM for overnight. Purification via preparative reverse phase HPLC (20-50% CH_3CN/H_2O over 45 min, 0.1% TFA) followed by lyophilization afforded GLP-1 (1-31, AO) **S46** (1.27 mg, 46.5% yield) as white solids.

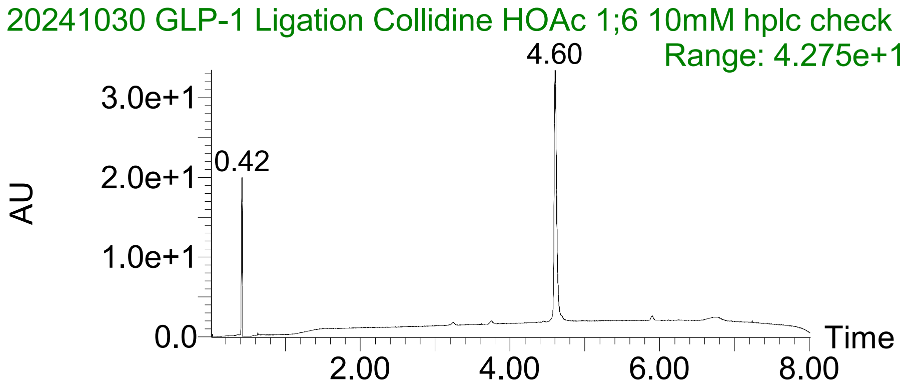

Figure S181. UV (190-400 nm) trace from UPLC-MS analysis of purified GLP-1 (1-31, AO) **S46** gradient 5-75% CH_3CN/H_2O containing 0.1% TFA over 8 min at a flow rate of 0.4 mL/min.

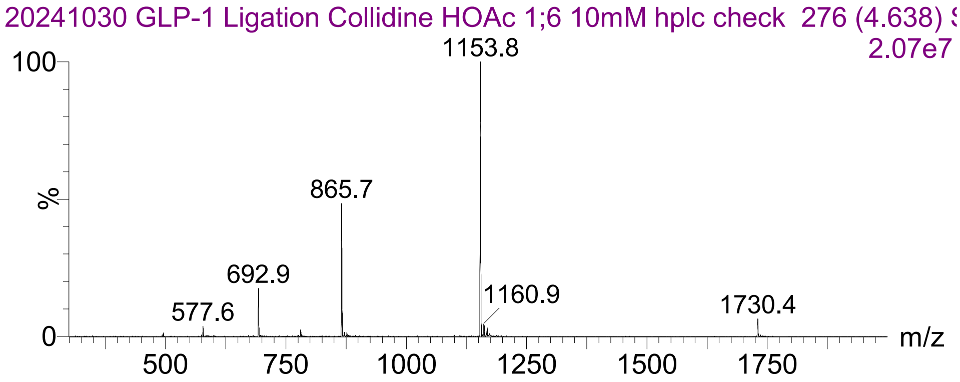

Figure S182. ESI-MS calcd. for $C_{158}H_{235}N_{41}O_{47}$ $[M+2H]^{2+}$ m/z = 1731.4 found 1730.4; $[M+3H]^{3+}$ m/z = 1154.6, found 1153.8; $[M+4H]^{4+}$ m/z = 866.2, found 865.7; $[M+5H]^{5+}$ m/z = 693.2, found 692.9; $[M+6H]^{6+}$ m/z = 577.8, found 577.6.

Synthesis of GLP-1 analogue ***38*** *by CEL*

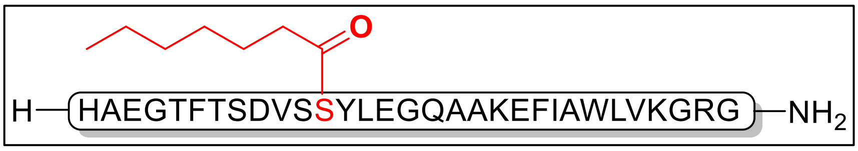

38

GLP-1 (1-31, lipid ester) **38** was synthesized according to the general CEL

method. GLP-1 (1-31, AO) (5 mg, µmol) was incubated with 2-Oxooctanoicacid (0.57 mg, 3.61 µmol) in aqueous DMSO with 0.1M oxalic acid at 20 mM, 60°C for 16 h. The crude peptide was purified by preparative reverse-phase HPLC (20-55% CH_3CN/H_2O over 45 min) and lyophilized to afford the desired lipid ester **38** (1.08 mg, 21.6% yield).

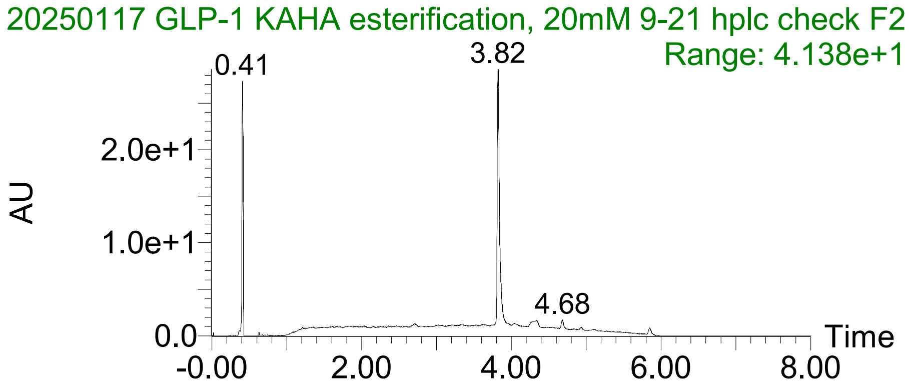

Figure S183. UV (190-400 nm) trace from UPLC-MS analysis of purified GLP-1 (1-31, lipid ester) **38** gradient 5-95% CH_3CN/H_2O containing 0.1% TFA over 8 min at a flow rate of 0.4 mL/min.

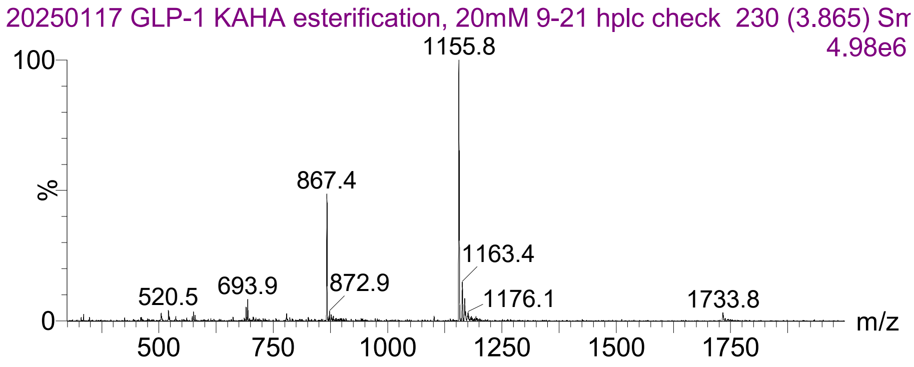

Figure S184. ESI-MS calcd. for $C_{158}H_{240}N_{40}O_{48}$ $[M+2H]^{2+}$ m/z = 1734.9 found 1733.8; $[M+3H]^{3+}$ m/z = 1157.0, found 1155.8; $[M+4H]^{4+}$ m/z = 868.0, found 867.4; $[M+5H]^{5+}$ m/z = 694.6, found 693.9.

5.1.5.5. Synthesis of peptide **39** with O-peptidylation by CEL

Synthesis of peptide ketoacid **S47**

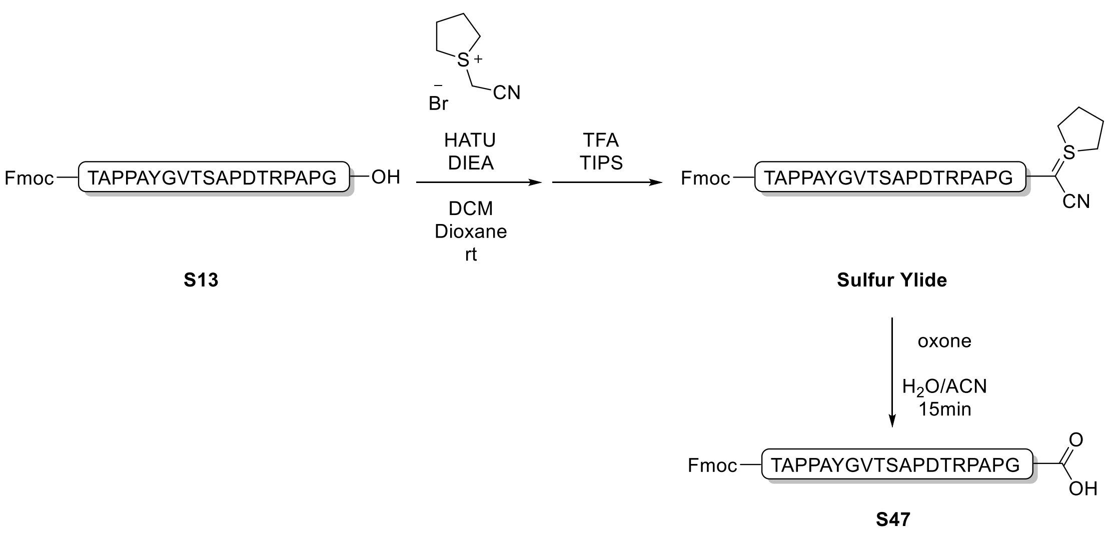

Scheme S12. Synthetic route of peptide ketoacid **S47**.

Peptide ketoacid **S47** was synthesized according to the reported method.[5] Crude peptide **S13** (264 mg, 0.1 mmol) was dissolved in DCM/Dioxane (1/1, v) with the addition of HATU and DIEA. The reaction mixture was stirred overnight and then deprotected by TFA/TIPS/H2O for 1h. The crude peptide was purified by preparative reverse-phase HPLC (20-60% CH_3CN/H_2O over 45 min) and lyophilized to afford the desired **sulfur ylide** intermediate (149 mg, 69% yield).

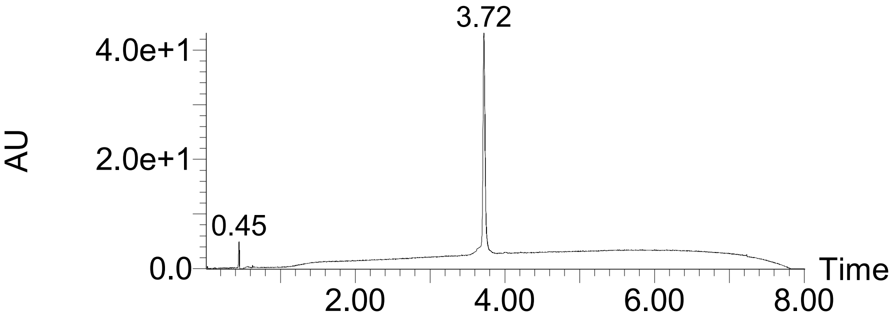

Figure S185. UV (190-400 nm) trace from UPLC-MS analysis of purified peptide **sulfur ylide** gradient 5-95% CH_3CN/H_2O containing 0.1% TFA over 8 min at a flow rate of 0.4 mL/min.

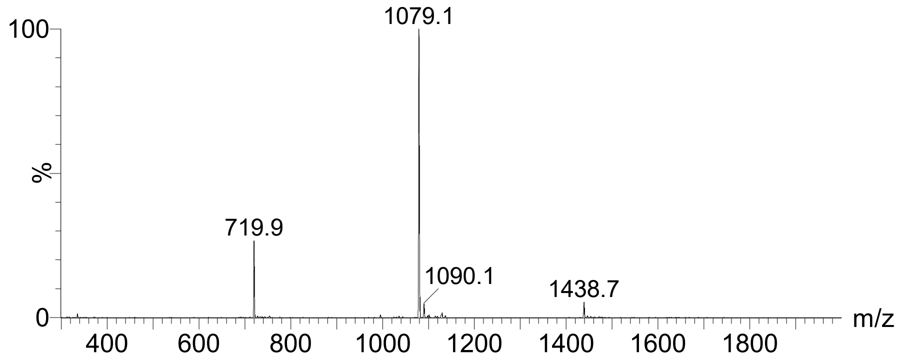

Figure S186. ESI-MS calcd. for $C_{101}H_{141}N_{23}O_{28}S$ $[M+2H]^{2+}$ m/z = 1079.2, found 1079.1; $[M+3H]^{3+}$ m/z = 719.8 found 719.9.

Then the sulfur ylide (30 mg, 14 umol) was activated by Oxone in CH_3CN/H_2O. The crude peptide was purified by preparative reverse-phase HPLC (20-55% CH_3CN/H_2O over 45 min) and lyophilized to afford the peptide ketoacid **S47** (16.4 mg, 56.6% yield).

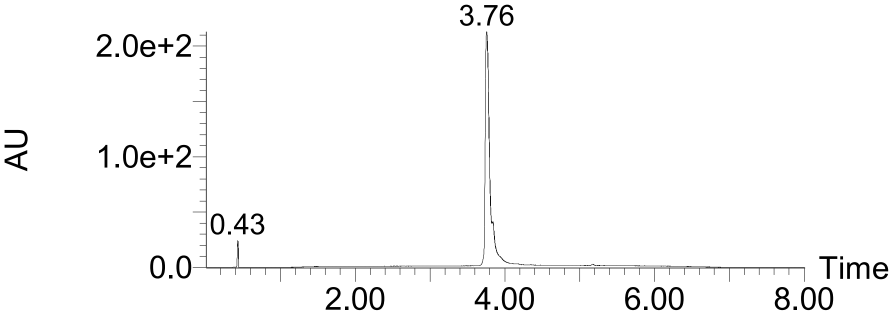

Figure S187. UV (190-400 nm) trace from UPLC-MS analysis of purified peptide ketoacid **S47** gradient 5-95% CH_3CN/H_2O containing 0.1% TFA over 8 min at a flow rate of 0.4 mL/min.

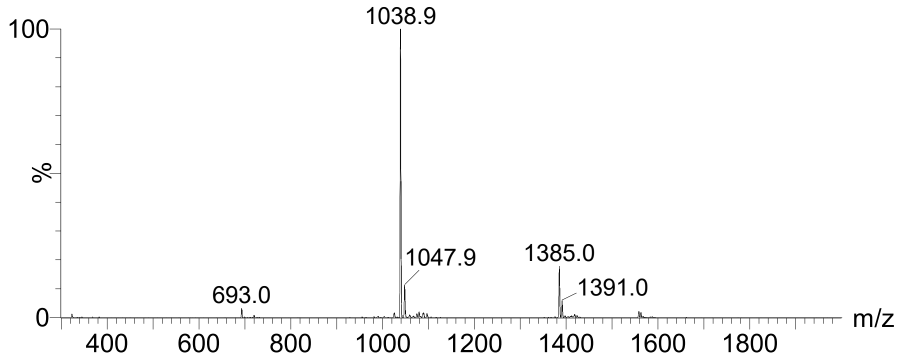

Figure S188. ESI-MS calcd. for $C_{96}H_{134}N_{22}O_{30}$ $[M+2H]^{2+}$ m/z = 1038.5, found 1038.9; $[M+3H]^{3+}$ m/z = 692.7 found 693.0.

Synthesis of peptide **39**

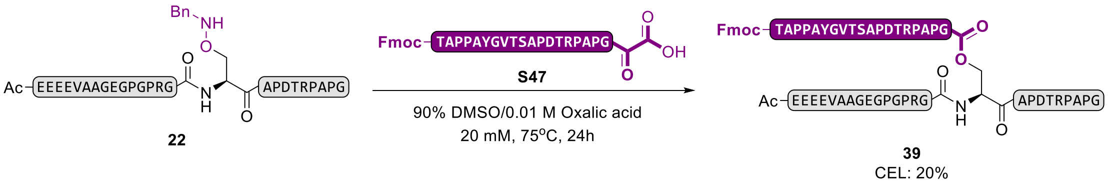

Peptide ester **39** was synthesized according to the general CEL method. **22** (1.5 mg, 0.581 µmol) was incubated with peptide ketoacid **S47** (2.4 mg, 1.16 µmol) in aqueous DMSO with 0.01M oxalic acid at 40 mM, 75°C for 24 h. The crude peptide was purified by preparative reverse-phase HPLC (20-55% CH_3CN/H_2O over 45 min) and lyophilized to afford the desired peptide ester (0.52 mg, 19.9% yield).

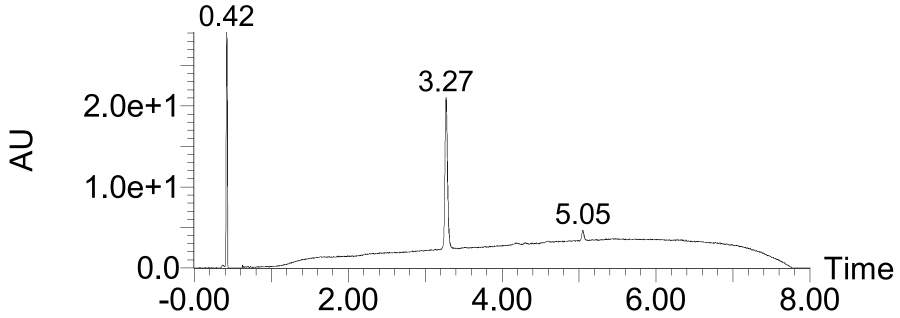

Figure S189. UV (190-400 nm) trace from UPLC-MS analysis of purified peptide ester **39** gradient 5-95% CH_3CN/H_2O containing 0.1% TFA over 8 min at a flow rate of 0.4 mL/min.

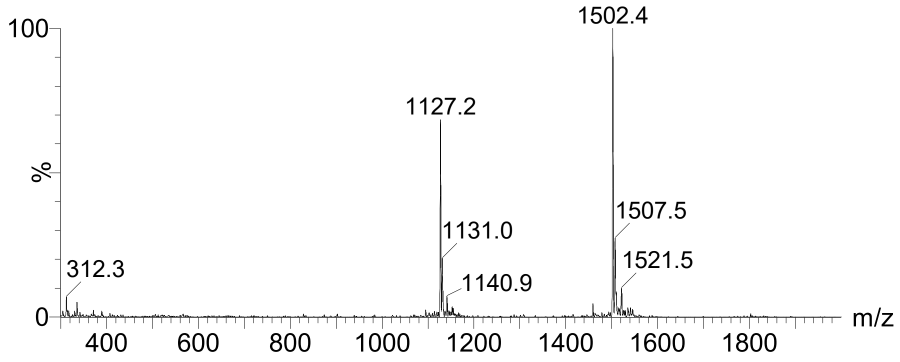

Figure S190. ESI-MS calcd. for $C_{197}H_{291}N_{53}O_{69}$ $[M+3H]^{3+}$ m/z = 1502.0, found 1502.4; $[M+4H]^{4+}$ m/z = 1126.8, found 1127.2.

5.1.5.6. Synthesis of Nesiritide analogue **43**

Synthesis of fully protected Nesiritide (1-19) ***S48*** *by SPPS*

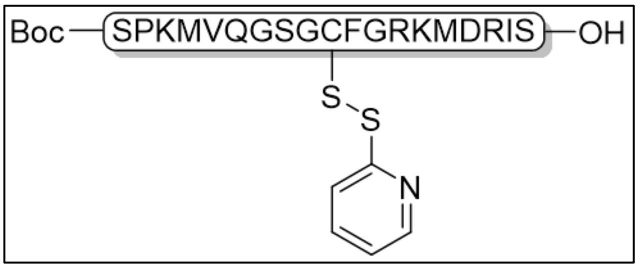

S48

Nesiritide (1-19) **S48** was synthesized according to the standard protocol of SPPS at 0.04 mmol scale. 4 equiv. of Fmoc-Cys(SSMe)-OH was used along with 4 equiv. of HATU, 8 equiv. DIEA in DMF for the coupling of Cys-10. After coupling the last amino acid, the -SMe protecting group was removed on resin with 10% 3,6-dioxa-1,8-octanedithiol (DODT) and 10% DIEA in DMF for overnight. The resin was then washed with DMF (5 mL × 3), CH_2Cl_2 (5 mL × 3), and DMF (5 mL × 3). 1 M of 4,4'-Dithiodipyridine in DMF was added to the resin and was incubated for 30 minutes for 2 times. Cleavage of the fully protected peptide from the resin was done according to the general protocol to yield the fully protected crude Nesiritide (1-19) **S48** as a yellow solid.

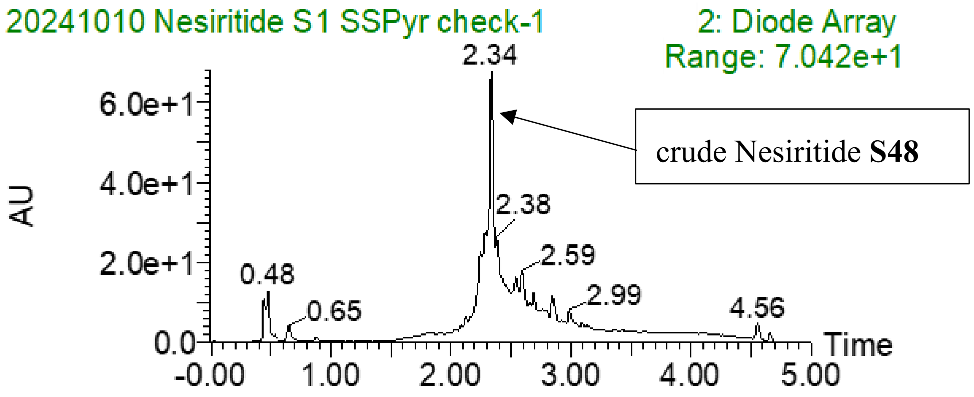

Figure S191. UV (190-400 nm) trace from UPLC-MS analysis of crude Nesiritide (1-19) **S48** gradient 5-95% CH_3CN/H_2O containing 0.1% TFA over 5 min at a flow rate of 0.4 mL/min.

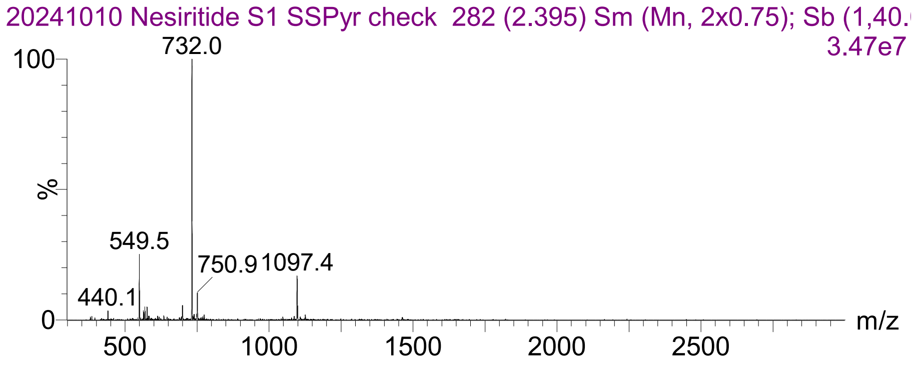

Figure S192. ESI-MS calcd. for $C_{91}H_{149}N_{29}O_{26}S_4$ $[M+2H]^{2+}$ m/z = 1097.8,

found 1097.4; $[M+3H]^{3+}$ m/z = 732.2, found 732.0; $[M+4H]^{4+}$ m/z = 549.4, found 549.4.

Synthesis of Nesiritide (1-20) SAL ester **41**

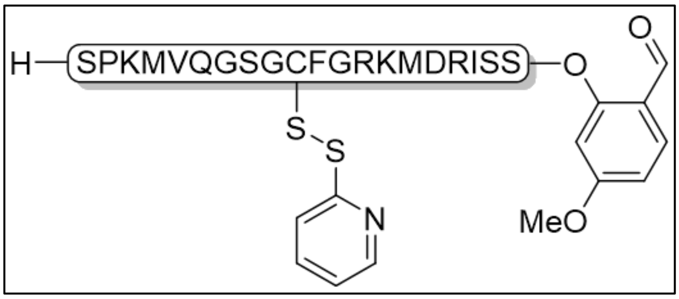

41

Nesiritide (1-20) SAL ester **41** was synthesized according to the general SAL ester preparation method at 0.085 mmol scale. The crude peptide was purified by preparative reverse-phase HPLC (10-45% CH_3CN/H_2O over 45 min) and lyophilized to afford the desired SAL ester **41** (23.2 mg, 11.3% yield).

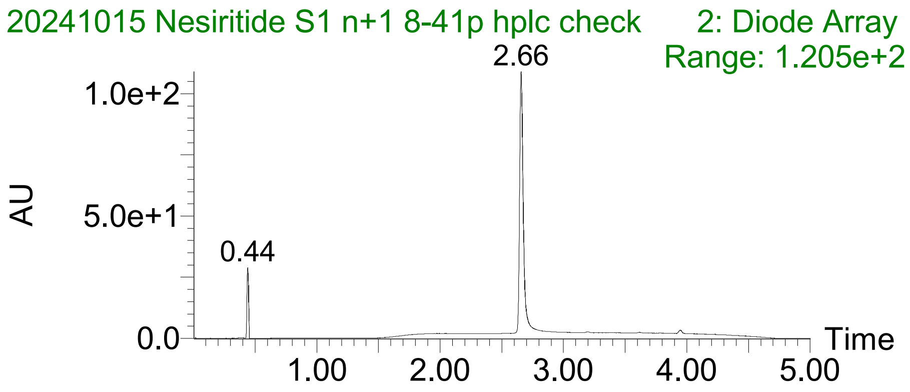

Figure S193. UV (190-400 nm) trace from UPLC-MS analysis of purified Nesiritide (1-20) SAL ester **41** gradient 5-95% CH_3CN/H_2O containing 0.1% TFA over 5 min at a flow rate of 0.4 mL/min.

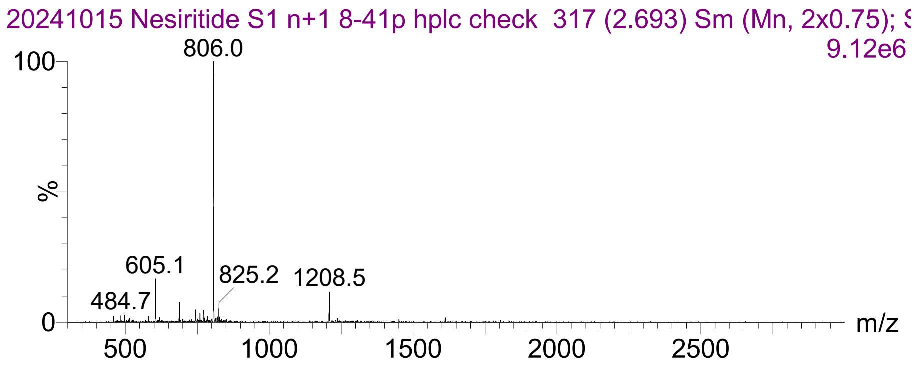

Figure S194. ESI-MS calcd. for $C_{102}H_{160}N_{30}O_{30}S_4$ $[M+2H]^{2+}$ m/z = 1208.4, found 1208.5; $[M+3H]^{3+}$ m/z = 805.9, found 806.0; $[M+4H]^{4+}$ m/z = 604.7, found 605.1.

Synthesis of Nesiritide (21-32, AO) with N-terminal AO(Bn) **40**

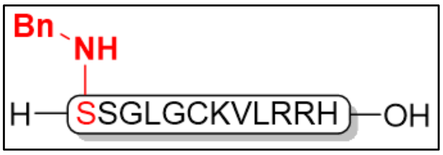

40

Nesiritide (21-32, AO) with N-terminal AO(Bn) **40** was synthesized according to the standard protocol of SPPS at 0.05 mmol scale. The crude peptide was purified by preparative reverse-phase HPLC (5-45% CH_3CN/H_2O over 45 min) and lyophilized to afford the desired peptide **40** (36.4 mg, 51.3% yield).

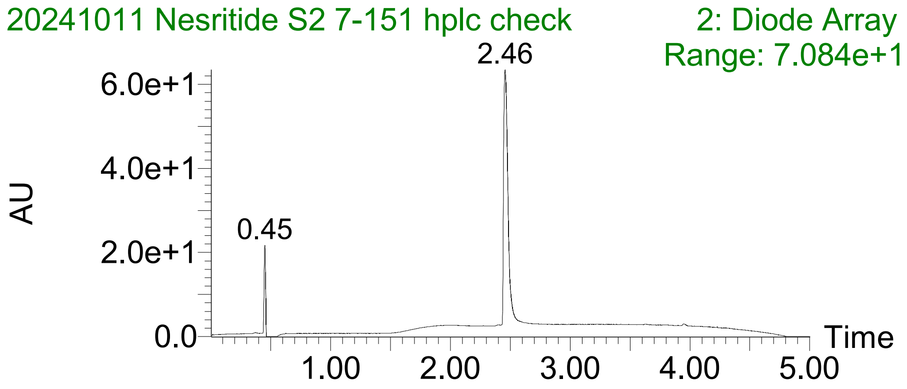

Figure S195. UV (190-400 nm) trace from UPLC-MS analysis of purified Nesiritide (21-32, AO) with N-terminal AO(Bn) **40** gradient 5-95% CH_3CN/H_2O containing 0.1% TFA over 5 min at a flow rate of 0.4 mL/min.

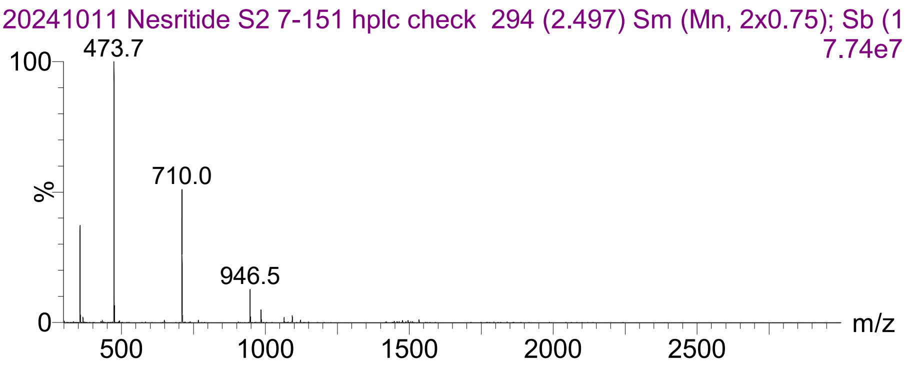

Figure S196. ESI-MS calcd. for $C_{61}H_{104}N_{22}O_{15}S$ $[M+2H]^{2+}$ m/z = 709.9, found 710.0; $[M+3H]^{3+}$ m/z = 473.6, found 473.7; $[M+4H]^{4+}$ m/z = 355.4, found 355.6.

Synthesis of Nesiritide (1-32, AO) **S49**

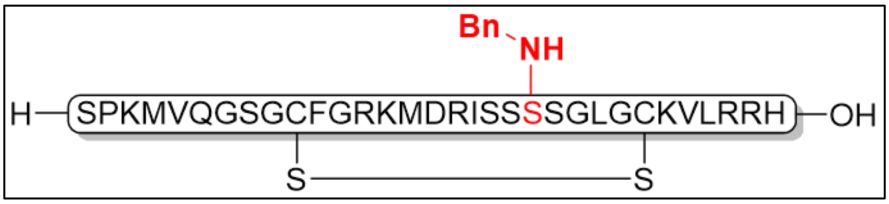

S49

Nesiritide (1-20) SAL ester **S49** (6 mg, 2.49 μmol) and Nesiritide (21-32, AO) (3.9 mg, 2.73 μmol) **S49** were first allowed to form disulfide bond in CH_3CN/H_2O 1:1 at 10 mM for 48 h. The reaction crude was then diluted with 6 M Guanidine-HCl buffered with 0.5 M sodium citrate at pH 4.5 to 0.5 mM and was further incubated at room temperature for 24 h. Purification via preparative reverse phase HPLC (10-40% CH_3CN/H_2O over 45 min, 0.1% TFA) followed by lyophilization afforded Nesiritide (1-32, AO) **S49** (2.88 mg, 32.5% yield) as white solids.

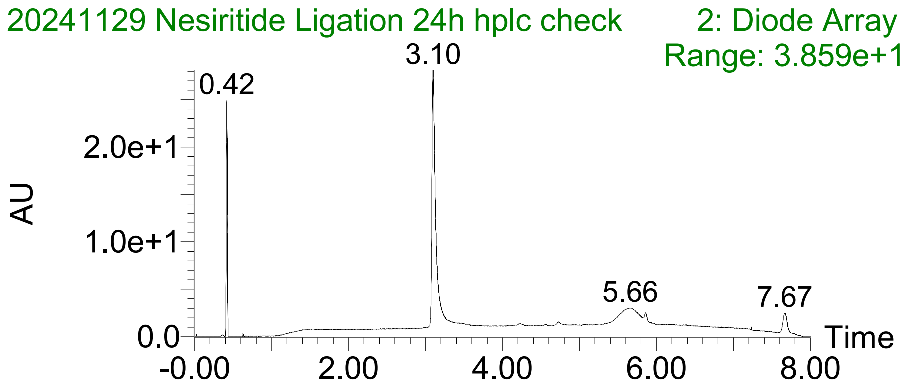

Figure S197. UV (190-400 nm) trace from UPLC-MS analysis of purified Nesiritide (1-32, AO) **S49** gradient 5-75% CH_3CN/H_2O containing 0.1% TFA over 8 min at a flow rate of 0.4 mL/min.

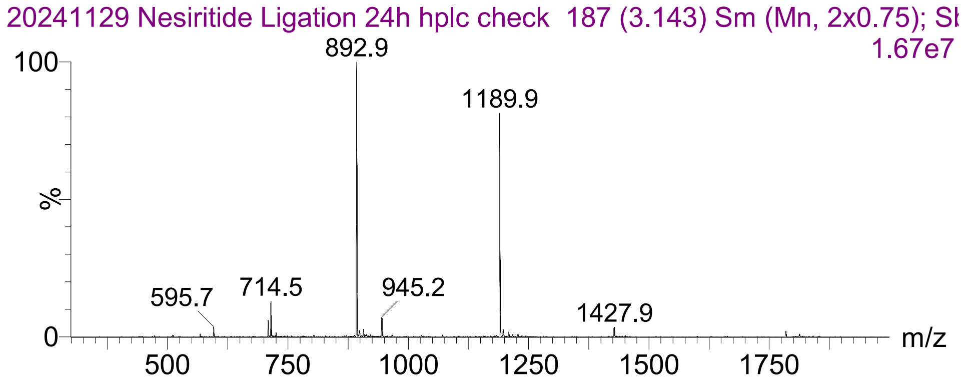

Figure S198. ESI-MS calcd. for $C_{150}H_{251}N_{51}O_{42}S_4$ $[M+3H]^{3+}$ m/z = 1190.7 found 1189.9; $[M+4H]^{4+}$ m/z = 893.3, found 892.9; $[M+5H]^{5+}$ m/z = 714.8, found 714.5; $[M+6H]^{6+}$ m/z = 595.9, found 595.7.

Synthesis of Nesiritide analogue ***43*** *by CEL*

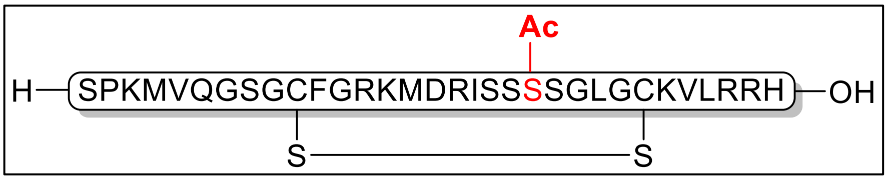

43

Nesiritide (1-32, Ac ester) **43** was synthesized according to the general CEL

method. Nesiritide (1-32, AO) (2 mg, 0.561 μmol) was incubated with pyruvic acid (0.198 μL, 2.81 μmol) in aqueous DMSO with 0.1M oxalic acid at 40 mM, 60°C for 5 h. The crude peptide was purified by preparative reverse-phase HPLC (10-30% CH_3CN/H_2O over 45 min) and lyophilized to afford the desired Ac ester **43** (0.70 mg, 35.7% yield).

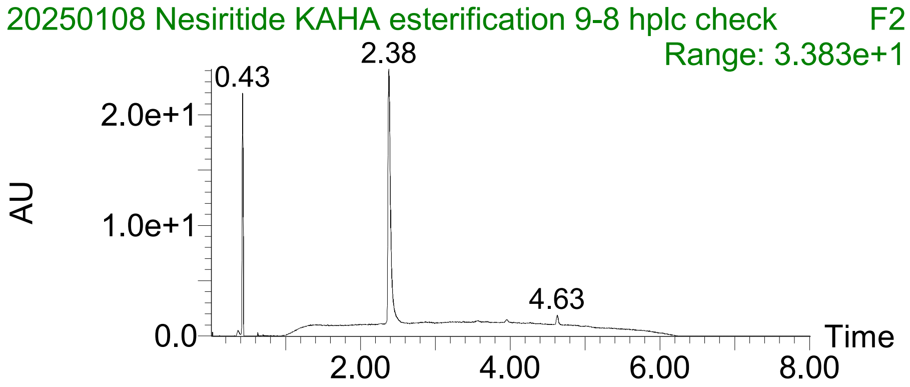

Figure S199. UV (190-400 nm) trace from UPLC-MS analysis of purified Nesiritide (1-32, Ac ester) **43** gradient 5-95% CH_3CN/H_2O containing 0.1% TFA over 8 min at a flow rate of 0.4 mL/min.

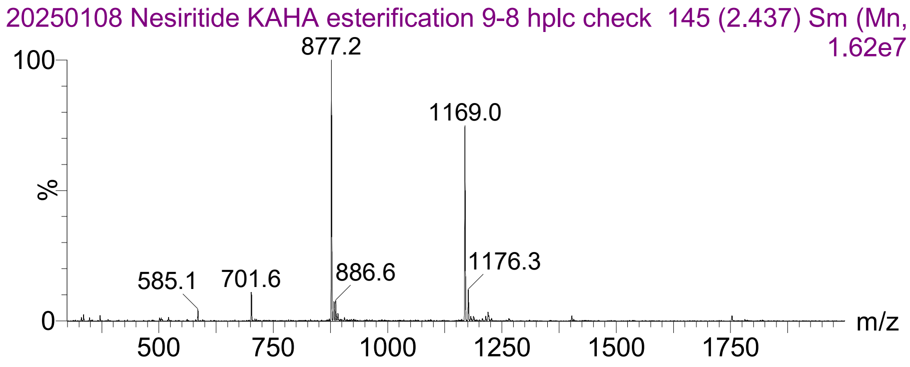

Figure S200. ESI-MS calcd. for $C_{145}H_{246}N_{50}O_{43}S_4$ $[M+3H]^{3+}$ m/z = 1169.7 found 1169.0; $[M+4H]^{4+}$ m/z = 877.5, found 877.2; $[M+5H]^{5+}$ m/z = 702.2, found 701.6; $[M+6H]^{6+}$ m/z = 585.4, found 585.1.

6. Chemical synthesis of homogeneous H2B 51 and 56

6.1. Synthesis of Histone H2B S92GlcNAc 51

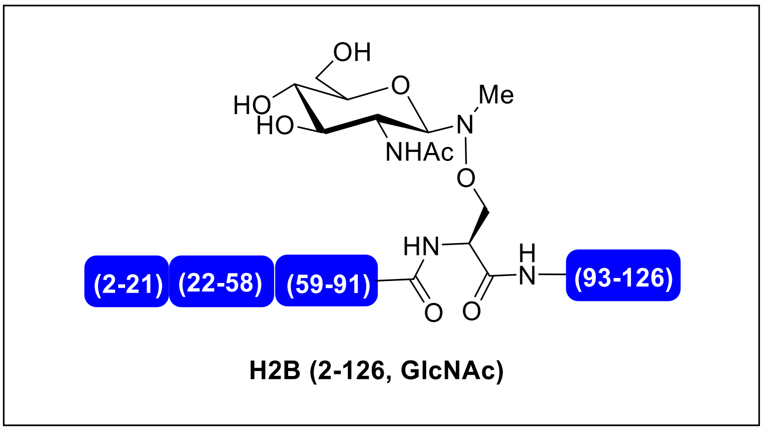

51

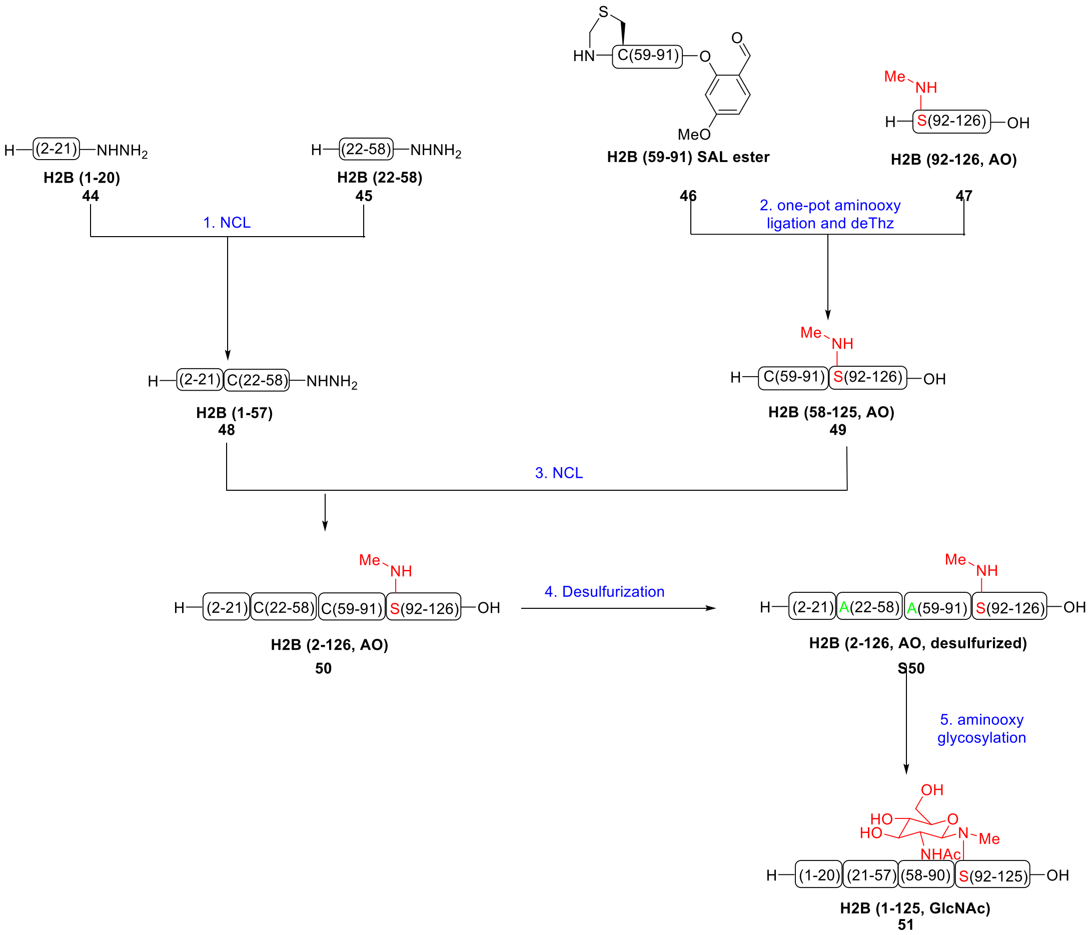

Scheme S13. Synthetic route of **51**.

6.1.1. Synthesis of Histone H2B (2-21) 44

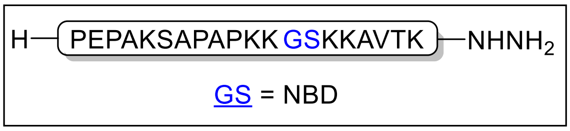

44

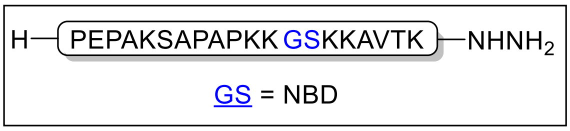

44

The commercially available 2-chlorotrityl resin at 0.1 mmol scale (CS Biochem, loading: ~0.45 mmol/g) was converted to hydrazine 2-chlorotrityl chloride resin as described in the general procedure for preparation of hydrazine 2-chlorotrityl chloride resin, the resulting resin was employed in the SPPS of Histone H2B (2-21) according to the standard protocol of SPPS. The crude peptide was purified by preparative reverse-phase HPLC (5-30% CH_3CN/H_2O over 45 min) and lyophilized to afford the desired peptide **44** (79.6, 39.1% yield)

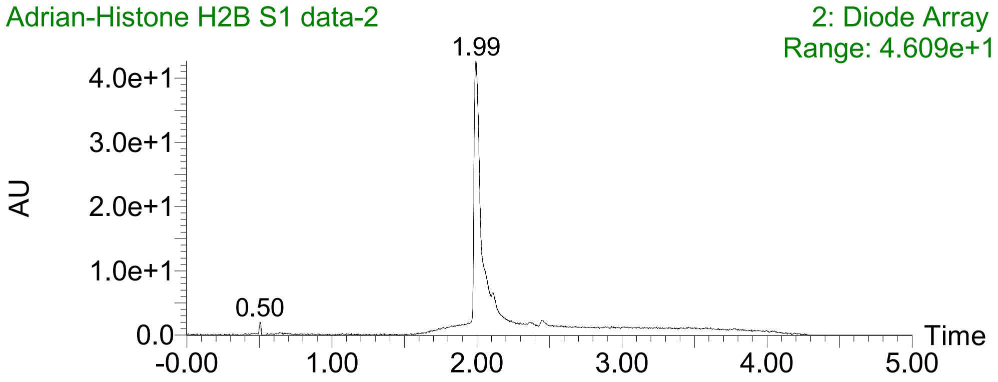

Figure S201. UV (190-400 nm) trace from UPLC-MS analysis of purified Histone H2B (2-21) **44** gradient 5-95% CH_3CN/H_2O containing 0.1% TFA over 5 min at a flow rate of 0.4 mL/min.

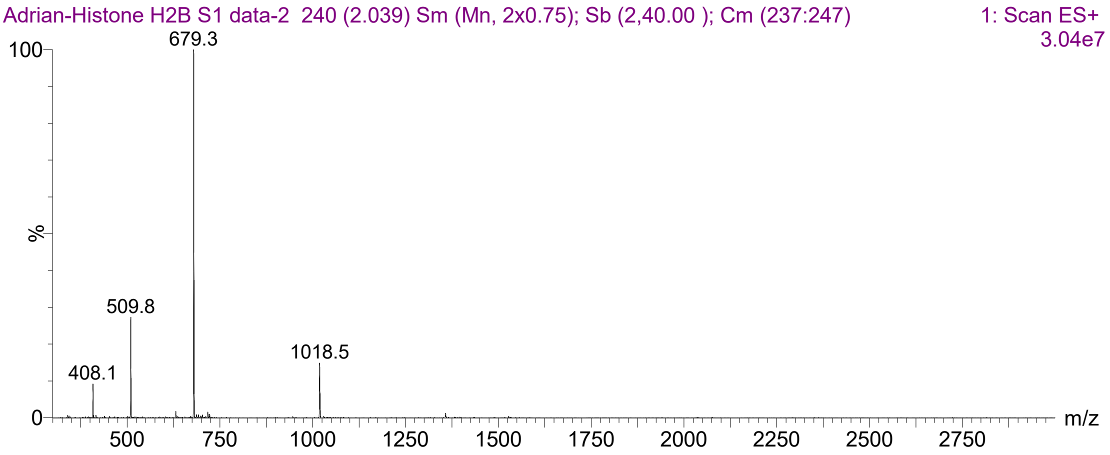

Figure S202. ESI-MS calcd. for $C_{90}H_{160}N_{28}O_{25}$ $[M+2H]^{2+}$ m/z = 1018.2 found 1018.5; $[M+3H]^{3+}$ m/z = 679.1, found 679.3; $[M+4H]^{4+}$ m/z = 509.6, found 509.8; $[M+5H]^{5+}$ m/z = 407.9, found 408.1.

6.1.2. Synthesis of Histone H2B (22-58) 45

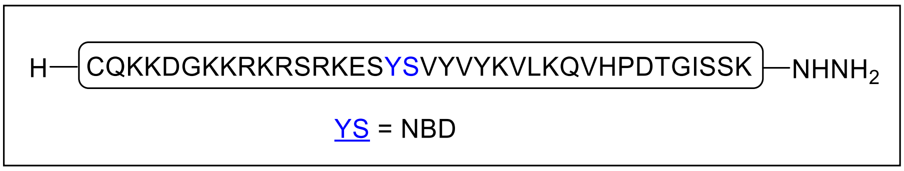

45

The commercially available 2-chlorotrityl resin at 0.1 mmol scale (CS Biochem, loading: ~0.45 mmol/g) was converted to hydrazine 2-chlorotrityl chloride resin as described in the general procedure for preparation of hydrazine 2-chlorotrityl chloride resin, the resulting resin was employed in the SPPS of Histone H2B (22-58) according to the standard protocol of SPPS. The crude peptide was purified by preparative reverse-phase HPLC (10-40% CH_3CN/H_2O over 45 min) and lyophilized to afford the desired peptide **45** (114 mg, 26.1% yield).

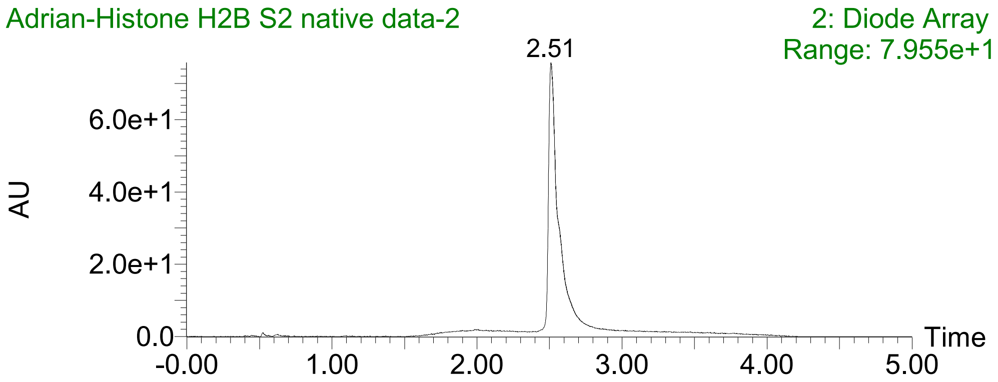

Figure S203. UV (190-400 nm) trace from UPLC-MS analysis of purified Histone H2B (22-58) **45** with internal AO(Bn) gradient 5-95% CH_3CN/H_2O containing 0.1% TFA over 5 min at a flow rate of 0.4 mL/min.

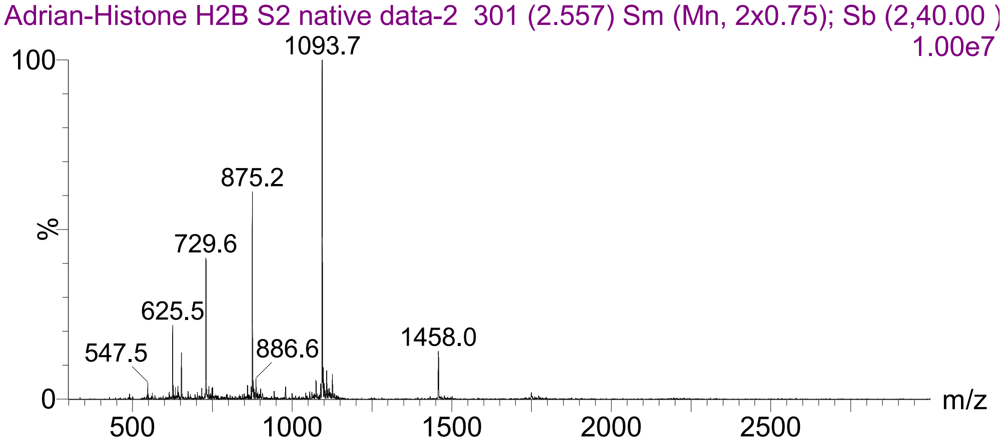

Figure S204. ESI-MS calcd. for $C_{191}H_{323}N_{61}O_{54}S$ $[M+3H]^{3+}$ m/z = 1457.7, found 1458.0; $[M+4H]^{4+}$ m/z = 1093.5, found 1093.7; $[M+5H]^{5+}$ m/z = 875.0, found 875.2; $[M+6H]^{6+}$ m/z = 729.4, found 729.6; $[M+7H]^{7+}$ m/z = 625.3, found 625.3; $[M+8H]^{8+}$ m/z = 547.3, found 547.5.

6.1.3. Synthesis of Histone H2B (59-91) SAL ester 46

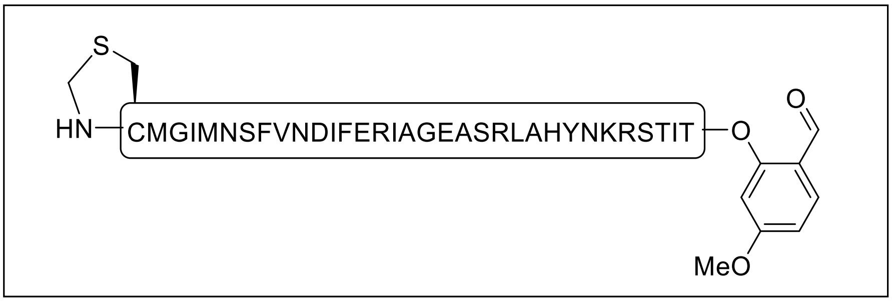

46

Histone H2B (59-91) SAL ester **46** was synthesized according to the general SAL ester preparation method at 0.05 mmol scale. The crude peptide was purified by preparative reverse-phase HPLC (35-70% CH_3CN/H_2O over 45 min) and lyophilized to afford the desired SAL ester **46** (11 mg, 5.6% yield)

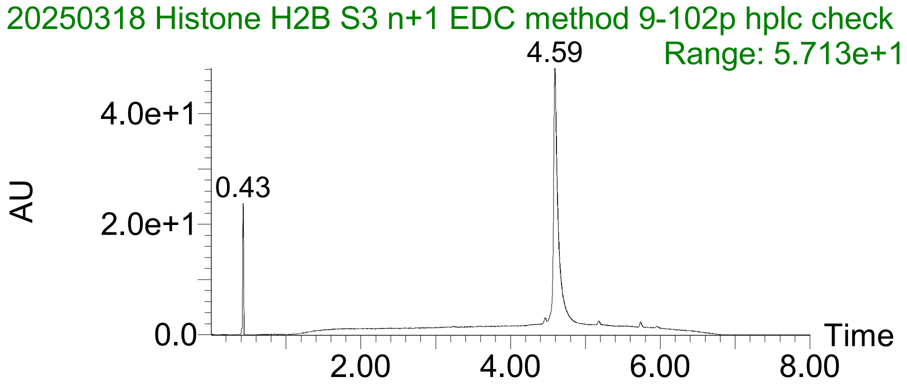

Figure S205. UV (190-400 nm) trace from UPLC-MS analysis of purified Histone H2B (59-91) SAL ester **46** gradient 5-95% CH_3CN/H_2O containing 0.1% TFA over 8 min at a flow rate of 0.4 mL/min.

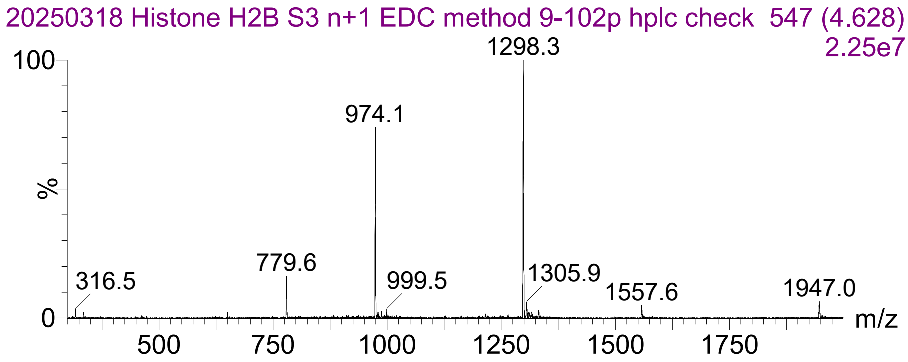

Figure S206. ESI-MS calcd. for $C_{170}H_{264}N_{48}O_{51}S_3$ $[M+2H]^{2+}$ m/z = 1947.2 found 1947.0; $[M+3H]^{3+}$ m/z = 1298.5, found 1298.3; $[M+4H]^{4+}$ m/z = 974.1, found 974.1; $[M+5H]^{5+}$ m/z = 779.5, found 779.6.

6.1.4. Synthesis of Histone H2B (92-126, AO Me) with N-terminal AO(Me) 47

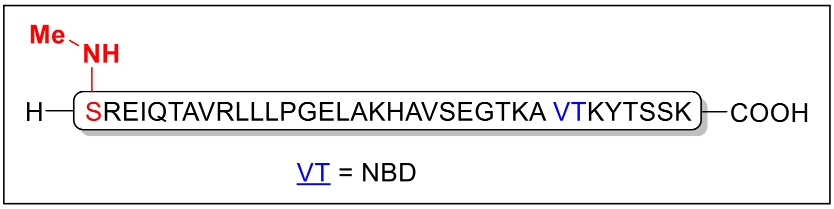

47

Histone H2B (92-126, AO) **47** with N-terminal AO(Me) was synthesized according to the standard protocol of SPPS at 0.1 mmol scale. The crude peptide was purified by preparative reverse-phase HPLC (10-60% CH_3CN/H_2O over 35 min) and lyophilized to afford the desired peptide **47** (86.7 mg, 22.8% yield).

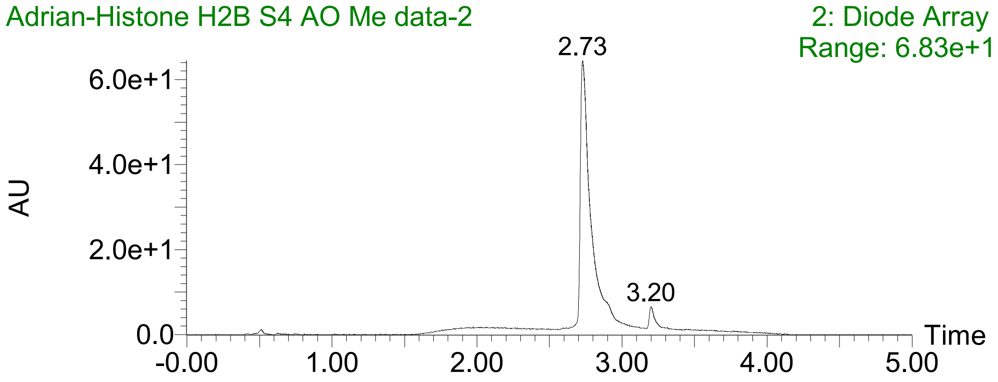

Figure S207. UV (190-400 nm) trace from UPLC-MS analysis of purified Histone H2B (92-126, AO, Me) with N-terminal AO(Me) **47** gradient 5-95% CH_3CN/H_2O containing 0.1% TFA over 5 min at a flow rate of 0.4 mL/min.

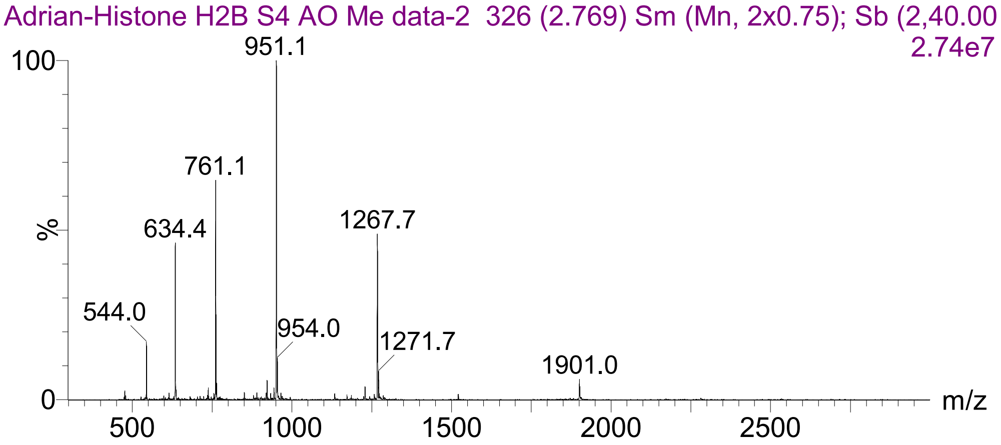

Figure S208. ESI-MS calcd. for $C_{166}H_{285}N_{49}O_{52}$ $[M+2H]^{2+}$ m/z = 1900.7 found 1901.0; $[M+3H]^{3+}$ m/z = 1267.5, found 1267.7; $[M+4H]^{4+}$ m/z = 950.9, found 951.1; $[M+5H]^{5+}$ m/z = 760.9, found 761.1; $[M+6H]^{6+}$ m/z = 634.2, found 634.4; $[M+7H]^{7+}$ m/z = 543.8, found 544.0.

6.1.5. Synthesis of Histone H2B (2-58) 48 by Native Chemical Ligation (NCL)

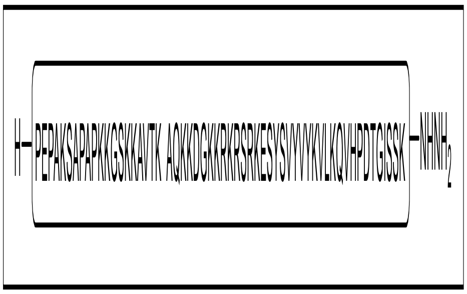

48

Histone H2B (2-58) **48** was synthesized according to the general Native Chemical Ligation method. Histone H2B (2-21) **44** (24.7 mg, 12 μmol) was dissolved in aqueous buffer containing 6 M Guanidine-HCl and 0.2 M NaH_2PO_4 (pH = 3.0) (peptide final concentration is 20 mg/mL). After that, MPAA (10.2 mg, 60 μmol) and AcAc (3.1 μL, 30 μmol) were added into the above mixture. The pH was adjusted to 2 and the reaction mixture was stirred under room temperature for 3 h, then Histone H2B (22-58) **45** (26.5mg, 6 μmol) was added

into the reaction mixture, and the pH of reaction mixture was then adjusted to 6.8 slowly with aqueous NaOH solution (1M) to initiate the Native Chemical Ligation at room temperature (N-terminal peptide final concentration is 3 mM). After no improvement of ligation product yield, 5% of $NH_2NH_2{\cdot}H_2O$ was added to the reaction mixture for 30 min to convert the N-terminal peptidyl hydrazone to peptidyl hydrazine. Purification via preparative reverse phase HPLC (10-60% CH_3CN/H_2O over 45 min, 0.1% TFA) followed by lyophilization afforded Histone H2B (2-58) **48** (18.2 mg, 47.1% yield) as white solids.

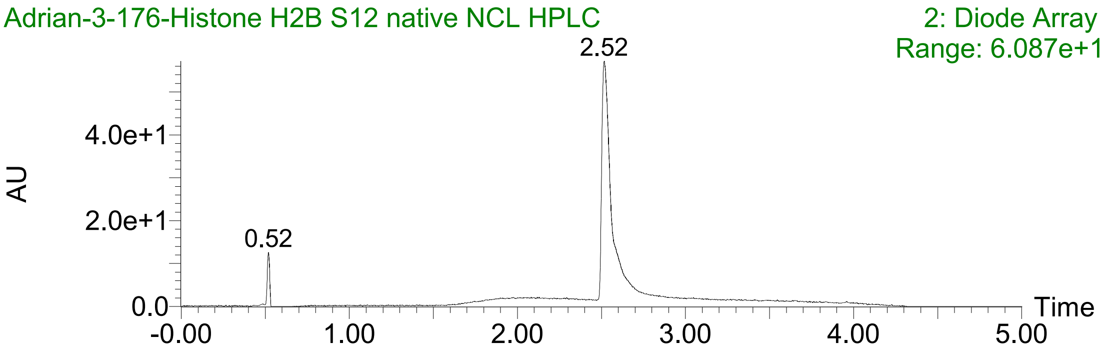

Figure S209. UV (190-400 nm) trace from UPLC-MS analysis of purified Histone H2B (2-58) **48** gradient 5-95% CH_3CN/H_2O containing 0.1% TFA over 5 min at a flow rate of 0.4 mL/min.

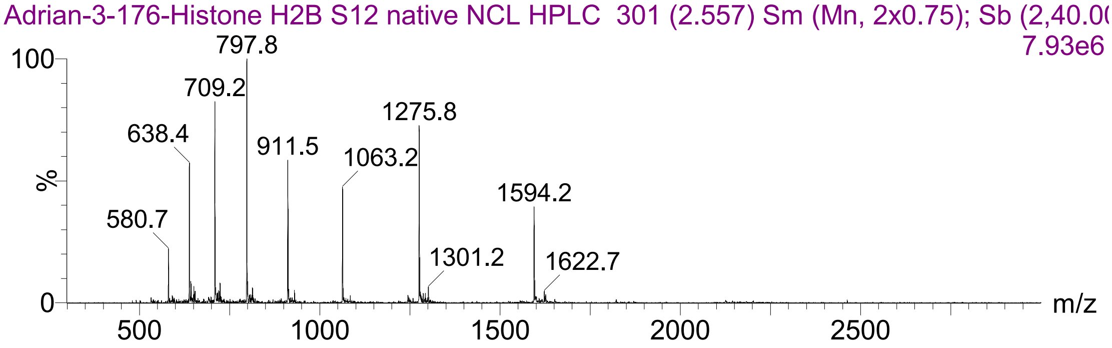

Figure S210. ESI-MS calcd. for $C_{281}H_{479}N_{87}O_{79}S$ $[M+4H]^{4+}$ m/z = 1594.1, found 1594.2; $[M+5H]^{5+}$ m/z = 1275.5, found 1275.8; $[M+6H]^{6+}$ m/z = 1063.1, found 1063.2; $[M+7H]^{7+}$ m/z = 911.4, found 911.5; $[M+8H]^{8+}$ m/z = 797.6, found 797.8; $[M+9H]^{9+}$ m/z = 709.1, found 709.2; $[M+10H]^{10+}$ m/z =

638.3, found 638.4; $[M+11H]^{11+}$ m/z = 580.3, found 580.7

6.1.6. Synthesis of Histone H2B (59-126, AO Me, deThz) 49 by aminooxy ligation

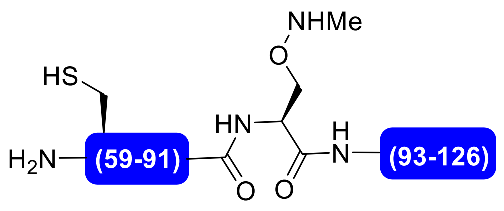

H2B (59-126, AO Me, deThz)

49

Histone H2B (59-126, AO Me, deThz) **49** was synthesized according to the general aminooxy ligation method. Histone H2B (59-91) SAL ester **46** (19.3 mg, 5.0 μmol) and Histone H2B (92-126, AO, Me) **47** (37.5 mg, 9.87 μmol) were incubated in 0.5 M sodium citrate with 6M Guanidine-HCl aqueous buffer at pH 4.5, 25 °C for 15h. After 15h, the reaction mixture was diluted to 10mM and $MeONH_2$ (20.7 mg, 248 μmol) and TCEP (14.2 mg, 49.6 μmol) were added to the reaction mixture. After that, the pH was adjusted to 4 and the mixture was stirred at 25 °C for 5 h to enable the Thz opening. Purification via preparative reverse phase HPLC (25-75% CH_3CN/H_2O over 45 min, 0.1% TFA) followed by lyophilization afforded Histone H2B (59-126, AO Me, deThz) **49** (11.1 mg, 29.8% yield) as white solids.

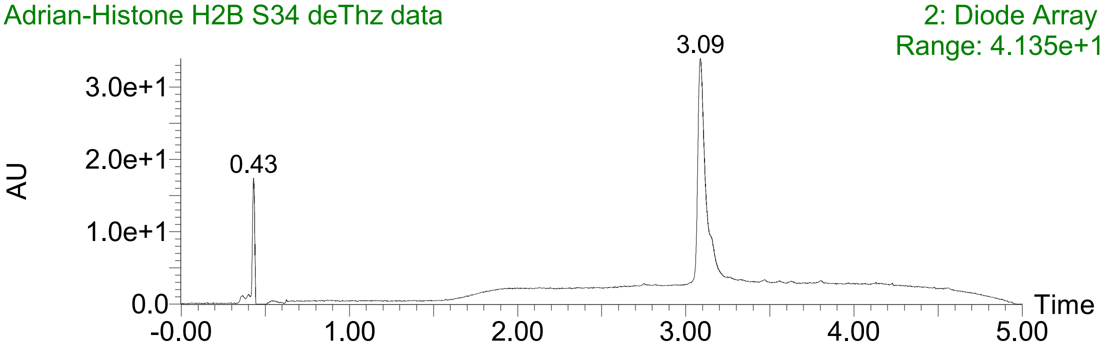

Figure S211. UV (190-400 nm) trace from UPLC-MS analysis of purified Histone H2B (59-126, AO Me, deThz) **49** gradient 5-95% CH_3CN/H_2O containing 0.1% TFA over 5 min at a flow rate of 0.4 mL/min.

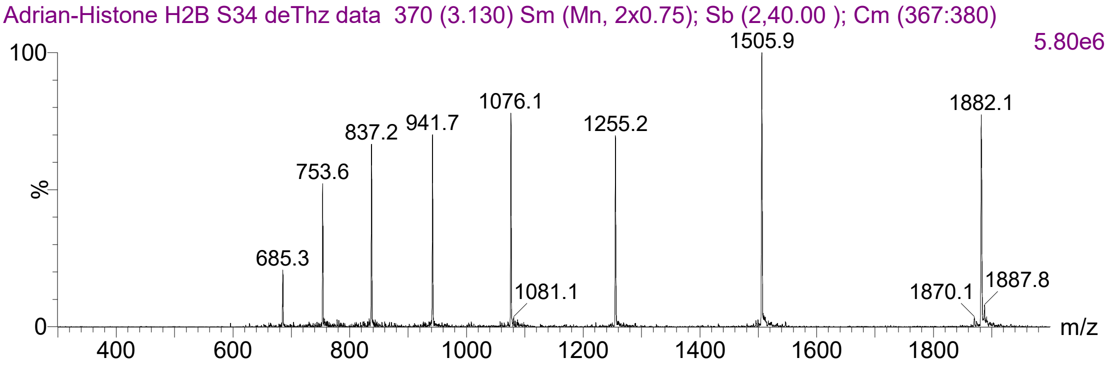

Figure S212. ESI-MS calcd. for $C_{327}H_{541}N_{97}O_{100}S_3$ $[M+4H]^{4+}$ m/z = 1882.9, found 1882.1; $[M+5H]^{5+}$ m/z = 1506.5, found 1505.9; $[M+6H]^{6+}$ m/z = 1255.6, found 1255.2; $[M+7H]^{7+}$ m/z = 1076.4, found 1076.1; $[M+8H]^{8+}$ m/z = 942.0, found 941.7; $[M+9H]^{9+}$ m/z = 837.4, found 837.2; $[M+10H]^{10+}$ m/z = 753.8, found 753.6; $[M+11H]^{11+}$ m/z = 685.3, found 685.3

6.1.7. Synthesis of Histone H2B (2-126, AO Me) 50 by Native Chemical Ligation

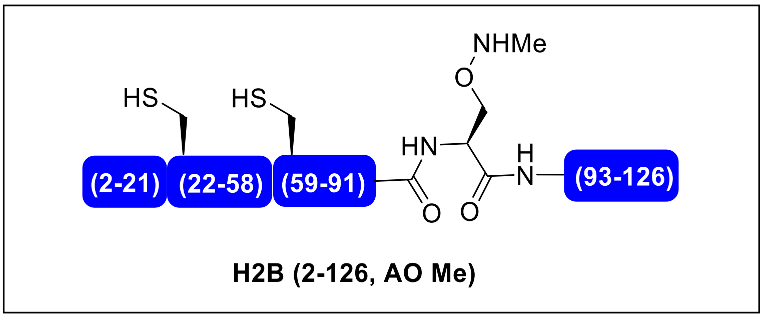

S50

Histone H2B (2-126, AO Me) S**50** was synthesized according to the general Native Chemical Ligation method. Histone H2B (2-58) **48** (13.7 mg, 2.2 μmol) was dissolved in aqueous buffer containing 6 M Guanidine-HCl and 0.2 M NaH_2PO_4 (pH = 3.0) (peptide final concentration is 20 mg/mL). After that, MPAA

(1.8 mg, 10.7 μmol) and AcAc (0.55 μL, 5.4 μmol) were added into the above mixture. The pH was adjusted to 2 and the reaction mixture was stirred under room temperature for 3 h, then Histone H2B (59-126, AO Me) **49** (8.1 mg, 1.1 μmol) was added into the reaction mixture, and the pH of reaction mixture was then adjusted to 6.8 slowly with aqueous NaOH solution (1M) to initiate the Native Chemical Ligation at room temperature (N-terminal peptide final concentration is 3 mM). After the completion of the reaction, purification via preparative reverse phase HPLC (25-75% CH_3CN/H_2O over 45 min, 0.1% TFA) followed by lyophilization afforded Histone H2B (2-126, AO Me) **S50** (4.81 mg, 32.2% yield) as white solids.

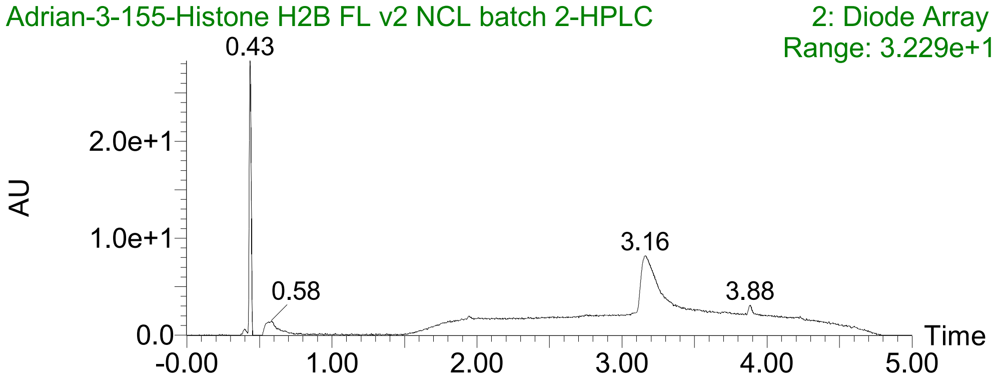

Figure S213. UV (190-400 nm) trace from UPLC-MS analysis of purified Histone H2B (2-126, AO Me) **S50** gradient 5-95% CH_3CN/H_2O containing 0.1% TFA over 5 min at a flow rate of 0.4 mL/min.

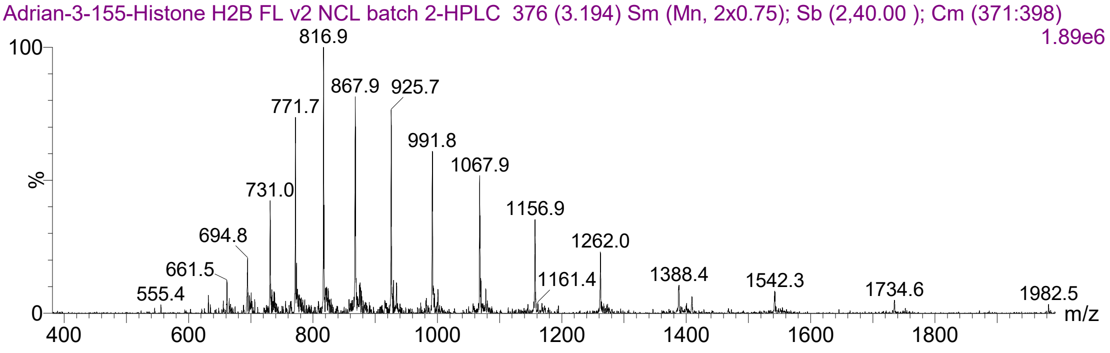

Figure S214. ESI-MS calcd. for $C_{608}H_{1016}N_{182}O_{179}S_4$ $[M+6H]^{6+}$ m/z = 2313.4,

found 2312.3; [M+7H]7^{+} m/z = 1982.2, found 1982.5; $[M+8H]^{8+}$ m/z = 1734.5, found 1734.6; $[M+9H]^{9+}$ m/z = 1541.9, found 1542.3; $[M+10H]^{10+}$ m/z = 1387.8, found 1388.4; $[M+11H]^{11+}$ m/z = 1261.7, found 1262.0; $[M+12H]^{12+}$ m/z = 1156.7, found 1156.9; $[M+13H]^{13+}$ m/z = 1067.8, found 1067.9; $[M+14H]^{14+}$ m/z = 991.6, found 991.8; $[M+15H]^{15+}$ m/z = 925.5, found 925.7; $[M+16]^{16+}$ m/z = 867.8, found 867.9; $[M+17H]^{17+}$ m/z = 816.8, found 816.9; $[M+18H]^{18+}$ m/z = 771.5, found 771.7; $[M+19H]^{19+}$ m/z = 730.9, found 731.0; $[M+20H]^{20+}$ m/z = 694.4, found 694.8; $[M+21H]^{21+}$ m/z = 661.4, found 661.5; $[M+22H]^{22+}$ m/z = 631.3, found 631.8; $[M+25H]^{25+}$ m/z = 555.7, found 555.4.

6.1.8. Synthesis of Histone H2B (2-126, AO Me, desulfurized) 50 by Add-and-Done Desulfurization

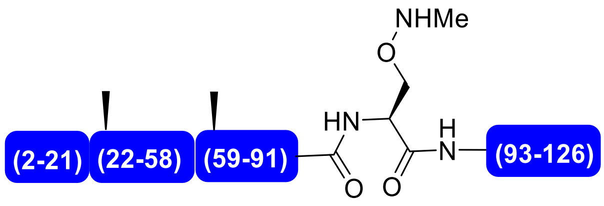

H2B (2-126, AO Me, desulfurized)

50

TCEP·HCl (0.2 M) was dissolved in a tube by 0.5 M sodium citrate with 6M Guanidine-HCl, and the pH of this solution would be adjusted to between 4.0~5.0 by addition of NaOH (1M). After that, Histone H2B (2-126, AO Me) **S50** (4.81 mg, 0.35 μmol) was added to the above solution with the final concentration of 1.0 mM. As follows, freshly weighted $NaBEt_4$ solid was added to the substrate solution at the final concentration of 0.1 M. After few minutes of shaking, another portion of $NaBEt_4$ solid was added at the final concentration of 0.05 M for complete desulfurization. Purification via preparative reverse phase HPLC (25-75% CH_3CN/H_2O over 45 min, 0.1% TFA) followed by

lyophilization afforded Histone H2B (2-126, AO Me, desulfurized) **50** (3.05 mg, 63.7% yield) as white solids.

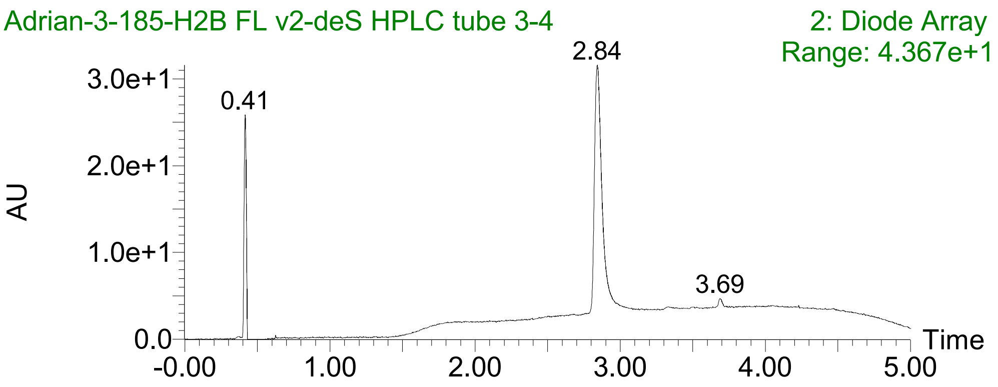

Figure S215. UV (190-400 nm) trace from UPLC-MS analysis of purified Histone H2B (2-126, AO Me, desulfurized) **50** gradient 5-95% CH_3CN/H_2O containing 0.1% TFA over 5 min at a flow rate of 0.4 mL/min.

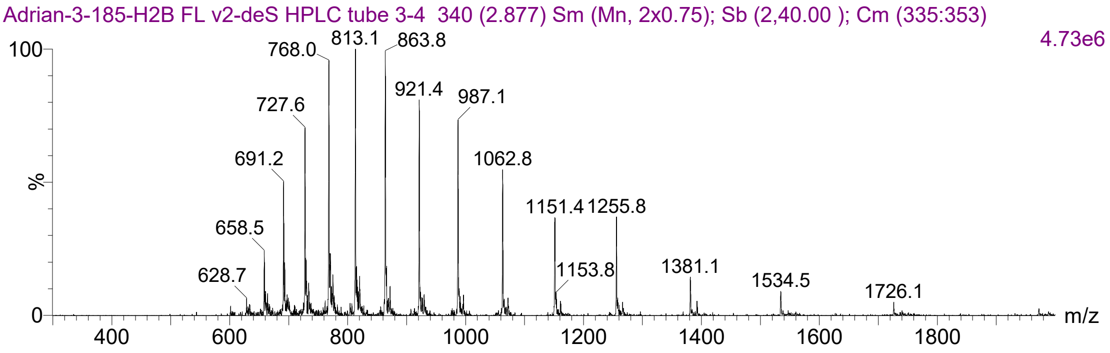

Figure S216. ESI-MS calcd. for $C_{608}H_{1016}N_{182}O_{179}S_2$ $[M+8H]^{8+}$ m/z = 1726.5, found 1726.1; $[M+9H]^{9+}$ m/z = 1534.8, found 1534.5; $[M+10H]^{10+}$ m/z = 1381.4, found 1381.1; $[M+11H]^{11+}$ m/z = 1255.9, found 1255.8; $[M+12H]^{12+}$ m/z = 1151.3, found 1151.4; $[M+13H]^{13+}$ m/z = 1062.8, found 1062.8; $[M+14H]^{14+}$ m/z = 987.0, found 987.1; $[M+15H]^{15+}$ m/z = 921.3, found 921.4; $[M+16H]^{16+}$ m/z = 863.8, found 863.8; $[M+17H]^{17+}$ m/z = 813.0, found 813.1; $[M+18H]^{18+}$ m/z = 767.9, found 768.0; $[M+19H]^{19+}$ m/z = 727.5, found 727.6; $[M+20H]^{20+}$ m/z = 691.2, found 691.2; $[M+21H]^{21+}$ m/z = 658.3, found 658.5; $[M+22H]^{22+}$ m/z = 628.5, found 628.7.

6.1.9. Synthesis of Histone H2B (2-126, GlcNAc) 51 by aminooxy glycosylation

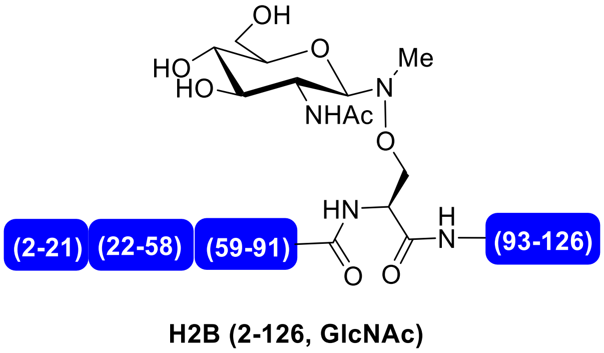

51

Histone H2B (2-126, GlcNAc) **51** was synthesized according to the general aminooxy glycosylation. Histone H2B (2-126, AO Me, desulfurized) **50** (0.87 mg, 63 nmol) was first dissolved in 15.5 μL of 6 M Guanidine-HCl buffered with 0.1 M sodium citrate at pH 4. Then 15.5 μL of 1M $ZnCl_2$ stock solution and 31.5 μL of 1M GlcNAc stock solution were added to the dissolved peptide, with the final peptide concentration at 1mM. The reaction was incubated and monitored at room temperature for 30 hours. After the completion of the reaction, the reaction mixture is diluted with 6 M Guanidine-HCl for purification by preparative reverse-phase HPLC (30-55% CH_3CN/H_2O over 45 min) and lyophilized to afford the desired glycosylated H2B **51** (0.49 mg, 55% yield).

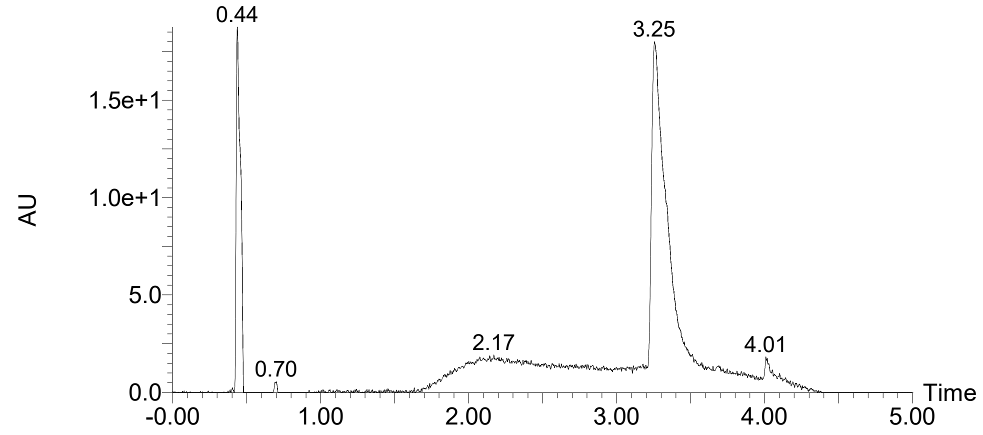

Figure S217. UV (190-400 nm) trace from UPLC-MS analysis of purified Histone H2B (2-126, GlcNAc) **51** gradient 5-95% CH_3CN/H_2O containing 0.1% TFA over 5 min at a flow rate of 0.4 mL/min.

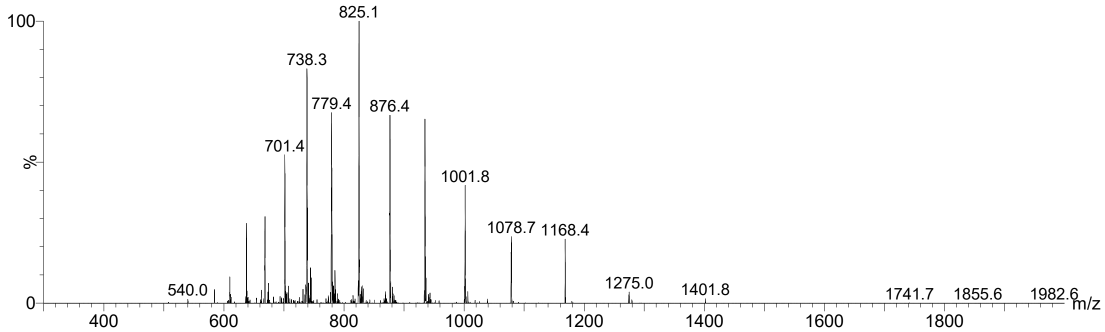

Figure S218. ESI-MS calcd. for $C_{616}H_{1029}N_{183}O_{184}S_2$ $[M+10H]^{10+}$ m/z = 1401.7, found 1401.8; $[M+11H]^{11+}$ m/z = 1274.4, found 1275.0; $[M+12H]^{12+}$ m/z = 1168.3, found 1168.4; $[M+13H]^{13+}$ m/z = 1078.5, found 1078.7; $[M+14H]^{14+}$ m/z = 1001.5, found 1001.8; $[M+15H]^{15+}$ m/z = 934.8, found 934.9; $[M+16H]^{16+}$ m/z = 876.5, found 876.4; $[M+17H]^{17+}$ m/z = 825.0, found 825.1; $[M+18H]^{18+}$ m/z = 779.2, found 779.4; $[M+19H]^{19+}$ m/z = 738.2, found 738.3; $[M+20H]^{20+}$ m/z = 701.4, found 701.4; $[M+21H]^{21+}$ m/z = 668.0, found 668.0; $[M+22H]^{22+}$ m/z = 637.7, found 637.6; $[M+23H]^{23+}$ m/z = 610.1, found 610.0; $[M+24H]^{24+}$ m/z = 584.6, found 585.5.

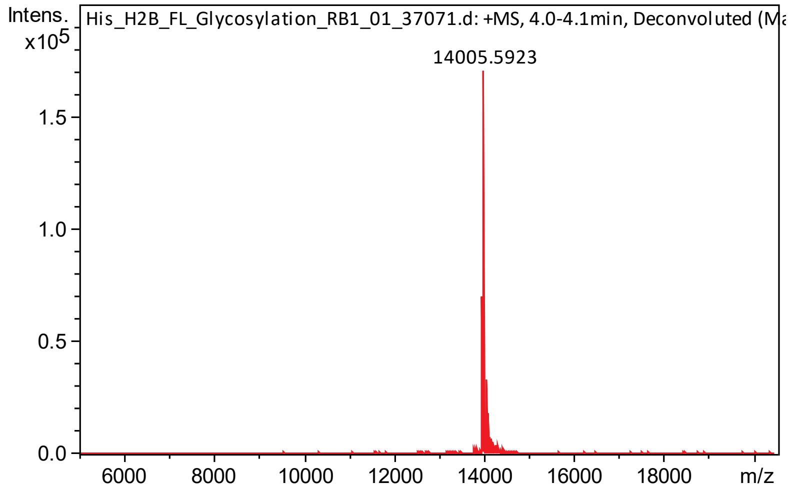

Figure S219. ESI-MS calcd. for $C_{616}H_{1029}N_{183}O_{184}S_2$ m/z = 14006.6 Da, found 14005.6 Da.

6.2. Synthesis of diacetylated Histone H2B 56

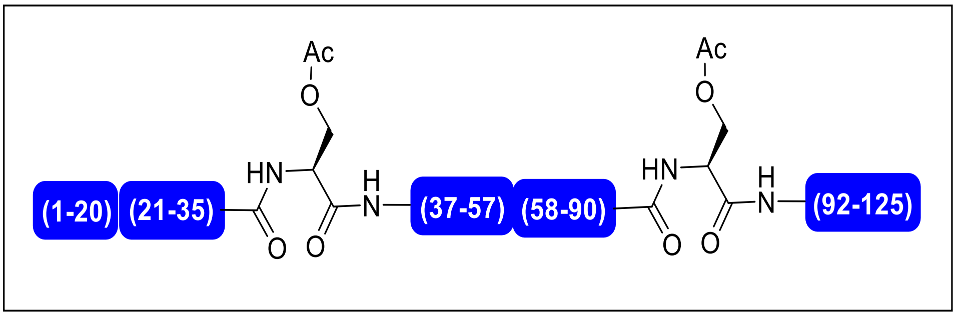

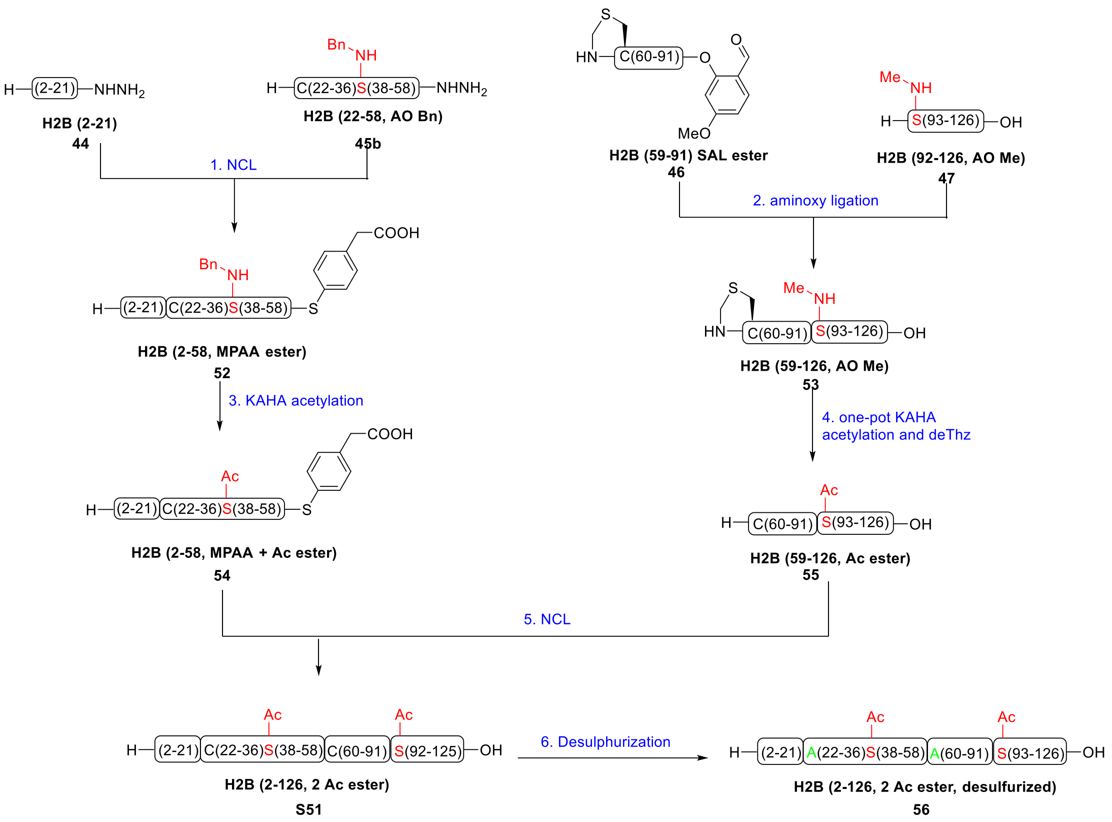

Scheme S14. Synthetic route of **56**.

6.2.1. Synthesis of Histone H2B (22-58, AO Bn) with internal AO(Bn) 45b

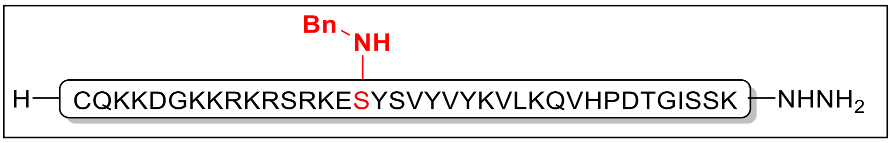

45b

The commercially available 2-chlorotrityl resin at 0.1 mmol scale (CS Biochem, loading: ~0.45 mmol/g) was converted to hydrazine 2-chlorotrityl chloride resin as described in the general procedure for preparation of hydrazine 2-chlorotrityl chloride resin, the resulting resin was employed in the SPPS of Histone H2B (22-58, AO Bn) with internal AO(Bn) according to the standard protocol of SPPS. The crude peptide was purified by preparative reverse-phase HPLC (15-40% CH_3CN/H_2O over 45 min) and lyophilized to afford the desired peptide **45b** (71.6 mg, 19% yield).

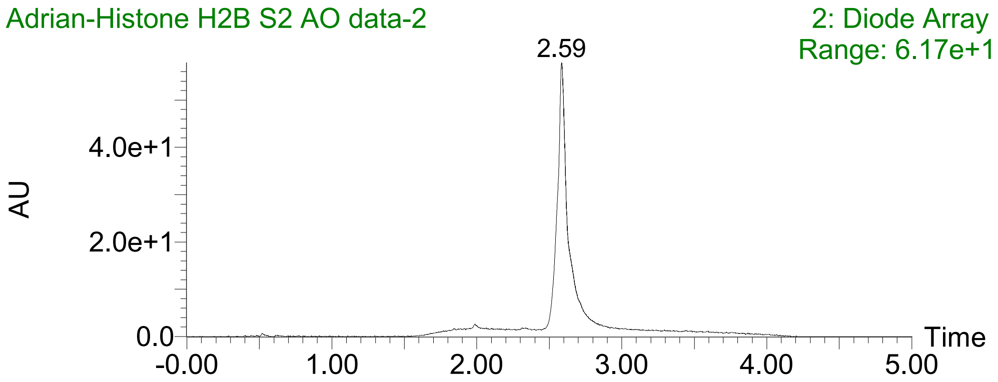

Figure S220. UV (190-400 nm) trace from UPLC-MS analysis of purified Histone H2B (22-58, AO Bn) **45b** with internal AO(Bn) gradient 5-95% CH_3CN/H_2O containing 0.1% TFA over 5 min at a flow rate of 0.4 mL/min.

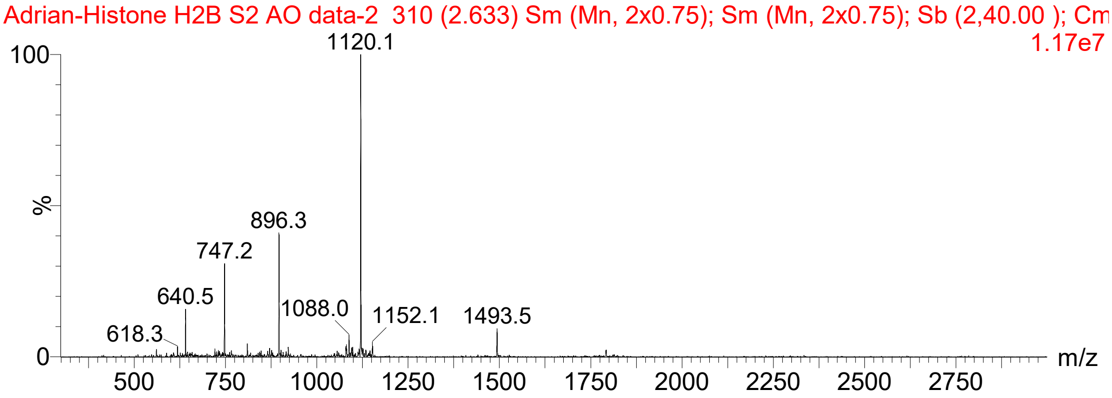

Figure S221. ESI-MS calcd. for $C_{198}H_{330}N_{62}O_{54}S$ $[M+3H]^{3+}$ m/z = 1492.8, found 1493.6; $[M+4H]^{4+}$ m/z = 1119.8, found 1120.1; $[M+5H]^{5+}$ m/z = 896.1, found 896.3; $[M+6H]^{6+}$ m/z = 746.9, found 747.2; $[M+7H]^{7+}$ m/z = 640.3, found 640.6.

6.2.2. Synthesis of Histone H2B (2-58, AO Bn, MPAA ester) 52 by Native Chemical Ligation (NCL)

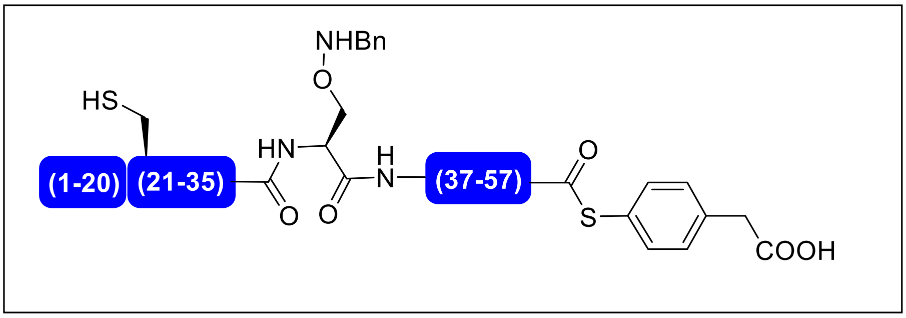

52

Histone H2B (2-58, AO Bn, MPAA ester) **52** was synthesized according to the general Native Chemical Ligation method. Histone H2B (2-21) **44** (15.5 mg, 7.62 μmol) was dissolved in aqueous buffer containing 6 M Guanidine-HCl and 0.2 M NaH_2PO_4 (pH = 3.0) (peptide final concentration is 20 mg/mL). After that, mercaptophenylacetic acid (MPAA) (12.8 mg, 76.2 μmol) and acetylacetone (AcAc) (1.95 μL, 19 μmol) were added into the above mixture. The pH was adjusted to 2 and the reaction mixture was stirred under room temperature for 3 h, then Histone H2B (22-58, AO Bn) **45b** (17 mg, 3.81 μmol) was added into the reaction mixture, and the pH of reaction mixture was then adjusted to 6.8 slowly with aqueous NaOH solution (1M) to initiate the Native Chemical Ligation at room temperature (N-terminal peptide final concentration is 3 mM). After the completion of the reaction, MPAA (12.8 mg, 76.2 μmol) and AcAc (1.95 μL, 19 μmol) were added into the above mixture. The pH was adjusted to 2 and the reaction mixture was stirred under room temperature for 3 h to convert the peptidyl hydrazone to MPAA ester. Purification via preparative reverse phase HPLC (10-60% CH_3CN/H_2O over 45 min, 0.1% TFA) followed by lyophilization afforded Histone H2B (2-58, AO Bn, MPAA ester) **52** (8.6 mg, 34.3% yield) as white solids.

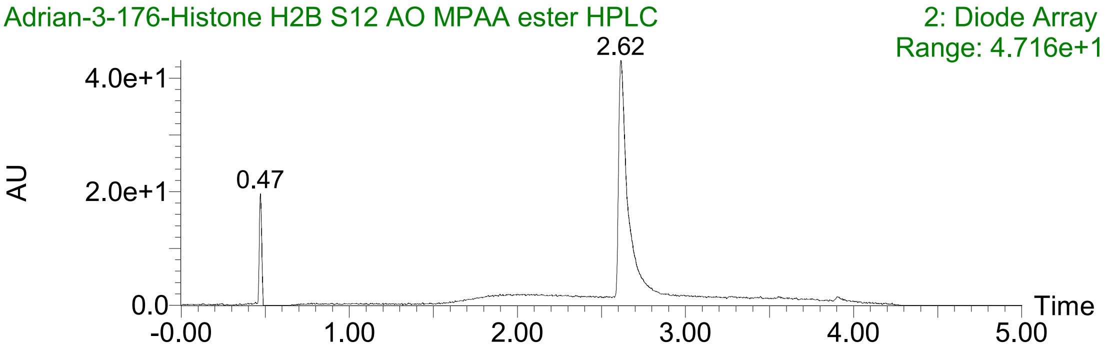

Figure S222. UV (190-400 nm) trace from UPLC-MS analysis of purified Histone H2B (2-58, AO Bn, MPAA ester) **52** gradient 5-95% CH_3CN/H_2O containing 0.1% TFA over 5 min at a flow rate of 0.4 mL/min.

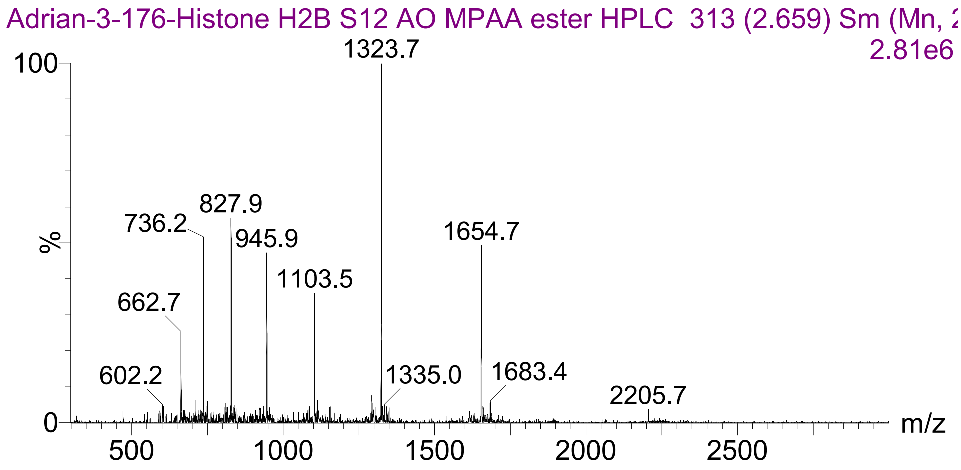

Figure S223. ESI-MS calcd. for $C_{296}H_{490}N_{86}O_{81}S_2$ $[M+4H]^{4+}$ m/z = 1654.5, found 1654.7; $[M+5H]^{5+}$ m/z = 1323.7, found 1323.7; $[M+6H]^{6+}$ m/z = 1103.3, found 1103.5; $[M+7H]^{7+}$ m/z = 945.8, found 945.9; $[M+8H]^{8+}$ m/z = 827.7, found 827.9; $[M+9H]^{9+}$ m/z = 735.9, found 736.2; $[M+10H]^{10+}$ m/z = 662.4, found 662.7; $[M+11H]^{11+}$ m/z = 602.3, found 602.2

6.2.3. Synthesis of Histone H2B (59-126, AO Me) 53 by aminooxy ligation

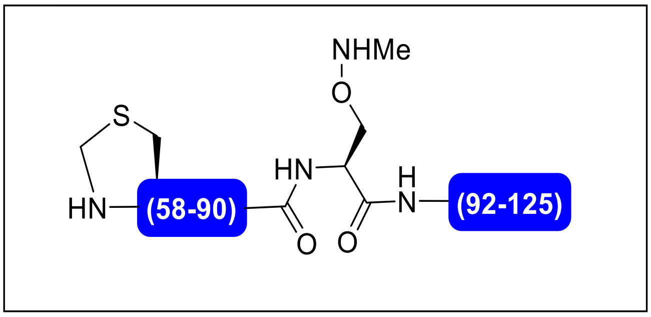

53

Histone H2B (59-126, AO Me) **53** was synthesized according to the general aminooxy ligation method. Histone H2B (59-91) SAL ester (34.7 mg, 8.9 μmol) and Histone H2B (92-126, AO, Me) (67.7 mg, 17.8 μmol) were incubated in 0.5 M sodium citrate with 6M Guanidine-HCl aqueous buffer at pH 4.5, 25 °C for overnight. Purification via preparative reverse phase HPLC (25-75% CH_3CN/H_2O over 45 min, 0.1% TFA) followed by lyophilization afforded Histone H2B (59-126, AO) **53** (19.2 mg, 28.6% yield) as white solids.

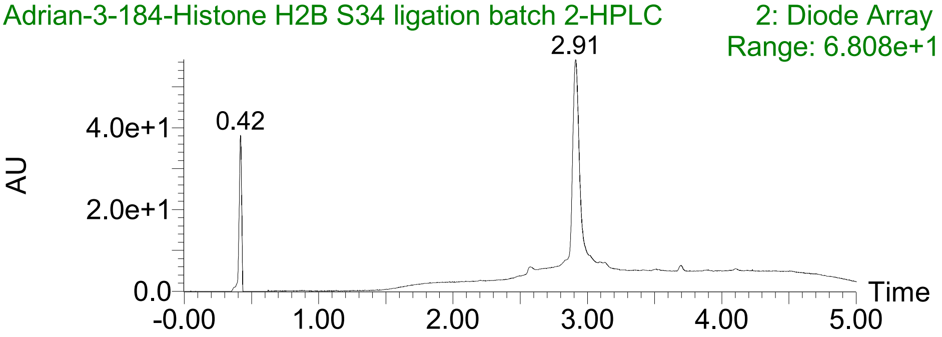

Figure S224. UV (190-400 nm) trace from UPLC-MS analysis of purified Histone H2B (59-126, AO Me) **53** gradient 5-95% CH_3CN/H_2O containing 0.1% TFA over 5 min at a flow rate of 0.4 mL/min.

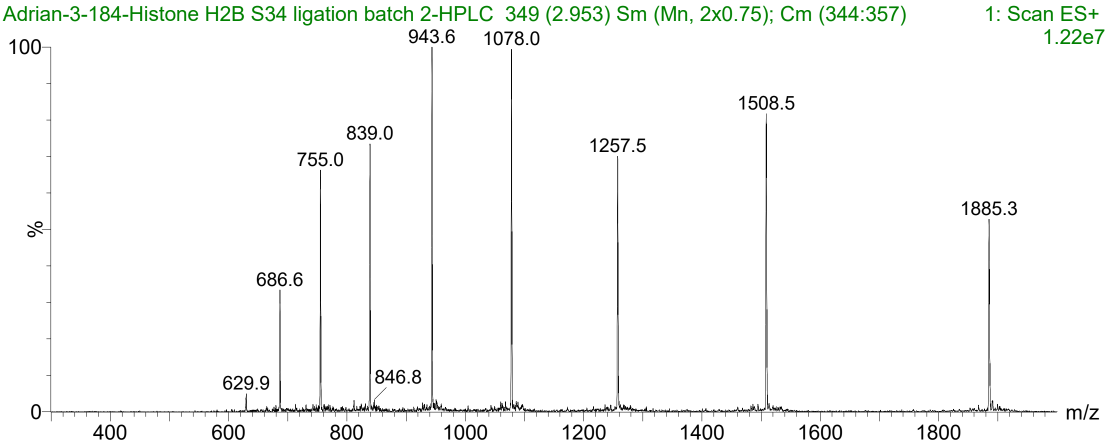

Figure S225. ESI-MS calcd. for $C_{328}H_{541}N_{97}O_{100}S_3$ $[M+4H]^{4+}$ m/z = 1884.74, found 1885.3; $[M+5H]^{5+}$ m/z = 1508.0, found 1508.5; $[M+6H]^{6+}$ m/z = 1256.8, found 1257.5; $[M+7H]^{7+}$ m/z = 1077.4, found 1078.0; $[M+8H]^{8+}$ m/z = 942.9, found 943.6; $[M+9H]^{9+}$ m/z = 838.2, found 839.0; $[M+10H]^{10+}$ m/z = 754.5, found 755.0; $[M+11H]^{11+}$ m/z = 686.0, found 686.6; $[M+12H]^{12+}$ m/z = 628.9, found 629.9.

6.2.4. Synthesis of Histone H2B (2-58, AO Bn, MPAA ester, desulfurized) by Add-and-Done Desulfurization

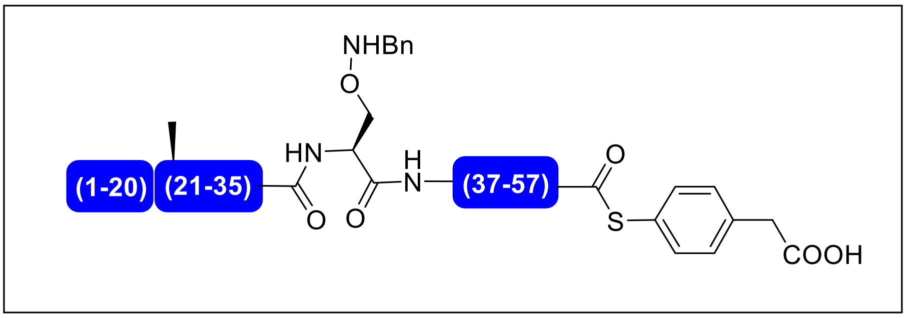

TCEP·HCl (0.2 M) was dissolved in a tube by 0.5 M sodium citrate with 6M Guanidine-HCl, and the pH of this solution would be adjusted to between 4.0~5.0 by addition of NaOH (1M). After that, Histone H2B (2-58, AO Bn, MPAA ester) **52** (8.61 mg, 1.3 μmol) was added to the above solution with the final concentration of 1.0 mM. As follows, freshly weighted $NaBEt_4$ solid was added to the substrate solution at the final concentration of 0.1 M. After few minutes

of shaking, another portion of $NaBEt_4$ solid was added at the final concentration of 0.15 M for complete desulfurization. Purification via preparative reverse phase HPLC (25-75% CH_3CN/H_2O over 45 min, 0.1% TFA) followed by lyophilization afforded Histone H2B (2-58, AO Bn, MPAA ester, desulfurized) (4.53mg, 52.9% yield) as white solids.

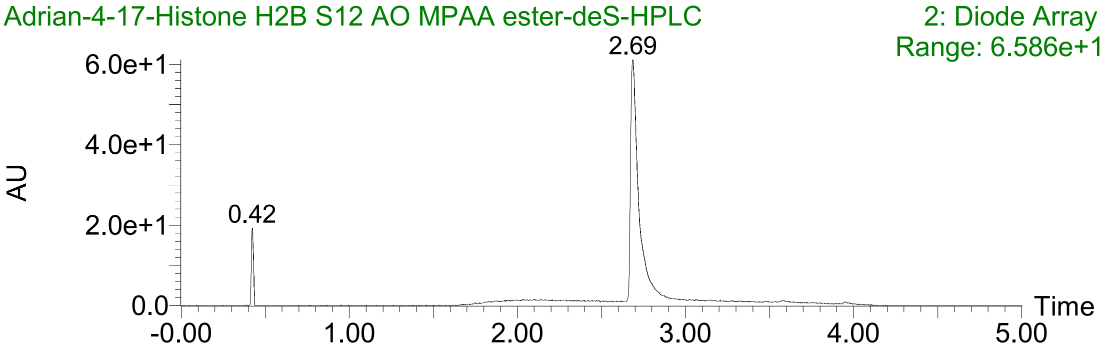

Figure S226. UV (190-400 nm) trace from UPLC-MS analysis of purified Histone H2B (2-58, AO Bn, MPAA ester, desulfurized) gradient 5-95% CH_3CN/H_2O containing 0.1% TFA over 5 min at a flow rate of 0.4 mL/min.

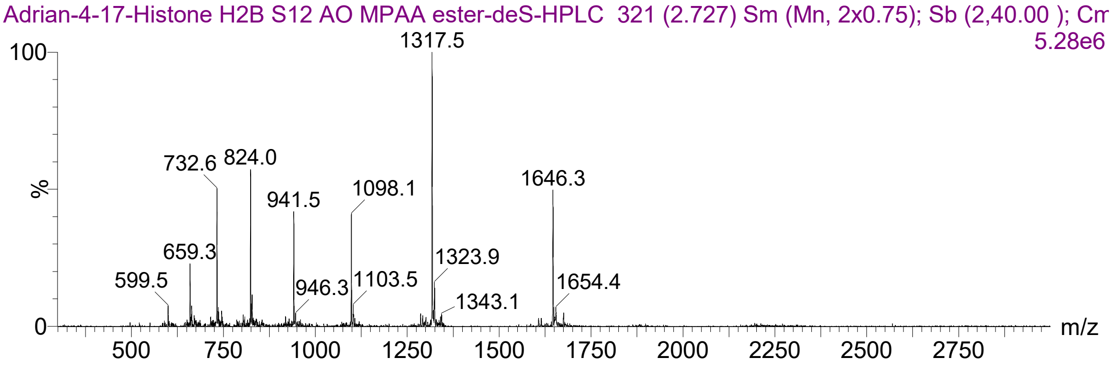

Figure S227. ESI-MS calcd. for $C_{296}H_{490}N_{86}O_{81}S$ $[M+4H]^{4+}$ m/z = 1646.4, found 1646.3; $[M+5H]^{5+}$ m/z = 1317.4, found 1317.5; $[M+6H]^{6+}$ m/z = 1098.0, found 1098.1; $[M+7H]^{7+}$ m/z = 941.2, found 941.5; $[M+8H]^{8+}$ m/z = 823.7, found 824.0; $[M+9H]^{9+}$ m/z = 732.3, found 732.6; $[M+10H]^{10+}$ m/z = 659.2, found 659.3; $[M+11H]^{11+}$ m/z = 599.3, found 599.5

6.2.5. Synthesis of Histone H2B (2-58, MPAA + Ac ester) 54

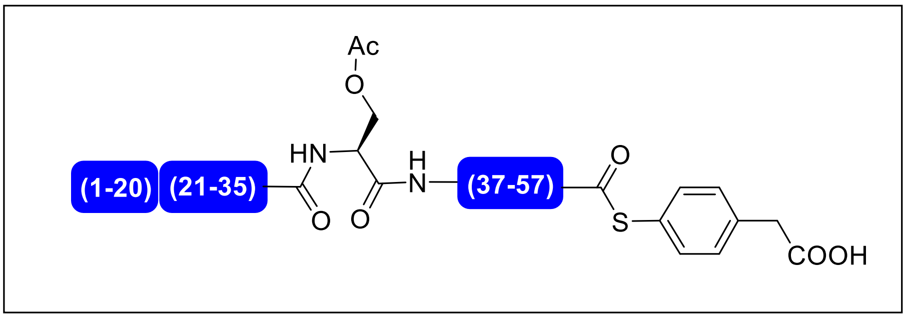

54

Histone H2B (2-58, MPAA + Ac ester) **54** was synthesized according to the general CEL method. Histone H2B (2-58, AO Bn, MPAA ester, desulfurized) (20 mg, 3.04 μmol) was incubated with pyruvic acid (1.08 μL, 15.2 μmol) in aqueous DMSO with 0.01M oxalic acid at 40 mM, 75°C for 1.5 h. The crude peptide was purified by preparative reverse-phase HPLC (21-26% CH_3CN/H_2O over 45 min) and lyophilized to afford the desired Ac ester **54** (6.45 mg, 32.6 % yield).

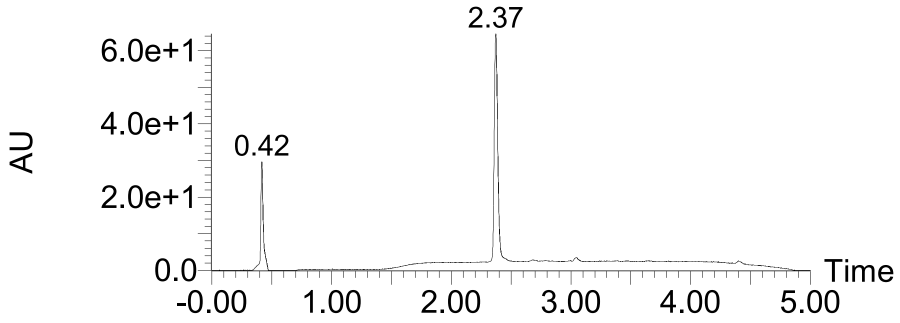

Figure S228. UV (190-400 nm) trace from UPLC-MS analysis of purified Histone H2B (2-58 MPAA + Ac ester) **54** gradient 5-95% CH_3CN/H_2O containing 0.1% TFA over 5 min at a flow rate of 0.4 mL/min.

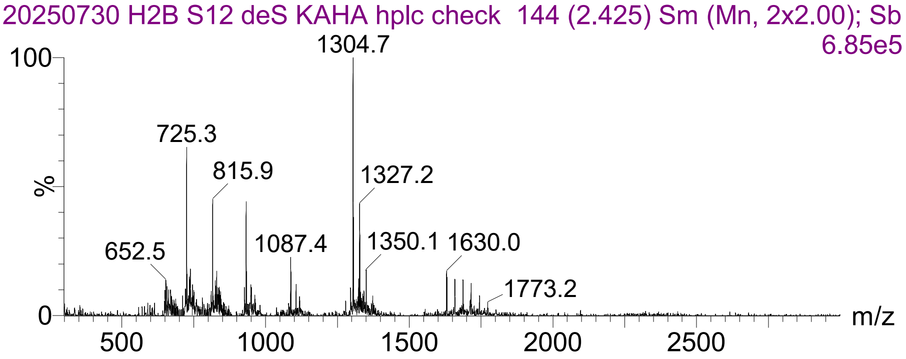

Figure S229. ESI-MS calcd. for $C_{291}H_{485}N_{85}O_{82}S$ $[M+4H]^{4+}$ m/z = 1630.7, found 1630.0; $[M+5H]^{5+}$ m/z = 1304.7, found 1304.7; $[M+6H]^{6+}$ m/z = 1087.4, found 1087.4; $[M+7H]^{7+}$ m/z = 932.2, found 932.4; $[M+8H]^{8+}$ m/z = 815.8, found 815.9; $[M+9H]^{9+}$ m/z = 725.3, found 725.3; $[M+10H]^{10+}$ m/z = 652.9, found 652.5.

6.2.6. Synthesis of Histone H2B (59-126, Ac ester) 55

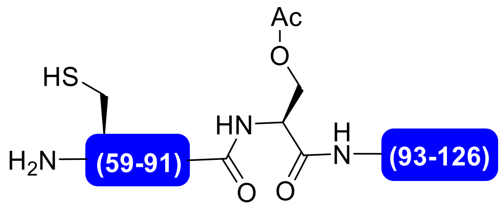

H2B (59-126, Ac ester)

Histone H2B (59-126, Ac ester) **55** was synthesized according to the general CEL method. Histone H2B (59-126, AO Me) (19.3 mg, 2.56 μmol) was incubated with pyruvic acid (0.90 μL, 12.8 μmol) in aqueous DMSO with 0.01 M oxalic acid and 0.1 M DMS at 20 mM, 60°C for 2 h. After the completion of reaction, the reaction crude was precipitated in ether and redissolved in 6M Guanidine-Citrate buffer premixed with 0.1M TCEP and 0.5 M $MeONH_2$ and adjusted to pH 4 at 5 mM. The reaction was incubated at 25°C for 4 h and was purified by preparative reverse-phase HPLC (30-55% CH_3CN/H_2O over 45 min) and lyophilized to afford the desired Ac ester **55** (8.35 mg, 43.3% yield).

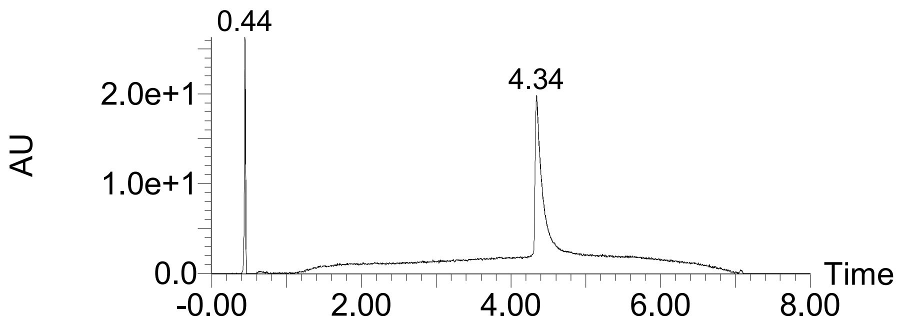

Figure S230. UV (190-400 nm) trace from UPLC-MS analysis of purified Histone H2B (59-126, Ac ester) **55** gradient 5-95% CH_3CN/H_2O containing 0.1% TFA over 8 min at a flow rate of 0.4 mL/min.

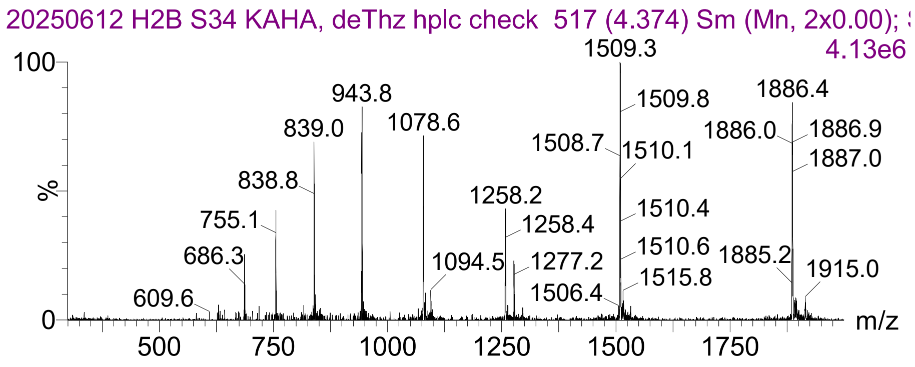

Figure S231. ESI-MS calcd. for $C_{328}H_{540}N_{96}O_{101}S_3$ $[M+4H]^{4+}$ m/z = 1886.2, found 1886.4; $[M+5H]^{5+}$ m/z = 1509.1, found 1509.3; $[M+6H]^{6+}$ m/z = 1257.8, found 1258.2; $[M+7H]^{7+}$ m/z = 1078.2, found 1078.6; $[M+8H]^{8+}$ m/z = 943.6, found 943.8; $[M+9H]^{9+}$ m/z = 838.9, found 839.0; $[M+10H]^{10+}$ m/z = 755.1, found 755.1; $[M+11H]^{11+}$ m/z = 686.5, found 686.3.

6.2.7. Synthesis of Histone H2B (2-126, 2 Ac ester) S51 by Native Chemical Ligation

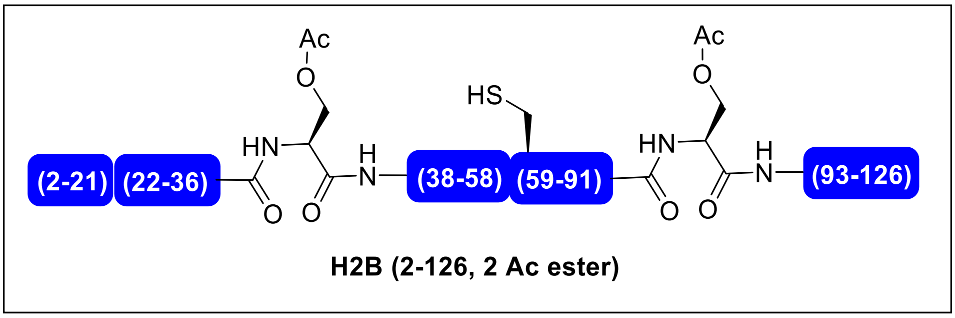

S51

Histone H2B (2-126, 2 Ac ester) **S51** was synthesized according to the general Native Chemical Ligation method. Histone H2B (2-58, MPAA + Ac ester) (1.82 mg, 0.28 μmol) **54** was dissolved in aqueous buffer containing 6 M Guanidine-HCl and 0.2 M NaH_2PO_4 (pH = 7.0) (peptide final concentration is 3 mM). After that, MPAA (1.86 mg, 11.1 μmol) and Histone H2B (59-126, Ac ester) **55** (3.13 mg, 0.42 μmol) was added into the reaction mixture, and the pH of reaction mixture was then adjusted to 6.8 slowly with aqueous NaOH solution (1M) to initiate the Native Chemical Ligation at room temperature. After the completion of the reaction, purification via preparative reverse phase HPLC (25-75% CH_3CN/H_2O over 45 min, 0.1% TFA) followed by lyophilization afforded Histone H2B (2-126, 2 Ac ester) **S51** (1.69 mg, 44% yield) as white solids.

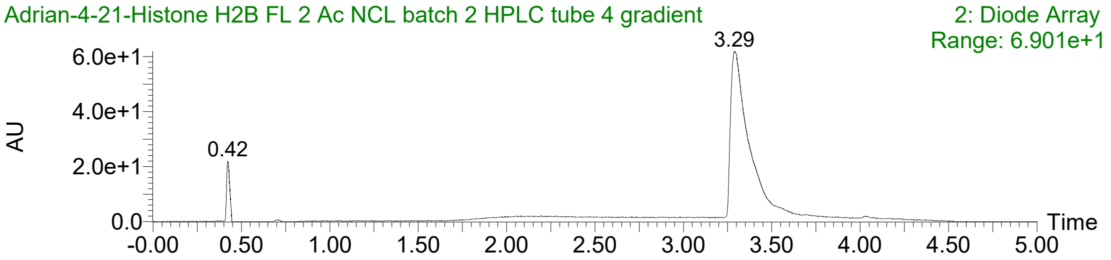

Figure S232. UV (190-400 nm) trace from UPLC-MS analysis of purified Histone H2B (2-126, 2 Ac ester) **S51** gradient 5-95% CH_3CN/H_2O containing 0.1% TFA over 5 min at a flow rate of 0.4 mL/min.

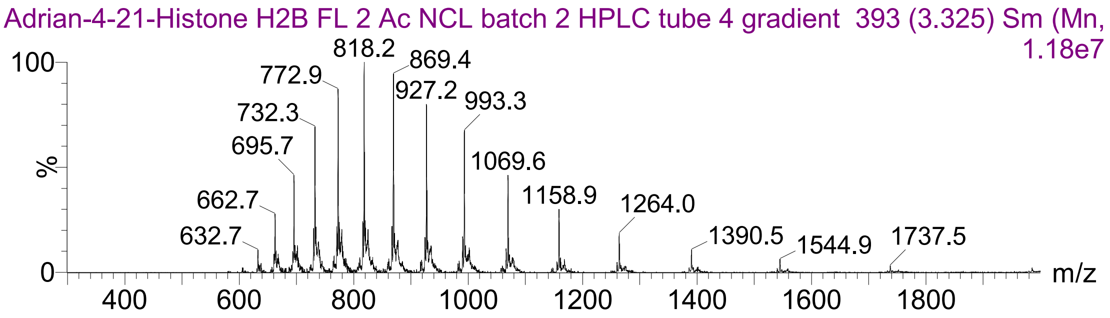

Figure S233. ESI-MS calcd. for $C_{611}H_{1017}N_{181}O_{181}S_3$ $[M+8H]^{8+}$ m/z = 1737.4, found 1737.5; $[M+9H]^{9+}$ m/z = 1544.5, found 1544.9; $[M+10H]^{10+}$ m/z = 1390.1, found 1390.5; $[M+11H]^{11+}$ m/z = 1263.8, found 1264.0; $[M+12H]^{12+}$ m/z = 1158.6, found 1158.9; $[M+13H]^{13+}$ m/z = 1069.5, found 1069.6; $[M+14H]^{14+}$ m/z = 993.2, found 993.3; $[M+15H]^{15+}$ m/z = 927.1, found 927.2; $[M+16]^{16+}$ m/z = 869.2, found 869.4; $[M+17H]^{17+}$ m/z = 818.1, found 818.2; $[M+18H]^{18+}$ m/z = 772.7, found 772.9; $[M+19H]^{19+}$ m/z = 732.1, found 732.3; $[M+20H]^{20+}$ m/z = 695.6, found 695.7; $[M+21H]^{21+}$ m/z = 662.5, found 662.7; $[M+22H]^{22+}$ m/z = 632.4, found 632.7.

6.2.8. Synthesis of Histone H2B (2-126, 2 Ac ester, desulfurized) 56 by Add-and-Done Desulfurization

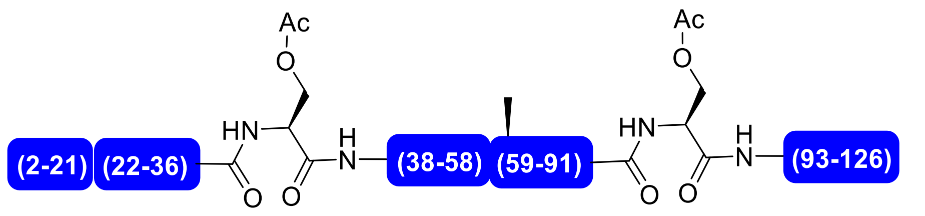

H2B (2-126, 2 Ac ester, desulfurized)

56

TCEP·HCl (0.2 M) was dissolved in a tube by 0.5 M sodium citrate with 6M Guanidine-HCl, and the pH of this solution would be adjusted to between 4.0~5.0 by addition of NaOH (1M). After that, Histone H2B (2-126, 2 Ac ester) **56** (1.69 mg, 0.12 μmol) was added to the above solution with the final

concentration of 1.0 mM. As follows, freshly weighted $NaBEt_4$ solid was added to the substrate solution at the final concentration of 0.1 M. After few minutes of shaking, another portion of $NaBEt_4$ solid was added at the final concentration of 0.15 M for complete desulfurization. Purification via preparative reverse phase HPLC (25-75% CH_3CN/H_2O over 45 min, 0.1% TFA) followed by lyophilization afforded Histone H2B (2-126, 2 Ac ester, desulfurized) **56** (0.36 mg, 21.4% yield) as white solids.

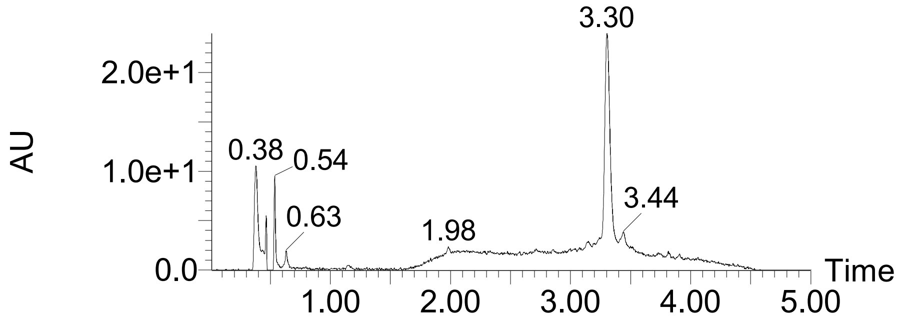

Figure S234. UV (190-400 nm) trace from UPLC-MS analysis of purified Histone H2B (2-126, 2 Ac ester, desulfurized) **56** gradient 5-95% CH_3CN/H_2O containing 0.1% TFA over 5 min at a flow rate of 0.4 mL/min.

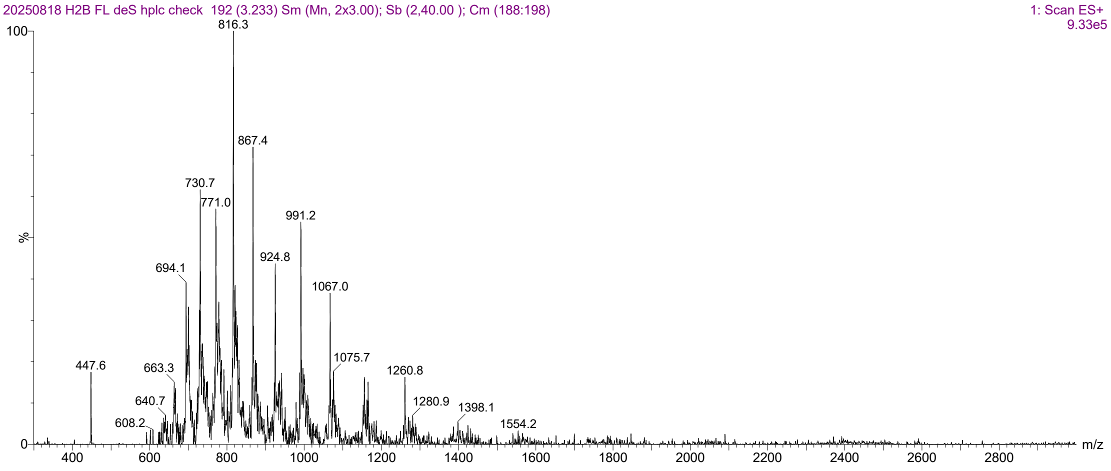

Figure S235. ESI-MS calcd. for $C_{611}H_{1017}N_{181}O_{181}S_2$ $[M+11H]^{11+}$ m/z = 1260.9, found 1260.8; $[M+12H]^{12+}$ m/z = 1155.9, found 1155.6; $[M+13H]^{13+}$ m/z = 1067.1, found 1067.0; $[M+14H]^{14+}$ m/z = 990.9, found 991.2; $[M+15H]^{15+}$

m/z = 924.9, found 924.8; $[M+16]^{16+}$ m/z = 867.2, found 867.4; $[M+17H]^{17+}$ m/z = 816.2, found 816.3; $[M+18H]^{18+}$ m/z = 770.9, found 771.0; $[M+19H]^{19+}$ m/z = 730.4, found 730.7; $[M+20H]^{20+}$ m/z = 694.0, found 694.1; $[M+21H]^{21+}$ m/z = 661.0, found 663.3; $[M+22H]^{22+}$ m/z = 631.0, found 631.3.

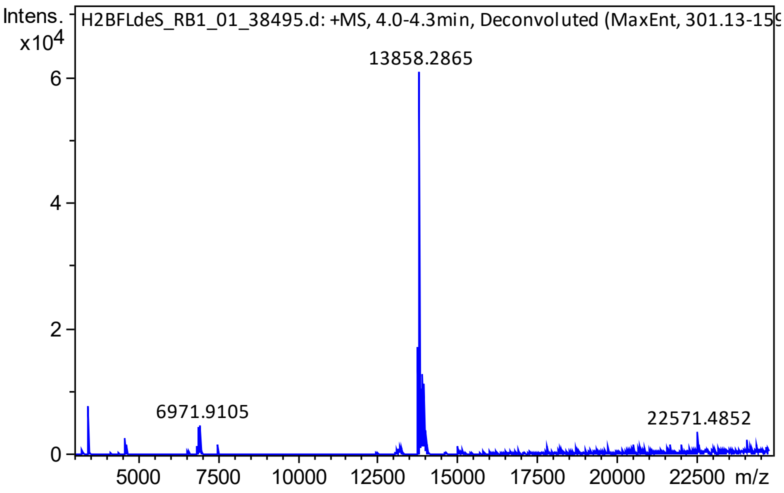

Figure S236. ESI-HRMS calcd. for $C_{611}H_{1017}N_{181}O_{181}S_2$ m/z = 13858.6 Da, found 13858.3 Da.

7. Mechanistic study of AOL and CEL

7.1. Capture of six-member ring intermediate

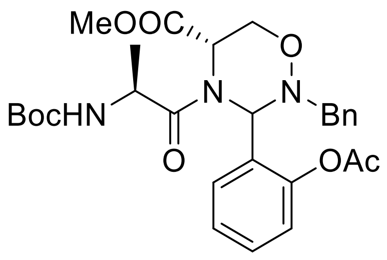

61

To a solution of compound **6a** (41 mg, 0.1 mmol, 1.0 equiv.) and $KHCO_3$ (15 mg, 0.15 mmol, 1.5 equiv.) dissolved in DMF (1 mL) was added iodomethane (7.5 μL, 0.12 mmol, 1.2 equiv.), the reaction mixture was stirred at room temperature for overnight. The solution was then diluted with EtOAc (20.0 mL) and the organic phase washed with 1 N HCl (10.0 mL) x2 and brine subsequently. The organic layer was dried with sodium sulfate and removed by reduced pressure evaporation to give the crude methyl ester which was used directly without purification. Trifluoroacetic acid (5 mL) was added to the crude product at room temperature and was stirred at room temperature for 0.5 h. After that, the trifluoroacetic acid was removed under reduced pressure and diluted with water (10 mL). The aqueous layer washed with Et_2O (10.0 mL) x 3 and was lyophilized.

The lyophilized crude product **58** was dissolved in DCM (1 mL) with 10% Pyridine-acetic acid 6:1 buffer and Boc-Ala-SAL ester **57** (29 mg, 0.1 mmol, 1.0 equiv.) was added, the reaction was then stirred at room temperature for overnight. Ac_2O and DIEA was then added subsequently to the reaction mixture and stirred for another 3 h. DCM was then blown away with compressed air and the reaction mixture was diluted with H_2O/ACN and purified by preparative reverse-phase HPLC (45-85% CH_3CN/H_2O over 45 min) and lyophilized to afford the desired intermediate **61** (14.0 mg, 25.8% yield).

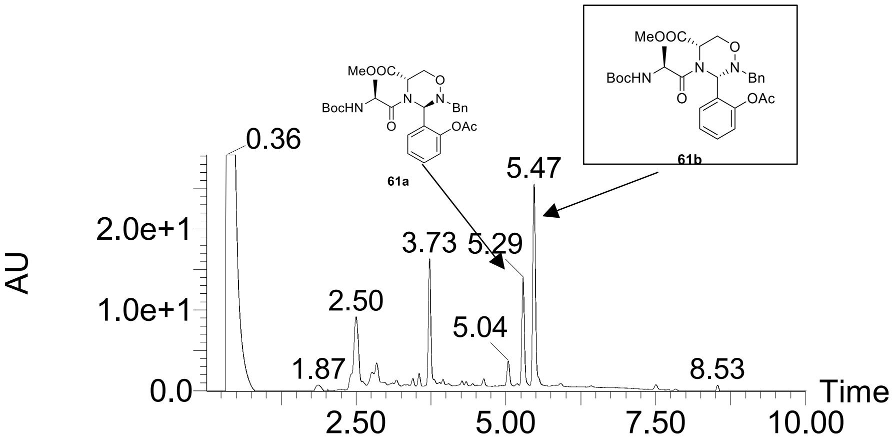

Figure S237. UV (190-400 nm) trace from UPLC-MS analysis of synthesis of aminooxy dipeptide **61a-b** gradient 45-85% CH_3CN/H_2O containing 0.1% TFA over 10 min at a flow rate of 0.4 mL/min. A pair of diastereomer existed at the acetal center.

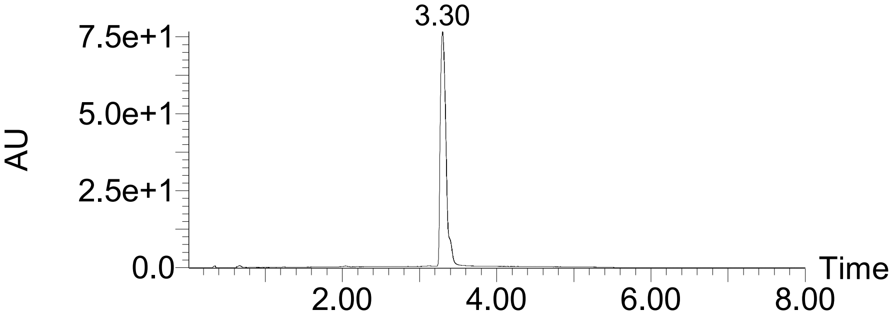

Figure S238. UV (190-400 nm) trace from UPLC-MS analysis of purified aminooxy dipeptide **61b** gradient 45-85% CH_3CN/H_2O containing 0.1% TFA over 8 min at a flow rate of 0.4 mL/min.

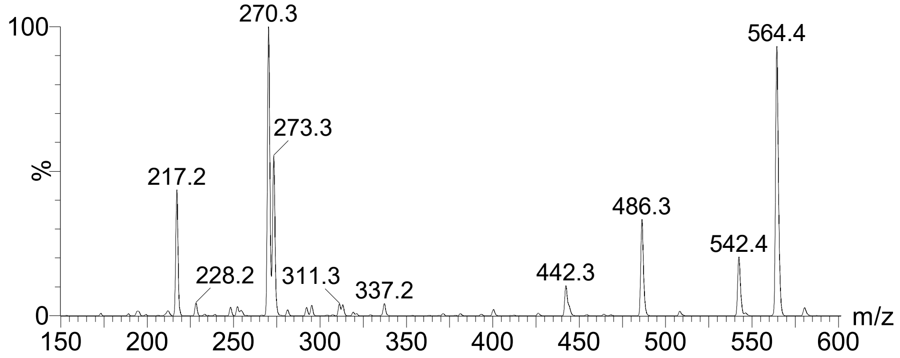

Figure S239. ESI-MS calcd. for $C_{28}H_{35}N_3O_8$ $[M+H]^+$ m/z = 542.6, found 542.4.

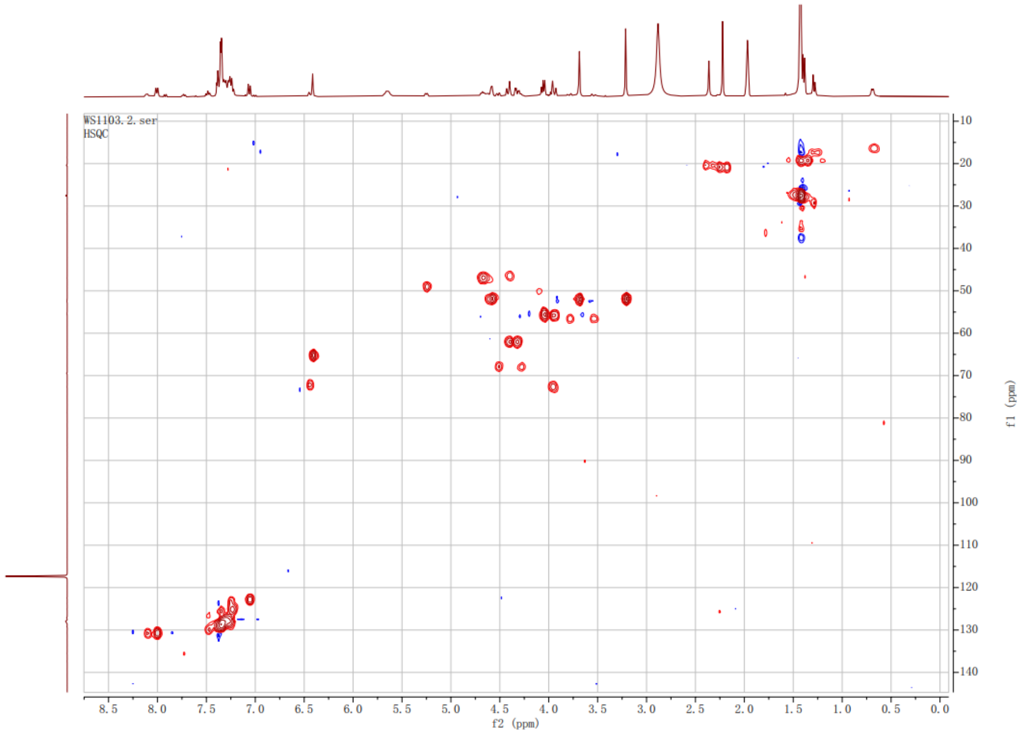

Figure S240. HSQC spectrum of **61b**

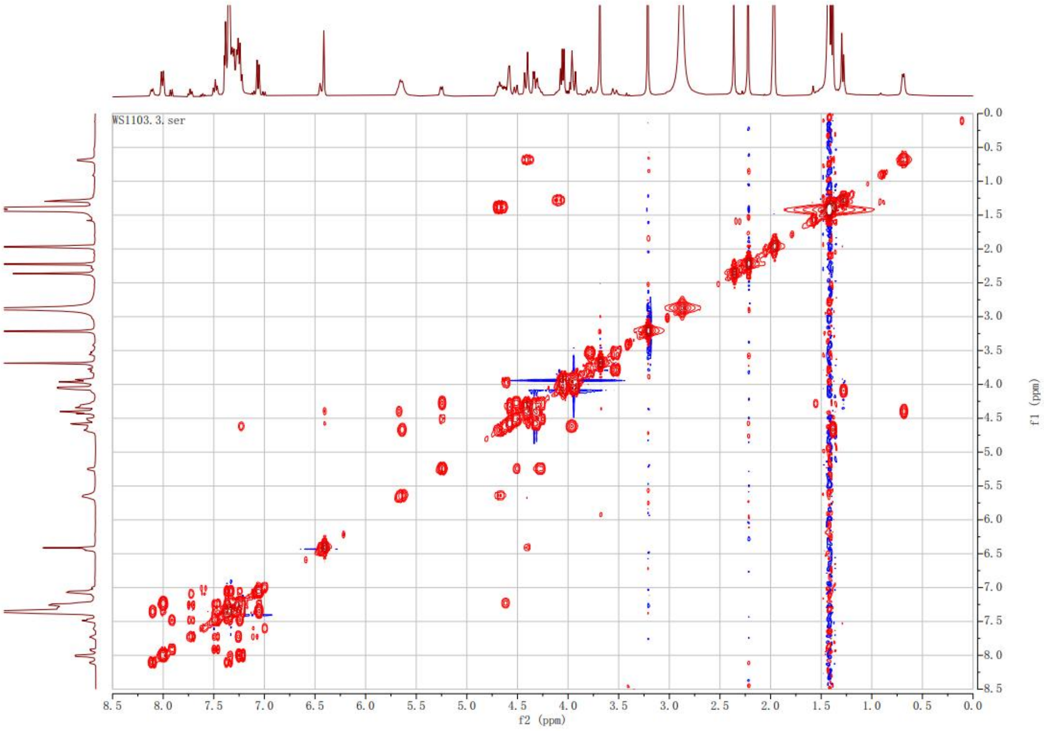

Figure S241. NOSEY spectrum of **61b**

7.2. Investigation of AOL pathway

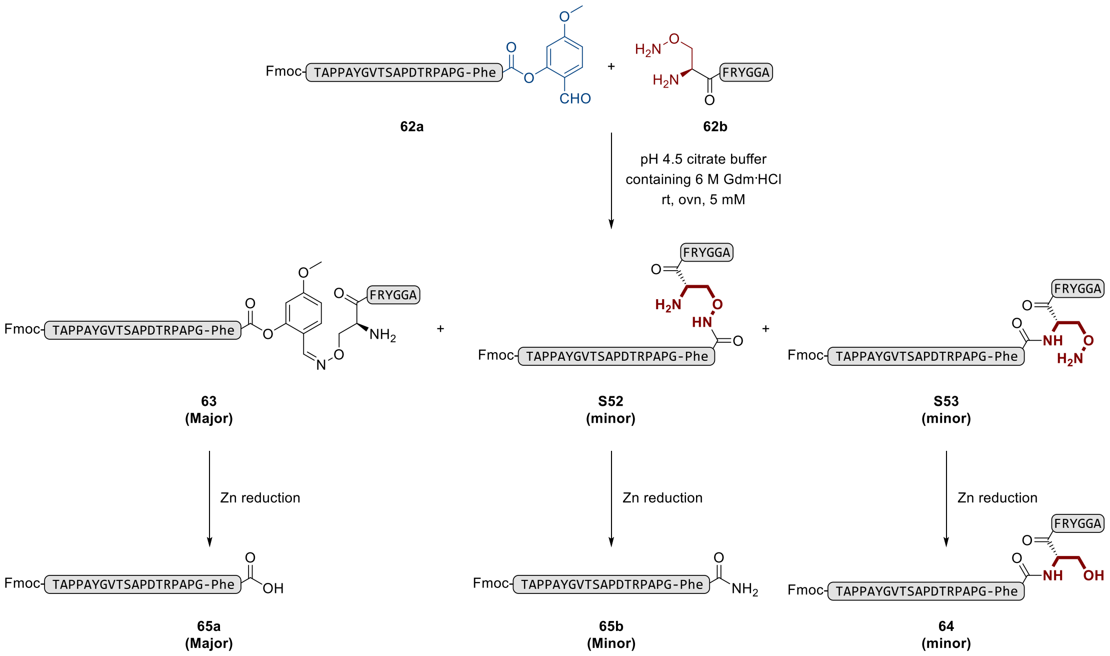

Figure S242. AOL pathway investigation.

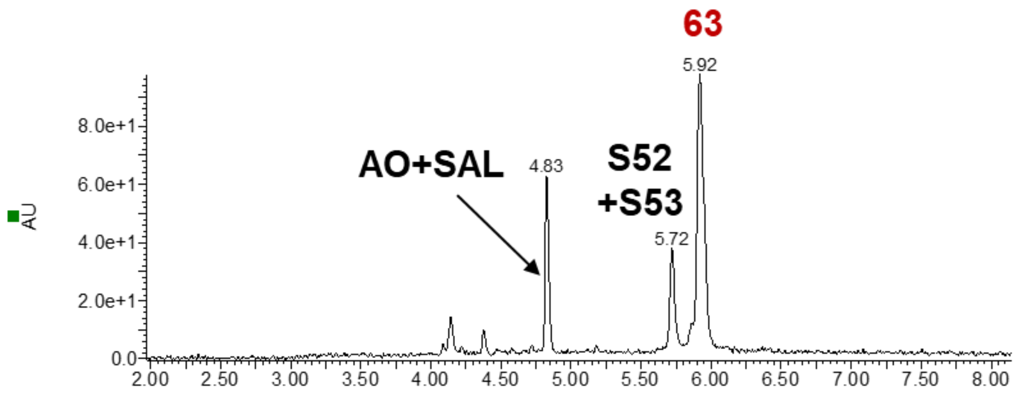

Figure S243. Reaction monitoring by UPLC-MS of AOL between **62a** and **62b** gradient 45-85% CH_3CN/H_2O containing 0.1% TFA over 10 min at a flow rate of 0.4 mL/min. Oxime product **63** was observed in major form.

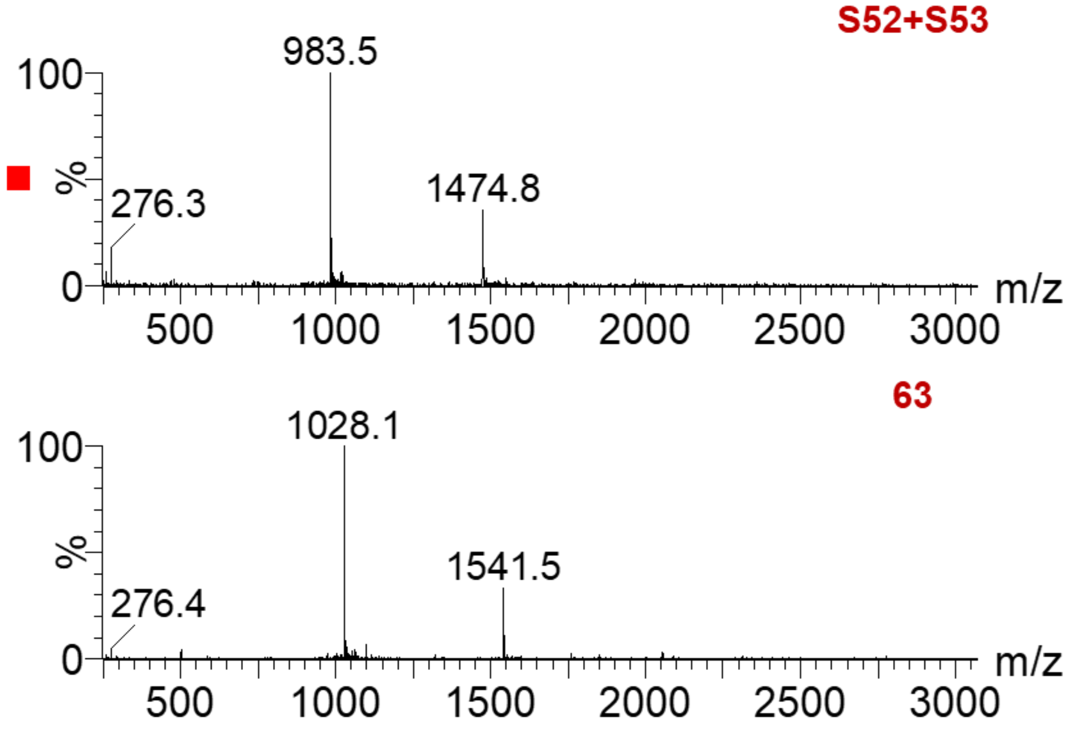

Figure S244. ESI-MS spectra of **63** (bottom). ESI-MS calcd. for C146H197N35O40 $[M+2H]^{2+}$ m/z = 1542.2, found 1541.5; $[M+3H]^{3+}$ m/z = 1028.4, found 1028.1. ESI-MS spectra of **S52** and **S53** (top). ESI-MS calcd. for C138H191N35O38 $[M+2H]^{2+}$ m/z = 1475.1, found 1474.8; $[M+3H]^{3+}$ m/z = 983.7, found 983.5.

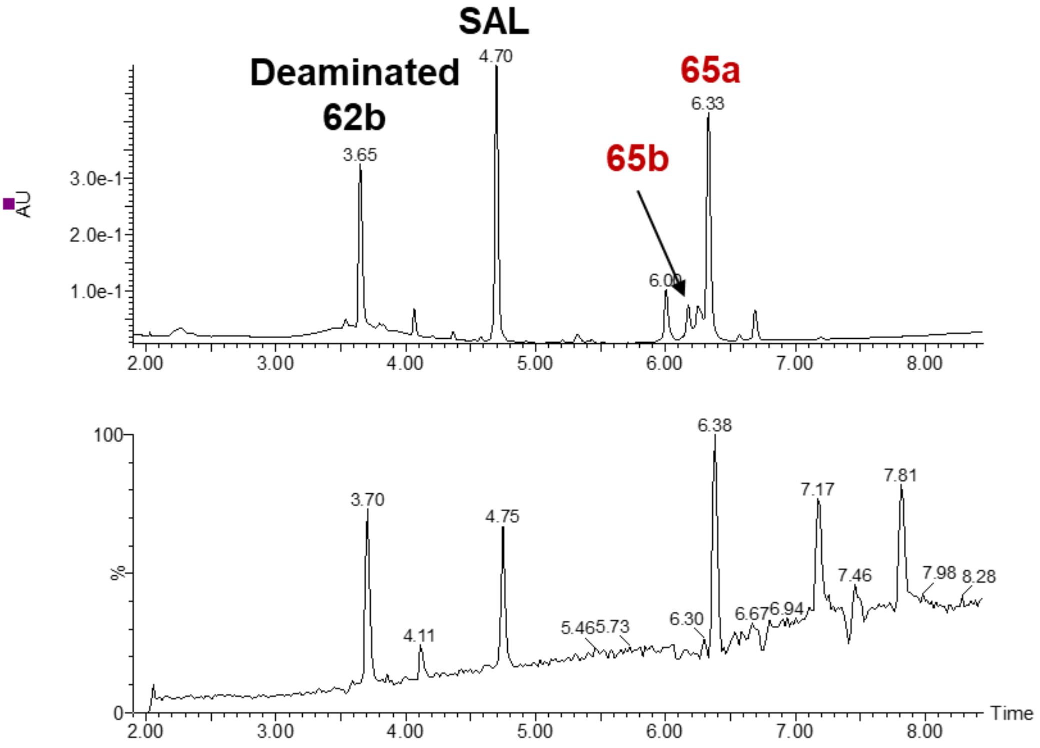

Figure S245. Reaction monitoring by UPLC-MS of Zinc reduction of AOL crude. Gradient 45-85% CH_3CN/H_2O containing 0.1% TFA over 10 min at a flow rate of 0.4 mL/min.

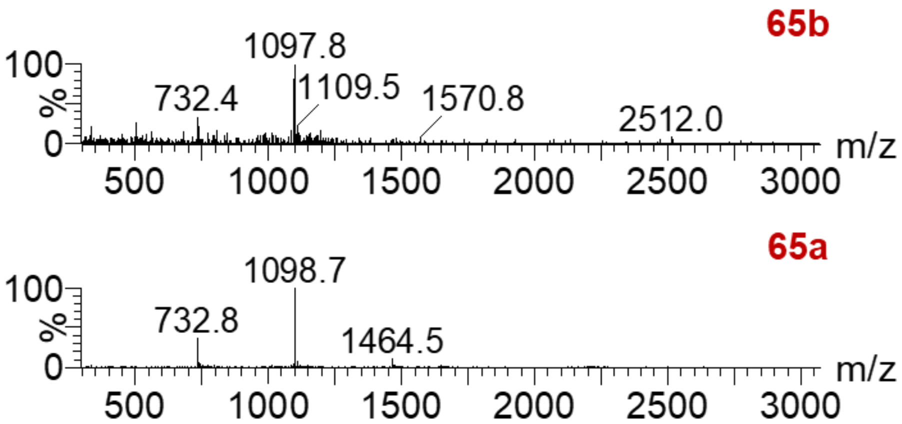

ESI-MS spectra of **65a** (bottom). ESI-MS calcd. for C104H143N23O30 $[M+2H]^{2+}$ m/z = 1098.0, found 1097.8; $[M+3H]^{3+}$ m/z = 732.4, found 732.4. ESI-MS spectra of **65b** (top). ESI-MS calcd. for C104H144N24O29 $[M+2H]^{2+}$ m/z = 1097.5, found 1097.8; $[M+3H]^{3+}$ m/z = 732.0, found 732.4.

7.3. Mechanistic study of CEL

7.3.1. Dipeptide model

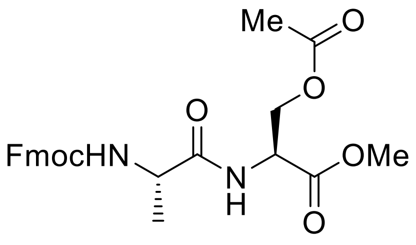

S54

Fmoc-Ala-Ser(Ac)-OMe **S54** was synthesized according to the general CEL procedure. Fmoc-Ala-AO(Bn)-OMe (2.2 mg, 3.48 μmol) was incubated with pyruvic acid (0.61 μL, 8.71 μmol) in aqueous DMSO with 0.01M oxalic acid at 40 mM, 60°C for 3 h. The crude peptide was purified by preparative reverse-phase HPLC (35-75% CH_3CN/H_2O over 45 min) and lyophilized to afford the desired Ac ester **S54** (0.3 mg, 19.0% yield).

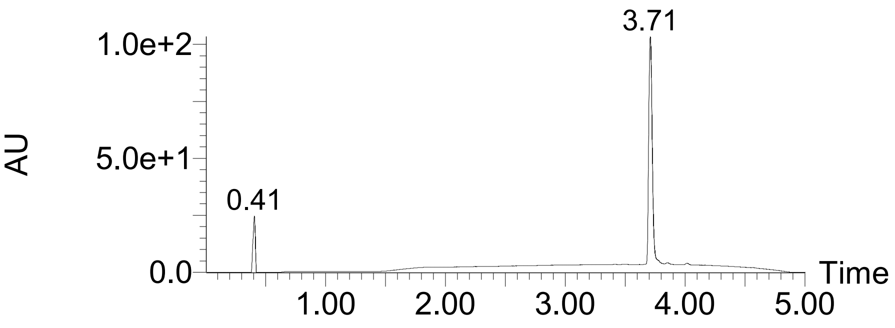

Figure S246. UV (190-400 nm) trace from UPLC-MS analysis of purified Fmoc-Ala-Ser(Ac)-OMe gradient 5-95% CH_3CN/H_2O containing 0.1% TFA over 5 min at a flow rate of 0.4 mL/min.

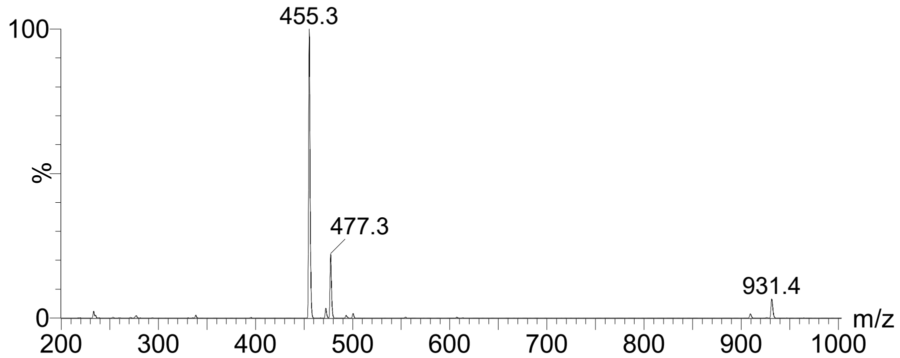

Figure S247. ESI-MS calcd. for $C_{24}H_{26}N_2O_7$ $[M+H]^+$ m/z = 455.5, found 455.3.

7.3.2. Isotope labeling of ester product with $H_2{}^{18}O$ under CEL conditions

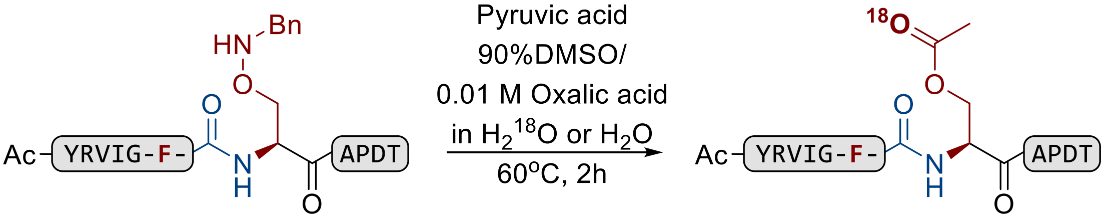

Scheme S15. Isotopic labeling of **16** under CEL conditions with $H_2{}^{18}O$.

Following the general CEL method, Ac-YRVIGF(AO, Bn)APDT-NH_2 **16** (1.0 equiv.) was incubated with different keto acid (5.0 equiv.) in aqueous DMSO with 0.01M oxalic acid dissolved either in $H_2{}^{18}O$ or H_2O at 40 mM, 60°C over 2 h time course. Small amount of reaction mixture was aliquot and was analyzed by UPLC-MS.

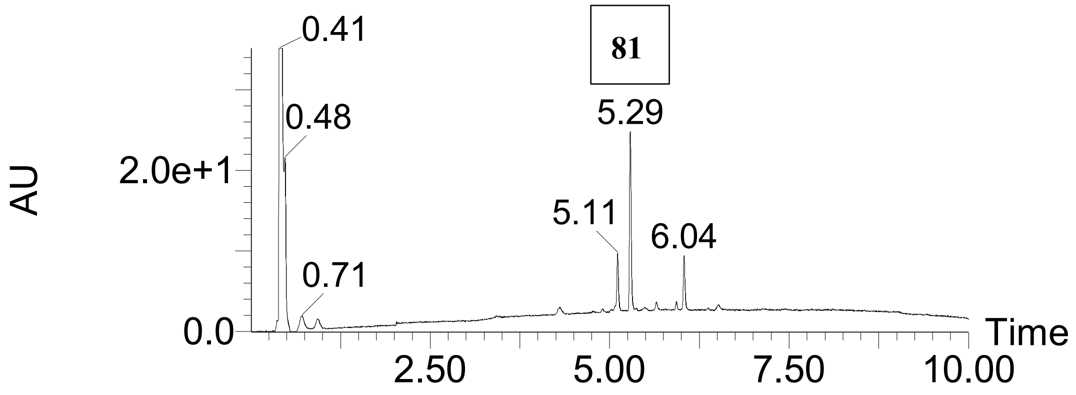

Figure S248. UV (190-400 nm) trace from UPLC-MS analysis of reaction crude of CEL modification in $H_2{}^{18}O$ at 2 h. Gradient 5-75% CH_3CN/H_2O containing 0.1% TFA over 10 min at a flow rate of 0.4 mL/min.

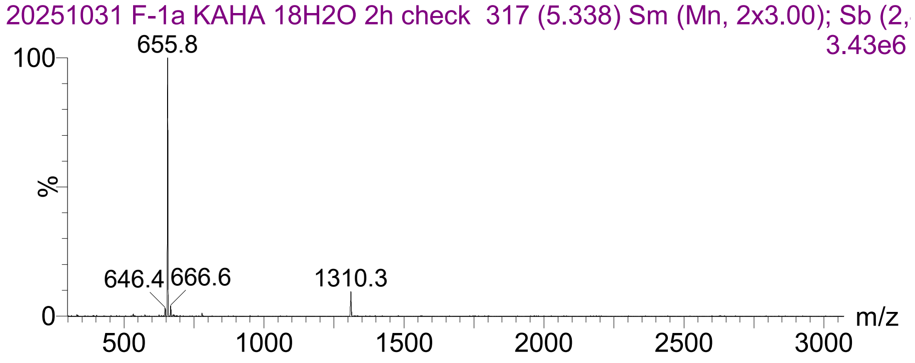

Figure S249. ESI mass spectrum of **81** under $H_2{}^{18}O$ conditions. ESI-MS calcd. for $C_{60}H_{89}N_{15}O_{17}{}^{18}O$ $[M+H]^+$ m/z = 1310.6, found 1310.3; $[M+2H]^{2+}$ m/z = 655.8, found 655.8.

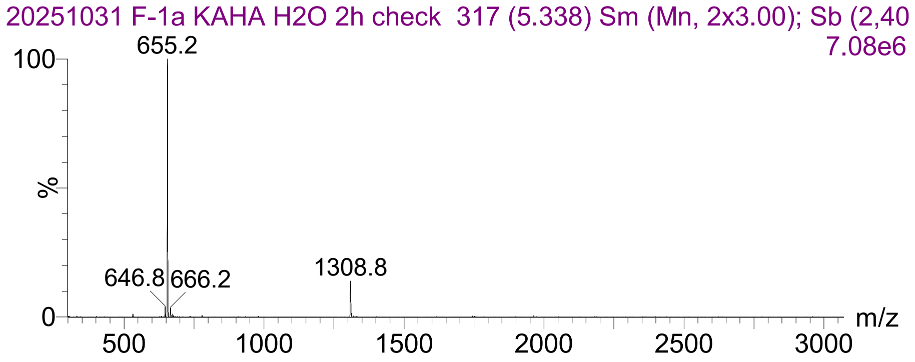

Figure S250. ESI mass spectrum of **81** under H_2O conditions. ESI-MS calcd. for $C_{60}H_{89}N_{15}O_{18}$ $[M+H]^+$ m/z = 1308.7, found 1308.8; $[M+2H]^{2+}$ m/z = 654.8, found 655.2.

7.3.3. Investigation of amide side product formation under isotopic labeling conditions

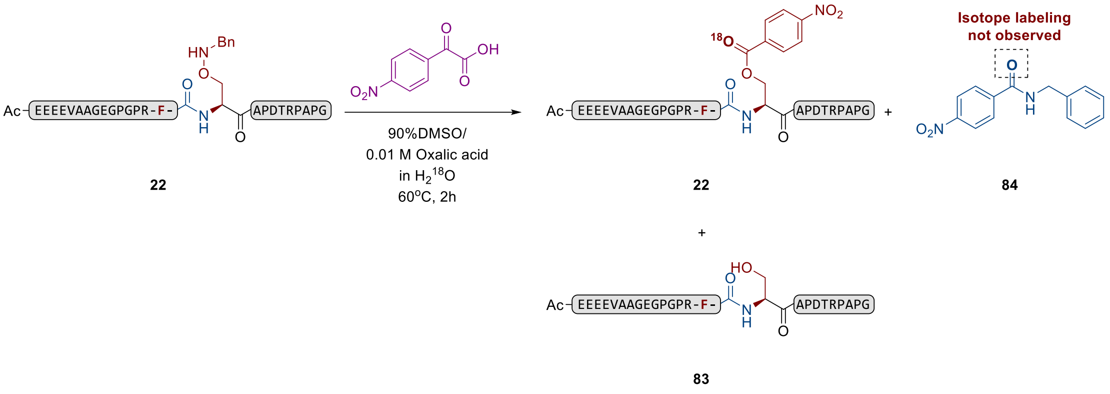

Scheme S16. Isotopic labeling of **22** under CEL conditions with $H_2{}^{18}O$.

Following the general CEL method, Ac-EEEEVAAGEGPGPRG(AO, Bn)APDTRPAPG-OH **22** (1.0 equiv.) was incubated with 2-(4-Nitrophenyl)-2-oxoacetic acid (5 equiv.) in aqueous DMSO with 0.01M oxalic acid dissolved in $H_2{}^{18}O$ at 40 mM, 60°C. Small amount of reaction mixture was aliquot at 2 h and was analyzed by UPLC.

NO2

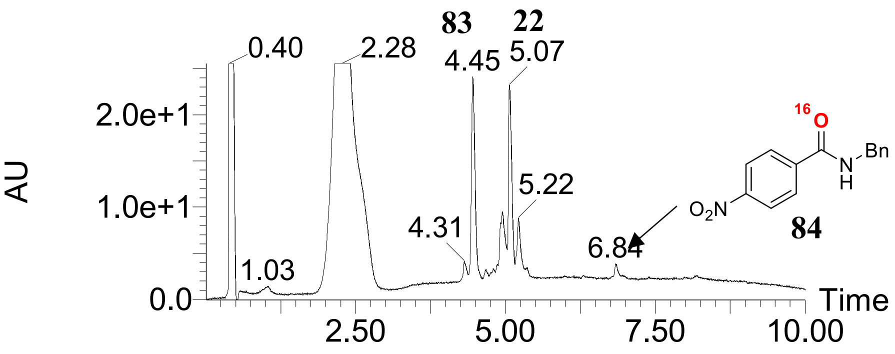

Figure S251. UV (190-400 nm) trace from UPLC-MS analysis of reaction of Ac-EEEEVAAGEGPGPRG(AO, Bn)APDTRPAPG-OH **22** with 2-(4-

Nitrophenyl)-2-oxoacetic acid in $H_2{}^{18}O$ at 2 h gradient 10-75% CH_3CN/H_2O containing 0.1% TFA over 10 min at a flow rate of 0.4 mL/min.

NO2

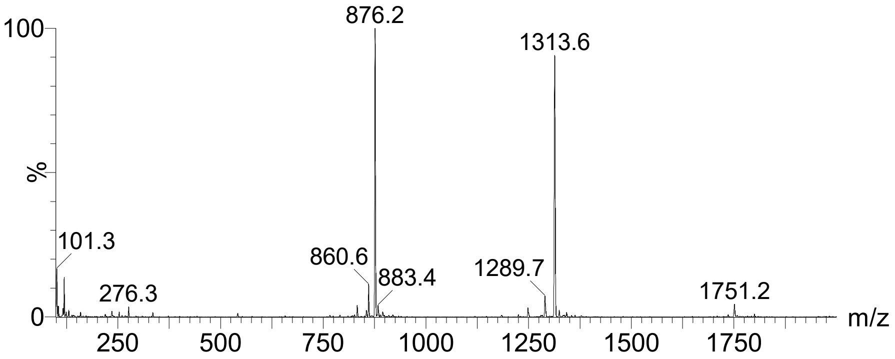

Figure S252. ESI mass spectrum of **22** under $H_2{}^{18}O$ conditions. ESI-MS calcd. for $C_{109}H_{162}N_{32}O_{43}{}^{18}O$ $[M+2H]^{2+}$ m/z = 1313.6, found 1313.6; $[M+3H]^{3+}$ m/z = 876.0, found 876.2.

NO2

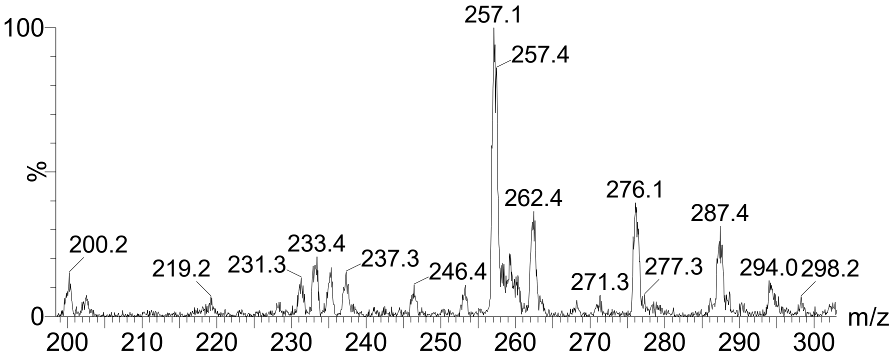

Figure S253. ESI mass spectrum of **84** under $H_2{}^{18}O$ conditions. ESI-MS calcd. for $C_{14}H_{12}N_2O_3$ $[M+H]^+$ m/z = 257.1, found 257.1.

7.3.4. Investigation of CEL applying steric hinder substrate 88

7.3.4.1. Synthesis of 88

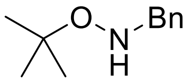

88

To a solution of O-(tert-Butyl)hydroxylamine hydrochloride (1 g, 7.96 mmol, 1.0 equiv.) dissolved in 25 mL of DMF, K_2CO_3 (4.4 g, 31.84 mmol, 4.0 equiv.) and benzyl bromide (1.3 mL, 10.59 mmol, 1.33 equiv.) were added to the reaction mixture at room temperature and stirred for overnight. After that, the reaction mixture was quenched with water (50 mL) and diluted with 200 mL of EA, and then washed with water (100.0 mL) x 2, and brine subsequently. The organic layer was dried with sodium sulfate and removed by reduced pressure evaporation. The residue was purified by silica gel chromatography (Hexane/EtOAc, 20:1) to give the desired product **88** (595 mg, 41.7%) as a pale yellow oil.

7.3.4.2. CEL of 88

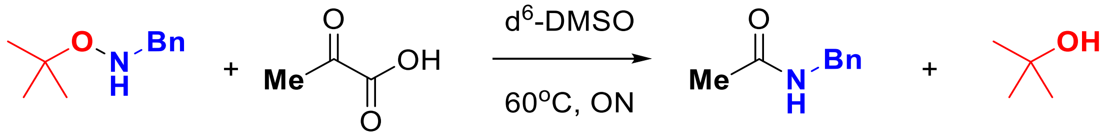

88 **87**

Scheme S17. Investigation of CEL with steric hinder aminooxy substrate **88**

Following the general CEL method, compound **88** was incubated with pyruvic acid (5 equiv.) in d^6-DMSO at 0.2 M, 60°C. Small amount of reaction mixture was aliquot at 6 h and was analyzed by UPLC and the reaction crude was directly analyzed by NMR after 14 h.

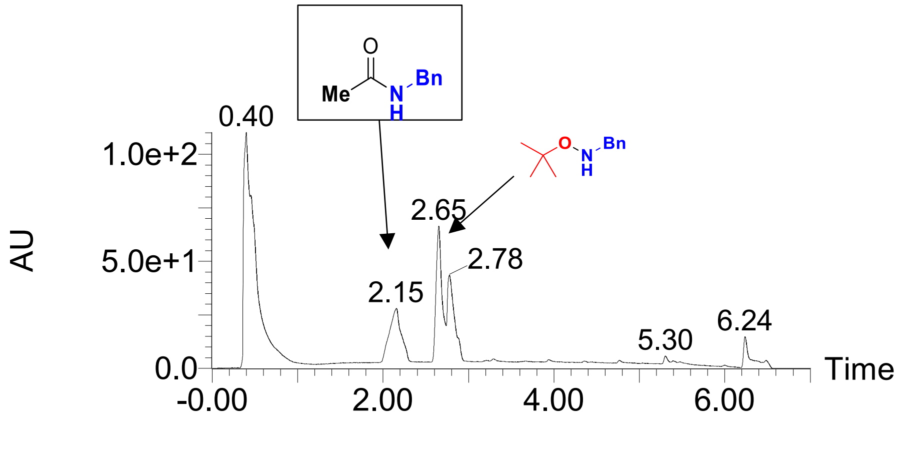

Figure S254. UV (190-400 nm) trace from UPLC-MS analysis of reaction of

compound **88** with pyruvic acid at 6 h gradient 40-95% CH_3CN/H_2O containing 0.1% TFA over 7 min at a flow rate of 0.4 mL/min.

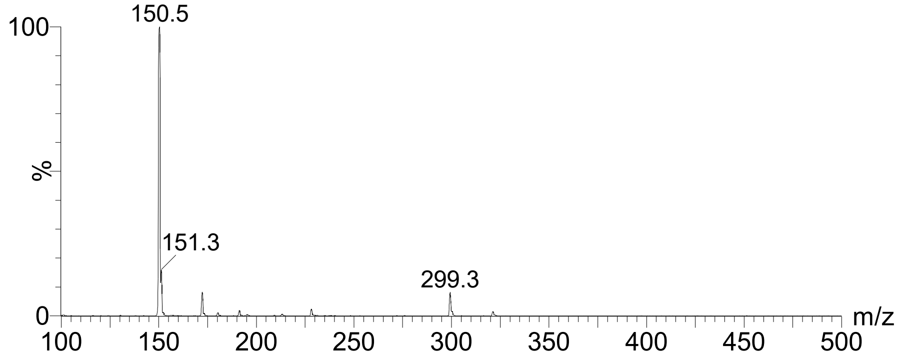

Figure S255. ESI mass spectrum of **87**. ESI-MS calcd. for $C_9H_{11}NO$ $[M+H]^+$ m/z = 150.1, found 150.5.

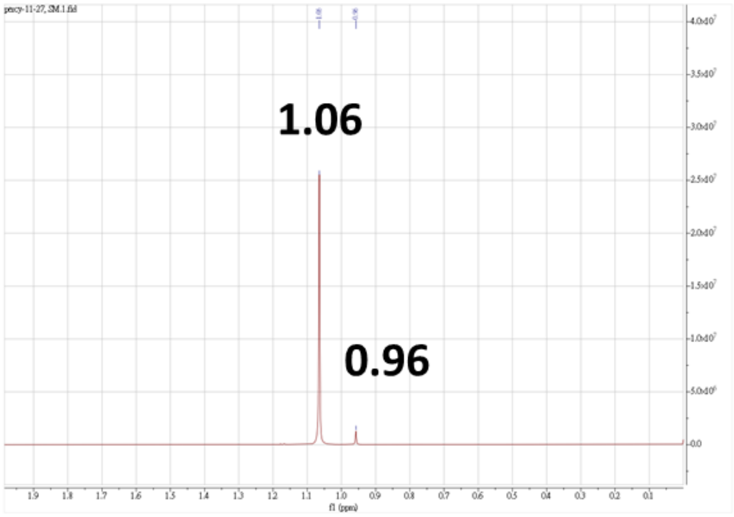

Figure S256. ^1H NMR analysis of reaction of compound **88** with pyruvic acid at 0 h.

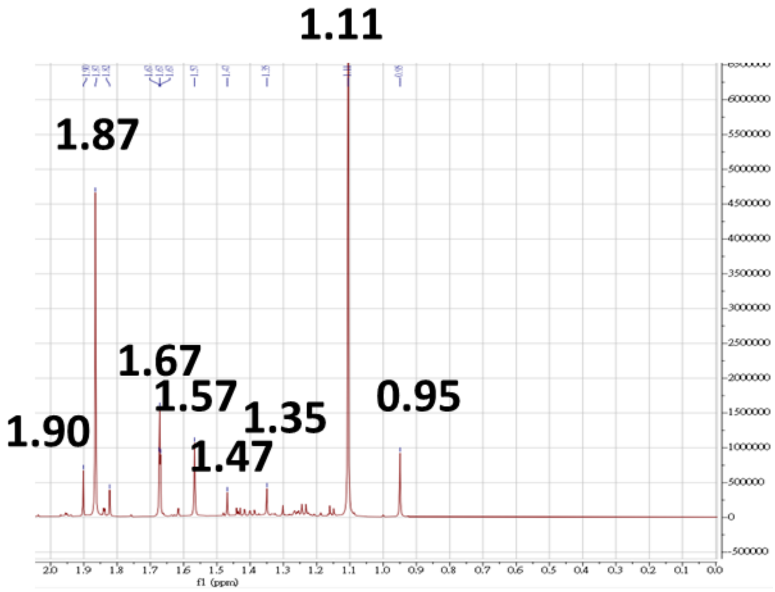

Figure S257. ^{1}H NMR analysis of reaction of compound **88** with pyruvic acid after overnight.

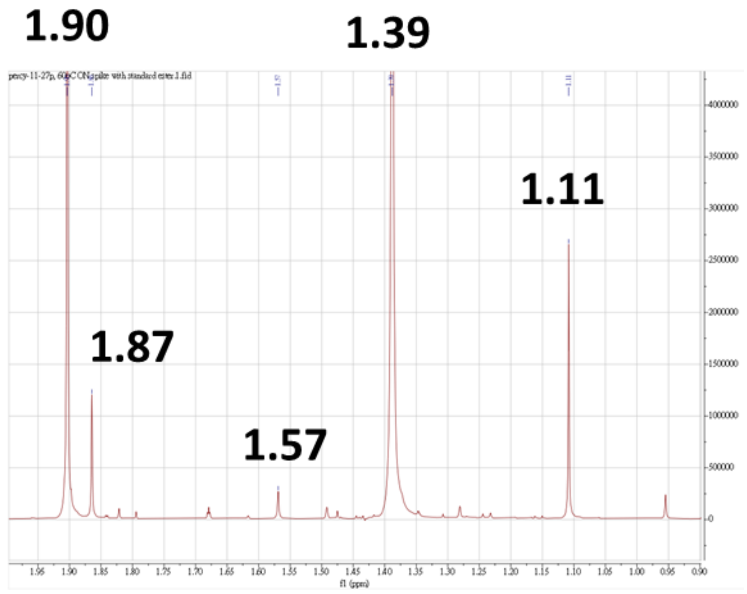

Figure S258. ^{1}H NMR analysis of reaction of compound **88** with pyruvic acid after overnight spike with tert-butyl acetate as reference.

8. NMR spectra

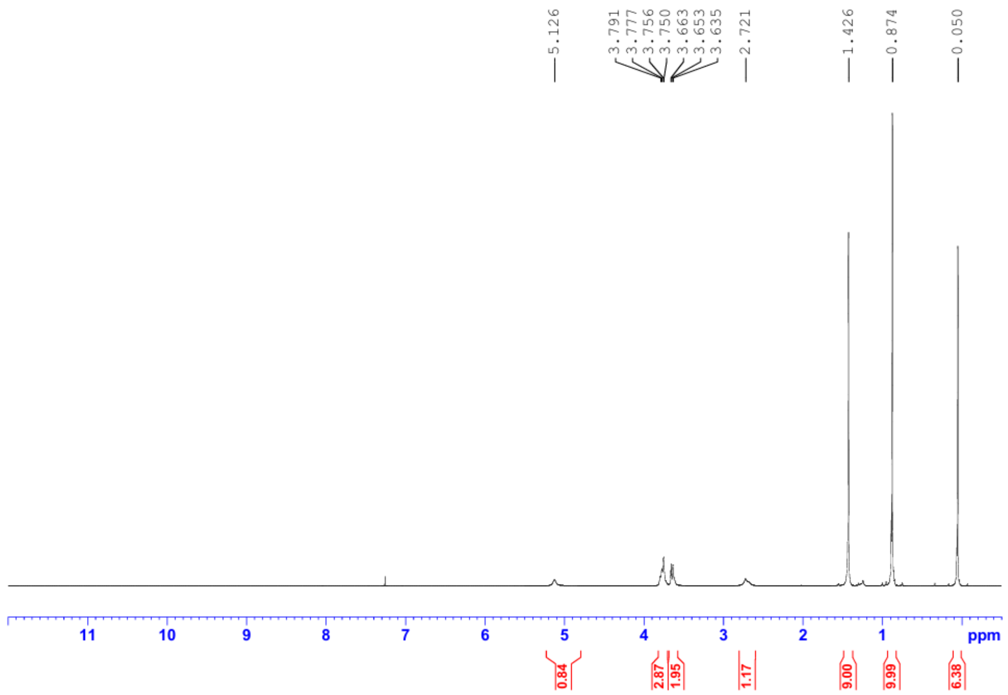

Figure S259. ^{1}H NMR spectrum of **S1**.

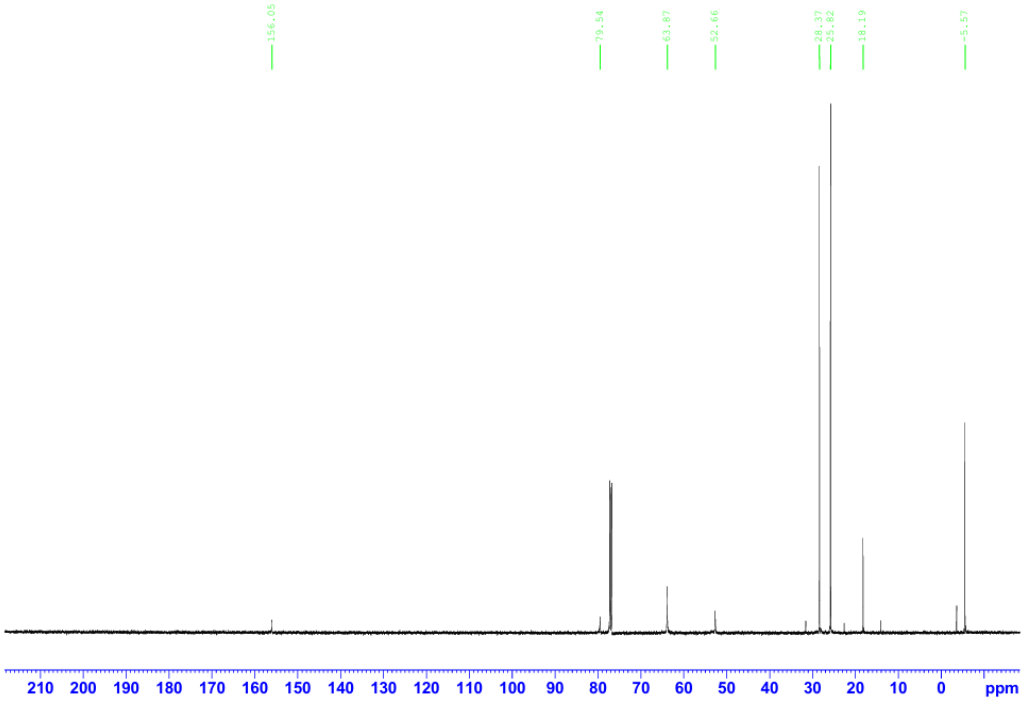

Figure S260. ^{13}C NMR spectrum of **S1**.

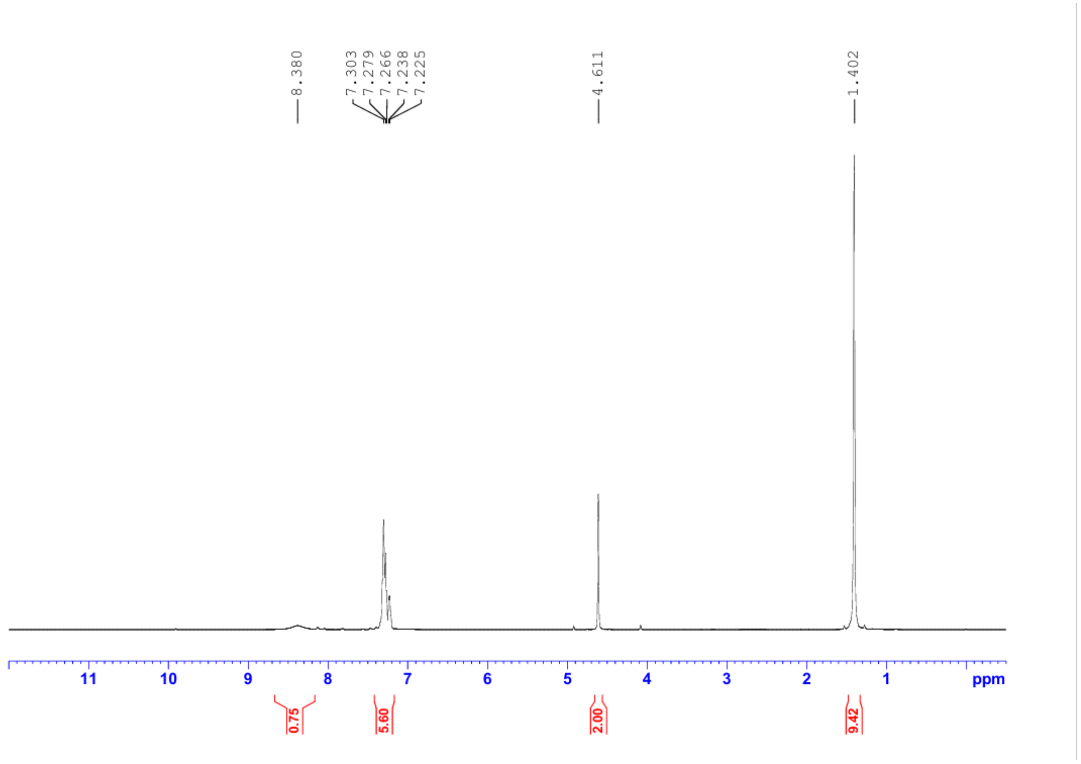

Figure S261. ^{1}H NMR spectrum of **S3a**.

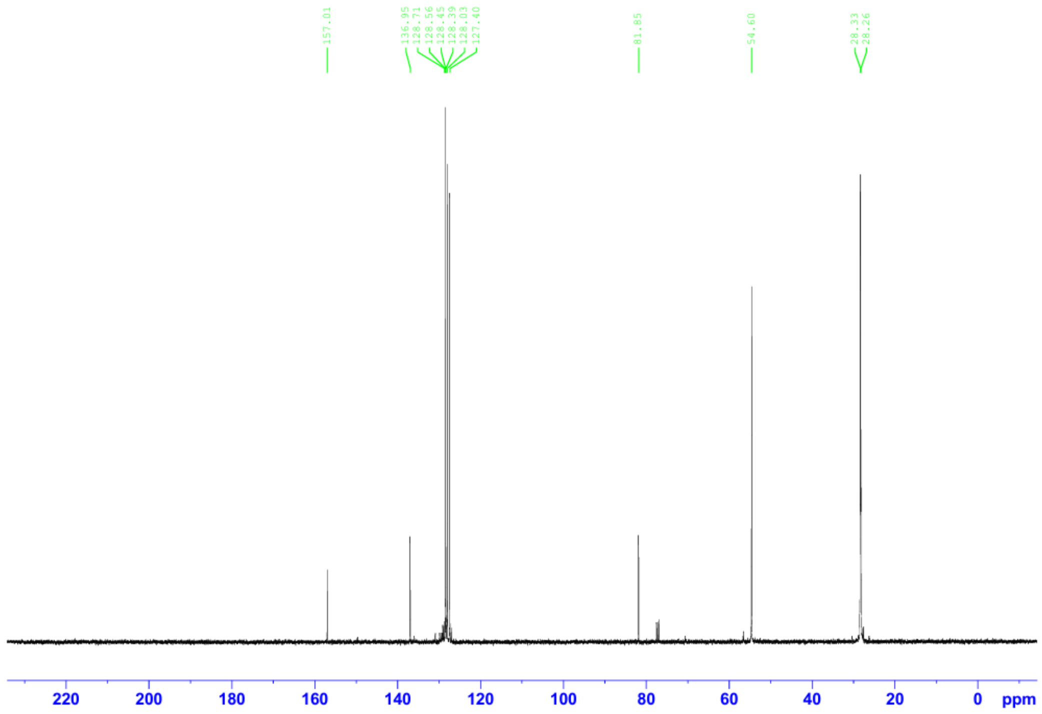

Figure S262. ^{13}C NMR spectrum of **S3a**.

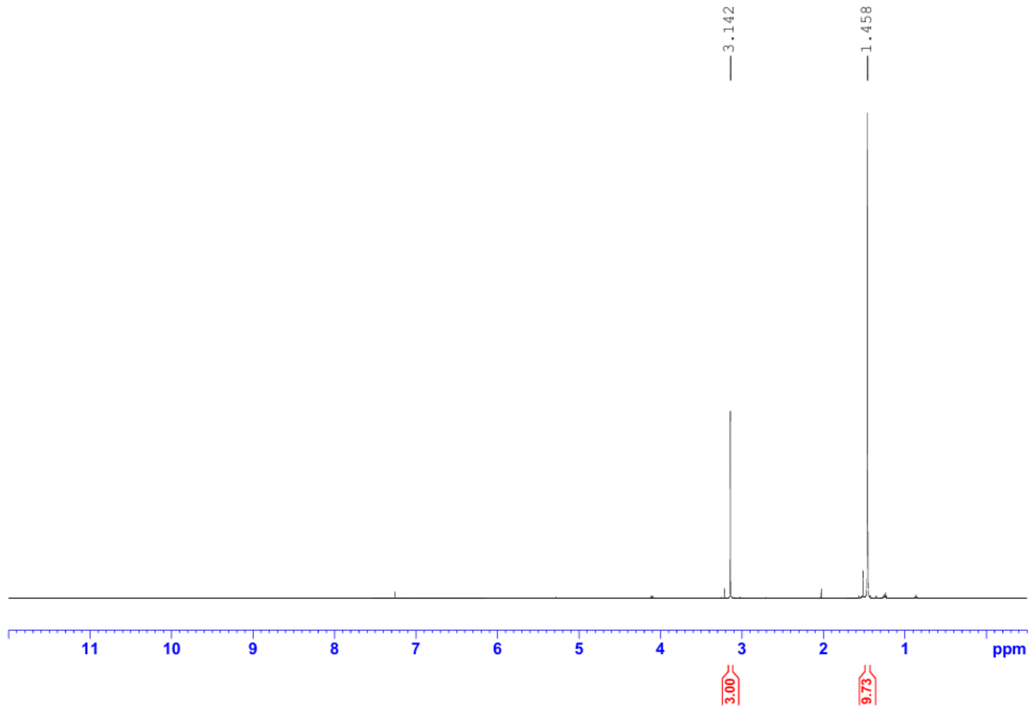

Figure S263. ¹H NMR spectrum of **S3b**.

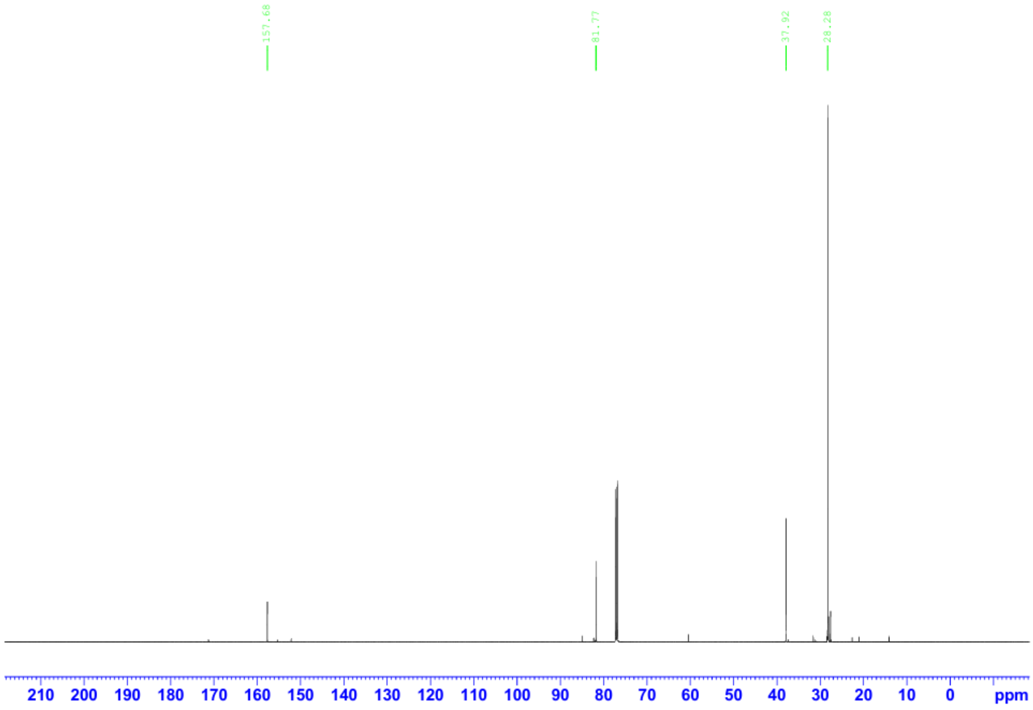

Figure S264. ¹³C NMR spectrum of **S3b**.

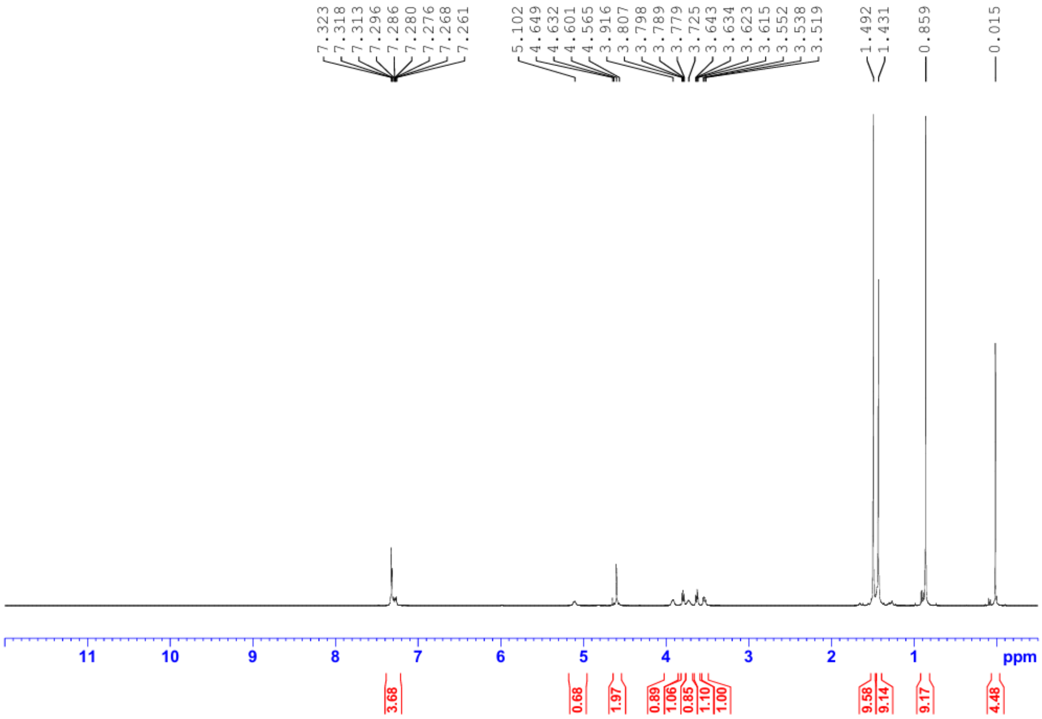

Figure S265. ^1H NMR spectrum of **S4a**.

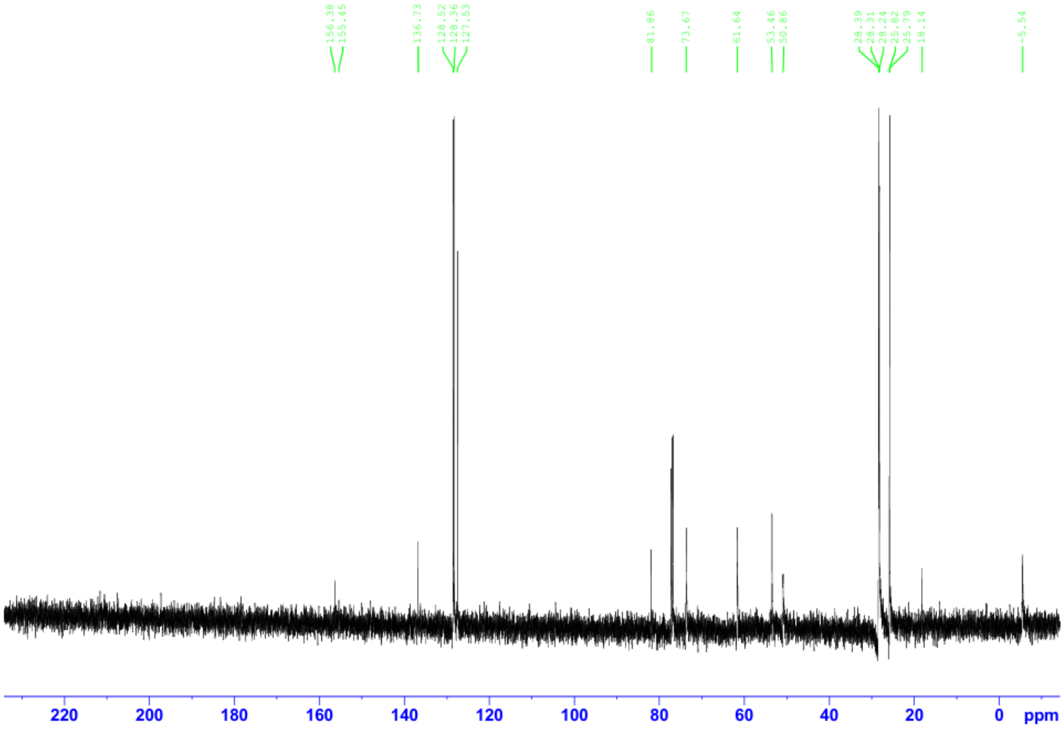

Figure S266. ^{13}C NMR spectrum of **S4a**.

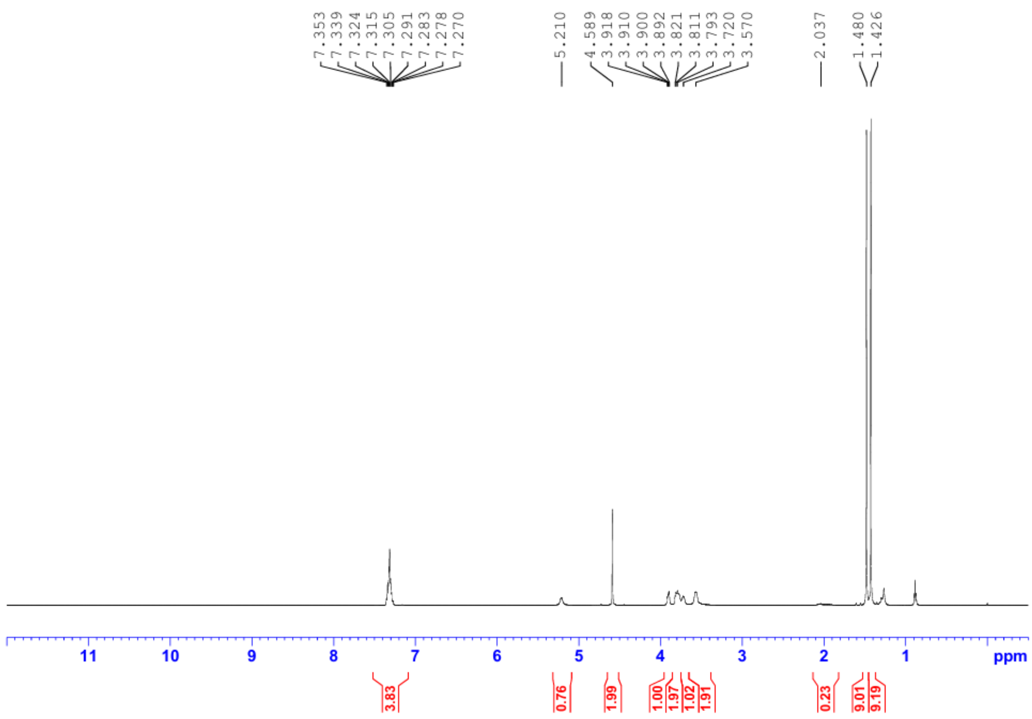

Figure S267. ¹H NMR spectrum of **S5a**.

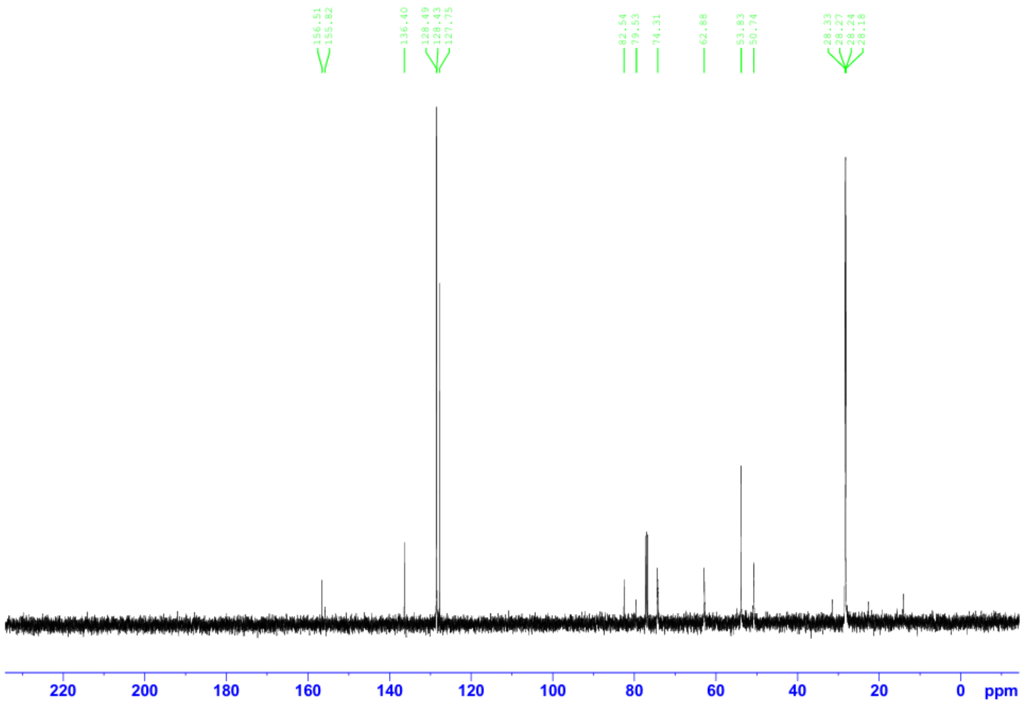

Figure S268. ^{13}C NMR spectrum of **S5a**.

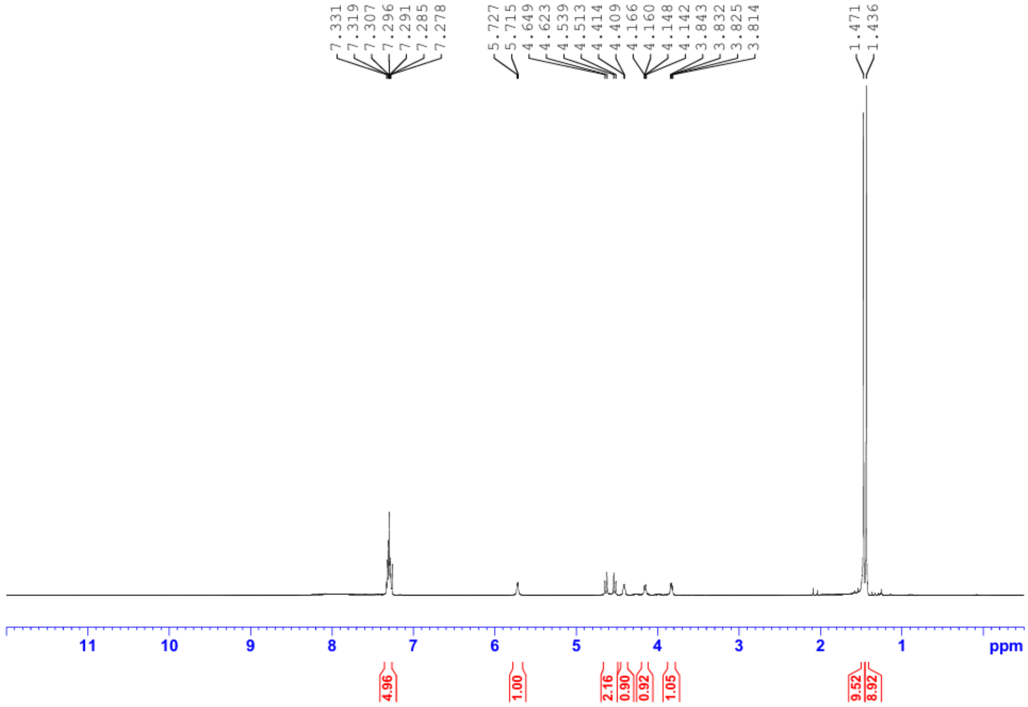

Figure S269. ^{1}H NMR spectrum of **S6a**.

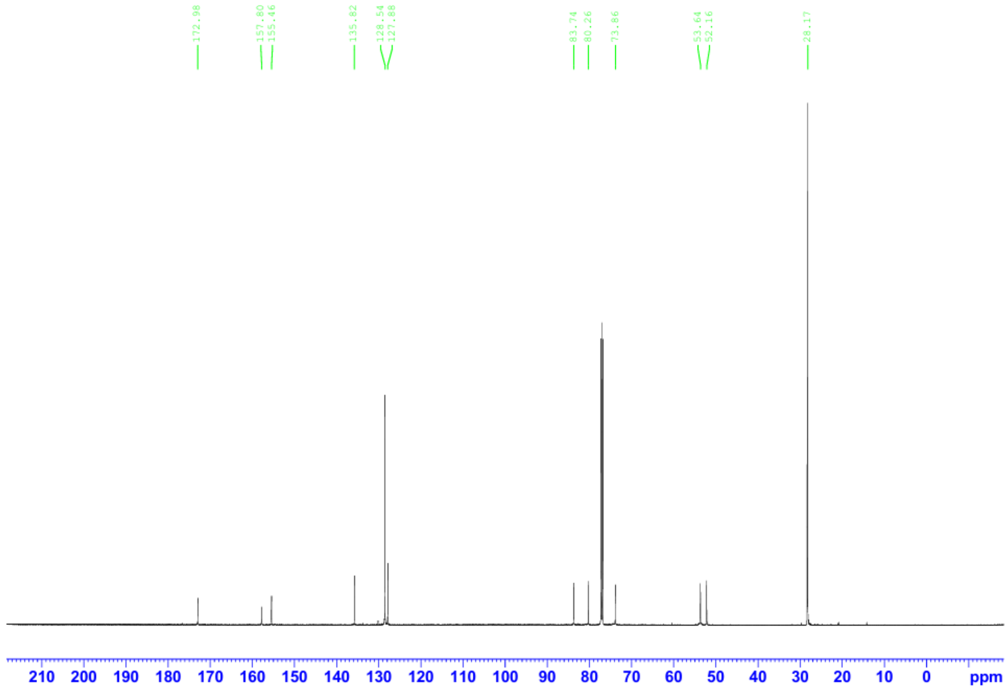

Figure S270. ^{13}C NMR spectrum of **S6a**.

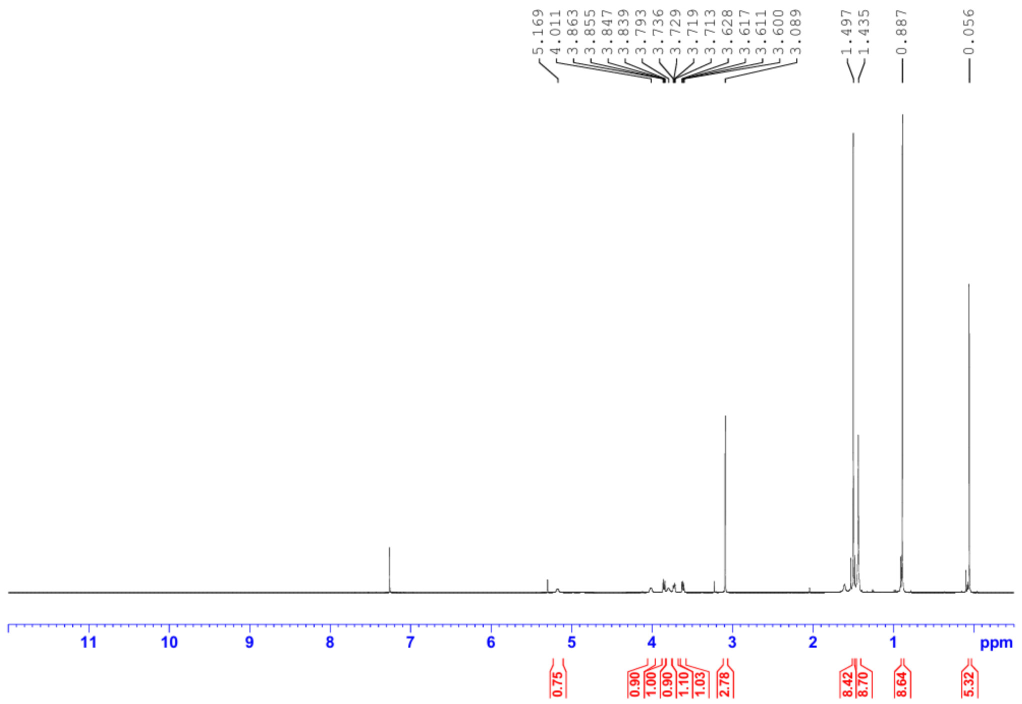

Figure S271. ^{1}H NMR spectrum of **S4b**.

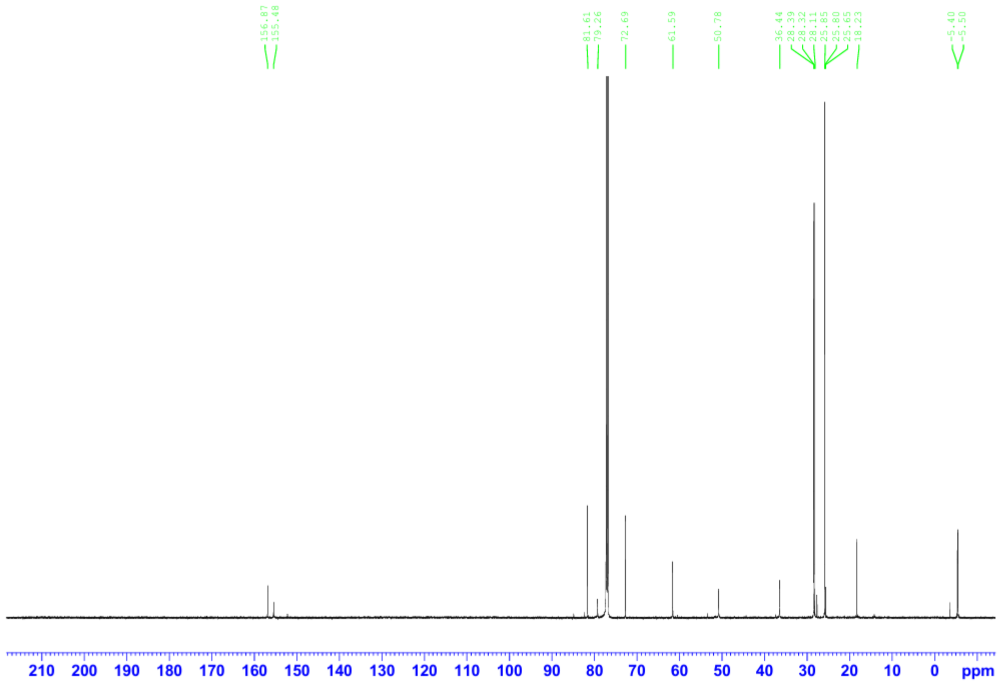

Figure S272. ^{13}C NMR spectrum of **S4b**.

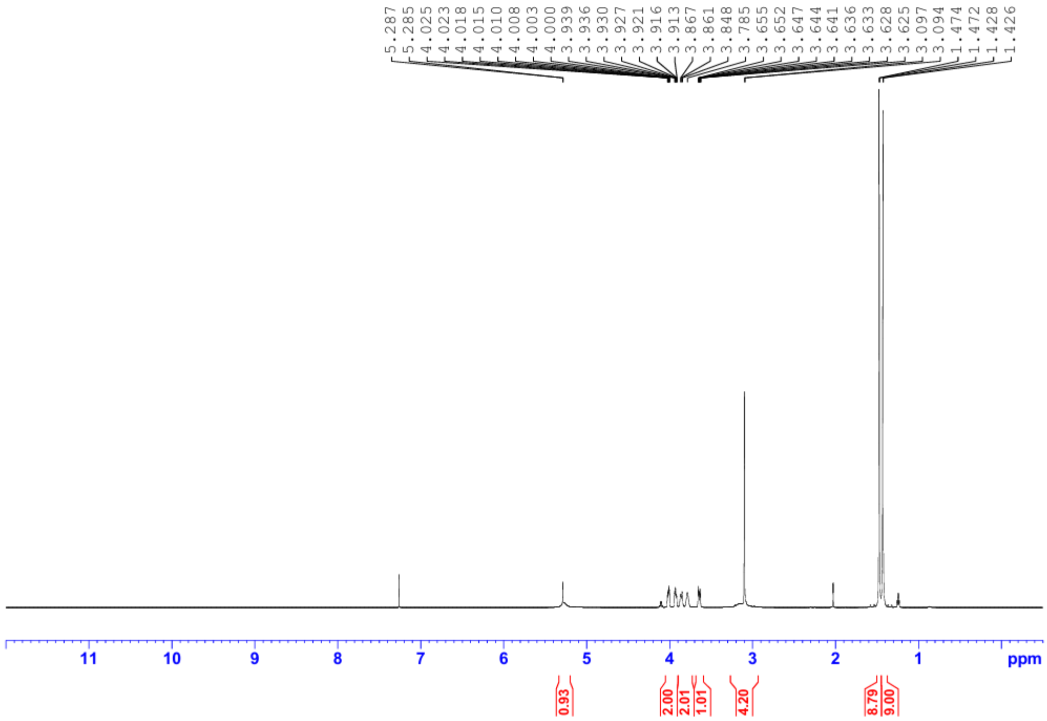

Figure S273. ^{1}H NMR spectrum of **S5b**.

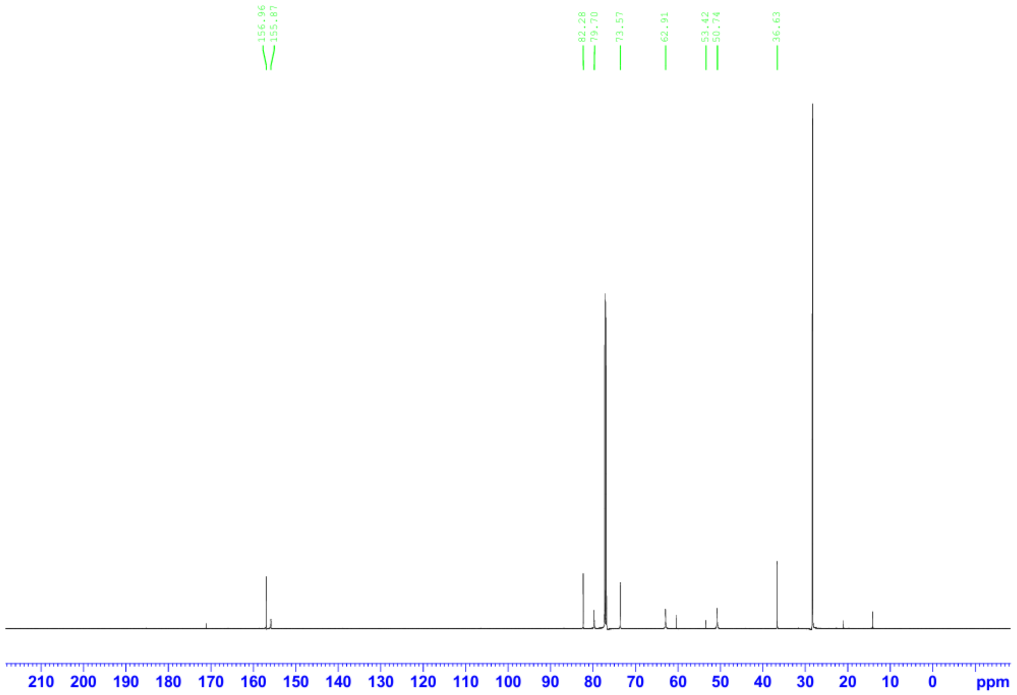

Figure S274. ^{13}C NMR spectrum of **S5b**.

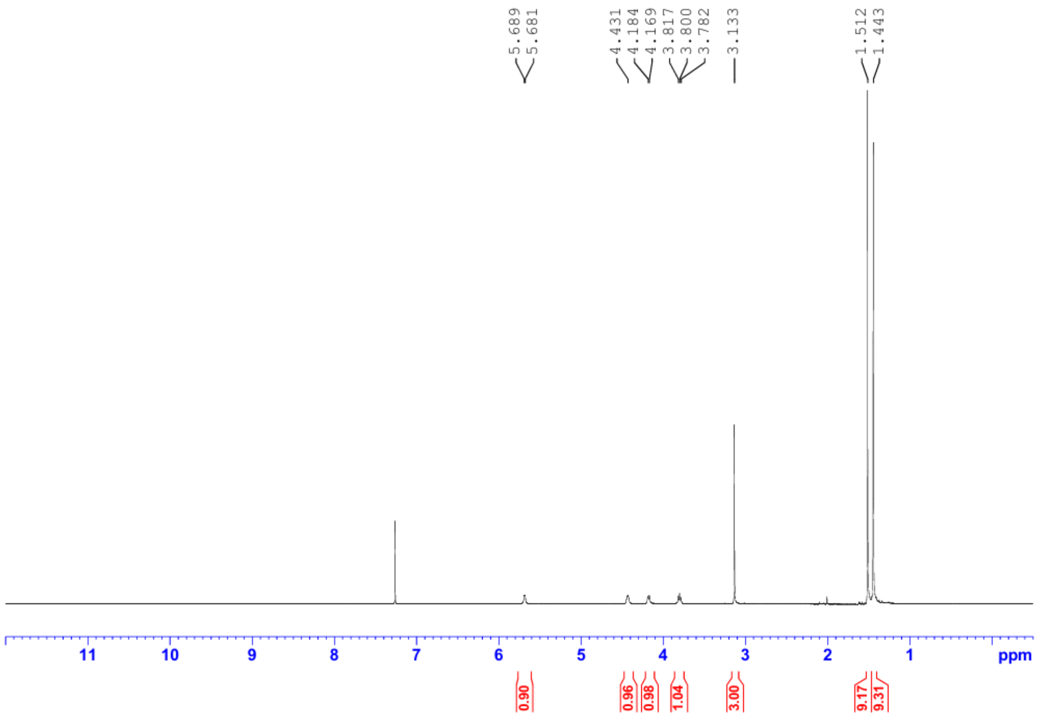

Figure S275. ^{1}H NMR spectrum of **S6b**.

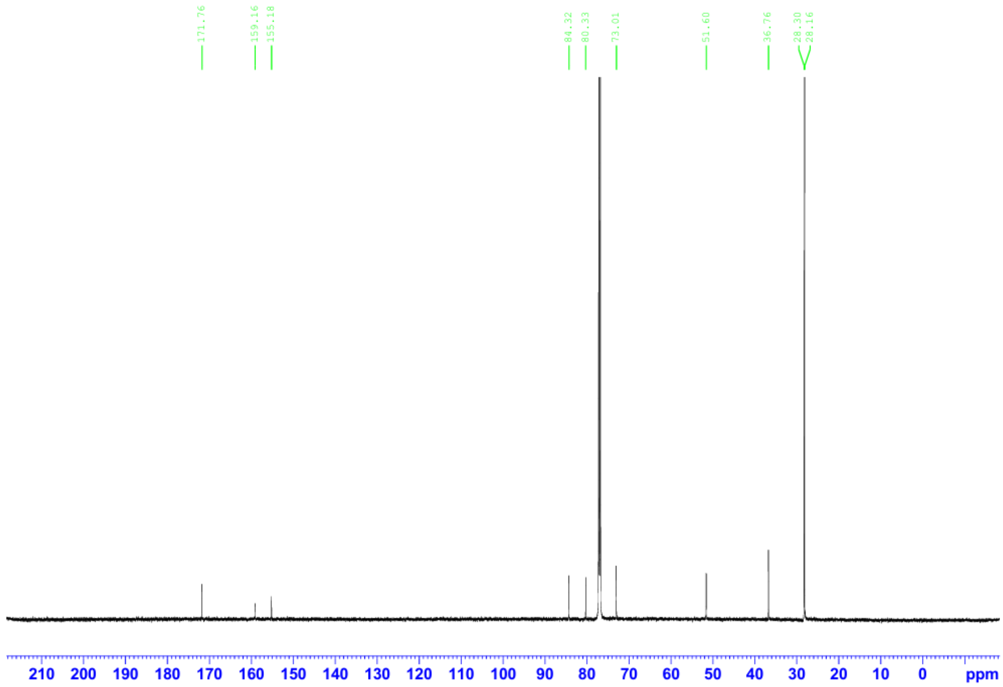

Figure S276. ^{13}C NMR spectrum of **S6b**.

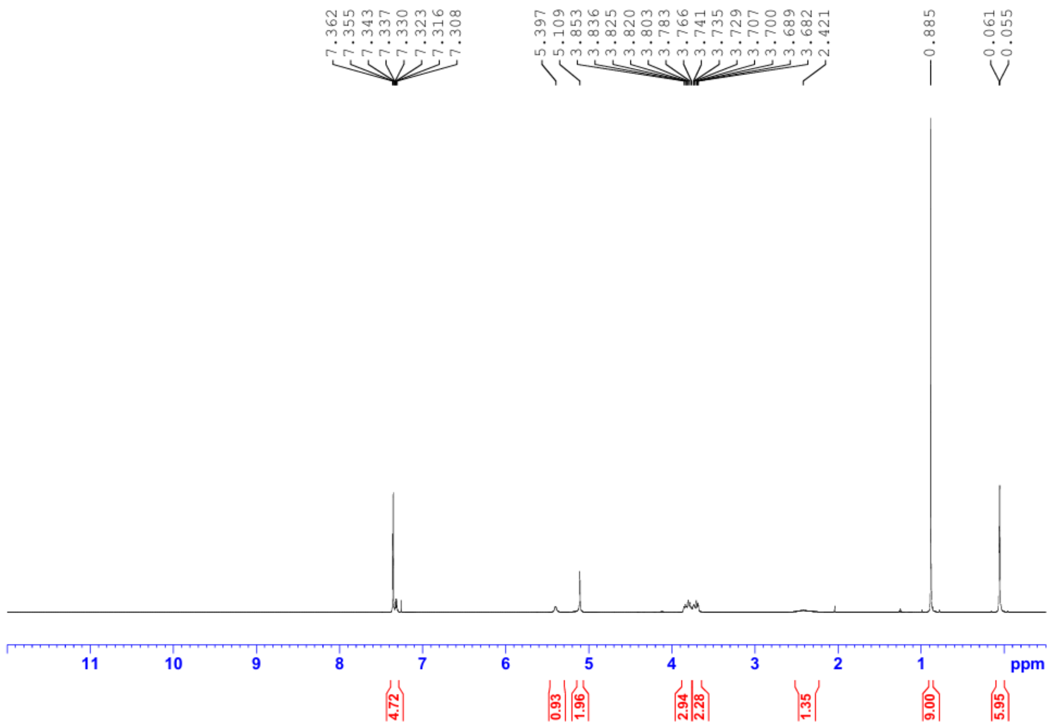

Figure S277. ^{1}H NMR spectrum of **S7**.

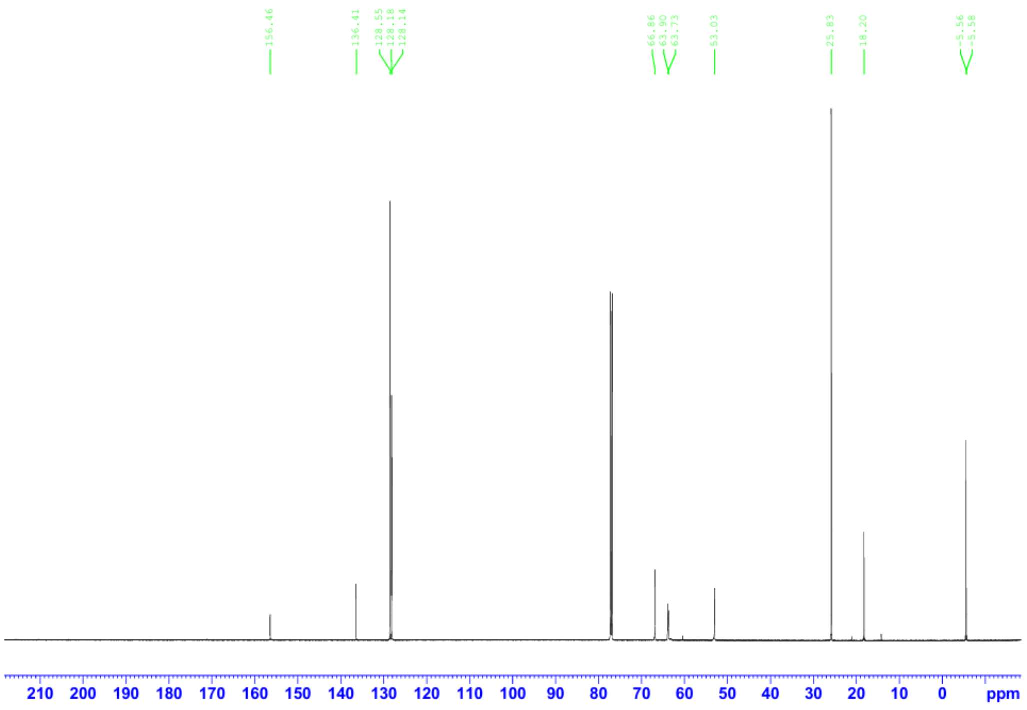

Figure S278. ^{13}C NMR spectrum of **S7**.

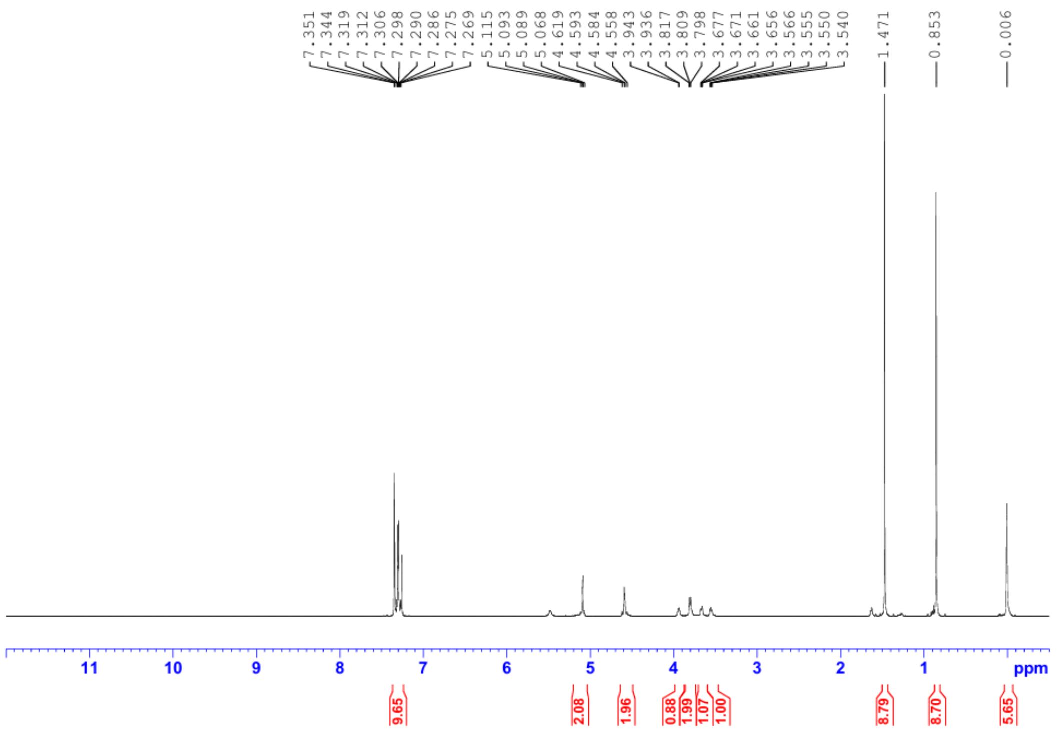

Figure S279. ^{1}H NMR spectrum of **S9**.

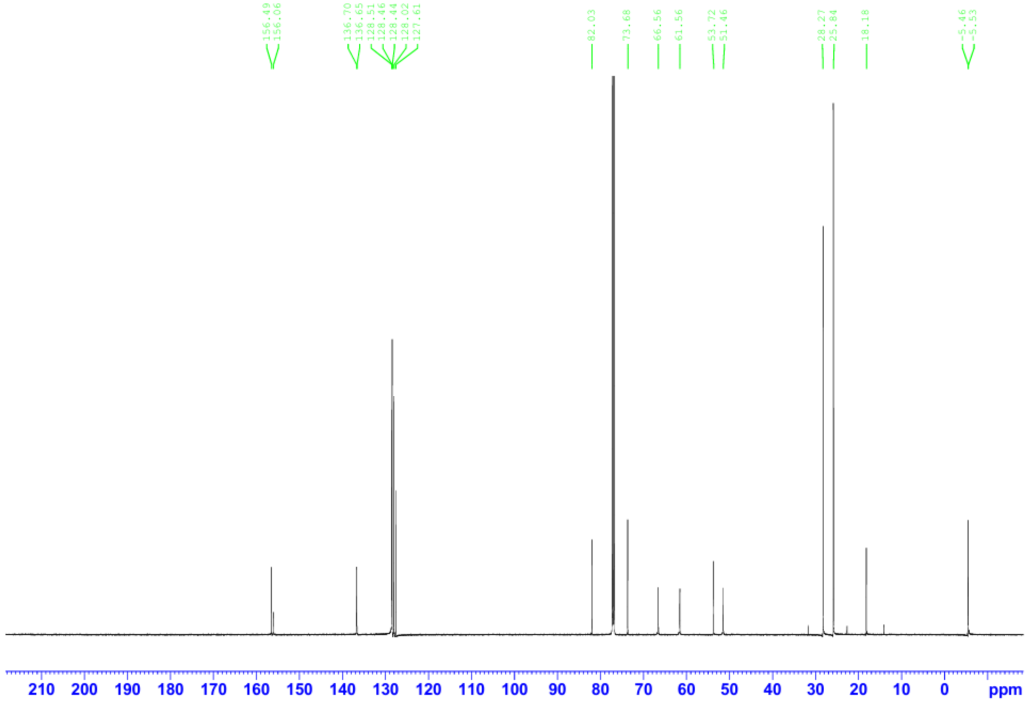

Figure S280. ^{13}C NMR spectrum of **S9**.

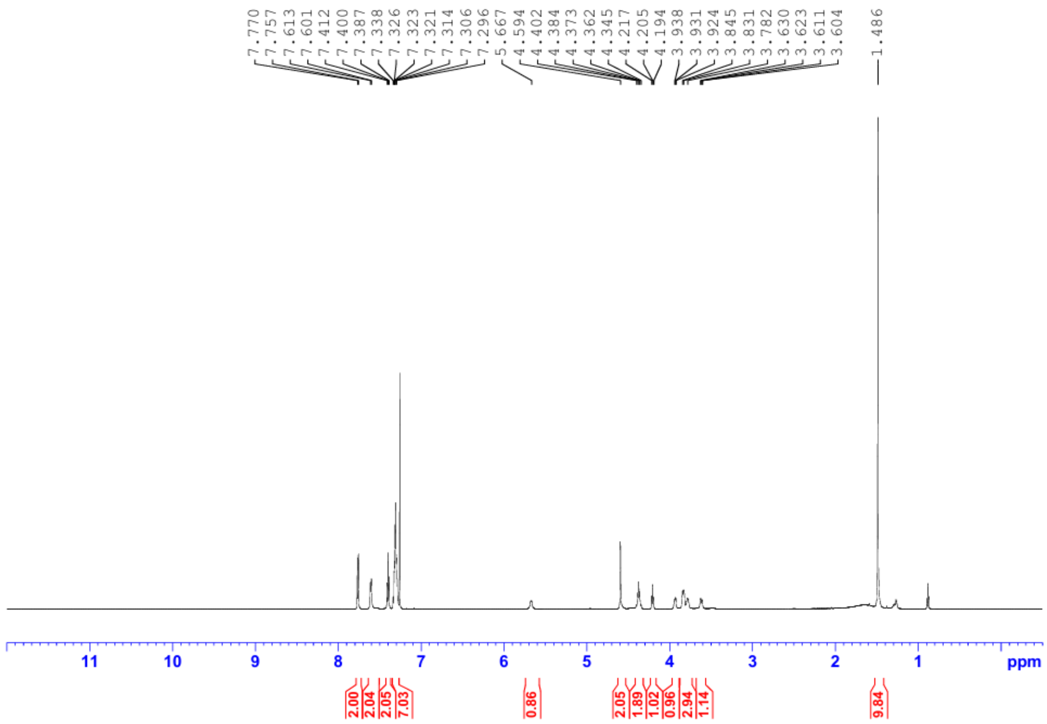

Figure S281. ^{1}H NMR spectrum of **S10**.

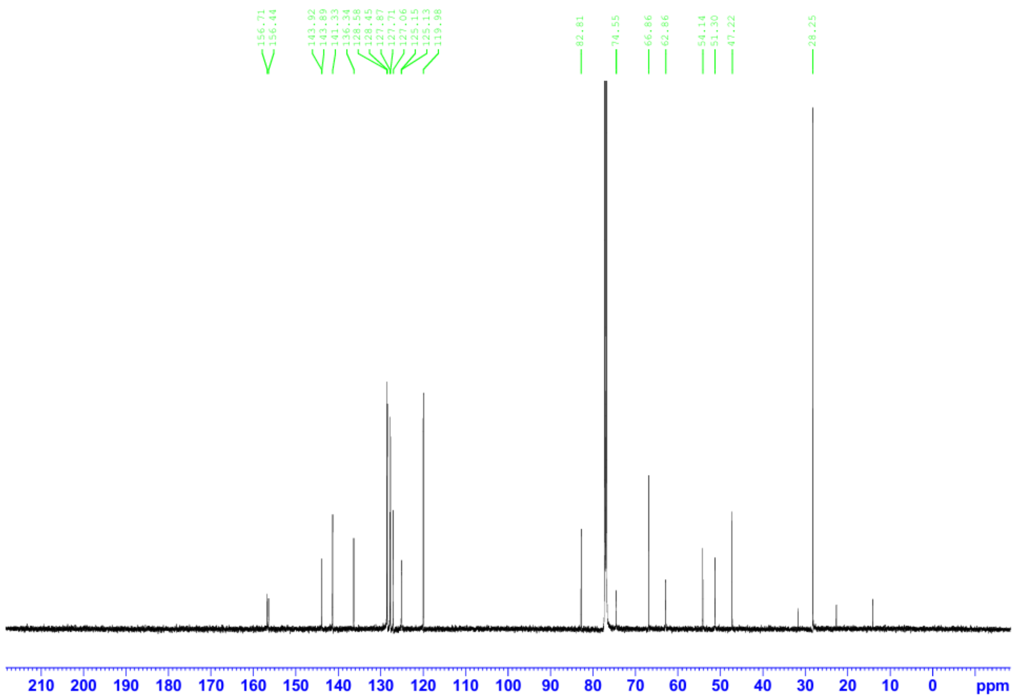

Figure S282. ^{13}C NMR spectrum of **S10**.

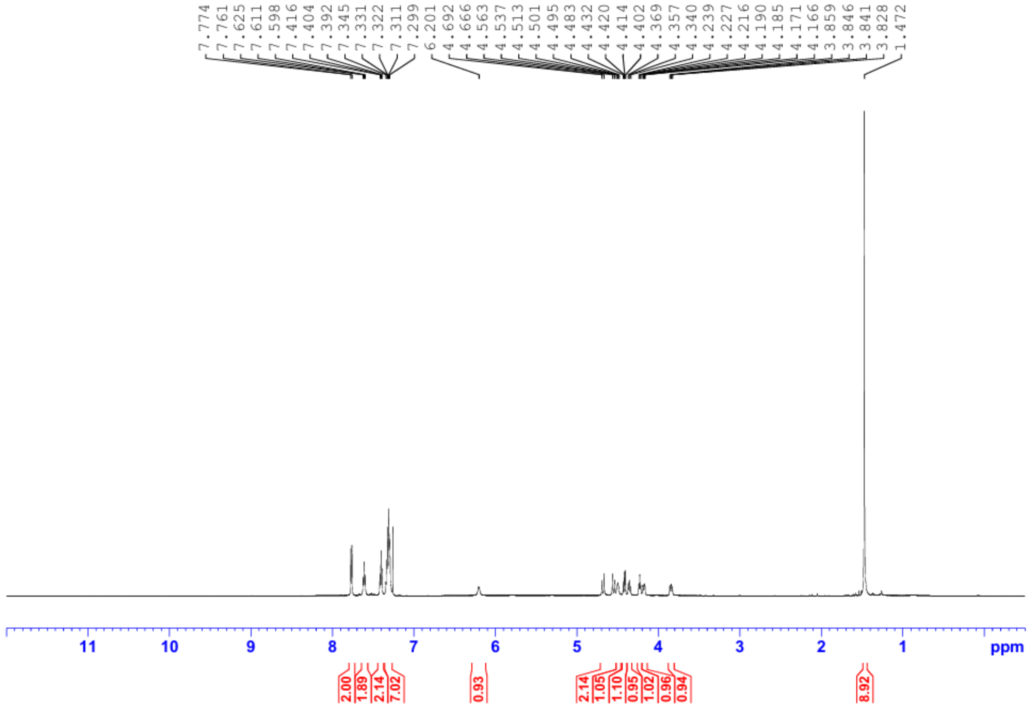

Figure S283. ^{1}H NMR spectrum of **S11**.

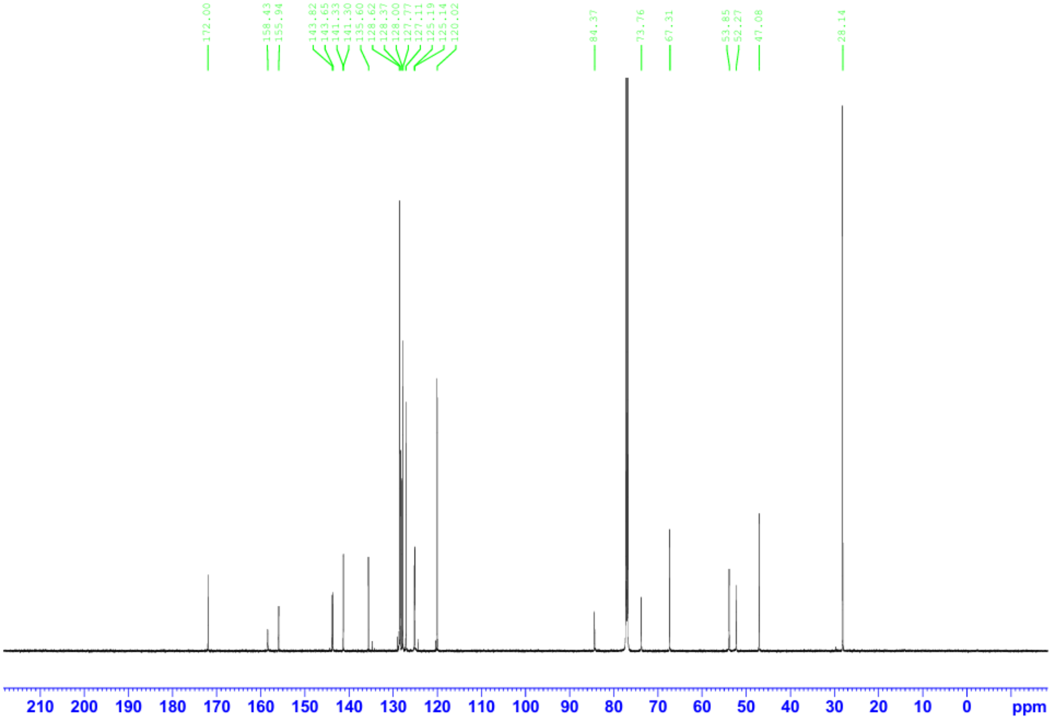

Figure S284. ^{13}C NMR spectrum of **S11**.

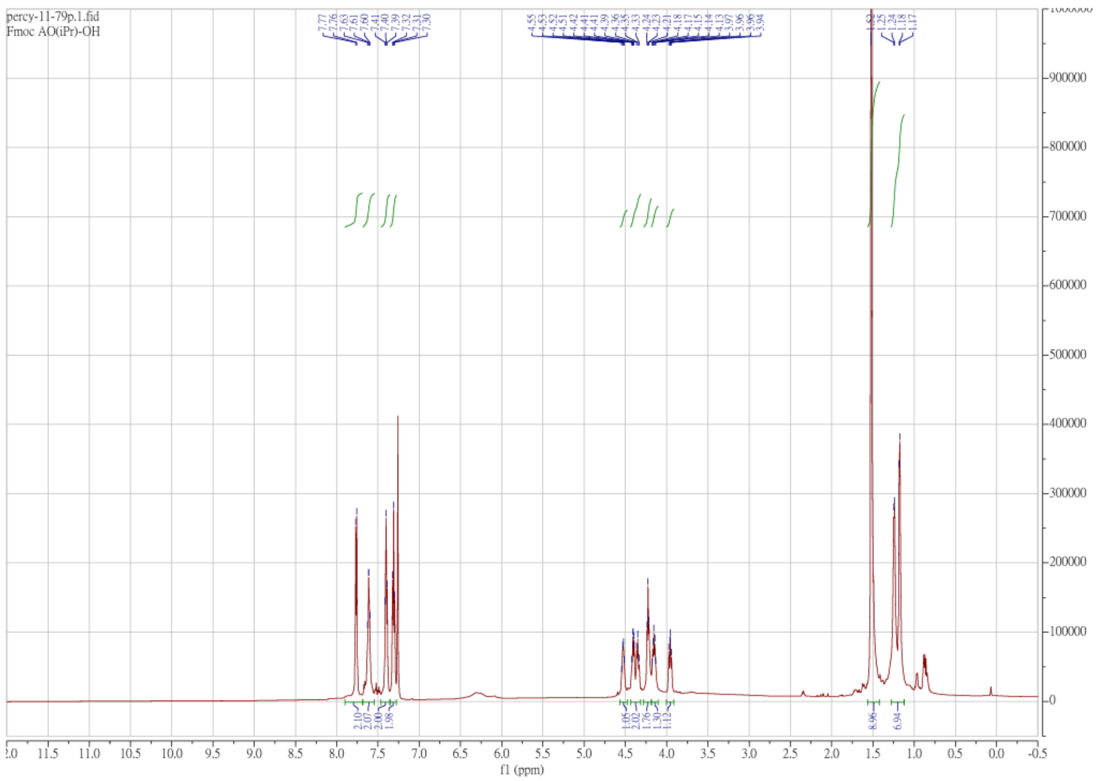

Figure S285. ^{1}H NMR spectrum of **S12**.

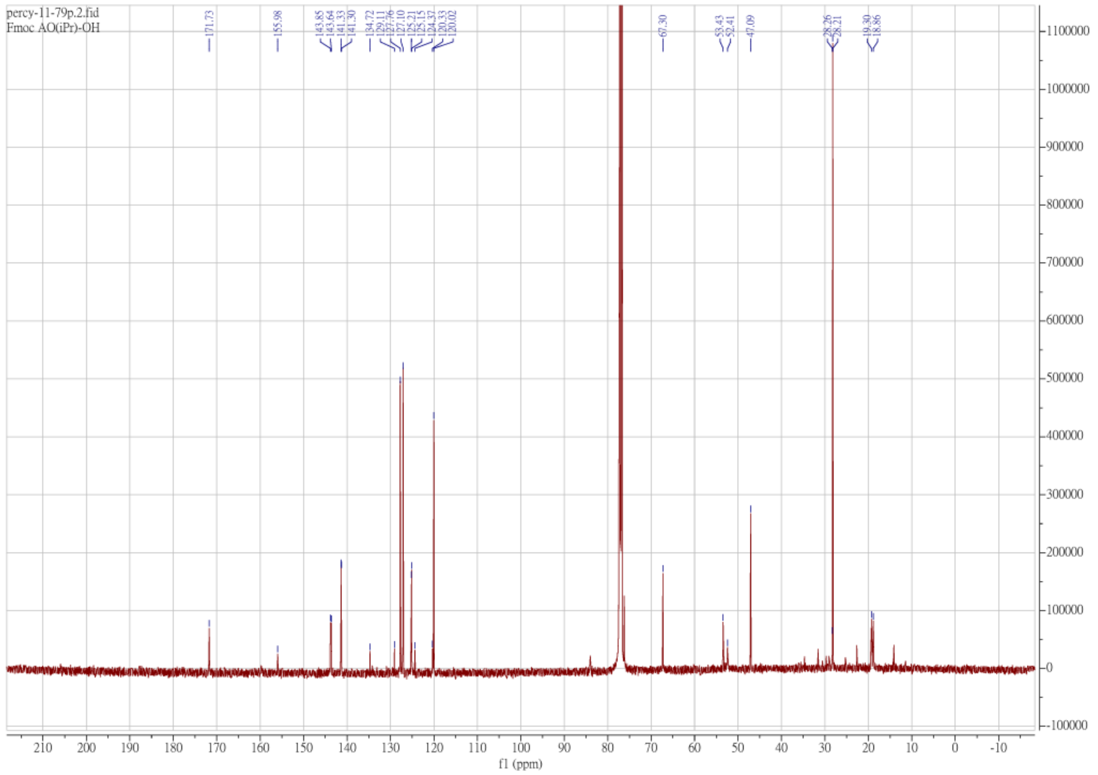

Figure S286. ^{13}C NMR spectrum of **S12**.

9. Reference

1. Lee, C. L., Liu, H., Wong, C. T. T., Chow, H. Y. & Li, X. Enabling N-to-C Ser/Thr Ligation for Convergent Protein Synthesis via Combining Chemical Ligation Approaches. *J. Am. Soc. Chem.* **138**, 10477–10484 (2016).
2. Zheng, J.-S., Tang, S., Qi, Y.-K., Wang, Z.-P. & Liu, L. Chemical synthesis of proteins using peptide hydrazides as thioester surrogates. *Nat. Protoc.* **8**, 2483–2495 (2013).
3. Dunkelmann, D. *et al.* Amide-forming chemical ligation via O-acyl hydroxamic acids. *Proc. Natl. Acad. Sci. U.S.A* **115**, 3752–3757 (2018).
4. Flood, D. T. *et al.* Leveraging the Knorr Pyrazole Synthesis for the Facile Generation of Thioester Surrogates for use in Native Chemical Ligation. *Angew. Chem. Int. Ed.* **57**, 11634–11639 (2018).
5. Harmand, T. J., Murar, C. E. & Bode, J. W. Protein chemical synthesis by α-ketoacid–hydroxylamine ligation. *Nat. Protoc.* **11**, 1130–1147 (2016).
